# The pivotal regulatory role of the FEV-SLC7A11 axis in ferroptosis elucidates the anti-aging mechanism of β-sitosterol in a cross-species study

**DOI:** 10.3389/fphar.2025.1600489

**Published:** 2025-08-07

**Authors:** Zhihan Fang, Liyao Xie, Jing Wang, Junyi Li, Yirui Pan, Lei Chen, Shuyi Cao, Qi Zhou, Shaobin Li, Chao Zhang, Li Li

**Affiliations:** ^1^ Department of Pathogenic Biology and Key Laboratory of Tropical Translational Medicine of Ministry of Education, School of Basic Medicine and Life Sciences, Hainan Medical University, Haikou, China; ^2^ Department of Biochemistry and Molecular Biology, School of Basic Medical Sciences, Centre for Experimental Management, Southern Medical University, Guangzhou, China; ^3^ The First School of Clinical Medicine, Southern Medical University, Guangzhou, China; ^4^ Department of Cardiology, The Fifth Affiliated Hospital of Southern Medical University, Guangzhou, China; ^5^ Department of Thoracic Surgery, Nanfang Hospital, Southern Medical University, Guangzhou, China

**Keywords:** β-sitosterol, ferroptosis, oxidative stress, cellular senescence, FEV, cross-species comparison

## Abstract

Traditional Chinese medicine is a valuable source of bioactive compounds for combating aging. Among these, β-sitosterol (BS), a sterol extracted from *Alpiniae oxyphyllae fructus*, has attracted attention for its potent antioxidant, anti-inflammatory, and free radical scavenging properties. However, its precise anti-aging mechanism remains unclear. Here, we aimed to elucidate how BS influences cellular and murine aging. Preliminary studies in *Caenorhabditis elegans* (*C. elegans*) showed that BS modulates intracellular oxidative stress via the transcription factor ETS-5. Building on this, we established an aging model in human umbilical vein endothelial cells by treating them with 200 μM H_2_O_2_, assessing senescence via β-galactosidase staining and oxidative stress by measuring reactive oxygen species, malondialdehyde, and the GSH/GSSG ratio. Both *in vitro* and *in vivo* experiments revealed that BS treatment significantly alleviated oxidative stress, upregulated ferroptosis-related proteins, and suppressed ferroptosis to mitigate cellular senescence. Furthermore, RNA interference targeting *fev*, the human homolog of *ets-5*, reduced oxidative stress, and subsequent BS treatment further enhanced this protective effect. Dual luciferase assays indicated that FEV functions as a transcriptional repressor of SLC7A11; BS treatment altered FEV expression, thereby promoting SLC7A11 expression and facilitating the nuclear import of reduced glutathione. In summary, our results indicate that BS modulates FEV expression to regulate intracellular oxidative stress, suppress ferroptosis, and alleviate aging phenotypes. Our multi-model approach, integrating insights from *C. elegans*, human endothelial cells, and murine systems, substantially enhances the robustness and translational potential of these findings.

## 1 Introduction

With the global population aging at an unprecedented rate, there is an urgent need to unravel the mechanisms underlying aging and develop effective strategies to preserve health and quality of life in later years. Aging is characterized by the progressive deterioration of cellular, tissue, and organ functions, driven by a complex interplay of genetic, epigenetic, and environmental factors (López-Otín et al., 2013; Cai et al., 2022; Shi et al., 2022).

Ferroptosis, an iron-dependent form of cell death driven by unchecked lipid peroxidation, has recently been identified as a principal contributor to accelerated ageing and numerous age-related disorders (Mazhar et al., 2021). When cystine uptake via SLC7A11 falls, the GSH-GPX4 defence breaks down, allowing Fe^2+^ to boost ROS and lipid-peroxide build-up (Ma et al., 2022). The ensuing redox imbalance causes multi-organ injury: in the brain, iron deposition coupled with GPX4 loss eliminates hippocampal neurons and precipitates cognitive decline (Hambright et al., 2017; Majerníková et al., 2021); in the cardiovascular system, reduced SLC7A11 expression in vascular smooth-muscle cells and cardiomyocytes accelerates atherogenesis and cardiomyopathy (Fang et al., 2020; Wang et al., 2022); in the musculoskeletal system, ferroptosis disrupts the osteoblast–osteoclast equilibrium, thereby fostering osteoporosis and osteoarthritis (Wu et al., 2024; Cao et al., 2023); and in the ovary, age-related GPX4 depletion diminishes oocyte quality and curtails reproductive span (Hu et al., 2025). Taken together, these observations indicate that ferroptosis functions not merely as a biomarker but as a causal driver of age-associated functional deterioration. Consequently, small molecules that restore the SLC7A11–GPX4 axis or neutralise lipid radicals are being pursued as promising geroprotective interventions.

Traditional Chinese medicines (TCMs) offer multi-target, low-toxicity, and synergistic effects that align well with the complex biology of aging (Zhou et al., 2016; Jen et al., 2021). A representative example is Alpiniae oxyphyllae fructus (AOF, Yi Zhi Ren), a ginger-family fruit traditionally used to strengthen the spleen and kidneys and to slow age-related decline (Wang et al., 2015). Contemporary studies confirm that AOF and its compounds exert strong antioxidant, anti-inflammatory, and neuroprotective actions with documented anti-aging benefits (Yu et al., 2020). Building on these findings, we interrogated AOF’s chemical profile to pinpoint the molecules most responsible for its geroprotective effects. TCMSP filtering (oral bioavailability >30%, drug-likeness >0.18) highlighted four compounds: daucosterol, β-sitosterol (BS), stigmasterol, and sitosterol palmitate. Our own lifespan experiments demonstrate that only β-sitosterol at 0.01–0.02 µM significantly prolongs *Caenorhabditis elegans* survival, whereas other three compounds show little or no benefit at the same doses. Taken together,we focus on BS as the lead compound for dissecting how A. oxyphyllae mitigates aging through modulation of ferroptosis.

High-throughput RNA sequencing of Alpinia oxyphylla fructus -treated *C. elegans* identified ets-5, the orthologue of the human ETS repressor FEV (Holtzman and Olesnicky, 2017), as one of the most strongly regulated transcripts. Using bioinformatic analysis, we identified two conserved, high-affinity ETS-binding motifs within the human SLC7A11 promoter; previous mechanistic work shows that activating ETS factors such as ETS1 and ETS2 can cooperate with ATF4 to transcriptionally upregulate SLC7A11 under oxidative stress (Koppula et al., 2019). By contrast, FEV belongs to the inhibitory branch of the ETS family and represses target genes through its DNA-binding domain and alanine-rich region (Lévy et al., 2003), yet its role in ageing-related redox imbalance has not been explored. Independently, β-sitosterol (BS) has been shown to lower ROS, elevate GPX4, and restore tissue antioxidant enzyme activity in a 1,2-dimethylhydrazine–induced colon carcinogenesis model (Baskar et al., 2012a). Combining these lines of evidence with our preliminary docking data, we propose that BS binds to FEV, attenuates its repression of SLC7A11, reinstates the GSH–GPX4 axis, and thereby curtails ferroptosis.

In this study, we employed a three-tiered experimental model comprising *C. elegans*, human umbilical vein endothelial cells (HUVECs), and aged mice to integrate the strengths of each system while addressing their respective limitations.*C. elegans* offers rapid, low-cost lifespan screens and shares many longevity genes with mammals (Huang et al., 2024; Ye et al., 2020), yet its invertebrate simplicity demands confirmation in higher systems. Human-derived HUVECs allow precise genetic manipulation and quantification of redox biomarkers (ROS, MDA, GSH/GSSG), but still lack whole-organism interactions. Aged mice fill this gap, providing intact immunity, pharmacokinetics, and multi-organ read-outs that closely mirror human physiology, letting us evaluate efficacy and safety *in vivo*.

**FIGURE 1:**
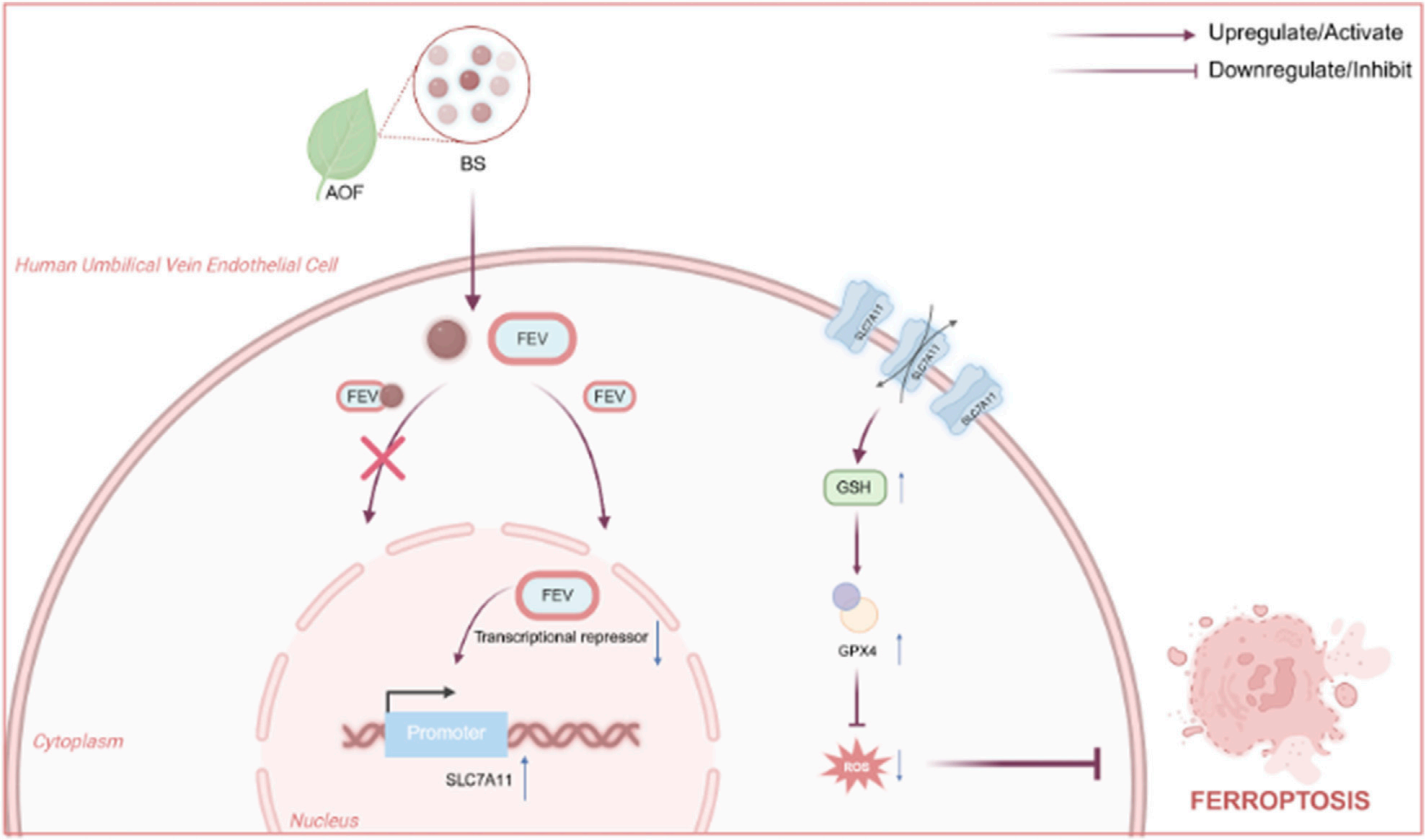
Mechanisms by which BS affects intracellular levels of oxidative stress by acting above the FEV and thereby alleviating senescence Fig.

To summarise, by elucidating how β-sitosterol re-activates the FEV-SLC7A11-GPX4 axis to suppress ferroptosis across nematode, cellular and mammalian models, this work seeks to establish a mechanistic foundation for developing safe, multitarget geroprotective agents derived from Alpinia oxyphylla. The proposed mechanism by which BS regulates intracellular oxidative stress via the FEV–SLC7A11 axis is illustrated in Figure 1.

## 2 Materials and methods

### 2.1 *Caenorhabditis elegans* model construction

Wild-type *C. elegans* (strain N2, Bristol; obtained from the *Caenorhabditis* Genetics Center in the United States) were used for all experiments. The worms were maintained at 20°C in the dark on Nematode Growth Medium (NGM) agar plates seeded with *Escherichia coli* OP50 for routine maintenance or HT115 (DE3) for RNAi feeding.

In the context of RNA interference (RNAi) experiments, HT115 (DE3) bacteria carrying either an empty vector (L4440) or a recombinant plasmid encoding double-stranded RNA that targets the ets-5 gene (MiaoLingBio, China) were cultivated overnight in Luria–Bertani (LB) medium containing 100 μg/mL ampicillin and 12.5 μg/mL tetracycline at 37°C. The cultures were then seeded onto NGM plates supplemented with 1 mM IPTG and 25 μg/mL carbenicillin. The plates were then incubated at room temperature for 12–16 h to induce dsRNA expression.

To synchronise the worm population, adult hermaphrodites were allowed to lay eggs on fresh NGM plates for two to 3 hours. After the adults were removed, the plates were incubated at 20°C for approximately 36 h to allow the eggs to hatch and develop into synchronised L4-stage larvae. These larvae were then transferred to plates for RNAi treatment or the control.

For the drug treatment, the worms were exposed to an Alpiniae oxyphylla fructus (AOF) extract (BS, Solarbio, China) at different concentrations, dissolved in dimethyl sulfoxide (DMSO) (final DMSO concentration ≤2%). The extract was administered by incorporating it into molten NGM agar prior to pouring the plates and by applying it to the surface of RNAi plates after seeding. Control groups received an equivalent volume of DMSO without BS.

The worms were kept on these plates at 20°C for 48 h. Their viability, development and behaviour were monitored daily using a stereomicroscope. Each experiment comprised a minimum of three biological replicates.

### 2.2 Cell culture

Human umbilical vein endothelial cells (HUVEC Catalog No. PCS-100-013), these cells are derived from a single, normal, full-term neonatal donor. And 293T cells (Catalog No. CRL-3216™), purchased from American Type Culture Collection (ATCC), were maintained at 37°C with 5% CO_2_ in DMEM medium (Gibco, United States) supplemented with 10% fetal bovine serum (FBS, Gibco, United States) and 1% penicillin-streptomycin. BS (Solarbio SS8580) dissolved in DMSO was used for 24-h pretreatment before inducing cellular senescence with 200 μM H_2_O_2_. All cell lines tested negative for *mycoplasma* contamination.

### 2.3 RNA interference

HUVECs were transfected with FEV-specific siRNA (sequence: 5′-agg​gcg​gtc​acg​gcg​agt​tca-3′) or negative control siRNA (Tsingke, China) using GeneTran™ III High Efficiency Transfection Reagent (BEIWO medical technology, China) for 6 h. The medium was subsequently replaced, and cells were cultured for an additional 48 h.

### 2.4 RNA extraction and qRT-PCR

Total RNA from HUVECs was extracted using TRIzol reagent (TaKaRa, 9109) and reverse-transcribed into cDNA using Evo M-MLV reverse transcription kit (ACCRATEBIOLOGY, AG11706). Quantitative real-time PCR was performed using SYBR Green premix (ACCRATEBIOLOGY, AG11739) on a Bio-Rad CFX96 Real-Time PCR Detection System (reaction conditions: pre-denaturation at 95°C for 5 min; 40 cycles at 95°C for 10 s and 60°C for 30 s). Primer sequences are listed in Supplementary Table S1. GAPDH served as the internal control, and relative gene expression was calculated using the 2^−ΔΔCT^ method (n = 3 technical replicates).

### 2.5 Western blot analysis

Cells were washed with precooled PBS, lysed on ice for 30 min with RIPA buffer containing protease inhibitors (Beyotime, P0013B), and centrifuged at 16,000 × *g* for 15 min. Protein concentrations were determined by BCA assay (Beyotime, P0009). Proteins (30 μg) underwent electrophoresis on 12% SDS-PAGE gels, transferred onto PVDF membranes (Millipore, United States), and blocked with QuickBlock™ Blocking Buffer (Beyotime, P0252) for 1 h. Membranes were sequentially incubated with primary antibodies against GPX4 (proteintech 26864-1-AP, 1:1,000), SLC7A11 (proteintech 30388-1-AP, 1:1,000), and GAPDH (proteintech 10494-1-AP, 1:2000), followed by incubation with HRP-conjugated secondary antibody (proteintech RGAR001, 1:5,000) at room temperature for 1 h. Blots were visualized using an ECL chemiluminescence imaging system (Tanon 5200), and gray-scale intensities quantified with ImageJ software.

### 2.6 Cellular functional assays

#### 2.6.1 Cell viability (CCK-8 assay)

HUVECs (5 × 10^3^ cells/well) seeded into 96-well plates were treated with BS for 24 h, then incubated with 10% CCK-8 reagent (Beyotime, C0039) at 37°C for 2 h in the dark. Absorbance at 450 nm was measured using a Multiskan GO microplate reader (Thermo Scientific).

#### 2.6.2 Cellular senescence (SA-β-gal staining)

HUVECs cultured to 70% confluence in six-well plates were treated with 200 μM H_2_O_2_ ± 0.05 μM BS for 24 h, fixed, and stained using a senescence β-galactosidase staining kit (Solarbio, G1580) for 16 h at 37°C (without CO_2_). Five random fields per well were photographed using a Nikon Eclipse Ci-L microscope, and percentages of positive cells calculated.

#### 2.6.3 ROS and glutathione measurement

Intracellular ROS was detected using DHE probe (Beyotime S0063, 5 μM) with a 30-min incubation in the dark. Glutathione levels were measured using a GSH/GSSG assay kit (Beyotime S0053), with results expressed as μmol/mg protein (n = 3 independent experiments).

### 2.7 Dual-luciferase reporter assay

Luciferase reporter vectors containing either wild-type (WT) or mutated (Mut) SLC7A11-binding sequences were constructed. 293T cells were co-transfected with these vectors and FEV-overexpression or negative control (NC) plasmids using GeneTran™ III High Efficiency Transfection Reagent. After 48-h transfection, luciferase activity was measured using a Dual-Luciferase Reporter Assay Kit (Yeasen Biotechnology, 11402ES80) to evaluate interactions between FEV and SLC7A11.

### 2.8 Animal experiments

Twelve 14-month-old male C57BL/6 mice were obtained from Aniphe Biolab (Guangzhou, China) and maintained in a specific pathogen-free (SPF) facility under controlled environmental conditions (temperature: 22°C ± 2°C; relative humidity: 50%–60%; light/dark cycle: 12 h/12 h). Animals had free access to standard rodent chow and water. After a 4-week acclimation period, the mice were randomly divided into two groups (n = 6 per group) using a computer-generated randomization list: a control group receiving 0.1 mL/10 g body weight of saline, and a BS treatment group receiving 4 mg/kg/day of BS dissolved in physiological saline. All treatments were administered once daily by oral gavage for four consecutive weeks. To minimize experimental bias, all histological and molecular analyses were performed by investigators blinded to group assignments. After sacrifice, liver tissues were prepared for paraffin-embedded SA-β-gal staining (fixed in 4% paraformaldehyde, Beyotime P0099), frozen section ROS detection (DHE probe), and protein extraction with RIPA buffer for Western blot analysis.

### 2.9 Statistical analysis

Data are expressed as mean ± SEM. Statistical analyses were conducted using GraphPad Prism 9.0 with two-tailed Student’s t-test or one-way ANOVA followed by Tukey’s *post hoc* test. A p-value of <0.05 was considered statistically significant. Animal experimental protocols were approved by the Institutional Animal Care and Use Committee (AM01012).

## 3 Results

### 3.1 BS is the main extract from AOF that affects cellular and *Caenorhabditis elegans* senescence

**FIGURE 2:**
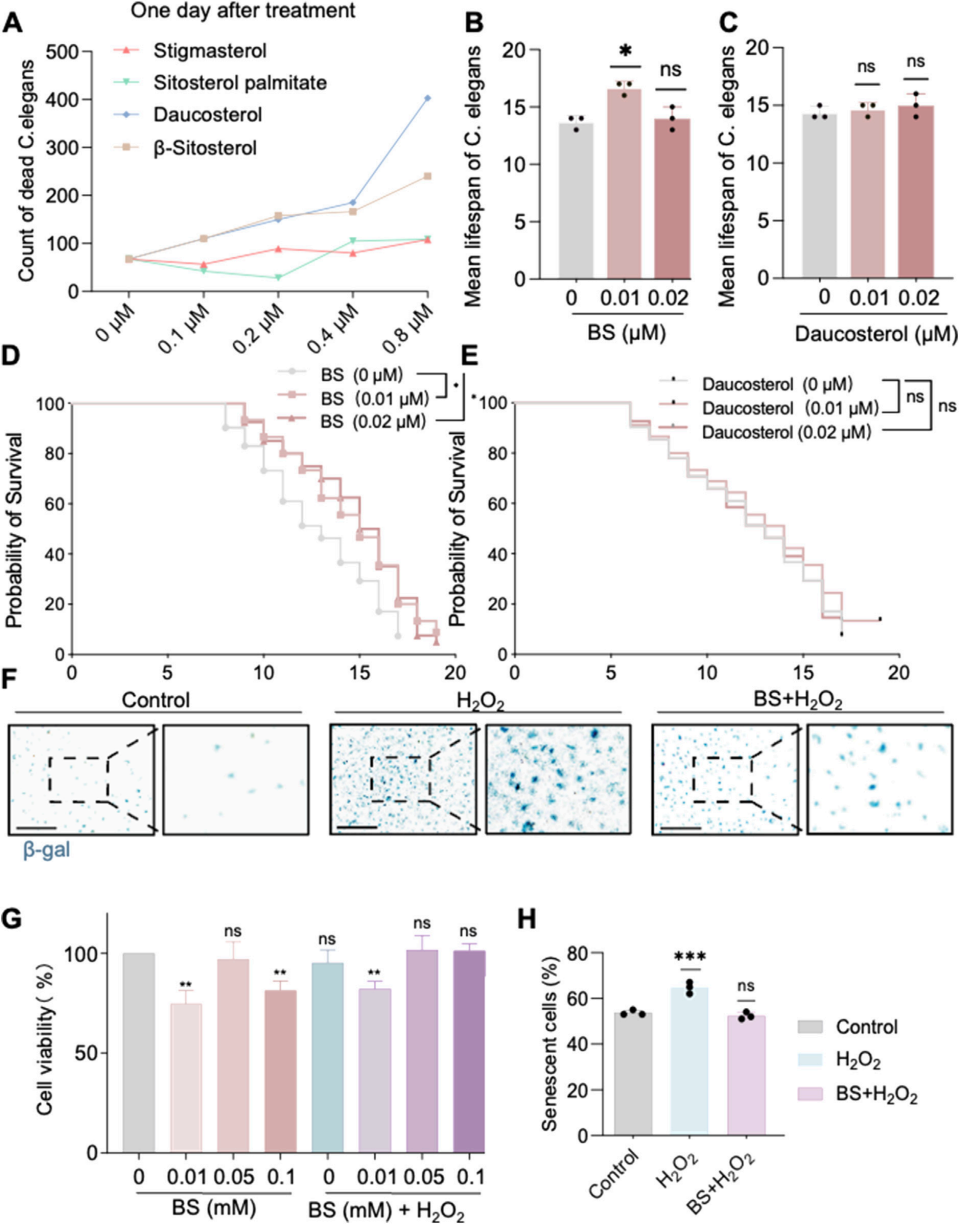
β-sitosterol alleviates aging in *Caenorhabditis elegans* and cells. **(A)** Effect of different concentrations of compounds on the lifespan of *Caenorhabditis elegans*. **(B)** Effect of different concentrations of β-sitosterol on the mean lifespan of *Caenorhabditis elegans*. **(C)** Effect of different concentrations of Daucosterol on the mean lifespan of *Caenorhabditis elegans*. **(D,E)** Probability of sirvival of *Caenorhabditis elegans*. Under treatment with different concentrations of β-sitosterol and daucosterol 0 μM β-sitosterol VS 0.01 μM,P = 0.0216; 0 μM β-sitosterol VS 0.02 μM,P = 0.0128; 0 μM Daucosterol VS 0.01 μM,P = 0.4042; 0 μM Daucosterol VS 0.02 μM,P = 0.9605. The statistical method used was the Log-rank test **(F)**. **(H)** β-galactosidase staining confirms the anti-aging effect of β-sitosterol on cells. **(G)** Effect of different concentrations of β-sitosterol on cell viability. Data are presented as mean ± SD. *P < 0.05; **P < 0.01; ***P < 0.001. All experiments were independently repeated at least three times.

Our previous findings indicated that AOF effectively ameliorates aging in *C. elegans*. To further elucidate the active constituents responsible for its anti-aging effects, the Traditional Chinese Medicine Systems Pharmacology (TCMSP) database was utilized, and four principal compounds—daucosterol, β-sitosterol (BS), stigmasterol, and sitosterol palmitate—were identified based on oral bioavailability (OB >30%) and drug-likeness (DL > 0.18). Subsequent evaluations involved treating *C. elegans* with these compounds at concentrations of 0.8 µM, 0.4 µM, 0.2 µM, 0.1 uM, and 0 µM (Figure 2A), respectively. Results indicated that lower concentrations of daucosterol and BS significantly prolonged the lifespan of *C. elegans*. Further comparison at concentrations of 0.2 µM and 0.1 µM demonstrated that BS at low concentration exhibited a more substantial effect in lifespan extension than daucosterol (Figures 2B–E). Consequently, BS was identified as the primary active ingredient in AOF responsible for anti-aging effects. Treatment with 200 µM H_2_O_2_ successfully induced a robust senescence model in HUVECs (Lin and Ruan, 2021). Notably, pretreatment with different concentrations of BS (0.01 mM, 0.05 mM and 0.1 mM) significantly restored cell viability compared to the H_2_O_2_-only treatment group (Figure 2G), and the concentration of 0.05 uM of BS had the least effect on the viability of the cells, and β-gal staining further confirmed that 0.05 µM of BS significantly attenuated hydrogen peroxide-induced HUVEC senescence (Figures 2F,H). Thus, BS is confirmed as the primary active compound in AOF affecting aging processes in both *C. elegans* and cellular models.

### 3.2 BS mitigates cellular senescence and ferroptosis

**FIGURE 3:**
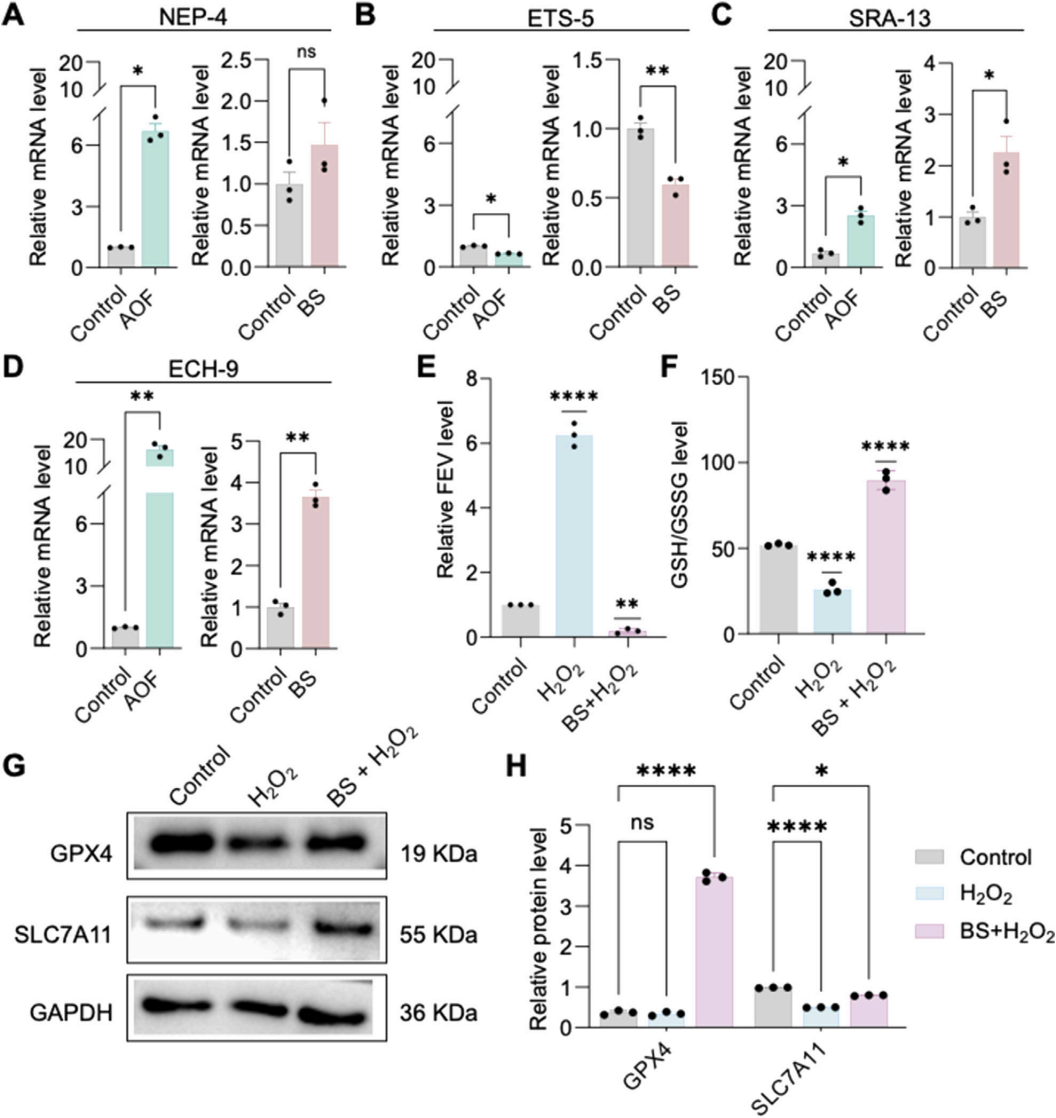
β-sitosterol attenuates cellular senescence. **(A–D)** qPCR analysis indicates that the downregulation of ETS-5 is the most consistent after *Caenorhabditis elegans* treatment with AOF extract or β-sitosterol. **(E)** FEV expression is associated with cellular senescence, where FEV is highly expressed in senescent cells, but its expression can be reduced by β-sitosterol. **(F)** β-sitosterol treatment significantly increases glutathione levels in the cellular senescence model. **(G,H)** β-sitosterol treatment increases the expression of GPX4 and SLC7A11. Data are presented as mean ± SD. *P < 0.05; **P < 0.01; ***P < 0.001; ****P < 0.0001; ns, not significant. All experiments were independently repeated at least three times.

To further investigate the mechanisms underlying the effects of BS on cellular senescence, high-throughput sequencing was performed on *C. elegans* treated with AOF, identifying four significantly differentially expressed genes: SRA-13, NEP-4, ETS-5, and ECH-9. Quantitative RT-PCR validation of these genes following individual treatments with AOF and BS revealed that ETS-5 exhibited the most consistent changes (Figures 3A–D). Thus, it is proposed that BS mediates aging primarily through modulation of ETS-5 expression. The human homolog of ETS-5, FEV, was significantly upregulated in H_2_O_2_-induced HUVEC senescence models compared to untreated controls, while pre-treatment with BS reduced its expression levels (Figure 3E). These results further support the hypothesis that BS modulates cellular senescence via regulating FEV. Previous studies suggest that oxidative stress induced by hydrogen peroxide may trigger cellular senescence accompanied by ferroptosis. Western blot demonstrated significantly elevated expression of the ferroptosis markers GPX4 and SLC7A11 following BS treatment (Figures 3G–H). Additionally, treatment with BS significantly increased intracellular reduced glutathione levels, clearly suggesting amelioration of oxidative stress (Figure 3F).

### 3.3 Effects of FEV knockdown on cellular senescence and ferroptosis

**FIGURE 4:**
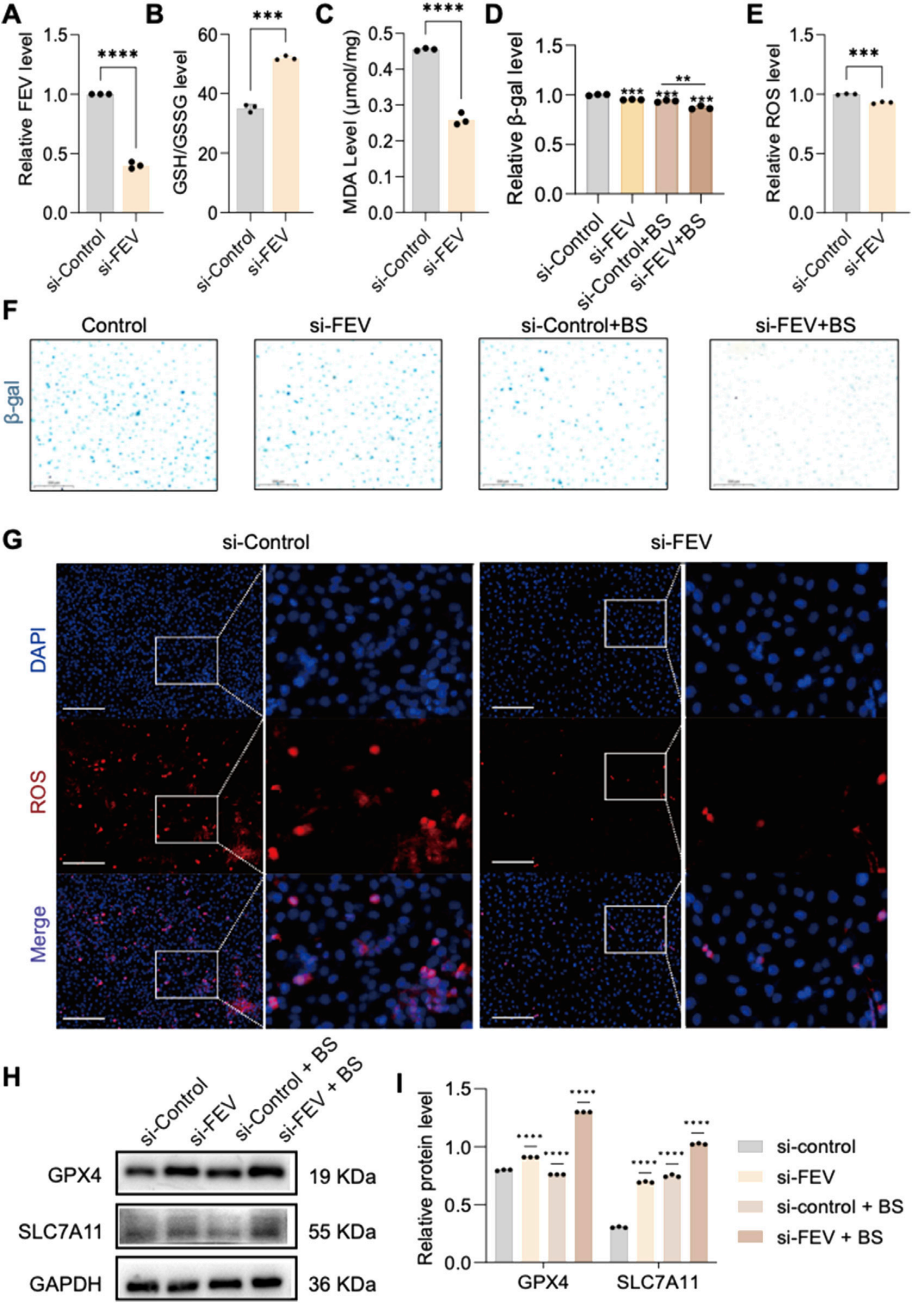
FEV plays a significant role in cellular senescence. **(A)** Verification of si-FEV knockdown efficiency. **(B,C)** Knockdown of FEV increases glutathione expression and reduces malondialdehyde production, alleviating cell death. **(D,F)** β-galactosidase staining confirms significant improvement in cell senescence after FEV knockdown. **(E,G)** Knockdown of si-FEV reduces intracellular ROS production. Furthermore, knockdown of si-FEV has been shown to significantly increase the expression of GPX4 and SLC7A11 **(H,I)**. The data presented here are expressed as mean ± SD. ***P < 0.001; ****P < 0.0001; ns, not significant. All experiments were independently repeated at least three times.

To further clarify whether FEV affects HUVEC senescence through ferroptosis, the researchers used RNA interference (RNAi) to knock down the expression of FEV (Figure 4A), and then assessed the senescence by β-gal staining. The results showed that silencing FEV could effectively alleviate cellular senescence (Figures 4D,F). Cellular senescence is often accompanied by the production of superoxide anion. Dihydroethidium (DHE) is a fluorescent probe that can be oxidised to ethidium by superoxide anion in cells. Using the DHE probe, the level of intracellular superoxide anions can be effectively monitored, and glutenol treatment resulted in a significant decrease in intracellular superoxide levels and an alleviation of intracellular oxidative stress. This further suggests that low expression of FEV can alleviate intracellular oxidative stress levels (Figures 4E,G). In addition, the ratio of reduced glutathione to oxidised glutathione (GSH/GSSG) was significantly increased, and the expression of malondialdehyde (MDA), a biomarker of iron metabolism indicative of lipid peroxidation, was also significantly reduced after FEV knockdown (Figures 4B,C). We also detected significant upregulation of the key markers of ferroptosis-related GPX4 and SLC7A11 by Western blot at both protein levels, Whereas ferroptosis was somewhat alleviated by the administration of BS treatment (Figures 4H,I).

### 3.4 FEV mediates ferroptosis and senescence through regulation of SLC7A11 expression

**FIGURE 5:**
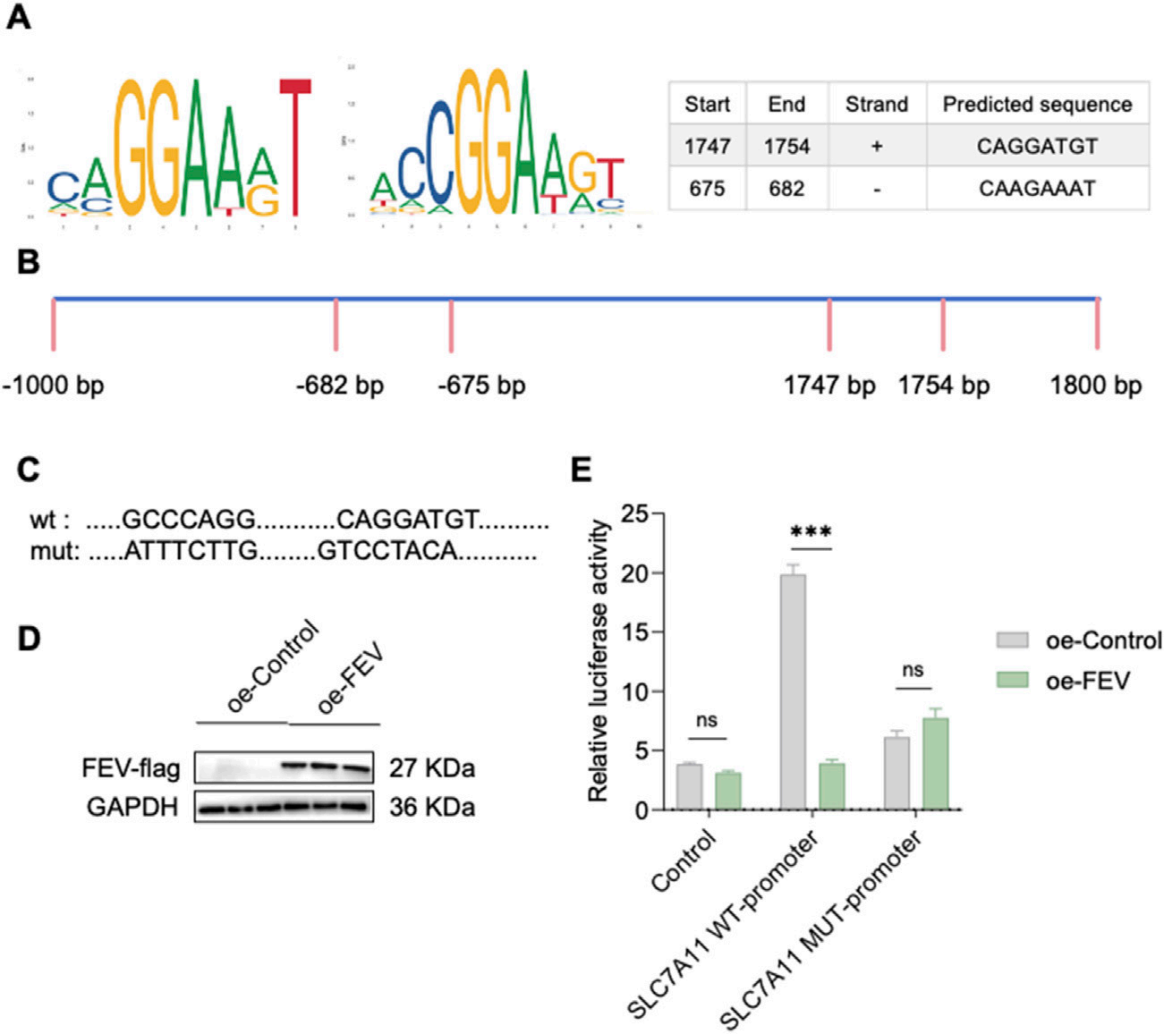
FEV has been found to inhibit the expression of SLC7A11 by competitively binding to its promoter. **(A,B)** Schematic of FEV binding to the SLC7A11 promoter region. **(C)** SLC7A11 wild-type and mutant plasmid insertion sequences. **(D,E)** Luciferase activity assay in 293T cells co-transfected with FEV overexpressing plasmid (with Flag tag), SLC7A11 control plasmid, SLC7A11 wild-type plasmid, or SLC7A11 mutant plasmid. ****P < 0.0001; ns, not significant. All experiments were independently repeated at least three times.

FEV is identified as a transcription factor, and Western blot revealed increased SLC7A11 expression following FEV knockdown. To explore the mechanism of FEV-mediated regulation of SLC7A11 expression, the binding motif for FEV was obtained from the Jaspar database, and potential binding sites on the SLC7A11 promoter were identified using the UCSC Genome Browser. Two FEV binding sites were identified within the SLC7A11 promoter region (Figures 5A–C). Therefore, we hypothesize that FEV inhibits transcription of SLC7A11 by direct promoter interaction. Dual-luciferase reporter assays demonstrated that mutations at these predicted FEV binding sites abolished the repressive effect of FEV overexpression on SLC7A11 promoter activity (Figures 5D,E).

### 3.5 BS alleviates aging in aged mice by reducing intracellular levels of oxidative stress

**FIGURE 6:**
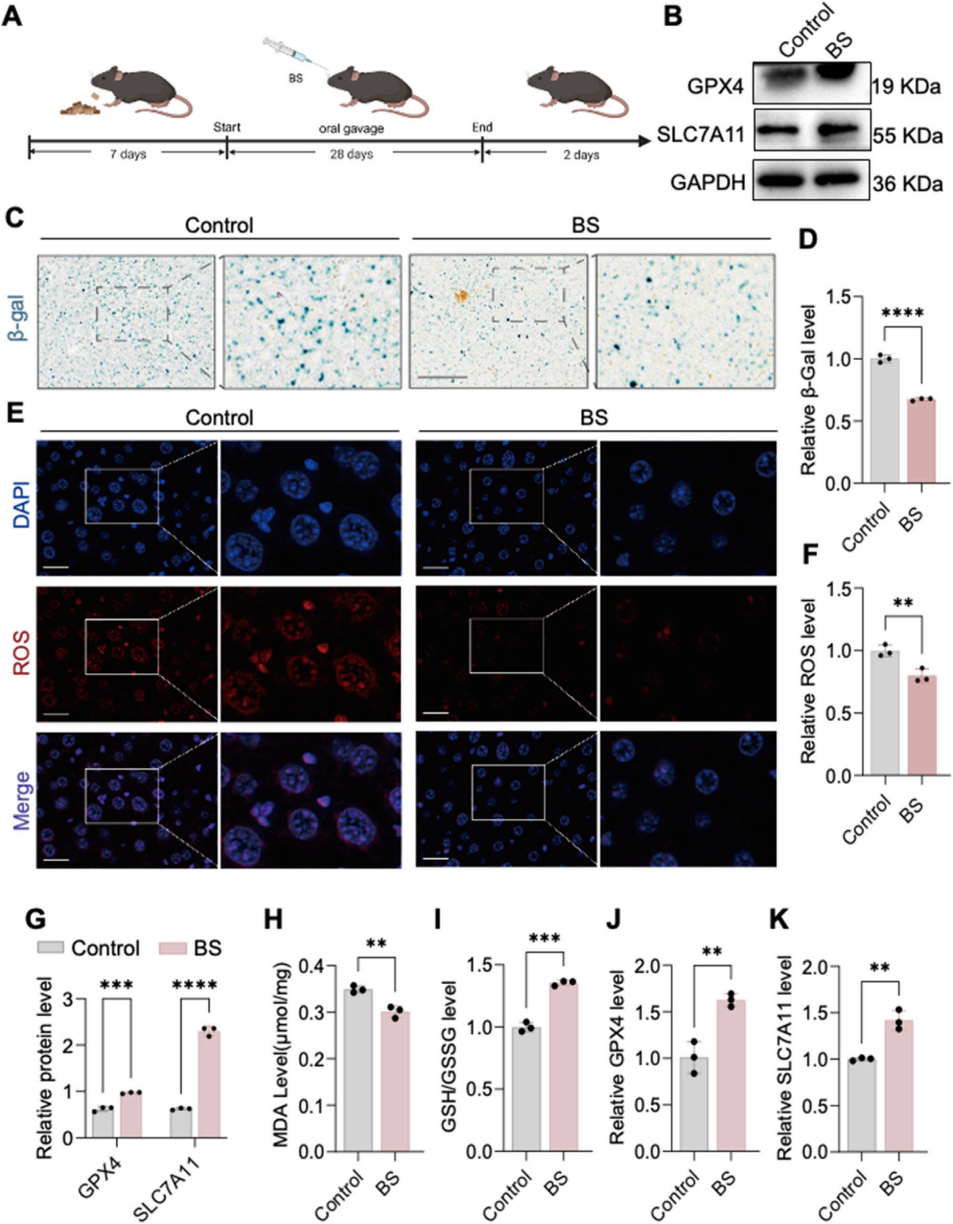
*In vivo* evidence shows that β-sitosterol alleviates aging. **(A)** Construction of an aging model in aged mice treated with β-sitosterol. **(B,C,F,G)** β-sitosterol enhances the expression of GPX4 and SLC7A11 in the liver of aged mice. **(D)** β-sitosterol reduces malondialdehyde (MDA) production in the liver of aged mice. **(E)** β-sitosterol increases the glutathione (GSH) content in the liver of aged mice. Furthermore, β-sitosterol has been observed to reduce ROS generation in the liver of aged mice **(H,I)**.I n addition, β-galactosidase staining of the liver of aged mice has been shown to demonstrate significant improvement in hepatic aging following β-sitosterol treatment **(J,K)**. The results of this study are supported by the statistical analysis, which shows that β-sitosterol treatment has a P < 0.01, P < 0.001, and P < 0.0001. It is important to note that all experiments were independently repeated at least three times.

Previous experiments established that HUVEC senescence induced by 200 µM H_2_O_2_ was effectively alleviated by BS, potentially through FEV-mediated suppression of SLC7A11 expression leading to ferroptosis. *In vivo* verification in aging C57 mice treated orally with BS for 28 consecutive days showed markedly decreased β-gal staining intensity in liver tissues compared to controls (Figures 6A,C,D). Further evaluation of oxidative stress-related ferroptosis markers demonstrated significant inhibition of superoxide anion levels (ROS staining) and reduced MDA levels, alongside increased GSH/GSSG ratios (Figures 6E,F,H,I). Quantitative RT-PCR and Western blot analyses further demonstrated a significant upregulation of SLC7A11 and GPX4 expression following BS administration in mice compared to controls (Figures 6B,G, J,K). Thus, it was further demonstrated by senescent mice that glutenol alleviates senescence in mice and that FEV is an important transcription factor affecting cellular senescence. In addition, immunohistochemistry showed that the expression of P53 in the nucleus was significantly downregulated after treatment with BS, which further said that the degree of oxidative stress in the account cells was alleviated to some extent (Supplementary Figure S1A).

## 4 Discussion

In this study, we identified the active compounds in AOF using TCSMP analysis based on oral bioavailability (OB >30%) and drug-likeness (DL >0.18). We further identified BS as the main compound affecting senescence in *C. elegans*. Our previous studies in the senescence model of *C. elegans* showed that BS treatment not only slowed down senescence, but also inhibited the expression of the GPX4 analogue of the ferroptosis protein. In a 200 μM H_2_O_2_-induced senescence model of human umbilical vein endothelial cells, early administration of BS treatment effectively slowed down cellular senescence. In addition, in aged mice, gavage of BS for 28 consecutive days further alleviated the senescence phenotype. These effects were mediated through the regulation of SLC7a11 expression by the FEV transcription factor, as schematically illustrated in Figure 5. Although a large number of studies have demonstrated that AOF can effectively alleviate senescence in individuals (Xiao M. et al., 2022; Wang et al., 2015), few studies have been reported on its sterols, and research on the role of sterols in senescence has been more limited.

Most people choose to use the HUVEC cell model induced by 200 μM H_2_O_2_ in experiments to study senescence. This is because vascular senescence accompanies individual aging, and senescence of vascular endothelial cells is a fundamental change in vascular aging and an important causative factor in vascular aging-related diseases such as atherosclerosis, stroke and coronary heart disease (Roth et al., 2015; Camici and Liberale, 2017; Paneni et al., 2017; North and Sinclair, 2012). However, senescence is a complex multicellular process and unicellular organisms do not usually age, so cellular senescence cannot be fully explained using cellular models alone (Campisi et al., 2019; Dimri et al., 1995). D-gal accelerates the aging process by promoting the formation of advanced glycosylation end products (AGEs) through a non-enzymatic glycosylation reaction and is a commonly used method for constructing animal models of senescence (Zhang J. J. et al., 2022). However, drug-induced animal models have drawbacks such as not easy to control and excessive apoptosis (Li et al., 2023; Li et al., 2010). *Caenorhabditis elegans*, which have a short life cycle and are easy to control, show obvious behavioural retardation and physiological decline during the aging process, making them an ideal model organism for aging (Xiao Y. et al., 2022). Based on this, this study used *C. elegans* as a target for pre-drug therapy and screening, as well as a tool for genetic screening; 200 μM H_2_O_2_-induced HUVEC cell model was selected for the study of related mechanisms; and finally, the results were further verified by a natural senescence model. This kind of aging study using multiple model organisms can not only improve the efficiency of drug selection and validate the universality of drugs, but also discover evolutionary conserved mechanisms and validate the universality of related mechanisms.

BS is a phytosterol that has previously been shown to possess a variety of biological activities including anti-inflammatory, antioxidant and free radical scavenging properties (Babu and Jayaraman, 2020; Yoshida and Niki, 2003; Vivancos and Moreno, 2005; Baskar et al., 2012b; Hah et al., 2022). Our experimental results further confirmed that BS can further reduce intracellular oxidative stress capacity. In the present study, we verified the senescence level of cells and mice by β-gal staining, and the results showed that BS treatment inhibited the senescence phenomenon of cells and mice in both *in vivo* and *in vitro* experiments. Not only that, BS also reduced ROS levels in HUVEC cells and liver tissues to a certain extent, inhibited MDA levels, and increased the GSSH/GSG ratio in cells and mice. In order to investigate how sterol affects the ability of oxidative stress in cells, we obtained the key gene for the action of BS, FEV, by means of high-throughput RNA-seq (Supplementary Figure S1C). FEV is a transcriptional repressor belonging to the ETS family of transcription factors (Maurer et al., 2003), members of which play important roles in cell proliferation, differentiation, apoptosis and angiogenesis (Zhang J. et al., 2022; Zhang et al., 2024). In addition, studies by Shiota and colleagues have shown that ETS transcription factor family members ETS1 and ETS2 regulate oxidative stress, which further supports our hypothesis (Shiota et al., 2008). Consistent with this, we observed a significant upregulation of SLC7a11 expression after RNA interference-mediated knockdown of FEV in cells, along with a decrease in oxidative stress indicators, such as changes in malondialdehyde (MDA) levels and glutathione redox ratio (GSSH/GSH). To further investigate the effect of BS treatment on FEV, we performed molecular docking, which showed that potential binding sites exist for BS and FEV (Supplementary Figure S1B). Therefore, we concluded that after BS treatment of the cells, BS entered the cells and bound to FEV, which in turn affected the conformational changes of FEV, and the inhibitory effect on SLC7a11 was alleviated, the level of oxidative stress in the cells was significantly reduced, and the senescence of the cells was alleviated to a certain extent.

Ferroptosis is a form of iron-dependent cell death driven by lipid peroxidation, and senescence is further exacerbated by the constant accumulation of cellular free radicals and increased levels of ROS (Yang et al., 2025). Ferroptosis has been shown to play an important role in senescent vascular smooth muscle cells and in animal models of senescence (Sun et al., 2024). SLC7a11 (solute carrier family 7 member 11) is an important transporter protein responsible for the transport of cystine into cells. Cystine is a key precursor for the biosynthesis of glutathione (GSH), which is a key cellular antioxidant (Yang et al., 2021; Koppula et al., 2021). In our experiments, FEV acts as a key transcriptional repressor that directly binds to the SLC7A11 promoter region (as confirmed by mutagenesis and dual-luciferase reporter assays), thereby constitutively suppressing SLC7A11 expression. This leads to reduced cystine uptake, impaired glutathione (GSH) synthesis, and diminished cellular antioxidant capacity. GSH deficiency further decreases the activity of glutathione peroxidase 4 (GPX4), resulting in the accumulation of lipid peroxides (LPO) and triggering ferroptosis. The accumulation of reactive oxygen species (ROS) and iron overload within the senescent microenvironment amplify this process, thereby forming a self-reinforcing positive feedback loop: oxidative stress → ferroptosis → accelerated senescence. Following BS treatment, BS enters cells and directly targets FEV, significantly downregulating FEV expression and releasing its transcriptional repression of SLC7A11 (potentially via inducing conformational changes or degradation of FEV). Upregulation of SLC7A11 protein promotes cystine influx and GSH synthesis, enhancing GPX4-mediated LPO clearance capacity and consequently inhibiting ferroptosis. This mechanism was validated by FEV RNAi experiments: Knockdown of FEV replicated the effects–decreased intracellular ROS levels, alleviated oxidative stress, and significantly improved cellular senescence phenotypes. Therefore, the FEV–SLC7A11 axis constitutes a core pathway linking senescence and ferroptosis, and BS exerts its anti-senescence effects by precisely targeting this axis.

Upon BS treatment, intracellular levels of FEV were significantly downregulated, SLC7a11 content was significantly upregulated, intracellular ROS levels were significantly decreased, and ferroptosis was inhibited. RNA interference with the expression of FEV further demonstrated that intracellular oxidative stress was alleviated. To further elucidate the mechanism of FEV-mediated intracellular oxidative stress, we performed bioinformatic analyses to identify potential binding sites for FEV within the SLC7a11 promoter region. The highest scoring binding sites were subsequently targeted for mutagenesis, and dual luciferase reporter assays confirmed the function of FEV as a transcriptional repressor that negatively regulates SLC7a11 expression. Therefore, we believe that after BS treatment of cells, BS enters the cells and binds to FEV, which in turn affects the conformational change of FEV, and the inhibitory effect on SLC7a11 is alleviated, and the level of oxidative stress in the cells is significantly reduced, and the senescence of the cells is alleviated to a certain extent.

In this study, BS was not isolated from the AOF extract but was obtained directly from commercial sources. This single-compound approach is inherently different from using full-spectrum extracts and may not fully capture the complete therapeutic potential of herbal medicine, particularly in terms of synergistic effects. Therefore, further experimental evaluation and validation will be conducted in future studies. While *C. elegans*, murine, and human cell models each provide unique mechanistic insights, we acknowledge that differences in physiology and gene regulation may limit direct translation of the findings. Future research will incorporate additional human-relevant models to systematically assess the combined effects across multiple organs and tissues, thereby strengthening the translational potential of our results. In subsequent work, we plan to systematically characterize the absorption and tissue distribution of BS in mice to optimize dosing strategies and further support its therapeutic potential.

In conclusion, our findings, for the first time, demonstrate that BS delays aging by modulating ferroptosis through FEV-mediated regulation of SLC7a11. By utilizing multi-level approaches in *C. elegans*, cellular, and mice models, this research provides a comprehensive mechanistic understanding of the anti-aging properties of BS. Nevertheless, the present study possesses certain limitations. Primarily, although we have confirmed the regulatory relationship between FEV and SLC7a11, further exploration of additional transcription factors or signaling pathways remains necessary. Future investigations should aim to clarify potential interactions of FEV with other transcriptional regulators or pathways to comprehensively elucidate the mechanisms underlying BS-mediated anti-aging effects.

## Data Availability

The processed RNA-seq data supporting the conclusions of this article are available in the article/Supplementary Material. Further inquiries can be directed to the corresponding authors.
