# Correction: The pivotal regulatory role of the FEV-SLC7A11 axis in ferroptosis elucidates the anti-aging mechanism of β-sitosterol in a cross-species study

**DOI:** 10.3389/fphar.2025.1685567

**Published:** 2025-08-25

**Authors:** Zhihan Fang, Liyao Xie, Jing Wang, Junyi Li, Yirui Pan, Lei Chen, Shuyi Cao, Qi Zhou, Shaobin Li, Chao Zhang, Li Li

**Affiliations:** ^1^ Department of Pathogenic Biology and Key Laboratory of Tropical Translational Medicine of Ministry of Education, School of Basic Medicine and Life Sciences, Hainan Medical University, Haikou, China; ^2^ Department of Biochemistry and Molecular Biology, School of Basic Medical Sciences, Centre for Experimental Management, Southern Medical University, Guangzhou, China; ^3^ The First School of Clinical Medicine, Southern Medical University, Guangzhou, China; ^4^ Department of Cardiology, The Fifth Affiliated Hospital of Southern Medical University, Guangzhou, China; ^5^ Department of Thoracic Surgery, Nanfang Hospital, Southern Medical University, Guangzhou, China

**Keywords:** β-sitosterol, ferroptosis, oxidative stress, cellular senescence, FEV, cross-species comparison

**Affiliation 1** “Department of Pathogenic Biology and Key Laboratory of Tropical Translational Medicine of Ministry of Education, School of Basic Medicine and Life Sciences, Hainan Medical University, Haikou, China” was previously listed as **Affiliation 5**, and was omitted for author **Zhihan Fang**. This affiliation has now been added for author **Zhihan Fang**, and the remaining affiliations have been renumbered accordingly.

The original article has been updated.

