# Supplementary material for: The pivotal regulatory role of the FEV-SLC7A11 axis in ferroptosis elucidates the anti-aging mechanism of β-sitosterol in a cross-species study
Source: Front Pharmacol. 2025 Aug 7;16:1600489. doi: 10.3389/fphar.2025.1600489 (PMC12368974; doi:10.3389/fphar.2025.1600489)
Supplement: Supplementary file 1 [file Supplementaryfile1.docx]

**Figure S1**
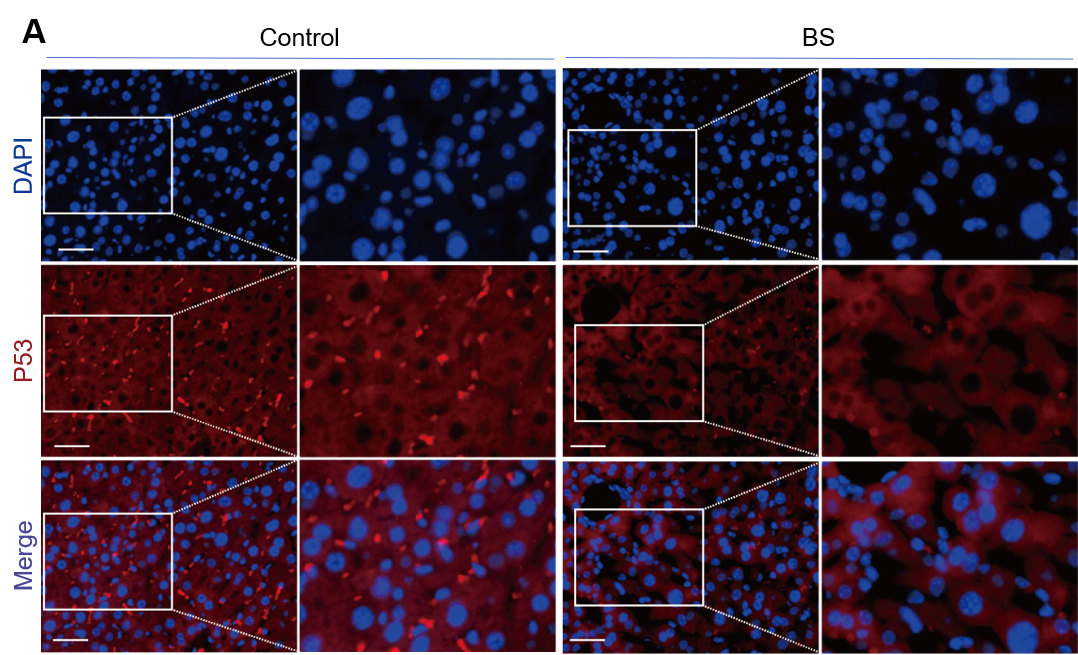

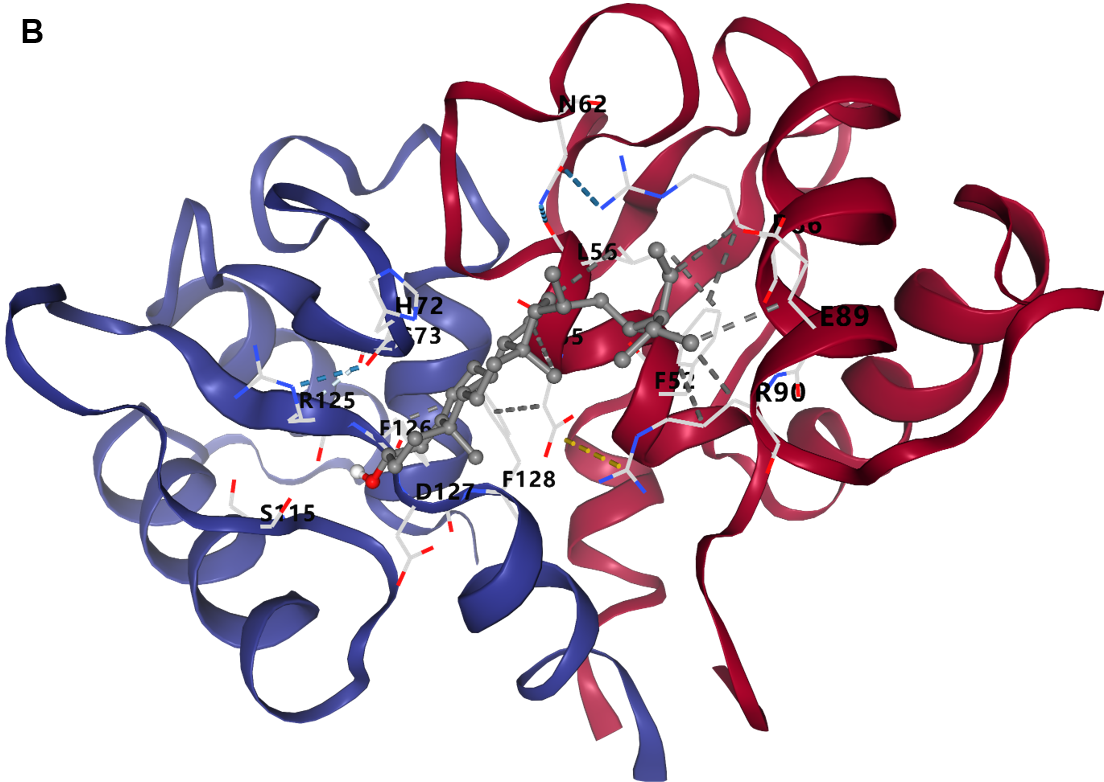


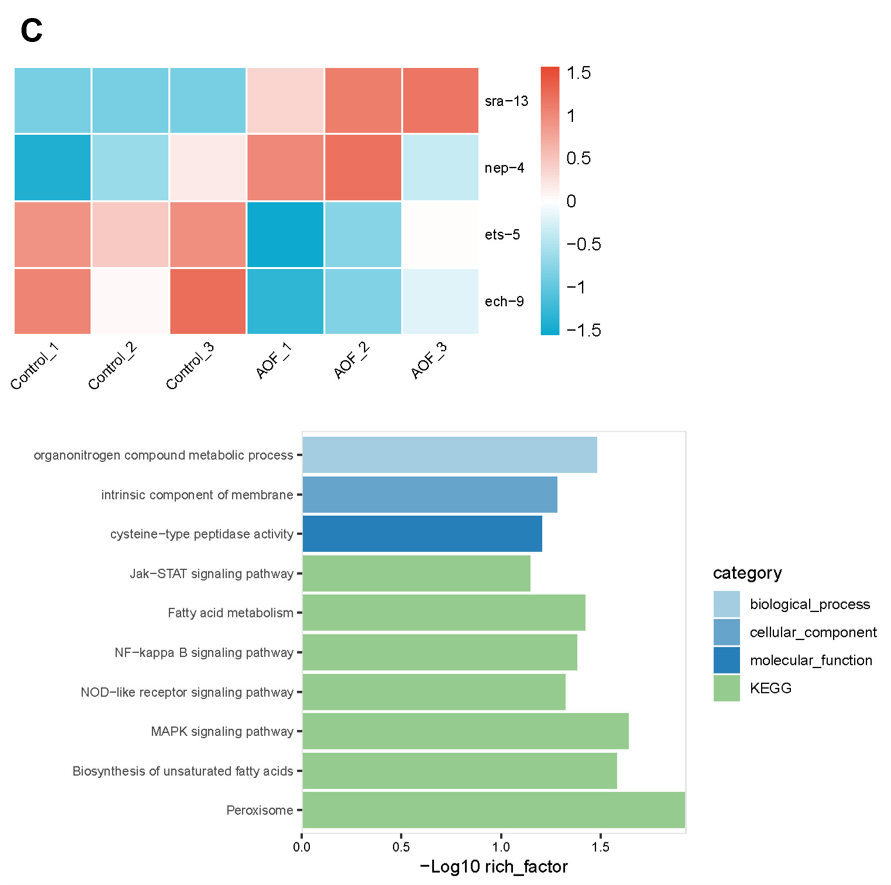


A The expression of the p53 protein in the liver tissue of aged mice

B Molecular docking diagram of the combination of BS and FEV

C ETS-5 transcriptomics and enrichment analysis

**Supplementary Table S1.** Oligonucleotide primers used for qRT-PCR analysis

| **Gene symbol** | **Primer direction** | **Sequence (5'→3')** | **Product size (bp)** |
| --- | --- | --- | --- |
| *GPX4* | Forward | GGAGCCAGGGAGTAACGAAG | 152 |
|  | Reverse | GACGGTGTCCAAACTTGGTG |  |
| *SLC7A11* | Forward | TGTGTGGGGTCCTGTCACTA | 168 |
|  | Reverse | CAGTAGCTGCAGGGCGTATT |  |
| *ets-5* | Forward | GGGGAGAGCGGAAAGAGTAA | 145 |
|  | Reverse | GATGACAGATCTCCGTTGGG |  |
| *sra-13* | Forward | CAGATATTTCCACTACTCAAATGGC | 132 |
|  | Reverse | CGTGTAGAGTAGGTCAGCCTTG |  |
| *nep-4* | Forward | ACCGTCATGCTTGTCCAGTT | 157 |
|  | Reverse | CCTCGTTTCGTTGGCCTTTC |  |
| *ech-9* | Forward | CTCTGCCAAATGATCGAG | 149 |
|  | Reverse | CTTGCTTGCATCTCTGCT |  |
| *GPX1* | Forward | GTCACTTTCGGATTACAAAGGAAA | 121 |
|  | Reverse | GGGAAGGCAAGAACTTCGAGA |  |
| *ACS-17* | Forward | GTCGGAGAGAGTCAAGGCTG | 163 |
|  | Reverse | ACGTCGGACACATTCTTCCC |  |
| *Ftn-1* | Forward | CGAGTGGGGAACTGTCCTTG | 138 |
|  | Reverse | TCATTGATCGAATGTACCTGCTCT |  |
| *Aat-9* | Forward | ACAAATGGCATGGCACTTGT | 175 |
|  | Reverse | CGACCCACATGAACGAGAAC |  |
| *daf-16* | Forward | CGCCGCTACCATCTGACATCAC | 142 |
|  | Reverse | TCCGCCAAAAGAAGCCGAACG |  |
| *GAPDH* | Forward | TGGATTTGGACGCATTGGTC | 112 |
|  | Reverse | TTTGCACTGGTACGTGTTGAT |  |
