# Supplementary material for: The pivotal regulatory role of the FEV-SLC7A11 axis in ferroptosis elucidates the anti-aging mechanism of β-sitosterol in a cross-species study
Source: Front Pharmacol. 2025 Aug 7;16:1600489. doi: 10.3389/fphar.2025.1600489 (PMC12368974; doi:10.3389/fphar.2025.1600489)
Supplement: Supplementary file 2 [file DataSheet1.zip › Analysis_report.html]

mRNA分析报告


- 实验
  - 1.1 抽提与质检
  - 1.2 文库构建
  - 1.3 上机测序
- 质控
  - 2.1 原始数据质控
  - 2.2 Reads质量评估
- 基因组比对
  - 3.1 基因组比对统计
  - 3.3 Reads分布统计
- 转录本组装
  - 4.1 转录本组装原理
  - 4.2 组装转录本分类
  - 4.3 编码蛋白质的新转录本预测
- 差异表达
  - 5.1 基因整体表达水平
  - 5.2 差异基因筛选
  - 5.3 表达趋势聚类
- 可变剪切
  - 6.1 可变性剪切统计
  - 6.2 差异可变性剪切分析
- 功能分析
  - 7.2 KEGG富集分析
  - 7.5 蛋白质互作分析
- 高级分析
- 方法与答疑
  - 9.1 方法和材料
  - 9.2 F&Q
  - 9.3 参考文献
  - 9.4 参考数据库
  - 9.5 相关软件
- 联系我们
  - 联系我们

客户：Zhang Chao                                                              合同号：SO.20220707001

  


背景简介：信使RNA(mRNA)是一类由DNA的一条链作为模板转录而来的，携带遗传信息，能指导蛋白质合成的一类单链核糖核酸。 通过对PolyA特征序列进行mRNA序列捕获和二代测序成为mRNA定量的常规手段， 通过mRNA转录组测序，能够全面获得物种特定组织或器官的转录本信息，从而进行转录本结构研究、变异研究、基因表达水平研究以及全新转录本发现等研究。

  

## 目录信息

  

展开 |  合拢

mRNA分析结果

1-QC

op50\_1

Read1\_fastqc\_data\_per\_base\_quality.pdf

Read1\_fastqc\_data\_per\_base\_quality.png

Read2\_fastqc\_data\_per\_base\_quality.pdf

Read2\_fastqc\_data\_per\_base\_quality.png

op50\_2

Read1\_fastqc\_data\_per\_base\_quality.pdf

Read1\_fastqc\_data\_per\_base\_quality.png

Read2\_fastqc\_data\_per\_base\_quality.pdf

Read2\_fastqc\_data\_per\_base\_quality.png

op50\_3

Read1\_fastqc\_data\_per\_base\_quality.pdf

Read1\_fastqc\_data\_per\_base\_quality.png

Read2\_fastqc\_data\_per\_base\_quality.pdf

Read2\_fastqc\_data\_per\_base\_quality.png

QC.xls

S20mg\_1

Read1\_fastqc\_data\_per\_base\_quality.pdf

Read1\_fastqc\_data\_per\_base\_quality.png

Read2\_fastqc\_data\_per\_base\_quality.pdf

Read2\_fastqc\_data\_per\_base\_quality.png

S20mg\_2

Read1\_fastqc\_data\_per\_base\_quality.pdf

Read1\_fastqc\_data\_per\_base\_quality.png

Read2\_fastqc\_data\_per\_base\_quality.pdf

Read2\_fastqc\_data\_per\_base\_quality.png

S20mg\_3

Read1\_fastqc\_data\_per\_base\_quality.pdf

Read1\_fastqc\_data\_per\_base\_quality.png

Read2\_fastqc\_data\_per\_base\_quality.pdf

Read2\_fastqc\_data\_per\_base\_quality.png

strand\_specific.xls

2-MAP

MAP.xls

op50\_1

RSEQC\_exon\_intron\_distribution.txt

RSEQC.junction.xls

RSEQC.pdf

RSEQC.png

op50\_2

RSEQC\_exon\_intron\_distribution.txt

RSEQC.junction.xls

RSEQC.pdf

RSEQC.png

op50\_3

RSEQC\_exon\_intron\_distribution.txt

RSEQC.junction.xls

RSEQC.pdf

RSEQC.png

S20mg\_1

RSEQC\_exon\_intron\_distribution.txt

RSEQC.junction.xls

RSEQC.pdf

RSEQC.png

S20mg\_2

RSEQC\_exon\_intron\_distribution.txt

RSEQC.junction.xls

RSEQC.pdf

RSEQC.png

S20mg\_3

RSEQC\_exon\_intron\_distribution.txt

RSEQC.junction.xls

RSEQC.pdf

RSEQC.png

3-ASSEMBLY

merge

assembled\_transcripts\_c.fa

assembled\_transcripts\_c.gtf

assembled\_transcripts\_combined.fa

assembled\_transcripts\_combined.gtf

assembled\_transcripts\_combined\_multiexon\_200bp.fa

assembled\_transcripts\_combined\_multiexon\_200bp.gtf

assembled\_transcripts\_e.fa

assembled\_transcripts\_e.gtf

assembled\_transcripts\_equal.fa

assembled\_transcripts\_equal.gtf

assembled\_transcripts\_i.fa

assembled\_transcripts\_i.gtf

assembled\_transcripts\_j.fa

assembled\_transcripts\_j.gtf

assembled\_transcripts\_o.fa

assembled\_transcripts\_o.gtf

assembled\_transcripts\_p.fa

assembled\_transcripts\_p.gtf

assembled\_transcripts\_r.fa

assembled\_transcripts\_r.gtf

assembled\_transcripts\_s.fa

assembled\_transcripts\_s.gtf

assembled\_transcripts\_u.fa

assembled\_transcripts\_u.fa.transdecoder.gff3

assembled\_transcripts\_u.fa.transdecoder.pep

assembled\_transcripts\_u.gtf

assembled\_transcripts\_x.fa

assembled\_transcripts\_x.gtf

class\_code\_stat.pdf

class\_code\_stat.png

class\_code\_stat.xls

gene\_model\_modification.xls

isoform\_exon\_structure\_info.xls

merged.gtf

novel\_protein2domain.xls

novel\_protein2GO.xls

novel\_protein2KEGG.xls

protein\_blastp2swissprot\_all.xls

protein\_blastp2swissprot\_bestone.xls

op50\_1

transcripts\_filter.gtf

op50\_2

transcripts\_filter.gtf

op50\_3

transcripts\_filter.gtf

S20mg\_1

transcripts\_filter.gtf

S20mg\_2

transcripts\_filter.gtf

S20mg\_3

transcripts\_filter.gtf

4-DGE

Cluster

Cluster.pdf

Cluster.png

Cluster\_with\_symbol.xls

Cluster.xls

merge

All\_samples\_boxplot.pdf

All\_samples\_boxplot.png

All\_samples\_PCA.pdf

All\_samples\_PCA.png

gene\_sample\_count\_with\_symbol.xls

gene\_sample\_count.xls

gene\_sample\_FPKM\_stat.xls

gene\_sample\_FPKM\_with\_symbol.xls

gene\_sample\_FPKM.xls

gene\_sample\_log2FPKM\_with\_symbol.xls

gene\_sample\_log2FPKM.xls

gene\_sample\_log2normalized\_count\_with\_symbol.xls

gene\_sample\_log2normalized\_count.xls

group1\_boxplot.pdf

group1\_boxplot.png

group1\_PCA.pdf

group1\_PCA.png

sample\_Corr.pdf

sample\_Corr.png

transcript\_sample\_count\_with\_symbol.xls

transcript\_sample\_count.xls

transcript\_sample\_log2normalized\_count\_with\_symbol.xls

transcript\_sample\_log2normalized\_count.xls

overlap

genelist\_overlap\_log2normalized\_count\_heatmap.pdf

genelist\_overlap\_log2normalized\_count\_heatmap.png

genelist\_overlap\_log2normalized\_count\_heatmap.xls

genelist\_overlap\_with\_symbol.xls

S20mg\_vs\_op50

gene\_exp\_significant\_log2normalized\_count\_heatmap.pdf

gene\_exp\_significant\_log2normalized\_count\_heatmap.png

gene\_exp\_significant\_with\_symbol\_GO\_KEGG\_FPKM.xls

gene\_exp\_significant\_with\_symbol\_GO\_KEGG.xls

gene\_exp\_with\_symbol.xls

Scatter\_Plot.pdf

Scatter\_Plot.png

Volcano\_and\_Scatter\_Plots.pdf

Volcano\_and\_Scatter\_Plots.png

Volcano\_Plot.pdf

Volcano\_Plot.png

5-AS

merge

A3SS.xls

A5SS.xls

AS\_event\_stat.pdf

AS\_event\_stat.png

AS\_event\_stat.xls

MXE.xls

RI.xls

SE.xls

S20mg\_vs\_op50

all\_A3SS\_with\_symbol.xls

all\_A5SS\_with\_symbol.xls

all\_MXE\_with\_symbol.xls

all\_RI\_with\_symbol.xls

all\_SE\_with\_symbol.xls

diff\_A3SS\_with\_symbol.xls

diff\_A5SS\_with\_symbol.xls

diff\_MXE\_with\_symbol.xls

diff\_RI\_with\_symbol.xls

diff\_SE\_with\_symbol.xls

6-FUN

S20mg\_vs\_op50

GO\_KEGG

allsymbol.txt

desymbol.txt

GO\_enrichment\_significant\_topGO\_and\_gene\_exp.xls

GO\_enrichment\_significant\_topGO.xls

GO\_enrichment\_topGO\_and\_gene\_exp.xls

GO\_enrichment\_topGO\_Pvalue\_Barplot.pdf

GO\_enrichment\_topGO\_Pvalue\_Barplot.png

GO\_enrichment\_topGO.xls

GO\_tree\_topGO\_BP\_MF\_CC.pdf

GO\_tree\_topGO\_BP\_MF\_CC.png

KEGG\_enrichment\_2D.pdf

KEGG\_enrichment\_2D.png **-  PATHWAY富集分析散点展示**

KEGG\_enrichment\_and\_gene\_exp.xls

KEGG\_enrichment\_GenePercentage\_Barplot.pdf

KEGG\_enrichment\_GenePercentage\_Barplot.png

KEGG\_enrichment\_KEGG2Symbol\_Net.pdf

KEGG\_enrichment\_KEGG2Symbol\_Net.png

KEGG\_enrichment\_Pathway2Pathway\_Net.pdf

KEGG\_enrichment\_Pathway2Pathway\_Net.png

KEGG\_enrichment\_Pvalue\_Barplot.pdf

KEGG\_enrichment\_Pvalue\_Barplot.png

KEGG\_enrichment\_significant\_and\_gene\_exp.xls

KEGG\_enrichment\_significant\_with\_web.xls

KEGG\_enrichment\_significant.xls

KEGG\_enrichment.xls

Rplots.pdf

strings\_network.pdf

strings\_network.png

strings\_network.xls

top50\_network.xls

GO\_KEGG\_Down

allsymbol.txt

desymbol.txt

GO\_enrichment\_significant\_topGO\_and\_gene\_exp.xls

GO\_enrichment\_significant\_topGO.xls

GO\_enrichment\_topGO\_and\_gene\_exp.xls

GO\_enrichment\_topGO\_Pvalue\_Barplot.pdf

GO\_enrichment\_topGO\_Pvalue\_Barplot.png

GO\_enrichment\_topGO.xls

GO\_tree\_topGO\_BP\_MF\_CC.pdf

GO\_tree\_topGO\_BP\_MF\_CC.png

KEGG\_enrichment\_2D.pdf

KEGG\_enrichment\_2D.png **-  PATHWAY富集分析散点展示**

KEGG\_enrichment\_and\_gene\_exp.xls

KEGG\_enrichment\_GenePercentage\_Barplot.pdf

KEGG\_enrichment\_GenePercentage\_Barplot.png

KEGG\_enrichment\_KEGG2Symbol\_Net.pdf

KEGG\_enrichment\_KEGG2Symbol\_Net.png

KEGG\_enrichment\_Pathway2Pathway\_Net.pdf

KEGG\_enrichment\_Pathway2Pathway\_Net.png

KEGG\_enrichment\_Pvalue\_Barplot.pdf

KEGG\_enrichment\_Pvalue\_Barplot.png

KEGG\_enrichment\_significant\_and\_gene\_exp.xls

KEGG\_enrichment\_significant\_with\_web.xls

KEGG\_enrichment\_significant.xls

KEGG\_enrichment.xls

Rplots.pdf

strings\_network.pdf

strings\_network.png

strings\_network.xls

top50\_network.xls

GO\_KEGG\_Up

allsymbol.txt

desymbol.txt

GO\_enrichment\_significant\_topGO\_and\_gene\_exp.xls

GO\_enrichment\_significant\_topGO.xls

GO\_enrichment\_topGO\_and\_gene\_exp.xls

GO\_enrichment\_topGO.xls

GO\_tree\_topGO\_BP\_MF\_CC.pdf

GO\_tree\_topGO\_BP\_MF\_CC.png

KEGG\_enrichment\_2D.pdf

KEGG\_enrichment\_2D.png **-  PATHWAY富集分析散点展示**

KEGG\_enrichment\_and\_gene\_exp.xls

KEGG\_enrichment\_GenePercentage\_Barplot.pdf

KEGG\_enrichment\_GenePercentage\_Barplot.png

KEGG\_enrichment\_KEGG2Symbol\_Net.pdf

KEGG\_enrichment\_KEGG2Symbol\_Net.png

KEGG\_enrichment\_Pathway2Pathway\_Net.pdf

KEGG\_enrichment\_Pathway2Pathway\_Net.png

KEGG\_enrichment\_Pvalue\_Barplot.pdf

KEGG\_enrichment\_Pvalue\_Barplot.png

KEGG\_enrichment\_significant\_and\_gene\_exp.xls

KEGG\_enrichment\_significant\_with\_web.xls

KEGG\_enrichment\_significant.xls

KEGG\_enrichment.xls

strings\_network.pdf

strings\_network.png

strings\_network.xls

top50\_network.xls

GN-method

mRNAseq\_method\_Chinese.pdf

mRNAseq\_method\_English.pdf

mRNA分析结果

1-QC

op50\_1

Read1\_fastqc\_data\_per\_base\_quality.pdf

Read1\_fastqc\_data\_per\_base\_quality.png

Read2\_fastqc\_data\_per\_base\_quality.pdf

Read2\_fastqc\_data\_per\_base\_quality.png

op50\_2

Read1\_fastqc\_data\_per\_base\_quality.pdf

Read1\_fastqc\_data\_per\_base\_quality.png

Read2\_fastqc\_data\_per\_base\_quality.pdf

Read2\_fastqc\_data\_per\_base\_quality.png

op50\_3

Read1\_fastqc\_data\_per\_base\_quality.pdf

Read1\_fastqc\_data\_per\_base\_quality.png

Read2\_fastqc\_data\_per\_base\_quality.pdf

Read2\_fastqc\_data\_per\_base\_quality.png

QC.xls

S20mg\_1

Read1\_fastqc\_data\_per\_base\_quality.pdf

Read1\_fastqc\_data\_per\_base\_quality.png

Read2\_fastqc\_data\_per\_base\_quality.pdf

Read2\_fastqc\_data\_per\_base\_quality.png

S20mg\_2

Read1\_fastqc\_data\_per\_base\_quality.pdf

Read1\_fastqc\_data\_per\_base\_quality.png

Read2\_fastqc\_data\_per\_base\_quality.pdf

Read2\_fastqc\_data\_per\_base\_quality.png

S20mg\_3

Read1\_fastqc\_data\_per\_base\_quality.pdf

Read1\_fastqc\_data\_per\_base\_quality.png

Read2\_fastqc\_data\_per\_base\_quality.pdf

Read2\_fastqc\_data\_per\_base\_quality.png

strand\_specific.xls

2-MAP

MAP.xls

op50\_1

RSEQC\_exon\_intron\_distribution.txt

RSEQC.junction.xls

RSEQC.pdf

RSEQC.png

op50\_2

RSEQC\_exon\_intron\_distribution.txt

RSEQC.junction.xls

RSEQC.pdf

RSEQC.png

op50\_3

RSEQC\_exon\_intron\_distribution.txt

RSEQC.junction.xls

RSEQC.pdf

RSEQC.png

S20mg\_1

RSEQC\_exon\_intron\_distribution.txt

RSEQC.junction.xls

RSEQC.pdf

RSEQC.png

S20mg\_2

RSEQC\_exon\_intron\_distribution.txt

RSEQC.junction.xls

RSEQC.pdf

RSEQC.png

S20mg\_3

RSEQC\_exon\_intron\_distribution.txt

RSEQC.junction.xls

RSEQC.pdf

RSEQC.png

3-ASSEMBLY

merge

assembled\_transcripts\_c.fa

assembled\_transcripts\_c.gtf

assembled\_transcripts\_combined.fa

assembled\_transcripts\_combined.gtf

assembled\_transcripts\_combined\_multiexon\_200bp.fa

assembled\_transcripts\_combined\_multiexon\_200bp.gtf

assembled\_transcripts\_e.fa

assembled\_transcripts\_e.gtf

assembled\_transcripts\_equal.fa

assembled\_transcripts\_equal.gtf

assembled\_transcripts\_i.fa

assembled\_transcripts\_i.gtf

assembled\_transcripts\_j.fa

assembled\_transcripts\_j.gtf

assembled\_transcripts\_o.fa

assembled\_transcripts\_o.gtf

assembled\_transcripts\_p.fa

assembled\_transcripts\_p.gtf

assembled\_transcripts\_r.fa

assembled\_transcripts\_r.gtf

assembled\_transcripts\_s.fa

assembled\_transcripts\_s.gtf

assembled\_transcripts\_u.fa

assembled\_transcripts\_u.fa.transdecoder.gff3

assembled\_transcripts\_u.fa.transdecoder.pep

assembled\_transcripts\_u.gtf

assembled\_transcripts\_x.fa

assembled\_transcripts\_x.gtf

class\_code\_stat.pdf

class\_code\_stat.png

class\_code\_stat.xls

gene\_model\_modification.xls

isoform\_exon\_structure\_info.xls

merged.gtf

novel\_protein2domain.xls

novel\_protein2GO.xls

novel\_protein2KEGG.xls

protein\_blastp2swissprot\_all.xls

protein\_blastp2swissprot\_bestone.xls

op50\_1

transcripts\_filter.gtf

op50\_2

transcripts\_filter.gtf

op50\_3

transcripts\_filter.gtf

S20mg\_1

transcripts\_filter.gtf

S20mg\_2

transcripts\_filter.gtf

S20mg\_3

transcripts\_filter.gtf

4-DGE

Cluster

Cluster.pdf

Cluster.png

Cluster\_with\_symbol.xls

Cluster.xls

merge

All\_samples\_boxplot.pdf

All\_samples\_boxplot.png

All\_samples\_PCA.pdf

All\_samples\_PCA.png

gene\_sample\_count\_with\_symbol.xls

gene\_sample\_count.xls

gene\_sample\_FPKM\_stat.xls

gene\_sample\_FPKM\_with\_symbol.xls

gene\_sample\_FPKM.xls

gene\_sample\_log2FPKM\_with\_symbol.xls

gene\_sample\_log2FPKM.xls

gene\_sample\_log2normalized\_count\_with\_symbol.xls

gene\_sample\_log2normalized\_count.xls

group1\_boxplot.pdf

group1\_boxplot.png

group1\_PCA.pdf

group1\_PCA.png

sample\_Corr.pdf

sample\_Corr.png

transcript\_sample\_count\_with\_symbol.xls

transcript\_sample\_count.xls

transcript\_sample\_log2normalized\_count\_with\_symbol.xls

transcript\_sample\_log2normalized\_count.xls

overlap

genelist\_overlap\_log2normalized\_count\_heatmap.pdf

genelist\_overlap\_log2normalized\_count\_heatmap.png

genelist\_overlap\_log2normalized\_count\_heatmap.xls

genelist\_overlap\_with\_symbol.xls

S20mg\_vs\_op50

gene\_exp\_significant\_log2normalized\_count\_heatmap.pdf

gene\_exp\_significant\_log2normalized\_count\_heatmap.png

gene\_exp\_significant\_with\_symbol\_GO\_KEGG\_FPKM.xls

gene\_exp\_significant\_with\_symbol\_GO\_KEGG.xls

gene\_exp\_with\_symbol.xls

Scatter\_Plot.pdf

Scatter\_Plot.png

Volcano\_and\_Scatter\_Plots.pdf

Volcano\_and\_Scatter\_Plots.png

Volcano\_Plot.pdf

Volcano\_Plot.png

5-AS

merge

A3SS.xls

A5SS.xls

AS\_event\_stat.pdf

AS\_event\_stat.png

AS\_event\_stat.xls

MXE.xls

RI.xls

SE.xls

S20mg\_vs\_op50

all\_A3SS\_with\_symbol.xls

all\_A5SS\_with\_symbol.xls

all\_MXE\_with\_symbol.xls

all\_RI\_with\_symbol.xls

all\_SE\_with\_symbol.xls

diff\_A3SS\_with\_symbol.xls

diff\_A5SS\_with\_symbol.xls

diff\_MXE\_with\_symbol.xls

diff\_RI\_with\_symbol.xls

diff\_SE\_with\_symbol.xls

6-FUN

S20mg\_vs\_op50

GO\_KEGG

allsymbol.txt

desymbol.txt

GO\_enrichment\_significant\_topGO\_and\_gene\_exp.xls

GO\_enrichment\_significant\_topGO.xls

GO\_enrichment\_topGO\_and\_gene\_exp.xls

GO\_enrichment\_topGO\_Pvalue\_Barplot.pdf

GO\_enrichment\_topGO\_Pvalue\_Barplot.png

GO\_enrichment\_topGO.xls

GO\_tree\_topGO\_BP\_MF\_CC.pdf

GO\_tree\_topGO\_BP\_MF\_CC.png

KEGG\_enrichment\_2D.pdf

KEGG\_enrichment\_2D.png **-  PATHWAY富集分析散点展示**

KEGG\_enrichment\_and\_gene\_exp.xls

KEGG\_enrichment\_GenePercentage\_Barplot.pdf

KEGG\_enrichment\_GenePercentage\_Barplot.png

KEGG\_enrichment\_KEGG2Symbol\_Net.pdf

KEGG\_enrichment\_KEGG2Symbol\_Net.png

KEGG\_enrichment\_Pathway2Pathway\_Net.pdf

KEGG\_enrichment\_Pathway2Pathway\_Net.png

KEGG\_enrichment\_Pvalue\_Barplot.pdf

KEGG\_enrichment\_Pvalue\_Barplot.png

KEGG\_enrichment\_significant\_and\_gene\_exp.xls

KEGG\_enrichment\_significant\_with\_web.xls

KEGG\_enrichment\_significant.xls

KEGG\_enrichment.xls

Rplots.pdf

strings\_network.pdf

strings\_network.png

strings\_network.xls

top50\_network.xls

GO\_KEGG\_Down

allsymbol.txt

desymbol.txt

GO\_enrichment\_significant\_topGO\_and\_gene\_exp.xls

GO\_enrichment\_significant\_topGO.xls

GO\_enrichment\_topGO\_and\_gene\_exp.xls

GO\_enrichment\_topGO\_Pvalue\_Barplot.pdf

GO\_enrichment\_topGO\_Pvalue\_Barplot.png

GO\_enrichment\_topGO.xls

GO\_tree\_topGO\_BP\_MF\_CC.pdf

GO\_tree\_topGO\_BP\_MF\_CC.png

KEGG\_enrichment\_2D.pdf

KEGG\_enrichment\_2D.png **-  PATHWAY富集分析散点展示**

KEGG\_enrichment\_and\_gene\_exp.xls

KEGG\_enrichment\_GenePercentage\_Barplot.pdf

KEGG\_enrichment\_GenePercentage\_Barplot.png

KEGG\_enrichment\_KEGG2Symbol\_Net.pdf

KEGG\_enrichment\_KEGG2Symbol\_Net.png

KEGG\_enrichment\_Pathway2Pathway\_Net.pdf

KEGG\_enrichment\_Pathway2Pathway\_Net.png

KEGG\_enrichment\_Pvalue\_Barplot.pdf

KEGG\_enrichment\_Pvalue\_Barplot.png

KEGG\_enrichment\_significant\_and\_gene\_exp.xls

KEGG\_enrichment\_significant\_with\_web.xls

KEGG\_enrichment\_significant.xls

KEGG\_enrichment.xls

Rplots.pdf

strings\_network.pdf

strings\_network.png

strings\_network.xls

top50\_network.xls

GO\_KEGG\_Up

allsymbol.txt

desymbol.txt

GO\_enrichment\_significant\_topGO\_and\_gene\_exp.xls

GO\_enrichment\_significant\_topGO.xls

GO\_enrichment\_topGO\_and\_gene\_exp.xls

GO\_enrichment\_topGO.xls

GO\_tree\_topGO\_BP\_MF\_CC.pdf

GO\_tree\_topGO\_BP\_MF\_CC.png

KEGG\_enrichment\_2D.pdf

KEGG\_enrichment\_2D.png **-  PATHWAY富集分析散点展示**

KEGG\_enrichment\_and\_gene\_exp.xls

KEGG\_enrichment\_GenePercentage\_Barplot.pdf

KEGG\_enrichment\_GenePercentage\_Barplot.png

KEGG\_enrichment\_KEGG2Symbol\_Net.pdf

KEGG\_enrichment\_KEGG2Symbol\_Net.png

KEGG\_enrichment\_Pathway2Pathway\_Net.pdf

KEGG\_enrichment\_Pathway2Pathway\_Net.png

KEGG\_enrichment\_Pvalue\_Barplot.pdf

KEGG\_enrichment\_Pvalue\_Barplot.png

KEGG\_enrichment\_significant\_and\_gene\_exp.xls

KEGG\_enrichment\_significant\_with\_web.xls

KEGG\_enrichment\_significant.xls

KEGG\_enrichment.xls

strings\_network.pdf

strings\_network.png

strings\_network.xls

top50\_network.xls

GN-method

mRNAseq\_method\_Chinese.pdf

mRNAseq\_method\_English.pdf

  
目录数目：36，文件数目：245  
  


# 1. 实验流程

## 1.1 抽提与质检

1）从样品中提取Total RNA，mRNA测序建库要求RNA总量1 μg

2）利用琼脂糖凝胶电泳检测RNA完整性（28S:18S ≥ 1.5）

3）Nanodrop检测RNA纯度（OD260/280比值为1.8~2.2）

4）Qubit精确定量RNA总量（≥ 500 ng）

  

注：抽提与质检流程图

  
  


## 1.2 文库构建

1）mRNA富集。真核生物mRNA用带有Oligo (dT)的磁珠富集，

原核生物mRNA则用试剂盒去除rRNA进行富集。

2）片段化mRNA。将mRNA随机打断成200bp左右的短片段。

3）链特异性合成cDNA。以mRNA为模板，利用随机引物反转录

合成一链cDNA，合成二链cDNA时dNTPs中的dTTP被dUTP代替。

4）连接测序接头。利用AMPure XP beads纯化双链cDNA，由End Repair

Mix末端补平，并加A尾和测序接头。

5）二链cDNA消化。利用USER酶消化二链cDNA，

使其文库只含一链cDNA。

6）PCR富集。PCR扩增约15个cycles得到最终的cDNA文库。

7）文库检测和定量。

  

注：文库构建流程示意图

  


## 1.3 上机测序

1）桥式扩增。在cBot仪器上进行桥式PCR扩增，生成clusters。

2）Illumina测序平台，采用2\*150测序模式进行边合成边测序（Sequencing by Synthesis）。在测序的flow cell中加入四种荧光标记的dNTP、DNA聚合酶以及接头引物进行扩增，在每一个测序簇延伸互补链时，每加入一个被荧光标记的dNTP就能释放出相对应的荧光，测序仪通过捕获荧光信号，并通过计算机软件将光信号转化为测序峰，从而获得待测片段的序列信息。

3）测序片段被高通量测序仪测得的图像数据经CASAVA碱基识别转化为序列数据（reads），文件为fastq格式，其中主要包含测序片段的序列信息以及其对应的测序质量信息。原始数据为fastq格式的文件，其中包含了reads的碱基序列和测序质量值，每条read以4行信息表示，第一行为read在Illumina测序中的唯一标识符，第二行为碱基序列，第三行+号后面省略read标识符，第四行为碱基质量值。

  

  
  


# 2. 测序数据质量统计

## 2.1 原始数据质控

目录链接

  
  
  
  

本实验采用illumina Novaseq6000测序平台的双端测序模式对多个样本进行高通量测序，如果是SMARTER建库则去除单细胞SMARTER建库接头，根据illumina测序数据的低质量分数集中于末端的分布特点，利用Skewer软件对测序数据从3'端动态去除接头序列片段和低质量片段, 利用FastQC软件对预处理数据进行质量控制分析以及统计Q20，Q30的碱基比例。

质控统计总表见报告->1-QC > QC.xls

### 测序数据质量统计结果(QC.xls)

| Sample | Raw reads | Raw read pairs | Raw bases | Clean reads | Clean bases | Clean read pairs | Average length | Clean reads % | Clean bases % | Q20 % | Q30 % |
| --- | --- | --- | --- | --- | --- | --- | --- | --- | --- | --- | --- |
| op50\_1 | 48960634 | 24480317 | 7393055734 | 48722368 | 7329179901 | 24361184 | 150.4 | 99.51% | 99.14% | 97.9% | 93.75% |
| op50\_2 | 37891450 | 18945725 | 5721608950 | 37617934 | 5663877403 | 18808967 | 150.6 | 99.28% | 98.99% | 97.85% | 93.75% |
| op50\_3 | 43598110 | 21799055 | 6583314610 | 43387188 | 6503913428 | 21693594 | 149.9 | 99.52% | 98.79% | 98.2% | 94.6% |
| S20mg\_1 | 38992514 | 19496257 | 5887869614 | 38773826 | 5829581198 | 19386913 | 150.3 | 99.44% | 99.01% | 98% | 94.15% |
| S20mg\_2 | 48184364 | 24092182 | 7275838964 | 47934352 | 7208398360 | 23967176 | 150.4 | 99.48% | 99.07% | 98% | 93.85% |
| S20mg\_3 | 46687868 | 23343934 | 7049868068 | 46434300 | 6988116693 | 23217150 | 150.5 | 99.46% | 99.12% | 98.1% | 94.15% |

  
  

| 表头 | 说明 |
| --- | --- |
| Sample | 样本名 |
| Raw reads | 原始序列数 |
| Raw read pairs | 原始序列对 |
| Raw bases | 原始序列碱基数 |
| Clean reads | 去除低质量片段后序列数 |
| Clean bases | 去除低质量片段后序列碱基数 |
| Clean read pairs | 去除低质量片段后序列对 |
| Average length | 去除低质量片段后序列平均长度 |
| Clean reads % | 去除低质量片段后序列数占原始序列数的比例 |
| Clean bases % | 去除低质量片段后序列碱基数占原始序列碱基数的比例 |
| Q20 % | 质量分数大于等于20 的碱基占高质量片段碱基总数的比例 |
| Q30 % | 质量分数大于等于30 的碱基占高质量片段碱基总数的比例 |

  
  


## 2.2 Reads质量评估

目录链接

  
  
  
  

核苷酸序列中鸟嘌呤(G)和胞嘧啶(C)所占的比例称为GC含量。GC含量在物种间存在一定特异性，但由于反转录过程中所使用的6bp随机引物，会引起前几位碱基在核苷酸组成上有一定偏好性，产生正常波动，随后则趋于稳定。对于链特异性建库而言，由于只保留了单链信息，可能会出现AT分离或GC分离现象。样本的GC含量分布如下图所示。

结果见报告->1-QC > Sample > Read1\_fastqc\_data\_per\_base\_quality.pdf

### 序列的质量分数分布(\*\_fastqc\_data\_per\_base\_quality.png)

  
  

注：图A:序列每个碱基位置的质量分数分布图， 横坐标代表序列的碱基位置， 纵坐标代表该碱基位置的质量分数分布。图B:序列长度分布图， 横坐标代表序列长度， 纵坐标代表序列数目的比例。图C:GC 百分比序列数密度分布图， 横坐标代表GC 百分比， 纵坐标代表序列数比例。

关于1-QC目录下其他文件的说明：

1-QC/strand\_specific.xls:链特异性检测结果文件。链特异性通常是“1+-，1-+，2++，2--”这种，表示如果read1在+链，相对的gene其实是在-链（reverse）。

  

  
  


# 3. 基因组比对

## 3.1 基因组比对统计

目录链接

  
  
  
  

针对每个样本，利用STAR软件将预处理序列与测序物种的参考基因组序列进行序列比对，参考基因组版本以及已知转录本在基因组位置信息文件版本文件和下载地址见下面的数据库说明部分。 STAR软件的原理是将整条序列分割成多个种子序列比对基因组序列，有些序列的比对是连续的，有些序列的比对是非连续的。 其中比配参数要求设置为--twopassMode Basic|--outSAMstrandField intronMotif|--alignSJstitchMismatchNmax 5 -1 5 5, 其余为默认，采用RSEQC对比对情况进行统计。

基因组比对统计结果见报告->2-MAP>MAP.xls

### Reads的参考基因组比对统计结果(MAP.xls)

| Sample | Total clean reads | Total mapped | Mapped ratio(%) | Multiple mapped | Unique mapped | Read-1 | Read-2 | Reads map to '+' | Reads map to '-' | Non-Splice reads | Splice reads | Reads Proper pair |
| --- | --- | --- | --- | --- | --- | --- | --- | --- | --- | --- | --- | --- |
| op50\_1 | 48722368 | 48131966 | 98.8% | 1119137 | 47012829 | 23508698 | 23504131 | 23506441 | 23506388 | 27666188 | 19346641 | 47008258 |
| op50\_2 | 37617934 | 37066854 | 98.5% | 994519 | 36072335 | 18037988 | 18034347 | 18036081 | 18036254 | 21209546 | 14862789 | 36068692 |
| op50\_3 | 43387188 | 42901797 | 98.9% | 1140657 | 41761140 | 20881495 | 20879645 | 20880542 | 20880598 | 24789155 | 16971985 | 41759288 |
| S20mg\_1 | 38773826 | 38398656 | 99% | 851054 | 37547602 | 18774211 | 18773391 | 18773784 | 18773818 | 22185390 | 15362212 | 37546782 |
| S20mg\_2 | 47934352 | 47420863 | 98.9% | 1213426 | 46207437 | 23104656 | 23102781 | 23103696 | 23103741 | 27569739 | 18637698 | 46205558 |
| S20mg\_3 | 46434300 | 45983003 | 99% | 1206330 | 44776673 | 22389581 | 22387092 | 22388295 | 22388378 | 26493267 | 18283406 | 44774182 |

  
  

| 表头 | 说明 |
| --- | --- |
| Sample | 样本名 |
| Total reads | 序列总数 |
| Total mapped | 匹配序列数 |
| Mapped ratio(%) | 匹配序列占序列总数百分比 |
| Uniquely mapped | 唯一匹配序列数 |
| Multiple mapped | 多个位置匹配序列数 |
| Reads map to '+' | 正义链匹配的序列数 |
| Reads map to '-' | 反义链匹配的序列数 |
| Non-splice reads | 不出现剪切Splice形式的序列数 |
| Splice reads | 出现剪切Splice 形式的序列数 |
| Reads Proper pair | 比对中正确配对的序列数 |

  


## 3.2 染色体reads统计

目录链接

  
  
  
  

Reads比对到参考基因组结果，统计每条染色体的序列数，然后计算参考基因组每5kb内计算平均深度后取log2，完成参考基因组密度分布统计，用于检测参考基因组上测序序列分布的异常情况。

结果见报告-> 2-MAP > Sample > RSEQC.png

### Reads染色体的序列数统计图(RSEQC.png)

  

注：图A:Reads比对外显子，内含子，基因间隔区域分布统计饼图，详细说明: 计算比对参考基因组的Reads在不同基因成分内的覆盖情况 (例如 CDS exon，5'UTR exon，3'UTR exon，Intron)，Reads比对分布评估统计每个区域比对到的唯一reads的数目，即如果同一reads比对到同一区域，但该区域可能被同时注释为外显子和内含子(两个不同的转录本元件)，则按一定的优先顺序只记录一次该Reads在优先基因组区域内的比对数量，区域计数的优先次序按 CDS exons > UTR exons > Introns 排序。例如，如果一个Reads比对到一个属于CDS exon和intron区域，该Reads将被标注为属于CDS exons。

图B:剪切位点注释统计饼状图，详细说明: 可变性剪切位点注释评估分析过程是依据各样本比对参考基因组与已知基因模型注释信息，比较已知 splice junction (剪切位点) 获得当前转录组内的新 splice junction (剪切位点) 数目与比例。每个探测到的 splice junction 可被划分为3个独立类型，(1)Annotated:全部属于已知基因模型注释内的剪切位点，即包括剪切位点的5端剪切位点和3端剪切位点。(2)complete\_novel:全部属于新的剪切位点，剪切位点两端均不属于已知基因模型中被注释的部分。(3)partial\_novel:某部分(5'SS or 3'SS)属于已知基因模型注释内的剪切位点，另外部分(3'SS or 5'SS) 属于新的剪切位点的情形。

图C:转录本覆盖均一性分布图，横坐标代表转录本长度归一化后100nt的位置，纵坐标代表覆盖在每个子区域位置上的reads数目，详细说明: 转录本覆盖匀一性分布评估用于检测转录本内测序reads是否均一并且是否存在5'/3'偏差，评估方法是: 分析过程中将所有 已知转录本归一化为长度100nt长度范围的区域并计算覆盖在每个子区域位置上的reads数目。最终提供描述基因体5'/3'方向的reads覆盖度分布图，正确的分布状态不存在5/3偏差，RNA-Seq测序实验质量很好，可用于后续的进一步数据分析。

  

## 3.3 Reads分布统计

目录链接

  
  
  
  

Reads分布统计结果见报告-> 2-MAP > Sample > RSEQC\_exon\_intron\_distribution.txt

### Reads参考基因组分布统计(RSEQC\_exon\_intron\_distribution.txt)

| 表头 | 表头说明 |
| --- | --- |
| Total Reads | 去除低质量序列，重复序列， 非唯一匹配的序列后的总序列数 |
| Total Tags | 序列切割(spliced)一次计数成2个标签(tags)切割2次计数3个标签，所以Total tags >= Total Reads |
| Total Assigned Tags | 能正确分配到表格中10组分类的标签数目 |
| CDS\_Exons | 基因区域的编码区域 |
| 5'UTR Exons | 基因区域的5端UTR区域 |
| 3'UTR Exons | 基因区域的3端UTR区域 |
| Introns | 内含子区域 |
| TSS\_up\_1kb | 转录起始位置上游1Kb内 |
| TSS\_up\_5kb | 转录起始位置上游1Kb到5Kb内 |
| TSS\_up\_10kb | 转录起始位置上游5Kb到10Kb内 |
| TES\_down\_1kb | 转录终止位置下游1Kb内 |
| TES\_down\_5kb | 转录终止位置下游1Kb到5Kb内 |
| TES\_down\_10kb | 转录终止位置下游5Kb到10Kb内 |

  
  

  
  


# 4. 转录本组装结果

## 4.1 转录本组装原理

目录链接

  
  
  
  

针对每个样本，从Reads比对到参考基因组的结果利用StringTie软件基于已知转录本在基因组上位置信息文件作为指导组装样本中的转录本，过滤表达量等于0的转录本， 分别将所有样本的StringTie组装好的转录本进行再次组装。StringTie组装原理如下，首先从BAM比对结果识别Spliced reads，这些序列可能是由于不同形式的mRNA剪切异构体(spliced isoforms)形成的， 它们相互之间连通成Splice Graph，每个节点(Node)表示Exon，根据Exon区域和Exon之间连接区域序列覆盖深度信息构建最有可能的mRNA结构形式，用于评估其基因的表达量(count值和FPKM值)情况。

针对StringTie组装转录本，采用gffcompare与已知基因参考基因组位置信息的比较结果可以对组装转录本进行分类。将已知基因间隔区域的新转录本(u)、新可变性剪切的转录本(j) 、与已知多个外显子复杂重叠的转录本(o)、与已知内含子链特异完全匹配的转录本(i)、与已知外显子重叠的反义转录本(x)、与已内含子重叠的反义转录本(s)，满足长度大于等于200bp归类为候选新转录本。 统计新转录本的外显子个数、转录本的开始位置与结束位置、转录本长度、对应的已知基因。 根据StringTie组装转录本与已知基因对应关系，与已知基因转录本在参考基因组位置信息进行比较对基因结构的开始位置与结束位置进行优化。

### StringTie的组装原理

  


结果见报告-> 3-ASSEMBLY

## 4.2 组装转录本分类

目录链接

  
  
  
  

### 根据分类对应的组装转录本总数柱状图

  
  

### 组装转录本分类说明(assembled\_transcripts\_\*.gtf)

| ClassCode | Description |
| --- | --- |
| c | Contained(包含于已知基因的转录本) |
| = | Complete match of intron chain(与已知基因的内含子结构完全匹配的转录本) |
| u | Unknown, intergenic transcript(已知基因间隔区域的新转录本) |
| j | Potentially novel isoform (fragment): at least one splice junction is shared with a reference transcript(新可变性剪切的转录本) |
| i | A transfrag falling entirely within a reference intron(与已知内含子链特异完全匹配的转录本) |
| o | Generic exonic overlap with a reference transcript(与已知多个外显子复杂重叠的转录本) |
| e | Single exon transfrag overlapping a reference exon and at least 10 bp of a reference intron, indicating a possible pre-mRNA fragment.(与已知单个外显子重叠的转录本) |
| x | Exonic overlap with reference on the opposite strand(与已知外显子重叠的反义转录本) |
| s | An intron of the transfrag overlaps a reference intron on the opposite strand (likely due to read mapping errors)(与已内含子重叠的反义转录本) |
| p | Possible polymerase run-on fragment(within 2Kbases of a reference transcript) (与可能RNA聚合酶错误转录已知转录本有关转录本) |
| r | A transfrag overlaps repetitive region(与重复区域重叠的转录本) |

  

### 新转录本基因结构结果格式说明(isoform\_exon\_structure\_info.xls)

显示 10203050100150全文 行  
ASSEMBLY-merge
 

| New\_transcriptid | geneid | symbol | description | transcript\_start | transcript\_end | Num\_of\_exons | exon\_sts | exon\_ens | transcript\_length | strand |
| --- | --- | --- | --- | --- | --- | --- | --- | --- | --- | --- |
| MSTRG.3.2 | WBGene00022278 | rcor-1 | --- | 17497 | 26643 | 10 | 17497,18006,19015,20271,20848,21013,24651,24929,25273,26371 | 17958,18115,19241,20478,20964,21139,24845,25090,25472,26643 | 2081 | - |
| MSTRG.3.3 | WBGene00022278 | rcor-1 | --- | 17502 | 25885 | 10 | 17502,18006,19015,20271,20848,21013,24651,24929,25273,25601 | 17958,18115,19241,20478,20964,21139,24845,25090,25472,25885 | 2088 | - |
| MSTRG.5.1 | WBGene00022279 | sesn-1 | --- | 27591 | 33146 | 6 | 27591,29100,29769,31769,32412,33037 | 28405,29367,30424,32072,32542,33146 | 2284 | - |
| MSTRG.5.2 | WBGene00022279 | sesn-1 | --- | 27591 | 34794 | 7 | 27591,29100,29769,31769,32412,33042,34693 | 28405,29367,30424,32072,32542,33144,34794 | 2379 | - |
| MSTRG.5.3 | WBGene00022279 | sesn-1 | --- | 27601 | 34745 | 7 | 27601,29100,29769,31769,32412,33042,34612 | 28405,29367,30424,32072,32542,33144,34745 | 2401 | - |
| MSTRG.7.2 | WBGene00021677 | pgs-1 | --- | 52292 | 54360 | 4 | 52292,52466,53266,53944 | 52410,52572,53695,54360 | 1073 | + |
| MSTRG.9.2 | WBGene00000812 | csk-1 | --- | 71425 | 80633 | 13 | 71425,71838,72511,72647,72941,73605,74625,75171,75951,76949,77713,79313,80306 | 71511,71932,72590,72897,73017,73766,74808,75490,76112,77153,77799,79447,80633 | 2173 | + |
| MSTRG.9.3 | WBGene00000812 | csk-1 | --- | 71617 | 80633 | 12 | 71617,72511,72647,73605,74625,75171,75951,76949,77713,78153,79313,80306 | 71932,72590,73017,73766,74808,75490,76112,77153,77799,78170,79447,80633 | 2368 | + |
| MSTRG.9.4 | WBGene00000812 | csk-1 | --- | 71726 | 80633 | 13 | 71726,72511,72647,72941,73605,74625,75171,75951,76949,77713,78153,79313,80306 | 71932,72590,72897,73017,73766,74808,75490,76112,77153,77799,78170,79447,80633 | 2216 | + |
| MSTRG.10.2 | WBGene00021683 | Y48G1C.10 | phosphatase activity | 81201 | 90957 | 16 | 81201,81879,82016,82586,83181,84190,84388,84614,84836,86824,87034,87520,88268,88566,89372,90419 | 81272,81963,82137,82676,83420,84313,84567,84738,84985,86904,87223,87734,88500,88706,89584,90957 | 2801 | + |
| MSTRG.13.2 | WBGene00021676 | pid-2 | --- | 93024 | 94884 | 5 | 93024,93609,94064,94269,94622 | 93556,94015,94208,94568,94884 | 1648 | + |
| MSTRG.15.3 | WBGene00021681 | Y48G1C.8 | --- | 96544 | 100960 | 6 | 96544,97600,98285,99629,100139,100395 | 96992,97960,99058,99789,100323,100960 | 2496 | - |
| MSTRG.16.2 | WBGene00004274 | rab-11.1 | --- | 108770 | 110057 | 3 | 108770,109492,109835 | 109446,109783,110057 | 1192 | - |
| MSTRG.17.1 | WBGene00018774 | F53G12.9 | --- | 111038 | 113721 | 10 | 111038,111161,111510,111755,112019,112527,112700,113239,113426,113596 | 111065,111260,111707,111971,112481,112652,112903,113382,113527,113721 | 1708 | + |
| MSTRG.17.2 | WBGene00004418 | rpl-7 | --- | 111038 | 112272 | 4 | 111038,111161,111510,112019 | 111065,111260,111971,112272 | 844 | + |
| MSTRG.21.3 | WBGene00003229 | mex-3 | --- | 128697 | 134065 | 5 | 128697,129167,132103,132612,133414 | 129124,129333,132553,132693,134065 | 1780 | + |
| MSTRG.24.2 | WBGene00000253 | bli-3 | --- | 146486 | 154850 | 19 | 146486,146793,147054,147784,148099,149060,149303,149520,150391,150734,151227,151461,152069,152407,152706,152956,153395,153599,154759 | 146748,146924,147731,147994,148223,149259,149472,149817,150688,151182,151383,151674,152360,152658,152909,153345,153553,153680,154850 | 4666 | - |
| MSTRG.24.3 | WBGene00000253 | bli-3 | --- | 146486 | 154850 | 18 | 146486,146793,147054,147784,148099,149060,149303,149520,150391,150734,151227,151461,152069,152407,152706,152956,153395,154759 | 146748,146924,147731,147994,148223,149259,149472,149817,150688,151182,151383,151674,152360,152658,152909,153345,153680,154850 | 4711 | - |
| MSTRG.25.2 | WBGene00004225 | ptr-11 | --- | 160190 | 161309 | 3 | 160190,160712,161257 | 160546,160999,161309 | 698 | - |
| MSTRG.26.3 | WBGene00018958 | F56C11.6 | --- | 173422 | 175932 | 7 | 173422,173775,174605,174878,175097,175579,175755 | 173725,174312,174832,175053,175241,175708,175932 | 1699 | + |
| MSTRG.28.2 | WBGene00018955 | F56C11.3 | --- | 182079 | 182772 | 4 | 182079,182394,182545,182672 | 182345,182500,182626,182772 | 557 | - |
| MSTRG.30.2 | WBGene00021666 | Y48G1BL.7 | --- | 209302 | 211634 | 5 | 209302,209958,210152,211257,211495 | 209550,210100,211176,211384,211634 | 1685 | + |
| MSTRG.31.2 | WBGene00004246 | puf-10 | --- | 212256 | 215318 | 4 | 212256,213524,214169,214858 | 212496,214113,214186,215318 | 1310 | - |
| MSTRG.32.1 | WBGene00021662 | snpc-3.3 | --- | 215515 | 217453 | 3 | 215515,216145,216331 | 216092,216277,217453 | 1834 | + |
| MSTRG.35.1 | WBGene00021675 | Y48G1BR.1 | --- | 219735 | 264213 | 12 | 219735,254701,254976,255195,255370,255818,256316,257418,258021,258548,259107,264174 | 219759,254802,255079,255325,255446,255960,256643,257661,258358,258683,259157,264213 | 1719 | - |
| MSTRG.35.3 | WBGene00021664 | Y48G1BL.5 | --- | 219735 | 221575 | 6 | 219735,220004,220223,220398,220996,221498 | 219828,220107,220350,220474,221456,221575 | 942 | - |
| MSTRG.37.1 | WBGene00021671 | Y48G1BM.6 | --- | 233332 | 237597 | 8 | 233332,233466,233626,233783,234038,236253,236520,237531 | 233418,233573,233733,233983,236206,236444,236666,237597 | 3079 | - |
| MSTRG.38.1 | WBGene00021673 | Y48G1BM.8 | --- | 248655 | 251623 | 8 | 248655,248732,248946,249495,249675,250909,251206,251476 | 248687,248899,249050,249587,249869,251160,251406,251623 | 1195 | - |
| MSTRG.40.2 | WBGene00016903 | marc-4 | --- | 280120 | 286093 | 6 | 280120,280311,284309,285008,285558,285835 | 280206,280431,284538,285211,285638,286093 | 982 | + |
| MSTRG.40.3 | WBGene00016903 | marc-4 | --- | 280120 | 286092 | 6 | 280120,283519,284309,285008,285558,285835 | 280431,283649,284538,285211,285638,286092 | 1216 | + |
| MSTRG.40.4 | WBGene00016903 | marc-4 | --- | 280120 | 286092 | 6 | 280120,280311,284309,285008,285558,285835 | 280206,283649,284538,285211,285638,286092 | 4199 | + |
| MSTRG.40.5 | WBGene00016903 | marc-4 | --- | 280308 | 286093 | 5 | 280308,283519,284309,285008,285835 | 280431,283649,284538,285638,286093 | 1375 | + |
| MSTRG.41.3 | WBGene00016905 | ztf-3 | --- | 286156 | 289185 | 5 | 286156,287509,288558,288750,288973 | 286997,288003,288644,288902,289185 | 1790 | - |
| MSTRG.45.1 | WBGene00235385 | Y48G1A.7 | --- | 316657 | 318141 | 4 | 316657,317192,317457,318001 | 317144,317414,317606,318141 | 1002 | - |
| MSTRG.45.2 | WBGene00235385 | Y48G1A.7 | --- | 316659 | 318072 | 3 | 316659,317192,318001 | 317144,317606,318072 | 973 | - |
| MSTRG.46.1 | WBGene00002079 | xpo-2 | --- | 318390 | 341843 | 6 | 318390,319738,321075,322785,323143,341716 | 319128,320417,322388,323093,323352,341843 | 3380 | - |
| MSTRG.46.2 | WBGene00002079 | xpo-2 | --- | 318546 | 323352 | 6 | 318546,319092,319738,321075,322785,323143 | 319025,319128,320417,322388,323093,323352 | 3030 | - |
| MSTRG.47.1 | WBGene00021660 | nol-14 | --- | 318529 | 330987 | 8 | 318529,323855,323979,324289,325299,326959,327847,330111 | 318546,323934,324240,324653,326048,327245,327979,330987 | 2772 | + |
| MSTRG.49.3 | WBGene00020087 | R119.1 | --- | 343072 | 363909 | 15 | 343072,343894,345021,346375,346561,346793,352880,357248,357376,358291,360225,361249,363070,363655,363772 | 343649,344033,345830,346516,346742,347044,353065,357327,357482,358758,361198,361593,363603,363714,363909 | 4996 | - |
| MSTRG.49.4 | WBGene00020087 | R119.1 | --- | 357165 | 363909 | 7 | 357165,357376,358291,360225,363070,363655,363772 | 357327,357482,358758,361593,363603,363714,363909 | 2839 | - |
| MSTRG.51.2 | WBGene00021657 | Y48G1A.1 | --- | 348474 | 357193 | 13 | 348474,348677,349352,350185,350641,351794,352866,353744,354381,355374,355538,356834,356994 | 348619,348904,349767,350346,350985,352080,353172,353964,354699,355481,355817,356946,357193 | 3132 | + |
| MSTRG.52.1 | WBGene00020091 | rnp-8 | --- | 364374 | 368319 | 7 | 364374,364517,364736,365531,365644,366653,367746 | 364402,364679,365191,365596,365960,367209,368319 | 2162 | + |
| MSTRG.53.2 | WBGene00020088 | R119.2 | --- | 368576 | 373445 | 5 | 368576,370424,371355,372617,373331 | 369246,370761,371673,372713,373445 | 1540 | - |
| MSTRG.54.1 | WBGene00004143 | pqn-59 | --- | 373571 | 381902 | 9 | 373571,377198,378571,378929,380183,380605,380971,381266,381791 | 373742,377518,378876,380128,380555,380921,381218,381470,381902 | 3254 | - |
| MSTRG.54.2 | WBGene00004143 | pqn-59 | --- | 373572 | 457414 | 11 | 373572,377198,378571,378703,378929,380183,380605,380971,381266,381791,457405 | 373742,377518,378641,378876,380128,380555,380921,381218,381470,381902,457414 | 3202 | - |
| MSTRG.54.3 | WBGene00004143 | pqn-59 | --- | 379177 | 381908 | 5 | 379177,380183,380605,380971,381791 | 380128,380555,380921,381470,381908 | 2260 | - |
| MSTRG.59.2 | WBGene00021026 | W04C9.4 | --- | 461995 | 462543 | 2 | 461995,462371 | 462318,462543 | 497 | + |
| MSTRG.62.2 | WBGene00021024 | W04C9.2 | --- | 488607 | 489905 | 3 | 488607,488768,489700 | 488723,488944,489905 | 500 | + |
| MSTRG.65.1 | WBGene00022036 | Y65B4BL.4 | --- | 502932 | 506325 | 9 | 502932,504057,504225,504323,505506,505660,505820,506093,506289 | 503212,504132,504278,504454,505605,505771,506049,506219,506325 | 1149 | - |
| MSTRG.68.4 | WBGene00022037 | acs-13 | --- | 511054 | 519776 | 5 | 511054,512481,513143,515023,519735 | 511753,513090,514208,515236,519776 | 2632 | - |
| MSTRG.70.1 | WBGene00022033 | Y65B4BL.1 | --- | 524064 | 533039 | 6 | 524064,530971,531267,531790,531947,532619 | 524155,531091,531348,531893,532553,533039 | 1427 | + |
| MSTRG.71.2 | WBGene00022043 | psf-3 | --- | 534455 | 535344 | 3 | 534455,534772,535031 | 534724,534970,535344 | 783 | + |
| MSTRG.72.2 | WBGene00022042 | icd-2 | protein transport | 535791 | 536586 | 2 | 535791,536350 | 536306,536586 | 753 | + |
| MSTRG.73.3 | WBGene00007009 | wwp-1 | --- | 537122 | 542173 | 9 | 537122,537606,538702,539453,539801,540064,540723,541064,541344 | 537560,537835,538911,539727,540008,540384,540983,541285,542173 | 2996 | + |
| MSTRG.73.4 | WBGene00007009 | wwp-1 | --- | 537122 | 542173 | 8 | 537122,537606,538702,539453,539801,540064,540723,541064 | 537560,537835,538911,539727,540008,540384,540983,542173 | 3054 | + |
| MSTRG.79.1 | WBGene00002040 | hum-7 | --- | 582684 | 607601 | 30 | 582684,585841,587154,587333,588629,588814,588928,590158,591535,591742,591937,592441,592572,593229,593537,595186,596334,597636,597885,598561,601170,601419,601604,603126,603375,603524,604330,604895,606569,607361 | 582884,586133,587278,587658,588757,588872,589058,590346,591607,591860,592122,592516,592692,593493,593735,595958,596695,597825,597971,598659,601375,601542,601771,603281,603474,603586,604593,605139,606852,607601 | 5854 | - |
| MSTRG.79.2 | WBGene00002040 | hum-7 | --- | 582684 | 595960 | 16 | 582684,585841,587154,587333,588629,588814,588928,590158,591535,591742,591920,592441,592572,593229,593537,595186 | 582884,586133,587278,587658,588757,588872,589058,590346,591607,591860,592122,592516,592692,593493,593735,595960 | 3284 | - |
| MSTRG.80.1 | WBGene00018923 | eme-1 | --- | 614651 | 636350 | 11 | 614651,615821,623627,624218,625364,625522,627138,629344,631095,633891,636234 | 615001,616114,623716,624375,625472,625629,627363,629451,631285,634797,636350 | 2659 | - |
| MSTRG.80.2 | WBGene00018923 | eme-1 | --- | 614723 | 634538 | 10 | 614723,615821,623627,624218,625364,625522,627138,629344,631175,633891 | 615001,616114,623716,624375,625472,625629,627363,629444,631285,634538 | 2124 | - |
| MSTRG.81.2 | WBGene00018921 | sago-2 | --- | 618160 | 621412 | 9 | 618160,618415,618959,619069,619247,619738,620070,620513,621215 | 618357,618912,619018,619187,619689,620024,620466,621168,621412 | 2856 | + |
| MSTRG.81.3 | WBGene00018921 | sago-2 | --- | 618160 | 621373 | 10 | 618160,618415,618959,619069,619247,619738,620070,620513,620614,621215 | 618357,618912,619018,619187,619689,620024,620466,620551,621168,621373 | 2755 | + |
| MSTRG.82.2 | WBGene00022027 | vps-20 | --- | 636804 | 637842 | 4 | 636804,637065,637250,637764 | 637010,637174,637706,637842 | 853 | + |
| MSTRG.82.3 | WBGene00022027 | vps-20 | --- | 636804 | 637842 | 4 | 636804,637250,637397,637764 | 637174,637337,637706,637842 | 848 | + |
| MSTRG.85.2 | WBGene00022031 | Y65B4A.8 | --- | 659565 | 663686 | 4 | 659565,661314,662932,663066 | 660276,661920,663014,663686 | 2023 | - |
| MSTRG.88.1 | WBGene00021215 | Y18H1A.11 | --- | 680194 | 683472 | 6 | 680194,680421,681314,681490,682932,683227 | 680256,680556,681430,681641,683153,683472 | 936 | + |
| MSTRG.88.3 | WBGene00021215 | Y18H1A.11 | --- | 681355 | 683450 | 3 | 681355,682932,683227 | 681641,683153,683450 | 733 | + |
| MSTRG.91.1 | WBGene00021210 | Y18H1A.4 | --- | 692026 | 694948 | 5 | 692026,692597,693511,694581,694833 | 692370,692684,693847,694736,694948 | 1042 | - |
| MSTRG.95.2 | WBGene00004332 | rec-1 | --- | 717939 | 719816 | 4 | 717939,718288,718657,719498 | 718238,718364,719437,719816 | 1477 | - |
| MSTRG.96.2 | WBGene00004028 | pif-1 | --- | 720493 | 730958 | 10 | 720493,720828,722103,722244,724570,725109,725286,726501,730281,730836 | 720784,720939,722201,722355,724766,725225,725392,726870,730532,730958 | 1781 | + |
| MSTRG.98.2 | WBGene00021213 | Y18H1A.9 | --- | 741916 | 756995 | 13 | 741916,742809,743213,749898,750046,750221,750571,752268,753702,754061,754453,754998,756870 | 742075,743168,743524,749995,750167,750305,750845,752553,753842,754136,754589,755079,756995 | 2260 | - |
| MSTRG.101.1 | WBGene00020283 | T06A4.3 | --- | 763597 | 776322 | 20 | 763597,765138,765432,766048,766326,766527,767583,768165,768274,768500,770462,771451,771806,771968,773164,773435,773700,774968,775110,776164 | 763824,765274,765645,766149,766445,766641,767734,768224,768448,768628,770633,771566,771911,772154,773385,773546,773987,775064,775572,776322 | 3354 | + |
| MSTRG.101.2 | WBGene00020283 | T06A4.3 | --- | 763597 | 776310 | 19 | 763597,765138,765432,766048,766326,766527,767583,768165,768274,768500,771451,771806,771968,773164,773435,773700,774968,775110,776164 | 763824,765274,765645,766149,766445,766641,767734,768224,768448,768628,771566,771911,772154,773385,773546,773987,775064,775572,776310 | 3170 | + |
| MSTRG.104.2 | WBGene00022519 | ZC123.4 | --- | 841754 | 846763 | 9 | 841754,842546,842717,842915,843713,845614,845930,846083,846596 | 841976,842674,842861,843097,844339,845761,845999,846142,846763 | 1753 | - |
| MSTRG.104.3 | WBGene00022519 | ZC123.4 | --- | 841769 | 846765 | 10 | 841769,842546,842717,842915,843713,844061,845614,845821,846083,846596 | 841976,842674,842861,843097,844012,844339,845761,845999,846142,846765 | 1801 | - |
| MSTRG.104.4 | WBGene00022519 | ZC123.4 | --- | 841772 | 846765 | 9 | 841772,842546,842717,842915,843713,844061,845614,846083,846596 | 841976,842674,842861,843097,844012,844339,845999,846142,846765 | 1857 | - |
| MSTRG.105.1 | WBGene00022388 | Y95B8A.8 | --- | 853294 | 872103 | 14 | 853294,853453,853561,853697,853828,853986,858489,861486,864404,865731,866763,868610,870194,871654 | 853376,853487,853626,853769,853930,854104,858835,861919,864819,865934,866858,868721,870324,872103 | 2669 | + |
| MSTRG.105.2 | WBGene00022388 | Y95B8A.8 | --- | 853444 | 872103 | 13 | 853444,853561,853697,853828,853986,858489,861486,864398,865731,866763,868610,870194,871654 | 853487,853626,853769,853930,854104,858835,861919,864819,865934,866858,868721,870324,872103 | 2601 | + |
| MSTRG.105.3 | WBGene00022388 | Y95B8A.8 | --- | 853451 | 872103 | 12 | 853451,853561,853828,853986,858489,861486,864398,865731,866763,868610,870194,871654 | 853487,853769,853930,854104,858835,861919,864819,865934,866858,868721,870324,872103 | 2664 | + |
| MSTRG.106.1 | WBGene00022389 | pde-6 | --- | 872504 | 882575 | 10 | 872504,874649,875521,877706,878217,878406,879483,879592,881109,881279 | 873626,874742,876649,877914,878318,878457,879536,879671,881201,882575 | 4233 | - |
| MSTRG.107.2 | WBGene00022386 | Y95B8A.6 | --- | 882918 | 890194 | 6 | 882918,884904,886507,887880,888150,889674 | 883164,885040,886707,888066,888346,890194 | 1490 | + |
| MSTRG.107.3 | WBGene00022386 | Y95B8A.6 | --- | 882918 | 890170 | 6 | 882918,884904,886507,887880,888150,889674 | 883164,885040,886707,888062,888346,890170 | 1462 | + |
| MSTRG.109.2 | WBGene00001678 | gpa-16 | --- | 897286 | 905904 | 8 | 897286,898173,900613,903103,903253,903382,904611,905734 | 897352,898349,900800,903190,903329,903511,904844,905904 | 1132 | + |
| MSTRG.112.1 | WBGene00003548 | nas-30 | --- | 938464 | 947710 | 15 | 938464,940221,940512,941232,943666,944027,944230,945230,945512,945631,945887,946220,946398,947157,947317 | 938675,940307,940583,941361,943973,944182,944342,945463,945578,945834,946013,946348,946608,947266,947710 | 2554 | + |
| MSTRG.113.3 | WBGene00003238 | mig-1 | --- | 948569 | 957373 | 8 | 948569,949046,950847,951019,951175,952677,952951,957192 | 948791,949500,950955,951110,951315,952889,953092,957373 | 1557 | - |
| MSTRG.114.1 | WBGene00018787 | cutl-20 | --- | 963700 | 973133 | 13 | 963700,964114,966476,966647,966779,968235,968363,969237,969893,971460,972648,972867,972987 | 964052,964196,966598,966733,966923,968317,968488,969838,969983,972086,972720,972937,973133 | 2611 | - |
| MSTRG.114.2 | WBGene00018787 | cutl-20 | --- | 963700 | 973133 | 13 | 963700,964114,966476,966647,966779,968235,968363,969237,969893,971460,972648,972867,972987 | 964052,964196,966598,966733,966923,968317,968488,969838,969983,971950,972720,972937,973133 | 2475 | - |
| MSTRG.114.3 | WBGene00018787 | cutl-20 | --- | 963700 | 976550 | 11 | 963700,964114,966476,966647,966779,968235,968363,969237,969893,971460,976481 | 964052,964196,966598,966733,966923,968317,968488,969838,969983,972086,976550 | 2390 | - |
| MSTRG.114.4 | WBGene00018787 | cutl-20 | --- | 963700 | 976550 | 11 | 963700,964114,966476,966647,966779,968235,968363,969237,969893,971460,976481 | 964052,964196,966598,966733,966923,968317,968488,969838,969983,971950,976550 | 2254 | - |
| MSTRG.114.5 | WBGene00018787 | cutl-20 | --- | 963719 | 976550 | 10 | 963719,964114,966476,966647,966779,968235,969237,969893,971460,976481 | 964052,964196,966598,966733,966923,968488,969838,969983,972086,976550 | 2416 | - |
| MSTRG.116.1 | WBGene00016932 | C54G6.3 | --- | 995756 | 1005066 | 6 | 995756,996804,996968,997137,1003850,1004701 | 996094,996906,997094,997249,1004089,1005066 | 1288 | + |
| MSTRG.117.2 | WBGene00016930 | madf-6 | --- | 1011408 | 1014308 | 4 | 1011408,1011697,1013667,1014263 | 1011654,1011852,1014206,1014308 | 989 | - |
| MSTRG.121.1 | WBGene00004952 | spd-1 | --- | 1030629 | 1034277 | 5 | 1030629,1031995,1032928,1033265,1034037 | 1031083,1032873,1033212,1033675,1034277 | 2271 | - |
| MSTRG.123.1 | WBGene00021332 | Y34D9A.7 | --- | 1041474 | 1050727 | 5 | 1041474,1043356,1046342,1047956,1050405 | 1041690,1043925,1046578,1049686,1050727 | 3078 | + |
| MSTRG.124.3 | WBGene00021333 | Y34D9A.8 | --- | 1050924 | 1053826 | 4 | 1050924,1051916,1052569,1052794 | 1051129,1052025,1052677,1053826 | 1458 | - |
| MSTRG.136.2 | WBGene00001816 | haf-6 | --- | 1166225 | 1175846 | 7 | 1166225,1166378,1167652,1169557,1170670,1173808,1175384 | 1166325,1166583,1167976,1169800,1170896,1174095,1175846 | 1854 | - |
| MSTRG.140.2 | WBGene00195068 | Y48G8AL.16 | --- | 1215045 | 1217263 | 2 | 1215045,1217172 | 1215186,1217263 | 234 | - |
| MSTRG.142.3 | WBGene00021691 | Y48G8AL.13 | integral component of membrane | 1223959 | 1232504 | 5 | 1223959,1224655,1225176,1226620,1232392 | 1224334,1224778,1225535,1226815,1232504 | 1169 | - |
| MSTRG.142.4 | WBGene00021691 | Y48G8AL.13 | integral component of membrane | 1223959 | 1230799 | 5 | 1223959,1224655,1225176,1226620,1229246 | 1224334,1224778,1225535,1226815,1230799 | 2610 | - |
| MSTRG.149.4 | WBGene00019674 | K12C11.3 | --- | 1335395 | 1336867 | 3 | 1335395,1335996,1336688 | 1335515,1336299,1336867 | 605 | + |
| MSTRG.150.2 | WBGene00045053 | K12C11.7 | --- | 1337012 | 1339773 | 6 | 1337012,1337255,1338786,1339276,1339430,1339631 | 1337116,1337372,1338995,1339370,1339566,1339773 | 808 | + |
| MSTRG.150.3 | WBGene00045053 | K12C11.7 | --- | 1338749 | 1339703 | 3 | 1338749,1339276,1339430 | 1338995,1339370,1339703 | 616 | + |
| MSTRG.151.1 | WBGene00004888 | smo-1 | --- | 1340548 | 1341258 | 3 | 1340548,1340685,1340976 | 1340606,1340925,1341258 | 583 | + |
| MSTRG.151.2 | WBGene00004888 | smo-1 | --- | 1340678 | 1383302 | 4 | 1340678,1340825,1340976,1383285 | 1340768,1340925,1341246,1383302 | 481 | + |
| MSTRG.151.3 | WBGene00004888 | smo-1 | --- | 1340678 | 1341258 | 2 | 1340678,1340825 | 1340768,1341258 | 525 | + |
| MSTRG.155.1 | WBGene00022365 | Y92H12BL.4 | --- | 1376979 | 1379667 | 2 | 1376979,1379232 | 1377359,1379667 | 817 | - |
| MSTRG.157.1 | WBGene00022367 | Y92H12BM.1 | --- | 1385218 | 1456573 | 2 | 1385218,1456561 | 1386403,1456573 | 1199 | - |
| MSTRG.158.1 | WBGene00022367 | Y92H12BM.1 | --- | 1389222 | 1392992 | 3 | 1389222,1391113,1392581 | 1389445,1391296,1392992 | 820 | - |
| MSTRG.159.1 | WBGene00022372 | Y92H12BR.7 | --- | 1394569 | 1413100 | 10 | 1394569,1395759,1395939,1397516,1399168,1402092,1405935,1408457,1411441,1412611 | 1394780,1395874,1396112,1397707,1399347,1402911,1406372,1408636,1411615,1413100 | 2977 | + |
| MSTRG.159.2 | WBGene00022372 | Y92H12BR.7 | --- | 1394569 | 1402975 | 5 | 1394569,1395759,1397516,1399168,1402092 | 1394780,1396112,1397707,1399347,1402975 | 1822 | + |
| MSTRG.160.2 | WBGene00022368 | Y92H12BR.2 | --- | 1413925 | 1415807 | 4 | 1413925,1415238,1415400,1415553 | 1414239,1415349,1415487,1415807 | 770 | - |
| MSTRG.166.1 | WBGene00022358 | Y92H12A.2 | --- | 1451744 | 1556194 | 12 | 1451744,1543525,1543824,1544285,1544761,1545631,1550351,1551999,1552703,1553516,1554797,1555787 | 1451833,1543680,1543973,1544490,1544964,1545763,1550728,1552112,1552848,1553951,1555122,1556194 | 2747 | + |
| MSTRG.166.3 | WBGene00022360 | Y92H12A.4 | --- | 1517542 | 1556170 | 22 | 1517542,1520158,1522174,1524298,1525845,1526977,1529890,1532237,1533100,1533938,1534116,1543525,1543824,1544285,1544761,1545631,1550351,1551999,1552703,1553516,1554797,1555787 | 1518120,1520270,1522458,1524643,1526005,1527345,1530098,1532397,1533284,1534051,1534185,1543680,1543973,1544490,1544964,1545763,1550728,1552112,1552848,1553951,1555122,1556170 | 5225 | + |
| MSTRG.168.2 | WBGene00001115 | dyb-1 | --- | 1478754 | 1490494 | 12 | 1478754,1479211,1483083,1484719,1484923,1485926,1486056,1487179,1487769,1488424,1489068,1490281 | 1478996,1479469,1483235,1484876,1485059,1486011,1486251,1487410,1488095,1488675,1489281,1490494 | 2471 | + |
| MSTRG.169.1 | WBGene00022361 | Y92H12A.5 | --- | 1490505 | 1514411 | 13 | 1490505,1492386,1494680,1495718,1495902,1497064,1498798,1500688,1502689,1504477,1506875,1511224,1514204 | 1491480,1492541,1494811,1495835,1496014,1497141,1499514,1501233,1503024,1504808,1507342,1512013,1514411 | 4970 | - |
| MSTRG.173.1 | MSTRG.173 | --- | --- | 1596999 | 1598313 | 1 | 1596999 | 1598313 | 1315 | . |
| MSTRG.174.2 | WBGene00022269 | Y73E7A.2 | nucleus | 1601838 | 1606876 | 3 | 1601838,1604481,1606377 | 1602318,1605043,1606876 | 1544 | + |
| MSTRG.176.2 | WBGene00044440 | adpr-1 | --- | 1610390 | 1619943 | 7 | 1610390,1611486,1611746,1613477,1616099,1617833,1619627 | 1610900,1611594,1611827,1613895,1616260,1617968,1619943 | 1736 | + |
| MSTRG.177.1 | WBGene00000269 | bre-4 | --- | 1621346 | 1638773 | 8 | 1621346,1622400,1623182,1623775,1624775,1626344,1628413,1638643 | 1621703,1622621,1623339,1623877,1625003,1626629,1628474,1638773 | 1549 | - |
| MSTRG.177.2 | WBGene00000269 | bre-4 | --- | 1621346 | 1630590 | 8 | 1621346,1622400,1623182,1623775,1624775,1626344,1628413,1630547 | 1621703,1622621,1623339,1623877,1625003,1626629,1628474,1630590 | 1462 | - |
| MSTRG.178.1 | WBGene00022268 | Y73E7A.1 | --- | 1622474 | 1633498 | 6 | 1622474,1629005,1629163,1629282,1630553,1633221 | 1622569,1629117,1629225,1629409,1630793,1633498 | 919 | + |
| MSTRG.178.2 | WBGene00022268 | Y73E7A.1 | --- | 1628376 | 1633438 | 6 | 1628376,1629005,1629163,1629282,1630553,1633221 | 1628456,1629117,1629225,1629409,1630793,1633438 | 844 | + |
| MSTRG.182.1 | WBGene00044358 | Y71G12B.33 | --- | 1657592 | 1663331 | 5 | 1657592,1657691,1657912,1660832,1663273 | 1657643,1657868,1658170,1661040,1663331 | 757 | - |
| MSTRG.185.1 | WBGene00022158 | Y71G12B.23 | --- | 1670036 | 1676434 | 10 | 1670036,1670969,1671780,1671941,1672078,1672684,1673071,1673744,1674941,1676324 | 1670284,1671255,1671872,1672016,1672280,1672739,1673224,1673939,1675386,1676434 | 1871 | - |
| MSTRG.185.2 | WBGene00022158 | Y71G12B.23 | --- | 1670079 | 1676434 | 11 | 1670079,1670969,1671780,1671941,1672078,1672684,1673071,1673744,1674941,1675217,1676324 | 1670284,1671255,1671872,1672016,1672280,1672739,1673224,1673939,1675163,1675386,1676434 | 1775 | - |
| MSTRG.190.1 | WBGene00022153 | Y71G12B.13 | --- | 1702095 | 1708008 | 7 | 1702095,1702368,1702572,1703962,1705661,1705798,1707336 | 1702149,1702475,1702688,1704486,1705755,1706005,1708008 | 1781 | + |
| MSTRG.190.2 | WBGene00022153 | Y71G12B.13 | --- | 1702095 | 1708008 | 5 | 1702095,1702572,1703962,1705661,1707336 | 1702149,1702688,1704486,1706005,1708008 | 1715 | + |
| MSTRG.190.3 | WBGene00022153 | Y71G12B.13 | --- | 1702095 | 1708008 | 5 | 1702095,1702572,1703962,1705661,1707336 | 1702475,1702688,1704486,1706005,1708008 | 2041 | + |
| MSTRG.190.5 | WBGene00022153 | Y71G12B.13 | --- | 1702105 | 1708008 | 6 | 1702105,1702368,1702572,1703962,1705661,1707336 | 1702149,1702475,1702688,1704486,1706005,1708008 | 1813 | + |
| MSTRG.191.3 | WBGene00022152 | atg-5 | --- | 1709587 | 1715455 | 3 | 1709587,1710669,1715162 | 1709733,1712828,1715455 | 2601 | + |
| MSTRG.192.2 | WBGene00006771 | tln-1 | --- | 1721592 | 1740654 | 10 | 1721592,1721910,1725047,1726666,1728205,1730817,1732792,1734447,1738827,1740057 | 1721729,1722459,1725438,1727126,1728818,1731573,1733370,1738527,1739210,1740654 | 8554 | + |
| MSTRG.192.3 | WBGene00006771 | tln-1 | --- | 1726686 | 1740654 | 8 | 1726686,1728205,1730817,1732792,1733297,1734447,1738827,1740057 | 1727126,1728818,1731573,1733087,1733370,1738527,1739210,1740654 | 7245 | + |
| MSTRG.192.4 | WBGene00006771 | tln-1 | --- | 1726686 | 1740654 | 8 | 1726686,1728205,1730817,1733297,1734447,1738827,1740057,1740505 | 1727126,1728818,1731573,1733370,1738527,1739210,1740300,1740654 | 6745 | + |
| MSTRG.192.5 | WBGene00006771 | tln-1 | --- | 1729827 | 1740654 | 7 | 1729827,1730817,1732792,1733297,1734447,1738827,1740057 | 1730055,1731573,1733087,1733370,1738527,1739210,1740654 | 6419 | + |
| MSTRG.192.6 | WBGene00006771 | tln-1 | --- | 1729827 | 1740654 | 6 | 1729827,1730817,1732792,1734447,1738827,1740057 | 1730055,1731573,1733370,1738527,1739210,1740654 | 6628 | + |
| MSTRG.194.2 | WBGene00022150 | Y71G12B.10 | --- | 1751481 | 1754404 | 3 | 1751481,1752604,1753999 | 1751859,1752937,1754404 | 1119 | + |
| MSTRG.200.1 | WBGene00022146 | Y71G12B.6 | --- | 1793143 | 1796324 | 4 | 1793143,1793792,1794622,1796061 | 1793364,1793968,1795453,1796324 | 1495 | + |
| MSTRG.210.2 | WBGene00001196 | egl-30 | --- | 1837910 | 1841902 | 7 | 1837910,1838780,1839053,1839251,1840095,1840319,1840806 | 1838286,1838996,1839192,1839380,1840248,1840426,1841902 | 2223 | + |
| MSTRG.211.1 | WBGene00001309 | emr-1 | --- | 1842296 | 1843227 | 2 | 1842296,1842976 | 1842923,1843227 | 880 | + |
| MSTRG.212.2 | WBGene00003750 | nlp-12 | --- | 1843384 | 1843899 | 2 | 1843384,1843657 | 1843599,1843899 | 459 | + |
| MSTRG.215.2 | WBGene00022139 | tub-2 | --- | 1877635 | 1884169 | 4 | 1877635,1879504,1881929,1883335 | 1877723,1880206,1882234,1884169 | 1933 | + |
| MSTRG.217.2 | WBGene00022138 | trpp-10 | --- | 1917049 | 1922991 | 3 | 1917049,1919230,1922785 | 1918231,1919339,1922991 | 1500 | + |
| MSTRG.230.2 | WBGene00021239 | Y20F4.4 | --- | 2036959 | 2043814 | 17 | 2036959,2037397,2037581,2037770,2037974,2038095,2038401,2038571,2038743,2038888,2039041,2039164,2039553,2040539,2041365,2043579,2043755 | 2037327,2037536,2037724,2037919,2038046,2038355,2038526,2038691,2038835,2038998,2039113,2039502,2039693,2040660,2042738,2043701,2043814 | 3820 | - |
| MSTRG.230.3 | WBGene00021239 | Y20F4.4 | --- | 2036959 | 2043805 | 17 | 2036959,2037397,2037581,2037770,2037920,2038095,2038401,2038571,2038743,2038888,2039041,2039164,2039553,2040539,2041365,2043579,2043755 | 2037327,2037536,2037724,2037877,2038046,2038355,2038526,2038691,2038835,2038998,2039113,2039502,2039693,2040660,2042738,2043701,2043805 | 3823 | - |
| MSTRG.230.4 | WBGene00021239 | Y20F4.4 | --- | 2036959 | 2043805 | 16 | 2036959,2037397,2037581,2037770,2037974,2038095,2038401,2038571,2038743,2038888,2039041,2039164,2039553,2040539,2041365,2043579 | 2037327,2037536,2037724,2037919,2038046,2038355,2038526,2038691,2038835,2038998,2039113,2039502,2039693,2040660,2042738,2043805 | 3864 | - |
| MSTRG.230.5 | WBGene00021239 | Y20F4.4 | --- | 2037020 | 2043814 | 16 | 2037020,2037397,2037581,2037770,2038095,2038401,2038571,2038743,2038888,2039041,2039164,2039553,2040539,2041365,2043579,2043755 | 2037327,2037536,2037724,2038046,2038355,2038526,2038691,2038835,2038998,2039113,2039502,2039693,2040660,2042738,2043701,2043814 | 3813 | - |
| MSTRG.230.6 | WBGene00021239 | Y20F4.4 | --- | 2037023 | 2040719 | 14 | 2037023,2037397,2037581,2037770,2037920,2038095,2038401,2038571,2038743,2038888,2039041,2039160,2039553,2040539 | 2037327,2037536,2037724,2037877,2038046,2038355,2038526,2038691,2038835,2038998,2039113,2039502,2039693,2040719 | 2274 | - |
| MSTRG.230.7 | WBGene00021239 | Y20F4.4 | --- | 2037023 | 2043814 | 16 | 2037023,2037397,2037581,2037770,2037920,2038095,2038401,2038571,2038743,2038888,2039041,2039164,2039553,2041365,2043579,2043755 | 2037327,2037536,2037724,2037877,2038046,2038355,2038526,2038691,2038835,2038998,2039113,2039502,2040660,2042738,2043701,2043814 | 4613 | - |
| MSTRG.238.1 | WBGene00021350 | Y37E3.8 | --- | 2069088 | 2070607 | 4 | 2069088,2069343,2070052,2070440 | 2069293,2069837,2070207,2070607 | 1025 | - |
| MSTRG.240.1 | WBGene00021351 | eif-2alpha | --- | 2072674 | 2076933 | 4 | 2072674,2073419,2074707,2075987 | 2072806,2073966,2074955,2076933 | 1877 | + |
| MSTRG.241.1 | WBGene00021352 | pcyt-2.1 | --- | 2078748 | 2085646 | 8 | 2078748,2079264,2080744,2081100,2082814,2084339,2084479,2085251 | 2079026,2079357,2080900,2081309,2083029,2084416,2084550,2085646 | 1502 | + |
| MSTRG.246.1 | WBGene00021355 | Y37E3.17 | --- | 2128451 | 2145968 | 10 | 2128451,2134787,2135048,2136531,2138634,2141030,2142453,2144869,2145039,2145687 | 2128773,2134920,2135143,2136902,2139238,2141216,2143090,2144983,2145134,2145968 | 2848 | + |
| MSTRG.248.1 | MSTRG.248 | --- | --- | 2156838 | 2157166 | 1 | 2156838 | 2157166 | 329 | . |
| MSTRG.254.3 | WBGene00017398 | slc-17.6 | --- | 2250160 | 2254793 | 11 | 2250160,2250271,2250711,2251467,2252339,2252882,2253060,2253697,2253950,2254410,2254695 | 2250218,2250388,2250836,2251603,2252682,2253007,2253189,2253848,2254174,2254582,2254793 | 1689 | + |
| MSTRG.255.1 | WBGene00000020 | abt-2 | --- | 2255948 | 2262859 | 13 | 2255948,2257052,2257619,2258515,2258880,2259084,2259256,2260370,2261076,2261459,2261661,2262351,2262480 | 2256328,2257570,2257769,2258832,2259036,2259216,2259519,2260629,2261413,2261598,2261784,2262437,2262859 | 3252 | - |
| MSTRG.255.2 | WBGene00000020 | abt-2 | --- | 2255948 | 2262859 | 12 | 2255948,2257052,2257619,2258515,2258880,2259084,2260370,2261076,2261459,2261661,2262351,2262480 | 2256328,2257570,2257769,2258832,2259036,2259519,2260629,2261413,2261598,2261784,2262437,2262859 | 3291 | - |
| MSTRG.260.2 | WBGene00021470 | tpxl-1 | --- | 2297425 | 2304395 | 4 | 2297425,2302826,2303027,2304248 | 2297919,2302974,2304199,2304395 | 1965 | - |
| MSTRG.264.2 | WBGene00021476 | nsun-4 | --- | 2309551 | 2312085 | 2 | 2309551,2311842 | 2309658,2312085 | 352 | + |
| MSTRG.264.3 | WBGene00021476 | nsun-4 | --- | 2310852 | 2313008 | 4 | 2310852,2311099,2311842,2312750 | 2311054,2311174,2312698,2313008 | 1395 | + |
| MSTRG.266.3 | WBGene00021465 | ekl-7 | --- | 2316307 | 2318285 | 4 | 2316307,2316647,2317215,2317392 | 2316587,2317169,2317343,2318285 | 1827 | + |
| MSTRG.15061.2 | WBGene00017625 | cgt-2 | --- | 17695232 | 17701744 | 7 | 17695232,17696527,17697979,17698260,17698706,17699697,17701254 | 17695319,17696722,17698214,17698480,17699029,17699828,17701744 | 1688 | + |

| New\_transcriptid | geneid | symbol | description | transcript\_start | transcript\_end | Num\_of\_exons | exon\_sts | exon\_ens | transcript\_length | strand |
| --- | --- | --- | --- | --- | --- | --- | --- | --- | --- | --- |
| MSTRG.3.2 | WBGene00022278 | rcor-1 | --- | 17497 | 26643 | 10 | 17497,18006,19015,20271,20848,21013,24651,24929,25273,26371 | 17958,18115,19241,20478,20964,21139,24845,25090,25472,26643 | 2081 | - |
| MSTRG.3.3 | WBGene00022278 | rcor-1 | --- | 17502 | 25885 | 10 | 17502,18006,19015,20271,20848,21013,24651,24929,25273,25601 | 17958,18115,19241,20478,20964,21139,24845,25090,25472,25885 | 2088 | - |
| MSTRG.5.1 | WBGene00022279 | sesn-1 | --- | 27591 | 33146 | 6 | 27591,29100,29769,31769,32412,33037 | 28405,29367,30424,32072,32542,33146 | 2284 | - |
| MSTRG.5.2 | WBGene00022279 | sesn-1 | --- | 27591 | 34794 | 7 | 27591,29100,29769,31769,32412,33042,34693 | 28405,29367,30424,32072,32542,33144,34794 | 2379 | - |
| MSTRG.5.3 | WBGene00022279 | sesn-1 | --- | 27601 | 34745 | 7 | 27601,29100,29769,31769,32412,33042,34612 | 28405,29367,30424,32072,32542,33144,34745 | 2401 | - |
| MSTRG.7.2 | WBGene00021677 | pgs-1 | --- | 52292 | 54360 | 4 | 52292,52466,53266,53944 | 52410,52572,53695,54360 | 1073 | + |
| MSTRG.9.2 | WBGene00000812 | csk-1 | --- | 71425 | 80633 | 13 | 71425,71838,72511,72647,72941,73605,74625,75171,75951,76949,77713,79313,80306 | 71511,71932,72590,72897,73017,73766,74808,75490,76112,77153,77799,79447,80633 | 2173 | + |
| MSTRG.9.3 | WBGene00000812 | csk-1 | --- | 71617 | 80633 | 12 | 71617,72511,72647,73605,74625,75171,75951,76949,77713,78153,79313,80306 | 71932,72590,73017,73766,74808,75490,76112,77153,77799,78170,79447,80633 | 2368 | + |
| MSTRG.9.4 | WBGene00000812 | csk-1 | --- | 71726 | 80633 | 13 | 71726,72511,72647,72941,73605,74625,75171,75951,76949,77713,78153,79313,80306 | 71932,72590,72897,73017,73766,74808,75490,76112,77153,77799,78170,79447,80633 | 2216 | + |
| MSTRG.10.2 | WBGene00021683 | Y48G1C.10 | phosphatase activity | 81201 | 90957 | 16 | 81201,81879,82016,82586,83181,84190,84388,84614,84836,86824,87034,87520,88268,88566,89372,90419 | 81272,81963,82137,82676,83420,84313,84567,84738,84985,86904,87223,87734,88500,88706,89584,90957 | 2801 | + |
| … | … | … | … | … | … | … | … | … | … | … |

| New\_transcriptid | geneid | symbol | description | transcript\_start | transcript\_end | Num\_of\_exons | exon\_sts | exon\_ens | transcript\_length | strand |
| --- | --- | --- | --- | --- | --- | --- | --- | --- | --- | --- |
| MSTRG.3.2 | WBGene00022278 | rcor-1 | --- | 17497 | 26643 | 10 | 17497,18006,19015,20271,20848,21013,24651,24929,25273,26371 | 17958,18115,19241,20478,20964,21139,24845,25090,25472,26643 | 2081 | - |
| MSTRG.3.3 | WBGene00022278 | rcor-1 | --- | 17502 | 25885 | 10 | 17502,18006,19015,20271,20848,21013,24651,24929,25273,25601 | 17958,18115,19241,20478,20964,21139,24845,25090,25472,25885 | 2088 | - |
| MSTRG.5.1 | WBGene00022279 | sesn-1 | --- | 27591 | 33146 | 6 | 27591,29100,29769,31769,32412,33037 | 28405,29367,30424,32072,32542,33146 | 2284 | - |
| MSTRG.5.2 | WBGene00022279 | sesn-1 | --- | 27591 | 34794 | 7 | 27591,29100,29769,31769,32412,33042,34693 | 28405,29367,30424,32072,32542,33144,34794 | 2379 | - |
| MSTRG.5.3 | WBGene00022279 | sesn-1 | --- | 27601 | 34745 | 7 | 27601,29100,29769,31769,32412,33042,34612 | 28405,29367,30424,32072,32542,33144,34745 | 2401 | - |
| MSTRG.7.2 | WBGene00021677 | pgs-1 | --- | 52292 | 54360 | 4 | 52292,52466,53266,53944 | 52410,52572,53695,54360 | 1073 | + |
| MSTRG.9.2 | WBGene00000812 | csk-1 | --- | 71425 | 80633 | 13 | 71425,71838,72511,72647,72941,73605,74625,75171,75951,76949,77713,79313,80306 | 71511,71932,72590,72897,73017,73766,74808,75490,76112,77153,77799,79447,80633 | 2173 | + |
| MSTRG.9.3 | WBGene00000812 | csk-1 | --- | 71617 | 80633 | 12 | 71617,72511,72647,73605,74625,75171,75951,76949,77713,78153,79313,80306 | 71932,72590,73017,73766,74808,75490,76112,77153,77799,78170,79447,80633 | 2368 | + |
| MSTRG.9.4 | WBGene00000812 | csk-1 | --- | 71726 | 80633 | 13 | 71726,72511,72647,72941,73605,74625,75171,75951,76949,77713,78153,79313,80306 | 71932,72590,72897,73017,73766,74808,75490,76112,77153,77799,78170,79447,80633 | 2216 | + |
| MSTRG.10.2 | WBGene00021683 | Y48G1C.10 | phosphatase activity | 81201 | 90957 | 16 | 81201,81879,82016,82586,83181,84190,84388,84614,84836,86824,87034,87520,88268,88566,89372,90419 | 81272,81963,82137,82676,83420,84313,84567,84738,84985,86904,87223,87734,88500,88706,89584,90957 | 2801 | + |
| … | … | … | … | … | … | … | … | … | … | … |

… … … … … … … … … … …

  
  

| 表头 | 表头说明 |
| --- | --- |
| New\_transcriptid | 新转录本编号 |
| geneid | 新转录本对应的已知基因或者新基因 |
| symbol | 已知基因简称 |
| transcript\_start | 转录本开始位置 |
| transcript\_end | 转录本结束位置 |
| Num\_of\_exons | 转录本的外显子个数 |
| exon\_sts | 各个外显子的开始位置 |
| exon\_ens | 各个外显子的结束位置 |
| transcript\_length | 转录本的长度 |
| strand | 转录本的链方向 |

  

### 已知基因结构模型优化结果格式说明(gene\_model\_modification.xls)

显示 10203050100150全文 行  
ASSEMBLY-merge
 

| Gene\_id | Chr | Strand | Gene\_start\_knownGTF | Gene\_end\_knownGTF | Gene\_start\_assemblyGTF | Gene\_end\_assemblyGTF |
| --- | --- | --- | --- | --- | --- | --- |
| WBGene00172216 | IV | - | 5596056 | 5596076 | --- | --- |
| WBGene00166897 | IV | + | 14422328 | 14422348 | --- | --- |
| WBGene00197957 | I | + | 9942323 | 9942758 | --- | --- |
| WBGene00048996 | IV | + | 16727358 | 16727378 | --- | --- |
| WBGene00014308 | II | + | 8786457 | 8787119 | --- | --- |
| WBGene00011842 | V | + | 17229555 | 17229990 | --- | --- |
| WBGene00165353 | IV | - | 16986283 | 16986303 | --- | --- |
| WBGene00013247 | II | + | 12147633 | 12150216 | 12147633 | 12150216 |
| WBGene00173191 | IV | - | 14407460 | 14407480 | --- | --- |
| WBGene00172369 | IV | - | 14046293 | 14046313 | --- | --- |
| WBGene00200336 | II | + | 10109634 | 10109738 | --- | --- |
| WBGene00173090 | IV | + | 13724677 | 13724697 | --- | --- |
| WBGene00049221 | IV | - | 15492703 | 15492723 | --- | --- |
| WBGene00198967 | V | - | 18836153 | 18836218 | --- | --- |
| WBGene00049695 | IV | - | 15111869 | 15111889 | --- | --- |
| WBGene00047108 | IV | + | 13726636 | 13726656 | --- | --- |
| WBGene00001668 | X | + | 13191246 | 13193955 | --- | --- |
| WBGene00007808 | IV | - | 11226502 | 11230307 | --- | --- |
| WBGene00197791 | II | + | 8569431 | 8569572 | --- | --- |
| WBGene00044964 | I | - | 7091584 | 7091718 | --- | --- |
| WBGene00007747 | V | + | 13297520 | 13298050 | --- | --- |
| WBGene00220034 | IV | + | 3521958 | 3522027 | --- | --- |
| WBGene00171592 | IV | + | 15605264 | 15605284 | --- | --- |
| WBGene00050743 | IV | + | 15123054 | 15123074 | --- | --- |
| WBGene00173038 | IV | - | 17226437 | 17226457 | --- | --- |
| WBGene00015062 | II | - | 7752637 | 7753607 | --- | --- |
| WBGene00171107 | IV | + | 15804457 | 15804477 | --- | --- |
| WBGene00009527 | X | + | 12661164 | 12661247 | --- | --- |
| WBGene00044463 | IV | - | 1219333 | 1221287 | --- | --- |
| WBGene00170729 | IV | + | 15750154 | 15750174 | --- | --- |
| WBGene00013566 | V | + | 15109577 | 15110345 | --- | --- |
| WBGene00020227 | II | - | 2708438 | 2710096 | --- | --- |
| WBGene00016940 | X | - | 7187355 | 7188482 | --- | --- |
| WBGene00174831 | IV | + | 15490472 | 15490492 | --- | --- |
| WBGene00171019 | IV | - | 5009510 | 5009530 | --- | --- |
| WBGene00001447 | II | + | 10054623 | 10055594 | 10048891 | 10055594 |
| WBGene00021357 | IV | + | 3709515 | 3715381 | --- | --- |
| WBGene00174232 | IV | + | 14323333 | 14323353 | --- | --- |
| WBGene00048906 | IV | - | 15261027 | 15261047 | --- | --- |
| WBGene00172473 | IV | + | 16442194 | 16442214 | --- | --- |
| WBGene00023351 | V | + | 3731808 | 3732837 | --- | --- |
| WBGene00169240 | IV | + | 13893996 | 13894016 | --- | --- |
| WBGene00172974 | IV | + | 5281768 | 5281788 | --- | --- |
| WBGene00167264 | IV | + | 15794793 | 15794813 | --- | --- |
| WBGene00166807 | IV | - | 14273696 | 14273716 | --- | --- |
| WBGene00199417 | I | - | 2835345 | 2835482 | --- | --- |
| WBGene00050374 | IV | - | 15661904 | 15661924 | --- | --- |
| WBGene00014563 | IV | - | 14013805 | 14013876 | --- | --- |
| WBGene00199320 | V | - | 14505853 | 14505975 | --- | --- |
| WBGene00007898 | IV | - | 8790742 | 8793294 | --- | --- |
| WBGene00000883 | V | - | 15186582 | 15187787 | --- | --- |
| WBGene00168480 | IV | - | 15839561 | 15839581 | --- | --- |
| WBGene00016810 | II | - | 92169 | 95277 | --- | --- |
| WBGene00047198 | IV | - | 16096595 | 16096615 | --- | --- |
| WBGene00012035 | II | - | 9245905 | 9246811 | --- | --- |
| WBGene00201460 | II | - | 241599 | 241651 | --- | --- |
| WBGene00011186 | V | + | 13946969 | 13948799 | --- | --- |
| WBGene00196021 | V | + | 7175054 | 7175254 | --- | --- |
| WBGene00043466 | IV | + | 7516585 | 7517035 | --- | --- |
| WBGene00049605 | IV | + | 15232380 | 15232400 | --- | --- |
| WBGene00197939 | IV | - | 7275874 | 7276076 | --- | --- |
| WBGene00008434 | II | - | 10663605 | 10665131 | --- | --- |
| WBGene00196120 | X | + | 10128229 | 10128631 | --- | --- |
| WBGene00173000 | III | - | 13480497 | 13480517 | --- | --- |
| WBGene00173101 | IV | + | 16453330 | 16453350 | --- | --- |
| WBGene00045275 | V | + | 7414941 | 7415344 | --- | --- |
| WBGene00045663 | IV | - | 15877277 | 15877297 | --- | --- |
| WBGene00168254 | IV | + | 15348392 | 15348412 | --- | --- |
| WBGene00008933 | II | + | 13043951 | 13045975 | --- | --- |
| WBGene00014398 | X | + | 13781640 | 13781712 | --- | --- |
| WBGene00047130 | IV | - | 14483678 | 14483698 | --- | --- |
| WBGene00049822 | IV | + | 14370650 | 14370670 | --- | --- |
| WBGene00046929 | IV | - | 16605152 | 16605172 | --- | --- |
| WBGene00047031 | IV | - | 15248478 | 15248498 | --- | --- |
| WBGene00009122 | I | + | 10569112 | 10570030 | 10569112 | 10570030 |
| WBGene00167884 | IV | + | 15211553 | 15211573 | --- | --- |
| WBGene00004972 | IV | - | 10389578 | 10391969 | --- | --- |
| WBGene00021339 | III | - | 1023221 | 1024561 | 1023221 | 1026411 |
| WBGene00171197 | IV | - | 14672729 | 14672749 | --- | --- |
| WBGene00011241 | IV | - | 10372638 | 10373821 | --- | --- |
| WBGene00014330 | V | - | 15013034 | 15013106 | --- | --- |
| WBGene00171502 | IV | + | 15254159 | 15254179 | --- | --- |
| WBGene00197701 | II | - | 11753268 | 11753479 | --- | --- |
| WBGene00002234 | X | - | 8243131 | 8246369 | --- | --- |
| WBGene00169086 | IV | - | 13711458 | 13711478 | --- | --- |
| WBGene00015081 | III | + | 5738008 | 5740471 | --- | --- |
| WBGene00015180 | X | + | 9278953 | 9279947 | --- | --- |
| WBGene00201854 | V | + | 6408902 | 6409045 | --- | --- |
| WBGene00017179 | II | + | 3278987 | 3281686 | --- | --- |
| WBGene00165089 | IV | - | 6028277 | 6028297 | --- | --- |
| WBGene00011912 | I | + | 7936223 | 7938328 | --- | --- |
| WBGene00167070 | IV | - | 16987903 | 16987923 | --- | --- |
| WBGene00166748 | IV | - | 14922154 | 14922174 | --- | --- |
| WBGene00167171 | IV | + | 14878914 | 14878934 | --- | --- |
| WBGene00165628 | IV | - | 16115790 | 16115810 | --- | --- |
| WBGene00023318 | II | - | 4048525 | 4049129 | 4048525 | 4049752 |
| WBGene00202027 | I | + | 14831271 | 14831388 | --- | --- |
| WBGene00048745 | IV | + | 16732426 | 16732446 | --- | --- |
| WBGene00000675 | IV | - | 325437 | 326711 | --- | --- |
| WBGene00000263 | III | - | 909957 | 910589 | 909957 | 910883 |
| WBGene00010660 | IV | - | 12887102 | 12890508 | --- | --- |
| WBGene00044379 | II | - | 3687791 | 3688691 | --- | --- |
| WBGene00000952 | X | + | 14268081 | 14277040 | --- | --- |
| WBGene00168725 | IV | - | 16571058 | 16571078 | --- | --- |
| WBGene00013732 | III | - | 12538664 | 12575401 | --- | --- |
| WBGene00010682 | II | + | 8767185 | 8769307 | --- | --- |
| WBGene00045648 | IV | - | 14976698 | 14976718 | --- | --- |
| WBGene00016212 | II | - | 6290652 | 6292524 | 6290652 | 6294356 |
| WBGene00173138 | IV | + | 16994763 | 16994783 | --- | --- |
| WBGene00046728 | IV | + | 16635235 | 16635255 | --- | --- |
| WBGene00015162 | III | - | 7287848 | 7290562 | --- | --- |
| WBGene00171007 | IV | - | 17198828 | 17198848 | --- | --- |
| WBGene00220134 | II | - | 12041957 | 12042146 | --- | --- |
| WBGene00197790 | X | - | 10163932 | 10164011 | --- | --- |
| WBGene00022945 | II | - | 5057447 | 5057520 | --- | --- |
| WBGene00174830 | IV | - | 15570339 | 15570359 | --- | --- |
| WBGene00014548 | V | - | 16645706 | 16645777 | --- | --- |
| WBGene00171119 | IV | + | 5119322 | 5119342 | --- | --- |
| WBGene00022588 | V | + | 5460106 | 5461438 | --- | --- |
| WBGene00016941 | X | - | 7204742 | 7205295 | --- | --- |
| WBGene00169842 | IV | + | 5573644 | 5573664 | --- | --- |
| WBGene00048589 | IV | + | 14321132 | 14321152 | --- | --- |
| WBGene00166949 | IV | + | 15171422 | 15171442 | --- | --- |
| WBGene00000248 | III | - | 3537688 | 3541628 | --- | --- |
| WBGene00195375 | X | - | 12909889 | 12910028 | --- | --- |
| WBGene00174898 | IV | + | 16440130 | 16440150 | --- | --- |
| WBGene00168462 | IV | + | 15302244 | 15302264 | --- | --- |
| WBGene00201482 | V | - | 11463141 | 11463277 | --- | --- |
| WBGene00022706 | IV | + | 5310042 | 5311445 | --- | --- |
| WBGene00021428 | IV | - | 2396257 | 2399420 | --- | --- |
| WBGene00166763 | IV | + | 16041084 | 16041104 | --- | --- |
| WBGene00047008 | IV | + | 14190706 | 14190726 | --- | --- |
| WBGene00199613 | III | + | 8287782 | 8287922 | --- | --- |
| WBGene00195496 | X | + | 13654544 | 13654669 | --- | --- |
| WBGene00049220 | IV | - | 15757139 | 15757159 | --- | --- |
| WBGene00007232 | V | - | 15089077 | 15089538 | --- | --- |
| WBGene00199499 | V | - | 13689501 | 13689725 | --- | --- |
| WBGene00173091 | IV | + | 16083588 | 16083608 | --- | --- |
| WBGene00012713 | III | - | 12967898 | 12972498 | --- | --- |
| WBGene00173190 | IV | + | 6386756 | 6386776 | --- | --- |
| WBGene00172677 | IV | + | 15807607 | 15807627 | --- | --- |
| WBGene00197738 | I | + | 8628595 | 8628744 | --- | --- |
| WBGene00167452 | IV | + | 17077165 | 17077185 | --- | --- |
| WBGene00015567 | II | + | 7394765 | 7397351 | --- | --- |
| WBGene00197700 | IV | + | 13435784 | 13435891 | --- | --- |
| WBGene00012289 | I | + | 13418458 | 13423379 | --- | --- |
| WBGene00014331 | V | - | 13003509 | 13003580 | --- | --- |
| WBGene00166386 | IV | + | 14386197 | 14386217 | --- | --- |
| WBGene00016858 | V | - | 3069898 | 3071648 | --- | --- |
| WBGene00171097 | IV | + | 15302716 | 15302736 | --- | --- |
| WBGene00011240 | IV | + | 10374905 | 10387318 | 10374905 | 10387318 |
| WBGene00001643 | I | + | 7110240 | 7112287 | --- | --- |
| WBGene00170928 | X | + | 13183502 | 13183522 | --- | --- |
| WBGene00194696 | II | - | 5412196 | 5412610 | --- | --- |
| WBGene00018776 | IV | - | 1297222 | 1309652 | --- | --- |
| WBGene00047030 | IV | - | 14092272 | 14092292 | --- | --- |
| WBGene00007831 | V | + | 16297370 | 16298278 | --- | --- |
| WBGene00015874 | II | - | 3837338 | 3838948 | --- | --- |
| WBGene00047131 | IV | + | 16137118 | 16137138 | --- | --- |
| WBGene00271799 | V | - | 8845380 | 8845768 | --- | --- |

| Gene\_id | Chr | Strand | Gene\_start\_knownGTF | Gene\_end\_knownGTF | Gene\_start\_assemblyGTF | Gene\_end\_assemblyGTF |
| --- | --- | --- | --- | --- | --- | --- |
| WBGene00172216 | IV | - | 5596056 | 5596076 | --- | --- |
| WBGene00166897 | IV | + | 14422328 | 14422348 | --- | --- |
| WBGene00197957 | I | + | 9942323 | 9942758 | --- | --- |
| WBGene00048996 | IV | + | 16727358 | 16727378 | --- | --- |
| WBGene00014308 | II | + | 8786457 | 8787119 | --- | --- |
| WBGene00011842 | V | + | 17229555 | 17229990 | --- | --- |
| WBGene00165353 | IV | - | 16986283 | 16986303 | --- | --- |
| WBGene00013247 | II | + | 12147633 | 12150216 | 12147633 | 12150216 |
| WBGene00173191 | IV | - | 14407460 | 14407480 | --- | --- |
| WBGene00172369 | IV | - | 14046293 | 14046313 | --- | --- |
| … | … | … | … | … | … | … |

| Gene\_id | Chr | Strand | Gene\_start\_knownGTF | Gene\_end\_knownGTF | Gene\_start\_assemblyGTF | Gene\_end\_assemblyGTF |
| --- | --- | --- | --- | --- | --- | --- |
| WBGene00172216 | IV | - | 5596056 | 5596076 | --- | --- |
| WBGene00166897 | IV | + | 14422328 | 14422348 | --- | --- |
| WBGene00197957 | I | + | 9942323 | 9942758 | --- | --- |
| WBGene00048996 | IV | + | 16727358 | 16727378 | --- | --- |
| WBGene00014308 | II | + | 8786457 | 8787119 | --- | --- |
| WBGene00011842 | V | + | 17229555 | 17229990 | --- | --- |
| WBGene00165353 | IV | - | 16986283 | 16986303 | --- | --- |
| WBGene00013247 | II | + | 12147633 | 12150216 | 12147633 | 12150216 |
| WBGene00173191 | IV | - | 14407460 | 14407480 | --- | --- |
| WBGene00172369 | IV | - | 14046293 | 14046313 | --- | --- |
| … | … | … | … | … | … | … |

… … … … … … …

  
  

| 表头 | 表头说明 |
| --- | --- |
| Gene\_ID | 基因编号 |
| Chromosome | 染色体编号 |
| Strand | 链方向 |
| Gene\_start\_knownGTF | 基因在已知GTF的开始位置 |
| Gene\_end\_knownGTF | 基因在已知GTF的结束位置 |
| Gene\_start\_assemblyGTF | 基因在组装GTF的开始位置 |
| Gene\_end\_assemblyGTF | 基因在组装GTF的结束位置 |

  

### GTF格式说明(\*.gtf)

| 列号 | 说明 |
| --- | --- |
| 第1列 | 染色体编号 |
| 第2列 | 转录本注释来源cufflinks组装 |
| 第3列 | 注释类型(如transcript、exon) |
| 第4列 | 开始位置 |
| 第5列 | 结束位置 |
| 第6列 | 得分和数字，是注释信息可能性的说明, ”.”表示为空 |
| 第7列 | 序列的方向， +代表正链， -代表负链， .代表未知 |
| 第8列 | 仅对注释类型为CDS有效，表示起始编码的位置，有效值为0、1、2 |
| 第9列 | 注释信息，包括基因编号，转录本编号，表达量等 |

  
  


## 4.3 编码蛋白质的新转录本预测

目录链接

  
  
  
  

针对StringTie组装的基因间隔预测的新转录本正链和反向互补链，利用Transdecoder程序基于马尔科夫模型(log似然比，基于可编码与非编码的log似然比)原理预测转录本潜在蛋白编码区域(CDS)， 根据标准密码子表将编码区序列翻译成氨基酸序列，得到转录本中潜在编码的蛋白质序列，针对转录本潜在多个蛋白质序列，采取最长序列为新转录本的预测蛋白质序列。 利用Blastp程序与Uniprot-Swissprot蛋白质序列数据库分别进行比对， Evalue设置为1E-3。 比对结果中Uniprot-Swissprot蛋白质靶标序列的GO注释信息对新转录本的编码蛋白质序列进行GO功能分类注释。 采用KEGG数据库对新转录本的编码蛋白质序列进行KEGG Pathway注释。 采用Interpro数据库对新转录本的编码蛋白质序列进行蛋白质结构功能域预测。

编码蛋白质新转录本的所有靶标序列与Uniprot-Swissprot蛋白质序列数据库Blastp比对产生最佳对应关系，对编码蛋白质新转录本进行GO功能/KEGG代谢途径/Interpro功能域等注释。

### Uniprot-Swissprot蛋白质序列比对结果说明(protein\_blastp2swissprot\_bestone.xls)

显示 10全文 行  
ASSEMBLY-merge
 

| Query\_Seq\_id | Query\_sequence\_length | Subject\_type | Subject\_id | Subject\_name | Subject\_sequence\_length | Query\_start | Query\_end | Subject\_start | Subject\_end | Alignment\_length | Number\_of\_identical\_matches | Evalue |
| --- | --- | --- | --- | --- | --- | --- | --- | --- | --- | --- | --- | --- |
| MSTRG.15060//MSTRG.15060.1:562-3237(+) | 891 | sp | Q10126 | YSM6\_CAEEL | 279 | 617 | 891 | 1 | 279 | 279 | 261 | 1.85e-175 |
| MSTRG.1577//MSTRG.1577.1:455-2548(+) | 697 | sp | Q09575 | YRD6\_CAEEL | 1268 | 1 | 399 | 1 | 420 | 421 | 206 | 1.95e-128 |
| MSTRG.6036//MSTRG.6036.1:1969-2400(+) | 143 | sp | Q09234 | YQ41\_CAEEL | 143 | 1 | 143 | 1 | 143 | 143 | 143 | 1.19e-102 |
| MSTRG.6838//MSTRG.6838.1:303-1271(+) | 322 | sp | Q9NBX4 | RTXE\_DROME | 908 | 131 | 314 | 475 | 658 | 190 | 62 | 1.79e-20 |
| MSTRG.8179//MSTRG.8179.1:2-412(+) | 136 | sp | P34457 | YMD3\_CAEEL | 286 | 27 | 136 | 188 | 286 | 110 | 84 | 6.66e-35 |

| Query\_Seq\_id | Query\_sequence\_length | Subject\_type | Subject\_id | Subject\_name | Subject\_sequence\_length | Query\_start | Query\_end | Subject\_start | Subject\_end | Alignment\_length | Number\_of\_identical\_matches | Evalue |
| --- | --- | --- | --- | --- | --- | --- | --- | --- | --- | --- | --- | --- |
| MSTRG.15060//MSTRG.15060.1:562-3237(+) | 891 | sp | Q10126 | YSM6\_CAEEL | 279 | 617 | 891 | 1 | 279 | 279 | 261 | 1.85e-175 |
| MSTRG.1577//MSTRG.1577.1:455-2548(+) | 697 | sp | Q09575 | YRD6\_CAEEL | 1268 | 1 | 399 | 1 | 420 | 421 | 206 | 1.95e-128 |
| MSTRG.6036//MSTRG.6036.1:1969-2400(+) | 143 | sp | Q09234 | YQ41\_CAEEL | 143 | 1 | 143 | 1 | 143 | 143 | 143 | 1.19e-102 |
| MSTRG.6838//MSTRG.6838.1:303-1271(+) | 322 | sp | Q9NBX4 | RTXE\_DROME | 908 | 131 | 314 | 475 | 658 | 190 | 62 | 1.79e-20 |
| MSTRG.8179//MSTRG.8179.1:2-412(+) | 136 | sp | P34457 | YMD3\_CAEEL | 286 | 27 | 136 | 188 | 286 | 110 | 84 | 6.66e-35 |

| Query\_Seq\_id | Query\_sequence\_length | Subject\_type | Subject\_id | Subject\_name | Subject\_sequence\_length | Query\_start | Query\_end | Subject\_start | Subject\_end | Alignment\_length | Number\_of\_identical\_matches | Evalue |
| --- | --- | --- | --- | --- | --- | --- | --- | --- | --- | --- | --- | --- |
| MSTRG.15060//MSTRG.15060.1:562-3237(+) | 891 | sp | Q10126 | YSM6\_CAEEL | 279 | 617 | 891 | 1 | 279 | 279 | 261 | 1.85e-175 |
| MSTRG.1577//MSTRG.1577.1:455-2548(+) | 697 | sp | Q09575 | YRD6\_CAEEL | 1268 | 1 | 399 | 1 | 420 | 421 | 206 | 1.95e-128 |
| MSTRG.6036//MSTRG.6036.1:1969-2400(+) | 143 | sp | Q09234 | YQ41\_CAEEL | 143 | 1 | 143 | 1 | 143 | 143 | 143 | 1.19e-102 |
| MSTRG.6838//MSTRG.6838.1:303-1271(+) | 322 | sp | Q9NBX4 | RTXE\_DROME | 908 | 131 | 314 | 475 | 658 | 190 | 62 | 1.79e-20 |
| MSTRG.8179//MSTRG.8179.1:2-412(+) | 136 | sp | P34457 | YMD3\_CAEEL | 286 | 27 | 136 | 188 | 286 | 110 | 84 | 6.66e-35 |

  
  

| 表头 | 表头说明 |
| --- | --- |
| Query\_Seq\_ID | 查询序列ID |
| Query\_sequence\_length | 查询序列长度 |
| Subject\_type | Uniprot-Swissprot蛋白质数据库类型 |
| Subject\_ID | Uniprot-Swissprot蛋白质靶标序列ID |
| Subject\_name | 查询序列名称 |
| Subject\_sequence\_length | 查询序列长度 |
| Query\_start | 查询序列比对的开始位置 |
| Query\_end | 查询序列比对的结束位置 |
| Subject\_start | 靶标序列比对的开始位置 |
| Subject\_end | 靶标序列比对的结束位置 |
| Alignment\_length | 比对的长度 |
| Number\_of\_identical\_matches | 比对上的碱基对总数 |
| Evalue | 比对的显著性指标 |

  

### 编码蛋白质新转录本的GO功能注释(novel\_protein2GO.xls)

显示 10203050100150全文 行  
ASSEMBLY-merge
 

| Seqid | GOid | GOname | GOcategory | GOlevel |
| --- | --- | --- | --- | --- |
| MSTRG.15060//MSTRG.15060.1:562-3237(+) | GO:0004520 | endodeoxyribonuclease activity | molecular\_function | 9 |
| MSTRG.1577//MSTRG.1577.1:455-2548(+) | GO:0046872 | metal ion binding | molecular\_function | 4 |
| MSTRG.15060//MSTRG.15060.1:562-3237(+) | GO:0046872 | metal ion binding | molecular\_function | 4 |
| MSTRG.15060//MSTRG.15060.1:562-3237(+) | GO:0009056 | catabolic process | biological\_process | 2 |
| MSTRG.15060//MSTRG.15060.1:562-3237(+) | GO:0044265 | cellular macromolecule catabolic process | biological\_process | 5 |
| MSTRG.6838//MSTRG.6838.1:303-1271(+) | GO:0016772 | transferase activity, transferring phosphorus-containing groups | molecular\_function | 3 |
| MSTRG.15060//MSTRG.15060.1:562-3237(+) | GO:0016772 | transferase activity, transferring phosphorus-containing groups | molecular\_function | 3 |
| MSTRG.15060//MSTRG.15060.1:562-3237(+) | GO:0001171 | reverse transcription | biological\_process | 9 |
| MSTRG.1577//MSTRG.1577.1:455-2548(+) | GO:0043167 | ion binding | molecular\_function | 2 |
| MSTRG.15060//MSTRG.15060.1:562-3237(+) | GO:0043167 | ion binding | molecular\_function | 2 |
| MSTRG.15060//MSTRG.15060.1:562-3237(+) | GO:0046483 | heterocycle metabolic process | biological\_process | 3 |
| MSTRG.6838//MSTRG.6838.1:303-1271(+) | GO:0046483 | heterocycle metabolic process | biological\_process | 3 |
| MSTRG.8179//MSTRG.8179.1:2-412(+) | GO:0046483 | heterocycle metabolic process | biological\_process | 3 |
| MSTRG.1577//MSTRG.1577.1:455-2548(+) | GO:0046483 | heterocycle metabolic process | biological\_process | 3 |
| MSTRG.6838//MSTRG.6838.1:303-1271(+) | GO:0044249 | cellular biosynthetic process | biological\_process | 3 |
| MSTRG.15060//MSTRG.15060.1:562-3237(+) | GO:0044249 | cellular biosynthetic process | biological\_process | 3 |
| MSTRG.15060//MSTRG.15060.1:562-3237(+) | GO:1901361 | organic cyclic compound catabolic process | biological\_process | 4 |
| MSTRG.15060//MSTRG.15060.1:562-3237(+) | GO:0006310 | DNA recombination | biological\_process | 7 |
| MSTRG.6838//MSTRG.6838.1:303-1271(+) | GO:0006310 | DNA recombination | biological\_process | 7 |
| MSTRG.15060//MSTRG.15060.1:562-3237(+) | GO:0044260 | cellular macromolecule metabolic process | biological\_process | 4 |
| MSTRG.6838//MSTRG.6838.1:303-1271(+) | GO:0044260 | cellular macromolecule metabolic process | biological\_process | 4 |
| MSTRG.8179//MSTRG.8179.1:2-412(+) | GO:0044260 | cellular macromolecule metabolic process | biological\_process | 4 |
| MSTRG.1577//MSTRG.1577.1:455-2548(+) | GO:0044260 | cellular macromolecule metabolic process | biological\_process | 4 |
| MSTRG.15060//MSTRG.15060.1:562-3237(+) | GO:0006725 | cellular aromatic compound metabolic process | biological\_process | 3 |
| MSTRG.6838//MSTRG.6838.1:303-1271(+) | GO:0006725 | cellular aromatic compound metabolic process | biological\_process | 3 |
| MSTRG.8179//MSTRG.8179.1:2-412(+) | GO:0006725 | cellular aromatic compound metabolic process | biological\_process | 3 |
| MSTRG.1577//MSTRG.1577.1:455-2548(+) | GO:0006725 | cellular aromatic compound metabolic process | biological\_process | 3 |
| MSTRG.6838//MSTRG.6838.1:303-1271(+) | GO:0044271 | cellular nitrogen compound biosynthetic process | biological\_process | 4 |
| MSTRG.15060//MSTRG.15060.1:562-3237(+) | GO:0044271 | cellular nitrogen compound biosynthetic process | biological\_process | 4 |
| MSTRG.15060//MSTRG.15060.1:562-3237(+) | GO:0009987 | cellular process | biological\_process | 1 |
| MSTRG.6838//MSTRG.6838.1:303-1271(+) | GO:0009987 | cellular process | biological\_process | 1 |
| MSTRG.8179//MSTRG.8179.1:2-412(+) | GO:0009987 | cellular process | biological\_process | 1 |
| MSTRG.1577//MSTRG.1577.1:455-2548(+) | GO:0009987 | cellular process | biological\_process | 1 |
| MSTRG.15060//MSTRG.15060.1:562-3237(+) | GO:0046700 | heterocycle catabolic process | biological\_process | 4 |
| MSTRG.6838//MSTRG.6838.1:303-1271(+) | GO:0016740 | transferase activity | molecular\_function | 2 |
| MSTRG.15060//MSTRG.15060.1:562-3237(+) | GO:0016740 | transferase activity | molecular\_function | 2 |
| MSTRG.15060//MSTRG.15060.1:562-3237(+) | GO:0016788 | hydrolase activity, acting on ester bonds | molecular\_function | 3 |
| MSTRG.6838//MSTRG.6838.1:303-1271(+) | GO:0016788 | hydrolase activity, acting on ester bonds | molecular\_function | 3 |
| MSTRG.6838//MSTRG.6838.1:303-1271(+) | GO:0006313 | transposition, DNA-mediated | biological\_process | 8 |
| MSTRG.15060//MSTRG.15060.1:562-3237(+) | GO:0015666 | restriction endodeoxyribonuclease activity | molecular\_function | 10 |
| MSTRG.15060//MSTRG.15060.1:562-3237(+) | GO:0071704 | organic substance metabolic process | biological\_process | 2 |
| MSTRG.6838//MSTRG.6838.1:303-1271(+) | GO:0071704 | organic substance metabolic process | biological\_process | 2 |
| MSTRG.8179//MSTRG.8179.1:2-412(+) | GO:0071704 | organic substance metabolic process | biological\_process | 2 |
| MSTRG.1577//MSTRG.1577.1:455-2548(+) | GO:0071704 | organic substance metabolic process | biological\_process | 2 |
| MSTRG.1577//MSTRG.1577.1:455-2548(+) | GO:0043169 | cation binding | molecular\_function | 3 |
| MSTRG.15060//MSTRG.15060.1:562-3237(+) | GO:0043169 | cation binding | molecular\_function | 3 |
| MSTRG.8179//MSTRG.8179.1:2-412(+) | GO:0097159 | organic cyclic compound binding | molecular\_function | 2 |
| MSTRG.1577//MSTRG.1577.1:455-2548(+) | GO:0097159 | organic cyclic compound binding | molecular\_function | 2 |
| MSTRG.6838//MSTRG.6838.1:303-1271(+) | GO:0034645 | cellular macromolecule biosynthetic process | biological\_process | 5 |
| MSTRG.15060//MSTRG.15060.1:562-3237(+) | GO:0034645 | cellular macromolecule biosynthetic process | biological\_process | 5 |
| MSTRG.15060//MSTRG.15060.1:562-3237(+) | GO:0006259 | DNA metabolic process | biological\_process | 6 |
| MSTRG.6838//MSTRG.6838.1:303-1271(+) | GO:0006259 | DNA metabolic process | biological\_process | 6 |
| MSTRG.8179//MSTRG.8179.1:2-412(+) | GO:0006259 | DNA metabolic process | biological\_process | 6 |
| MSTRG.1577//MSTRG.1577.1:455-2548(+) | GO:0006259 | DNA metabolic process | biological\_process | 6 |
| MSTRG.15060//MSTRG.15060.1:562-3237(+) | GO:0090304 | nucleic acid metabolic process | biological\_process | 5 |
| MSTRG.6838//MSTRG.6838.1:303-1271(+) | GO:0090304 | nucleic acid metabolic process | biological\_process | 5 |
| MSTRG.8179//MSTRG.8179.1:2-412(+) | GO:0090304 | nucleic acid metabolic process | biological\_process | 5 |
| MSTRG.1577//MSTRG.1577.1:455-2548(+) | GO:0090304 | nucleic acid metabolic process | biological\_process | 5 |
| MSTRG.15060//MSTRG.15060.1:562-3237(+) | GO:0003824 | catalytic activity | molecular\_function | 1 |
| MSTRG.6838//MSTRG.6838.1:303-1271(+) | GO:0003824 | catalytic activity | molecular\_function | 1 |
| MSTRG.15060//MSTRG.15060.1:562-3237(+) | GO:0032196 | transposition | biological\_process | 3 |
| MSTRG.6838//MSTRG.6838.1:303-1271(+) | GO:0032196 | transposition | biological\_process | 3 |
| MSTRG.6838//MSTRG.6838.1:303-1271(+) | GO:0009059 | macromolecule biosynthetic process | biological\_process | 4 |
| MSTRG.15060//MSTRG.15060.1:562-3237(+) | GO:0009059 | macromolecule biosynthetic process | biological\_process | 4 |
| MSTRG.15060//MSTRG.15060.1:562-3237(+) | GO:0019439 | aromatic compound catabolic process | biological\_process | 4 |
| MSTRG.15060//MSTRG.15060.1:562-3237(+) | GO:0034641 | cellular nitrogen compound metabolic process | biological\_process | 3 |
| MSTRG.6838//MSTRG.6838.1:303-1271(+) | GO:0034641 | cellular nitrogen compound metabolic process | biological\_process | 3 |
| MSTRG.8179//MSTRG.8179.1:2-412(+) | GO:0034641 | cellular nitrogen compound metabolic process | biological\_process | 3 |
| MSTRG.1577//MSTRG.1577.1:455-2548(+) | GO:0034641 | cellular nitrogen compound metabolic process | biological\_process | 3 |
| MSTRG.6838//MSTRG.6838.1:303-1271(+) | GO:0034654 | nucleobase-containing compound biosynthetic process | biological\_process | 5 |
| MSTRG.15060//MSTRG.15060.1:562-3237(+) | GO:0034654 | nucleobase-containing compound biosynthetic process | biological\_process | 5 |
| MSTRG.15060//MSTRG.15060.1:562-3237(+) | GO:0044238 | primary metabolic process | biological\_process | 2 |
| MSTRG.6838//MSTRG.6838.1:303-1271(+) | GO:0044238 | primary metabolic process | biological\_process | 2 |
| MSTRG.8179//MSTRG.8179.1:2-412(+) | GO:0044238 | primary metabolic process | biological\_process | 2 |
| MSTRG.1577//MSTRG.1577.1:455-2548(+) | GO:0044238 | primary metabolic process | biological\_process | 2 |
| MSTRG.15060//MSTRG.15060.1:562-3237(+) | GO:0004536 | deoxyribonuclease activity | molecular\_function | 8 |
| MSTRG.15060//MSTRG.15060.1:562-3237(+) | GO:0000737 | DNA catabolic process, endonucleolytic | biological\_process | 8 |
| MSTRG.15060//MSTRG.15060.1:562-3237(+) | GO:0016893 | endonuclease activity, active with either ribo- or deoxyribonucleic acids and producing 5'-phosphomonoesters | molecular\_function | 9 |
| MSTRG.15060//MSTRG.15060.1:562-3237(+) | GO:0008152 | metabolic process | biological\_process | 1 |
| MSTRG.6838//MSTRG.6838.1:303-1271(+) | GO:0008152 | metabolic process | biological\_process | 1 |
| MSTRG.8179//MSTRG.8179.1:2-412(+) | GO:0008152 | metabolic process | biological\_process | 1 |
| MSTRG.1577//MSTRG.1577.1:455-2548(+) | GO:0008152 | metabolic process | biological\_process | 1 |
| MSTRG.15060//MSTRG.15060.1:562-3237(+) | GO:0044270 | cellular nitrogen compound catabolic process | biological\_process | 4 |
| MSTRG.6838//MSTRG.6838.1:303-1271(+) | GO:0006278 | RNA-dependent DNA biosynthetic process | biological\_process | 8 |
| MSTRG.15060//MSTRG.15060.1:562-3237(+) | GO:0006278 | RNA-dependent DNA biosynthetic process | biological\_process | 8 |
| MSTRG.8179//MSTRG.8179.1:2-412(+) | GO:1901363 | heterocyclic compound binding | molecular\_function | 2 |
| MSTRG.1577//MSTRG.1577.1:455-2548(+) | GO:1901363 | heterocyclic compound binding | molecular\_function | 2 |
| MSTRG.6838//MSTRG.6838.1:303-1271(+) | GO:0071897 | DNA biosynthetic process | biological\_process | 7 |
| MSTRG.15060//MSTRG.15060.1:562-3237(+) | GO:0071897 | DNA biosynthetic process | biological\_process | 7 |
| MSTRG.15060//MSTRG.15060.1:562-3237(+) | GO:0009036 | Type II site-specific deoxyribonuclease activity | molecular\_function | 11 |
| MSTRG.6838//MSTRG.6838.1:303-1271(+) | GO:0019438 | aromatic compound biosynthetic process | biological\_process | 4 |
| MSTRG.15060//MSTRG.15060.1:562-3237(+) | GO:0019438 | aromatic compound biosynthetic process | biological\_process | 4 |
| MSTRG.15060//MSTRG.15060.1:562-3237(+) | GO:1901575 | organic substance catabolic process | biological\_process | 3 |
| MSTRG.15060//MSTRG.15060.1:562-3237(+) | GO:0006807 | nitrogen compound metabolic process | biological\_process | 2 |
| MSTRG.6838//MSTRG.6838.1:303-1271(+) | GO:0006807 | nitrogen compound metabolic process | biological\_process | 2 |
| MSTRG.8179//MSTRG.8179.1:2-412(+) | GO:0006807 | nitrogen compound metabolic process | biological\_process | 2 |
| MSTRG.1577//MSTRG.1577.1:455-2548(+) | GO:0006807 | nitrogen compound metabolic process | biological\_process | 2 |
| MSTRG.1577//MSTRG.1577.1:455-2548(+) | GO:0008270 | zinc ion binding | molecular\_function | 6 |
| MSTRG.8179//MSTRG.8179.1:2-412(+) | GO:0015074 | DNA integration | biological\_process | 7 |
| MSTRG.1577//MSTRG.1577.1:455-2548(+) | GO:0015074 | DNA integration | biological\_process | 7 |
| MSTRG.15060//MSTRG.15060.1:562-3237(+) | GO:0006139 | nucleobase-containing compound metabolic process | biological\_process | 4 |
| MSTRG.6838//MSTRG.6838.1:303-1271(+) | GO:0006139 | nucleobase-containing compound metabolic process | biological\_process | 4 |
| MSTRG.8179//MSTRG.8179.1:2-412(+) | GO:0006139 | nucleobase-containing compound metabolic process | biological\_process | 4 |
| MSTRG.1577//MSTRG.1577.1:455-2548(+) | GO:0006139 | nucleobase-containing compound metabolic process | biological\_process | 4 |
| MSTRG.15060//MSTRG.15060.1:562-3237(+) | GO:0044237 | cellular metabolic process | biological\_process | 2 |
| MSTRG.6838//MSTRG.6838.1:303-1271(+) | GO:0044237 | cellular metabolic process | biological\_process | 2 |
| MSTRG.8179//MSTRG.8179.1:2-412(+) | GO:0044237 | cellular metabolic process | biological\_process | 2 |
| MSTRG.1577//MSTRG.1577.1:455-2548(+) | GO:0044237 | cellular metabolic process | biological\_process | 2 |
| MSTRG.8179//MSTRG.8179.1:2-412(+) | GO:0003676 | nucleic acid binding | molecular\_function | 3 |
| MSTRG.1577//MSTRG.1577.1:455-2548(+) | GO:0003676 | nucleic acid binding | molecular\_function | 3 |
| MSTRG.6838//MSTRG.6838.1:303-1271(+) | GO:0009058 | biosynthetic process | biological\_process | 2 |
| MSTRG.15060//MSTRG.15060.1:562-3237(+) | GO:0009058 | biosynthetic process | biological\_process | 2 |
| MSTRG.15060//MSTRG.15060.1:562-3237(+) | GO:0009057 | macromolecule catabolic process | biological\_process | 4 |
| MSTRG.15060//MSTRG.15060.1:562-3237(+) | GO:0044248 | cellular catabolic process | biological\_process | 3 |
| MSTRG.6838//MSTRG.6838.1:303-1271(+) | GO:0044710 | single-organism metabolic process | biological\_process | 2 |
| MSTRG.15060//MSTRG.15060.1:562-3237(+) | GO:0032199 | reverse transcription involved in RNA-mediated transposition | biological\_process | 10 |
| MSTRG.15060//MSTRG.15060.1:562-3237(+) | GO:0043170 | macromolecule metabolic process | biological\_process | 3 |
| MSTRG.6838//MSTRG.6838.1:303-1271(+) | GO:0043170 | macromolecule metabolic process | biological\_process | 3 |
| MSTRG.8179//MSTRG.8179.1:2-412(+) | GO:0043170 | macromolecule metabolic process | biological\_process | 3 |
| MSTRG.1577//MSTRG.1577.1:455-2548(+) | GO:0043170 | macromolecule metabolic process | biological\_process | 3 |
| MSTRG.15060//MSTRG.15060.1:562-3237(+) | GO:0044763 | single-organism cellular process | biological\_process | 2 |
| MSTRG.6838//MSTRG.6838.1:303-1271(+) | GO:0044763 | single-organism cellular process | biological\_process | 2 |
| MSTRG.15060//MSTRG.15060.1:562-3237(+) | GO:0090305 | nucleic acid phosphodiester bond hydrolysis | biological\_process | 6 |
| MSTRG.6838//MSTRG.6838.1:303-1271(+) | GO:0090305 | nucleic acid phosphodiester bond hydrolysis | biological\_process | 6 |
| MSTRG.15060//MSTRG.15060.1:562-3237(+) | GO:0032197 | transposition, RNA-mediated | biological\_process | 4 |
| MSTRG.6838//MSTRG.6838.1:303-1271(+) | GO:1901576 | organic substance biosynthetic process | biological\_process | 3 |
| MSTRG.15060//MSTRG.15060.1:562-3237(+) | GO:1901576 | organic substance biosynthetic process | biological\_process | 3 |
| MSTRG.15060//MSTRG.15060.1:562-3237(+) | GO:0016787 | hydrolase activity | molecular\_function | 2 |
| MSTRG.6838//MSTRG.6838.1:303-1271(+) | GO:0016787 | hydrolase activity | molecular\_function | 2 |
| MSTRG.15060//MSTRG.15060.1:562-3237(+) | GO:0044699 | single-organism process | biological\_process | 1 |
| MSTRG.6838//MSTRG.6838.1:303-1271(+) | GO:0044699 | single-organism process | biological\_process | 1 |
| MSTRG.1577//MSTRG.1577.1:455-2548(+) | GO:0005488 | binding | molecular\_function | 1 |
| MSTRG.8179//MSTRG.8179.1:2-412(+) | GO:0005488 | binding | molecular\_function | 1 |
| MSTRG.15060//MSTRG.15060.1:562-3237(+) | GO:0005488 | binding | molecular\_function | 1 |
| MSTRG.1577//MSTRG.1577.1:455-2548(+) | GO:0046914 | transition metal ion binding | molecular\_function | 5 |
| MSTRG.6838//MSTRG.6838.1:303-1271(+) | GO:0016779 | nucleotidyltransferase activity | molecular\_function | 4 |
| MSTRG.15060//MSTRG.15060.1:562-3237(+) | GO:0016779 | nucleotidyltransferase activity | molecular\_function | 4 |
| MSTRG.15060//MSTRG.15060.1:562-3237(+) | GO:0004519 | endonuclease activity | molecular\_function | 8 |
| MSTRG.6838//MSTRG.6838.1:303-1271(+) | GO:0004519 | endonuclease activity | molecular\_function | 8 |
| MSTRG.6838//MSTRG.6838.1:303-1271(+) | GO:0034061 | DNA polymerase activity | molecular\_function | 8 |
| MSTRG.15060//MSTRG.15060.1:562-3237(+) | GO:0034061 | DNA polymerase activity | molecular\_function | 8 |
| MSTRG.6838//MSTRG.6838.1:303-1271(+) | GO:0003964 | RNA-directed DNA polymerase activity | molecular\_function | 9 |
| MSTRG.15060//MSTRG.15060.1:562-3237(+) | GO:0003964 | RNA-directed DNA polymerase activity | molecular\_function | 9 |
| MSTRG.6838//MSTRG.6838.1:303-1271(+) | GO:1901362 | organic cyclic compound biosynthetic process | biological\_process | 4 |
| MSTRG.15060//MSTRG.15060.1:562-3237(+) | GO:1901362 | organic cyclic compound biosynthetic process | biological\_process | 4 |
| MSTRG.6838//MSTRG.6838.1:303-1271(+) | GO:0018130 | heterocycle biosynthetic process | biological\_process | 4 |
| MSTRG.15060//MSTRG.15060.1:562-3237(+) | GO:0018130 | heterocycle biosynthetic process | biological\_process | 4 |
| MSTRG.15060//MSTRG.15060.1:562-3237(+) | GO:0034655 | nucleobase-containing compound catabolic process | biological\_process | 5 |
| MSTRG.15060//MSTRG.15060.1:562-3237(+) | GO:0016888 | endodeoxyribonuclease activity, producing 5'-phosphomonoesters | molecular\_function | 10 |
| MSTRG.15060//MSTRG.15060.1:562-3237(+) | GO:1901360 | organic cyclic compound metabolic process | biological\_process | 3 |
| MSTRG.6838//MSTRG.6838.1:303-1271(+) | GO:1901360 | organic cyclic compound metabolic process | biological\_process | 3 |
| MSTRG.8179//MSTRG.8179.1:2-412(+) | GO:1901360 | organic cyclic compound metabolic process | biological\_process | 3 |
| MSTRG.1577//MSTRG.1577.1:455-2548(+) | GO:1901360 | organic cyclic compound metabolic process | biological\_process | 3 |
| MSTRG.15060//MSTRG.15060.1:562-3237(+) | GO:0006308 | DNA catabolic process | biological\_process | 7 |
| MSTRG.15060//MSTRG.15060.1:562-3237(+) | GO:0004518 | nuclease activity | molecular\_function | 7 |
| MSTRG.6838//MSTRG.6838.1:303-1271(+) | GO:0004518 | nuclease activity | molecular\_function | 7 |

| Seqid | GOid | GOname | GOcategory | GOlevel |
| --- | --- | --- | --- | --- |
| MSTRG.15060//MSTRG.15060.1:562-3237(+) | GO:0004520 | endodeoxyribonuclease activity | molecular\_function | 9 |
| MSTRG.1577//MSTRG.1577.1:455-2548(+) | GO:0046872 | metal ion binding | molecular\_function | 4 |
| MSTRG.15060//MSTRG.15060.1:562-3237(+) | GO:0046872 | metal ion binding | molecular\_function | 4 |
| MSTRG.15060//MSTRG.15060.1:562-3237(+) | GO:0009056 | catabolic process | biological\_process | 2 |
| MSTRG.15060//MSTRG.15060.1:562-3237(+) | GO:0044265 | cellular macromolecule catabolic process | biological\_process | 5 |
| MSTRG.6838//MSTRG.6838.1:303-1271(+) | GO:0016772 | transferase activity, transferring phosphorus-containing groups | molecular\_function | 3 |
| MSTRG.15060//MSTRG.15060.1:562-3237(+) | GO:0016772 | transferase activity, transferring phosphorus-containing groups | molecular\_function | 3 |
| MSTRG.15060//MSTRG.15060.1:562-3237(+) | GO:0001171 | reverse transcription | biological\_process | 9 |
| MSTRG.1577//MSTRG.1577.1:455-2548(+) | GO:0043167 | ion binding | molecular\_function | 2 |
| MSTRG.15060//MSTRG.15060.1:562-3237(+) | GO:0043167 | ion binding | molecular\_function | 2 |
| … | … | … | … | … |

| Seqid | GOid | GOname | GOcategory | GOlevel |
| --- | --- | --- | --- | --- |
| MSTRG.15060//MSTRG.15060.1:562-3237(+) | GO:0004520 | endodeoxyribonuclease activity | molecular\_function | 9 |
| MSTRG.1577//MSTRG.1577.1:455-2548(+) | GO:0046872 | metal ion binding | molecular\_function | 4 |
| MSTRG.15060//MSTRG.15060.1:562-3237(+) | GO:0046872 | metal ion binding | molecular\_function | 4 |
| MSTRG.15060//MSTRG.15060.1:562-3237(+) | GO:0009056 | catabolic process | biological\_process | 2 |
| MSTRG.15060//MSTRG.15060.1:562-3237(+) | GO:0044265 | cellular macromolecule catabolic process | biological\_process | 5 |
| MSTRG.6838//MSTRG.6838.1:303-1271(+) | GO:0016772 | transferase activity, transferring phosphorus-containing groups | molecular\_function | 3 |
| MSTRG.15060//MSTRG.15060.1:562-3237(+) | GO:0016772 | transferase activity, transferring phosphorus-containing groups | molecular\_function | 3 |
| MSTRG.15060//MSTRG.15060.1:562-3237(+) | GO:0001171 | reverse transcription | biological\_process | 9 |
| MSTRG.1577//MSTRG.1577.1:455-2548(+) | GO:0043167 | ion binding | molecular\_function | 2 |
| MSTRG.15060//MSTRG.15060.1:562-3237(+) | GO:0043167 | ion binding | molecular\_function | 2 |
| … | … | … | … | … |

… … … … …

  
  

| 表头 | 表头说明 |
| --- | --- |
| Seq\_ID | 序列ID |
| GO\_ID | Gene Ontology ID |
| GO\_name | GO名称 |
| GO\_category | GO类别 |
| GO\_level | GO树的层级 |

  

### 编码蛋白质新转录本的KEGG Pathway注释(novel\_protein2KEGG.xls)

显示 10全文 行  
ASSEMBLY-merge
 

| Seq\_id | KO | Pathway\_id | Pathway\_name | Pathway\_class |
| --- | --- | --- | --- | --- |
|
|  |

| Seq\_id | KO | Pathway\_id | Pathway\_name | Pathway\_class |
| --- | --- | --- | --- | --- |
|
|  |

| Seq\_id | KO | Pathway\_id | Pathway\_name | Pathway\_class |
| --- | --- | --- | --- | --- |
|
|  |

  
  

| 表头 | 表头说明 |
| --- | --- |
| Seq\_ID | 序列ID |
| Pathway\_ID | KEGG Pathway ID |
| Pathway\_class | KEGG Pathway类别 |
| Pathway\_name | KEGG Pathway名称 |

  

### 编码蛋白质新转录本的Interpro注释(novel\_protein2domain.xls)

显示 1020全文 行  
ASSEMBLY-merge
 

| Seqid | Interproscan\_id | Interproscan\_name |
| --- | --- | --- |
| MSTRG.6838//MSTRG.6838.1:303-1271(+) | IPR000477 | Reverse transcriptase domain |
| MSTRG.6838//MSTRG.6838.1:303-1271(+) | IPR005135 | Endonuclease/exonuclease/phosphatase |
| MSTRG.15060//MSTRG.15060.1:562-3237(+) | IPR000477 | Reverse transcriptase domain |
| MSTRG.15060//MSTRG.15060.1:562-3237(+) | IPR013544 | Domain of unknown function DUF1725 |
| MSTRG.15060//MSTRG.15060.1:562-3237(+) | IPR005135 | Endonuclease/exonuclease/phosphatase |
| MSTRG.1577//MSTRG.1577.1:455-2548(+) | IPR001584 | Integrase, catalytic core |
| MSTRG.1577//MSTRG.1577.1:455-2548(+) | IPR021109 | Aspartic peptidase domain |
| MSTRG.1577//MSTRG.1577.1:455-2548(+) | IPR012337 | Ribonuclease H-like domain |
| MSTRG.1577//MSTRG.1577.1:455-2548(+) | IPR000477 | Reverse transcriptase domain |
| MSTRG.1577//MSTRG.1577.1:455-2548(+) | IPR001878 | Zinc finger, CCHC-type |
| MSTRG.8179//MSTRG.8179.1:2-412(+) | IPR001584 | Integrase, catalytic core |
| MSTRG.8179//MSTRG.8179.1:2-412(+) | IPR012337 | Ribonuclease H-like domain |

| Seqid | Interproscan\_id | Interproscan\_name |
| --- | --- | --- |
| MSTRG.6838//MSTRG.6838.1:303-1271(+) | IPR000477 | Reverse transcriptase domain |
| MSTRG.6838//MSTRG.6838.1:303-1271(+) | IPR005135 | Endonuclease/exonuclease/phosphatase |
| MSTRG.15060//MSTRG.15060.1:562-3237(+) | IPR000477 | Reverse transcriptase domain |
| MSTRG.15060//MSTRG.15060.1:562-3237(+) | IPR013544 | Domain of unknown function DUF1725 |
| MSTRG.15060//MSTRG.15060.1:562-3237(+) | IPR005135 | Endonuclease/exonuclease/phosphatase |
| MSTRG.1577//MSTRG.1577.1:455-2548(+) | IPR001584 | Integrase, catalytic core |
| MSTRG.1577//MSTRG.1577.1:455-2548(+) | IPR021109 | Aspartic peptidase domain |
| MSTRG.1577//MSTRG.1577.1:455-2548(+) | IPR012337 | Ribonuclease H-like domain |
| MSTRG.1577//MSTRG.1577.1:455-2548(+) | IPR000477 | Reverse transcriptase domain |
| MSTRG.1577//MSTRG.1577.1:455-2548(+) | IPR001878 | Zinc finger, CCHC-type |
| … | … | … |

| Seqid | Interproscan\_id | Interproscan\_name |
| --- | --- | --- |
| MSTRG.6838//MSTRG.6838.1:303-1271(+) | IPR000477 | Reverse transcriptase domain |
| MSTRG.6838//MSTRG.6838.1:303-1271(+) | IPR005135 | Endonuclease/exonuclease/phosphatase |
| MSTRG.15060//MSTRG.15060.1:562-3237(+) | IPR000477 | Reverse transcriptase domain |
| MSTRG.15060//MSTRG.15060.1:562-3237(+) | IPR013544 | Domain of unknown function DUF1725 |
| MSTRG.15060//MSTRG.15060.1:562-3237(+) | IPR005135 | Endonuclease/exonuclease/phosphatase |
| MSTRG.1577//MSTRG.1577.1:455-2548(+) | IPR001584 | Integrase, catalytic core |
| MSTRG.1577//MSTRG.1577.1:455-2548(+) | IPR021109 | Aspartic peptidase domain |
| MSTRG.1577//MSTRG.1577.1:455-2548(+) | IPR012337 | Ribonuclease H-like domain |
| MSTRG.1577//MSTRG.1577.1:455-2548(+) | IPR000477 | Reverse transcriptase domain |
| MSTRG.1577//MSTRG.1577.1:455-2548(+) | IPR001878 | Zinc finger, CCHC-type |
| … | … | … |

… … …

  
  

| 表头 | 表头说明 |
| --- | --- |
| Seq\_ID | 蛋白序列编号 |
| Interproscan\_ID | 数据库编号 |
| Interproscan\_name | 结构域名称 |

  

### 潜在编码蛋白序列gff3位置文件说明(assembled\_transcripts\_\*.gtf)

| 列号 | 列说明 |
| --- | --- |
| 第1列 | 转录本标识 |
| 第2列 | 预测蛋白质方法 |
| 第3列 | 区域类型gene，mRNA，exon，CDS，five\_primer\_UTR，three\_primer\_UTR，其中CDS代表潜在蛋白质序列区域，\*\_UTR代表潜在UTR序列区域 |
| 第4列 | 开始位置(1based) |
| 第5列 | 结束位置(1based) |
| 第6列 | 点号 |
| 第7列 | 编码蛋白序列区域在转录本的方向 |
| 第8列 | 点号 |
| 第9列 | 编码蛋白序列区域编号，长度，方向(+/-)，类型，其中分为internal， 不包括起始密码子和终止密码子的不完整蛋白质序列，5prime\_partial，不包括起始密码子包括终止密码子的不完整蛋白质序列，3prime\_partial， 包括起始密码子不包括终止密码子的不完整蛋白质序列，complete，包括起始密码子包括终止密码子的完整蛋白质序列 |

  
  

  


# 5. 基因表达

## 5.1 基因整体表达水平

目录链接

  
  
  
  

针对所有样本采用StringTie软件统计已知基因的原始序列计数，已知基因的表达量计算采用FPKM计算度量指标(FPKM- Fragments Per Kilobase of transcript per Million fragments mapped)。 FPKM含义是以每百万比配成对序列每1Kbp长度做转录本表达量指标， 其中转录本长度和总比配成对read数目用于归一化表达量数值。FPKM计算公式为 FPKM=total fragments / mapped reads(millions) \* exon length(KB)。 采用StringTie计算各个样本FPKM表达量取log2(FPKM+1)，基因的外显子长度定义为基因内部已知转录本的外显子非冗余区域的长度和， Mapped reads定义为比对到参考基因组序列的序列对总数。 根据各个基因FPKM表达量分别在[0-1]，[1-5]，[5-10]，[10-20]，[20-30]，[30-40]，[40-50]，[>=50]区间范围内统计每个样本中转录本个数并计算占有表达转录本总数百分比， 一般来说FPKM >= 0.1表示转录本有表达。

结果见报告-> 4-DGE > gene\_sample\_FPKM\_stat.xls

### FPKM表达量按照区间统计(gene\_sample\_FPKM\_stat.xls)

|
|  |
|

  
  

注：Boxplot图是利用数据中的五个统计量：最小值、第一四分位数（25%）、中位数（50%）、第三四分位数（75%）和最大值来描述数据的一种方法，它也可以粗略地看出各样本的基因表达是否具有有对称性，分布的分散程度等信息。

  
  

注：PCA图是通过降维度的方式来看样本间的相似程度，样本用不同标号显示，样本距离越近表示样本基因的表达趋势越接近。

  
  

注：样本相关系数聚类图是通过计算样本间相关系数构建的层级聚类图。

  
  


## 5.2 差异表达基因筛选

目录链接

  
  
  
  

针对实验设计利用DESeq2软件对不同样本组之间筛选差异表达基因，满足|log2FC| >= 1 和 Pvalue <= 0.05 差异表达范围筛选两组之间的差异基因。

差异分析结果见报告-> 4-DGE > \*\_vs\_\* > gene\_exp\_significant\_with\_symbol\_GO\_KEGG\_FPKM.xls

  

### 差异表达转录本筛选结果说明(gene\_exp\_significant\_\*.xls)

显示 10203050100150全文 行  
DGE-S20mg\_vs\_op50
 

| Gene\_ID | Symbol | description | op50\_1 | op50\_2 | op50\_3 | S20mg\_1 | S20mg\_2 | S20mg\_3 | G1 | G2 | G2\_vs\_G1\_log2FoldChange | G2\_vs\_G1\_Pvalue | G2\_vs\_G1\_FDR | biological\_process | cellular\_component | molecular\_function | KEGG\_pathway |
| --- | --- | --- | --- | --- | --- | --- | --- | --- | --- | --- | --- | --- | --- | --- | --- | --- | --- |
| WBGene00014161 | arrd-5 | --- | 4.69113265039224 | 4.59163971678492 | 5.09884313101069 | 0 | 4.01276678014068 | 3.6337778023327 | 4.79387183272928 | 2.54884819415779 | -2.24502363857149 | 0.0427566498311083 | 0.529320350796076 | --- | --- | --- | --- |
| WBGene00008529 | F02E9.3 | --- | 5.48976675508523 | 6.97500206945319 | 6.95758920437985 | 8.12630528602139 | 7.99353174469564 | 6.66980166461679 | 6.47411934297276 | 7.59654623177794 | 1.12242688880518 | 0.0123667749936071 | 0.413425244614386 | --- | --- | --- | --- |
| WBGene00012372 | W09H1.1 | --- | 7.21029768889039 | 6.77641041662129 | 7.20726960578539 | 5.80830885368026 | 5.32882278978024 | 6.32084188278136 | 7.06465923709902 | 5.81932450874729 | -1.24533472835174 | 0.000339354826826513 | 0.115716049961483 | --- | --- | --- | --- |
| WBGene00016619 | C43H6.6 | --- | 7.70883252812198 | 6.79153863525114 | 7.05635084982705 | 5.78025451726996 | 5.81312840960928 | 6.65626167202517 | 7.18557400440006 | 6.08321486630147 | -1.10235913809859 | 0.00269037000954025 | 0.262548496842802 | --- | --- | --- | ko04977//Vitamin digestion and absorption |
| WBGene00021233 | Y19D10B.4 | --- | 0 | 0 | 0 | 3.26802428728875 | 1.47583054764563 | 2.26433442226316 | 0 | 2.33606308573251 | 2.33606308573251 | 0.0181355419170823 | 0.443675833929587 | --- | --- | --- | --- |
| WBGene00020547 | srz-7 | --- | 0 | 2.15920951431739 | 3.4309644979598 | 0 | 0 | 0 | 1.86339133742573 | 0 | -1.86339133742573 | 0.047529690000053 | 0.547450180381601 | --- | --- | --- | --- |
| WBGene00005408 | srh-195 | integral component of membrane | 6.04324273772971 | 5.20251847810575 | 6.46086016951256 | 4.86086841734319 | 4.8827895448963 | 4.73764398207432 | 5.90220712844934 | 4.8271006481046 | -1.07510648034474 | 0.00617910925759978 | 0.338641765940775 | --- | GO:0031224//intrinsic component of membrane,GO:0016020//membrane,GO:0044425//membrane part,GO:0016021//integral component of membrane | --- | --- |
| WBGene00012945 | Y47H9A.1 | --- | 9.53187586302052 | 8.46535368459049 | 8.96023912772479 | 7.51867395156043 | 7.53433209201753 | 8.85557167350941 | 8.9858228917786 | 7.96952590569579 | -1.01629698608281 | 0.0104276735123871 | 0.388300803894593 | --- | --- | --- | --- |
| WBGene00004149 | trpl-5 | --- | 1.54112629318484 | 2.76082432963029 | 1.56414170329639 | 4.42653282953089 | 4.48396063605928 | 2.52491846837501 | 1.95536410870384 | 3.81180397798839 | 1.85643986928455 | 0.0134996960071696 | 0.421564246360991 | --- | --- | --- | --- |
| WBGene00009041 | cut-3 | --- | 10.2087510697115 | 9.7061838707901 | 10.0171718238778 | 8.57313020008737 | 8.41093376145063 | 9.20853141411787 | 9.9773689214598 | 8.73086512521862 | -1.24650379624118 | 6.63083830197897e-08 | 0.000277784761722191 | --- | --- | --- | --- |
| WBGene00015068 | srt-68 | --- | 1.95058941391142 | 3.18411645795122 | 2.29690683561209 | 0 | 0 | 0 | 2.47720423582491 | 0 | -2.47720423582491 | 0.011764465030506 | 0.406223711323865 | --- | --- | --- | --- |
| WBGene00015062 | trx-1 | --- | 5.22087574604194 | 4.90155095969583 | 4.36248061248209 | 3.68602244576112 | 3.54703312181276 | 3.93227165856632 | 4.82830243940662 | 3.72177574204673 | -1.10652669735989 | 0.0261673629620215 | 0.481198798674787 | --- | --- | --- | --- |
| WBGene00021032 | clec-118 | --- | 3.1111756519858 | 2.98793946356524 | 2.55888921229042 | 4.00972402417049 | 4.48396063605928 | 4.01946253970874 | 2.88600144261382 | 4.17104906664617 | 1.28504762403235 | 0.0359757426973084 | 0.512628112049838 | --- | --- | --- | --- |
| WBGene00022699 | ZK353.3 | --- | 2.75074548289308 | 4.28459328350387 | 4.96964493681175 | 5.63116524831505 | 6.15718058499202 | 5.17752027863485 | 4.0016612344029 | 5.65528870398064 | 1.65362746957774 | 0.00940228823658716 | 0.377752447714155 | --- | --- | --- | --- |
| WBGene00010854 | M04C7.3 | --- | 6.08443773604644 | 4.52078489894459 | 5.6417015684825 | 4.00972402417049 | 3.02250078652137 | 4.51569237370833 | 5.41564140115784 | 3.8493057281334 | -1.56633567302445 | 0.0047701123818735 | 0.306762161400089 | --- | --- | --- | --- |
| WBGene00196553 | T05C1.7 | --- | 0.967248346793504 | 2.76082432963029 | 2.55888921229042 | 0 | 0 | 0 | 2.09565396290474 | 0 | -2.09565396290474 | 0.0371627268700382 | 0.517220675704335 | --- | --- | --- | --- |
| WBGene00001664 | gpa-2 | --- | 8.05810020550279 | 7.72323882121186 | 8.06680275548752 | 6.72353524864349 | 6.34278006579546 | 7.48262484952948 | 7.94938059406739 | 6.84964672132281 | -1.09973387274458 | 0.000957816065876994 | 0.182178613991442 | --- | --- | --- | ko04725//Cholinergic synapse,ko05145//Toxoplasmosis,ko04726//Serotonergic synapse,ko04723//Retrograde endocannabinoid signaling,ko05034//Alcoholism,ko04915//Estrogen signaling pathway,ko04728//Dopaminergic synapse,ko04730//Long-term depression,ko04916//Melanogenesis,ko05142//Chagas disease (American trypanosomiasis),ko04713//Circadian entrainment,ko05032//Morphine addiction,ko04724//Glutamatergic synapse,ko04727//GABAergic synapse |
| WBGene00018439 | fbxa-184 | --- | 0 | 4.28459328350387 | 3.877656608957 | 5.31649607097358 | 5.66903363932878 | 4.10168291453629 | 2.72074996415362 | 5.02907087494622 | 2.30832091079259 | 0.0413511945302929 | 0.525959160408246 | --- | --- | --- | --- |
| WBGene00013521 | Y73F8A.12 | --- | 3.84514002926505 | 4.28459328350387 | 4.29216665132285 | 5.31649607097358 | 6.2274647115546 | 4.97428232339812 | 4.14063332136392 | 5.50608103530877 | 1.36544771394484 | 0.00300808386294642 | 0.271870574624068 | --- | --- | --- | --- |
| WBGene00013296 | Y57G11B.3 | --- | 3.84514002926505 | 5.05986881279458 | 4.49359406380416 | 5.53370728074314 | 6.40475924352026 | 5.60152093819677 | 4.46620096862126 | 5.84666248748672 | 1.38046151886546 | 0.00330014408723701 | 0.277192646374062 | --- | --- | --- | --- |
| WBGene00007314 | gcy-29 | --- | 7.11411253524034 | 6.74566959003539 | 6.36134963878335 | 5.59940518351477 | 5.15938804268104 | 6.138097123376 | 6.74037725468636 | 5.6322967831906 | -1.10808047149576 | 0.00312389942697991 | 0.273481902846497 | --- | --- | --- | ko04744//Phototransduction,ko00230//Purine metabolism |
| WBGene00005422 | srh-212 | integral component of membrane | 7.22878846118613 | 5.66361142672134 | 6.4925613769898 | 5.10659485791198 | 3.84406212723193 | 6.35476868340023 | 6.46165375496576 | 5.10180855618138 | -1.35984519878438 | 0.0452031021613607 | 0.540333587186214 | --- | GO:0031224//intrinsic component of membrane,GO:0016020//membrane,GO:0044425//membrane part,GO:0016021//integral component of membrane | --- | --- |
| WBGene00020242 | phat-5 | --- | 9.79799476918098 | 9.85401662982874 | 10.0089748414064 | 8.8238991762479 | 8.5511384457074 | 8.87323569360394 | 9.88699541347204 | 8.74942443851975 | -1.13757097495229 | 1.28656103292971e-13 | 1.88642011453319e-09 | --- | --- | --- | --- |
| WBGene00020240 | T05B4.9 | --- | 0 | 3.65026690971359 | 3.97063416719798 | 5.59940518351477 | 5.39139161201497 | 3.83947041366737 | 2.54030035897052 | 4.9434224030657 | 2.40312204409518 | 0.0209456182486593 | 0.457581046440414 | --- | --- | --- | --- |
| WBGene00077768 | K04G2.12 | --- | 3.52433812248388 | 4.28459328350387 | 3.14209662450744 | 2.41051223419307 | 2.18994296053331 | 1.94607408114065 | 3.65034267683173 | 2.18217642528901 | -1.46816625154272 | 0.0370530285883733 | 0.517170764262389 | --- | --- | --- | --- |
| WBGene00001362 | exc-1 | --- | 6.59261857699403 | 5.66361142672134 | 5.80210086391771 | 5.15108941575715 | 4.74582030763867 | 4.39044193885787 | 6.01944362254436 | 4.76245055408456 | -1.2569930684598 | 0.00232482395118312 | 0.260973196997361 | --- | --- | --- | --- |
| WBGene00015611 | C08F1.8 | --- | 7.6687638088864 | 6.50992084691615 | 6.55394757357561 | 4.80626946887653 | 5.26341688783582 | 6.43625118784829 | 6.91087740979272 | 5.50197918152021 | -1.40889822827251 | 0.00474743523190745 | 0.306553778601445 | --- | --- | --- | --- |
| WBGene00017562 | F18C5.9 | --- | 4.84288501226145 | 5.05986881279458 | 4.14033939827645 | 3.55968471353959 | 2.66546365057537 | 0 | 4.68103107444416 | 2.07504945470499 | -2.60598161973917 | 0.005877053154859 | 0.332573371918942 | --- | --- | --- | --- |
| WBGene00016233 | srt-58 | --- | 5.95714652822996 | 4.72369344023804 | 5.87607365934219 | 3.68602244576112 | 3.43267949213248 | 4.78817883564214 | 5.51897120927006 | 3.96896025784525 | -1.55001095142482 | 0.00461711032202495 | 0.30021454588333 | --- | --- | --- | --- |
| WBGene00019265 | H35N09.1 | extracellular region | 6.5043050852378 | 5.24709728418621 | 5.09884313101069 | 2.67712217824935 | 3.54703312181276 | 5.38875843712698 | 5.6167485001449 | 3.8709712457297 | -1.7457772544152 | 0.0309309803424666 | 0.50197006666041 | --- | GO:0005576//extracellular region | --- | --- |
| WBGene00015345 | C02F5.2 | --- | 3.84514002926505 | 4.44626965381121 | 4.6137781794739 | 5.96606093706295 | 5.83580467125913 | 5.28700207279648 | 4.30172928751672 | 5.69628922703952 | 1.3945599395228 | 0.00143219064660326 | 0.223232898952113 | --- | --- | --- | --- |
| WBGene00000738 | col-165 | --- | 11.4150544917448 | 10.6627615453566 | 10.7920397554409 | 9.13484207320498 | 9.32914181195297 | 10.2983848009607 | 10.9566185975141 | 9.58745622870622 | -1.36916236880788 | 0.00235167449064765 | 0.260973196997361 | --- | --- | --- | --- |
| WBGene00008390 | D1086.3 | --- | 6.77981544755181 | 7.76215608164129 | 8.55745196729412 | 9.08410983111399 | 8.77004077366869 | 8.40358320393948 | 7.69980783216241 | 8.75257793624072 | 1.05277010407831 | 0.0131143881112835 | 0.421187542609031 | --- | --- | --- | --- |
| WBGene00044261 | Y87G2A.20 | --- | 2.94218725187982 | 4.10248475785757 | 5.13945998188477 | 5.23616195420957 | 5.88011514039006 | 5.21494498072375 | 4.06137733054072 | 5.44374069177446 | 1.38236336123374 | 0.0406559332996613 | 0.525699473916166 | --- | --- | --- | --- |
| WBGene00010487 | K02A11.2 | --- | 3.1111756519858 | 2.98793946356524 | 4.87660150221946 | 5.06068424222558 | 5.56429792063246 | 4.92995836187629 | 3.6585722059235 | 5.18498017491144 | 1.52640796898794 | 0.0326684650378347 | 0.506076459183572 | --- | --- | --- | --- |
| WBGene00194652 | F15H9.8 | --- | 0 | 0 | 0 | 4.35225271272185 | 3.02250078652137 | 2.26433442226316 | 0 | 3.21302930716879 | 3.21302930716879 | 0.000412296071669277 | 0.127269287386332 | --- | --- | --- | --- |
| WBGene00304203 | D1086.21 | --- | 5.57894761177708 | 5.20251847810575 | 5.66970400104249 | 2.41051223419307 | 4.2337632522627 | 5.05904292354522 | 5.48372336364177 | 3.901106136667 | -1.58261722697478 | 0.033530595858159 | 0.507370858380038 | --- | --- | --- | --- |
| WBGene00018767 | F53G2.2 | --- | 2.26916251373634 | 1.72732524432905 | 3.29374844389101 | 3.80218203025436 | 3.6529845535538 | 4.92995836187629 | 2.43007873398547 | 4.12837498189482 | 1.69829624790935 | 0.0215855286921085 | 0.460906201874755 | --- | --- | --- | --- |
| WBGene00007398 | C07A4.3 | extracellular region | 4.69113265039224 | 5.95988701501225 | 6.01352918779826 | 7.43117218725375 | 6.87877607670145 | 6.65626167202517 | 5.55484961773425 | 6.98873664532679 | 1.43388702759254 | 0.000685035330006314 | 0.158178433483741 | --- | GO:0005576//extracellular region | --- | --- |
| WBGene00017918 | F29A7.8 | --- | 4.58030189189444 | 3.35678254004612 | 1.97655534744632 | 0 | 0 | 1.94607408114065 | 3.30454659312896 | 0.648691360380217 | -2.65585523274874 | 0.00896475922906255 | 0.376792615567032 | --- | --- | --- | --- |
| WBGene00005138 | srd-61 | integral component of membrane | 4.25905046063254 | 0 | 0 | 0 | 0 | 0 | 1.41968348687751 | 0 | -1.41968348687751 | 0.0450970439274519 | 0.540216053574397 | --- | GO:0031224//intrinsic component of membrane,GO:0016020//membrane,GO:0044425//membrane part,GO:0016021//integral component of membrane | --- | --- |
| WBGene00009728 | sdz-19 | --- | 4.63678128998955 | 4.72369344023804 | 4.29216665132285 | 3.80218203025436 | 2.44720705578603 | 3.6337778023327 | 4.55088046051681 | 3.29438896279103 | -1.25649149772578 | 0.0442565423475274 | 0.53772899875954 | --- | --- | --- | --- |
| WBGene00017497 | irld-32 | --- | 5.57894761177708 | 4.28459328350387 | 4.14033939827645 | 3.26802428728875 | 3.75168443677823 | 2.93688715379468 | 4.66796009785247 | 3.31886529262055 | -1.34909480523191 | 0.0167262464191981 | 0.439345144126105 | --- | --- | --- | --- |
| WBGene00269436 | F13A2.14 | --- | 5.39470779007679 | 4.72369344023804 | 4.77714121903117 | 3.80218203025436 | 3.75168443677823 | 4.01946253970874 | 4.96518081644867 | 3.85777633558044 | -1.10740448086822 | 0.0198720355940931 | 0.452443667544085 | --- | --- | --- | --- |
| WBGene00017359 | F10E9.10 | --- | 3.74592621346043 | 5.24709728418621 | 3.877656608957 | 2.67712217824935 | 3.54703312181276 | 2.74555808119468 | 4.29022670220121 | 2.98990446041893 | -1.30032224178228 | 0.0361317246561503 | 0.513584449370317 | --- | --- | --- | --- |
| WBGene00009470 | F36D3.4 | protein homodimerization activity | 6.04324273772971 | 7.96925661759167 | 7.61986436178503 | 8.68327505005959 | 8.5916735889573 | 7.41187161489734 | 7.21078790570214 | 8.22894008463808 | 1.01815217893594 | 0.0358333626503691 | 0.512341959883996 | --- | --- | GO:0042803//protein homodimerization activity,GO:0046983//protein dimerization activity,GO:0005488//binding,GO:0005515//protein binding,GO:0042802//identical protein binding | --- |
| WBGene00005305 | srh-84 | integral component of membrane | 3.93796774921411 | 3.65026690971359 | 1.56414170329639 | 0 | 0 | 1.94607408114065 | 3.05079212074136 | 0.648691360380217 | -2.40210076036115 | 0.0232587221860548 | 0.470368969204507 | --- | GO:0031224//intrinsic component of membrane,GO:0016020//membrane,GO:0044425//membrane part,GO:0016021//integral component of membrane | --- | --- |
| WBGene00018708 | oac-32 | --- | 0 | 2.15920951431739 | 0 | 3.68602244576112 | 2.85499905925905 | 3.51877429894335 | 0.719736504772463 | 3.35326526798784 | 2.63352876321538 | 0.0168757621927542 | 0.44052189561082 | --- | --- | --- | --- |
| WBGene00010574 | K04H4.5 | --- | 6.71571190110007 | 8.46535368459049 | 8.07729429133224 | 9.1595545100037 | 8.94449736180138 | 8.18997788177063 | 7.75278662567427 | 8.76467658452524 | 1.01188995885097 | 0.0177034761928924 | 0.443675833929587 | --- | --- | --- | --- |
| WBGene00220178 | Y52D3.2 | --- | 0.967248346793504 | 5.00900241559391 | 0.984412639926697 | 1.05599915505846 | 0.918934670839788 | 0.964274272708593 | 2.3202211341047 | 0.979736032868947 | -1.34048510123576 | 0.0220157188412236 | 0.462073973922094 | --- | --- | --- | --- |
| WBGene00007057 | ant-1.2 | --- | 6.96767640773586 | 5.15651816051488 | 6.23585304165929 | 4.00972402417049 | 4.42536048117021 | 6.07843321717785 | 6.12001586997001 | 4.83783924083952 | -1.28217662913049 | 0.0482230822459168 | 0.5488946756657 | --- | --- | --- | ko05016//Huntington\*s disease,ko05166//HTLV-I infection,ko05012//Parkinson\*s disease,ko04020//Calcium signaling pathway |
| WBGene00021804 | Y53G8AM.4 | --- | 4.18522789582735 | 4.10248475785757 | 3.4309644979598 | 1.65915520751611 | 0 | 2.52491846837501 | 3.90622571721491 | 1.39469122529704 | -2.51153449191787 | 0.0067730583998486 | 0.348650608112257 | --- | --- | --- | --- |
| WBGene00005540 | sri-28 | --- | 5.25735820612055 | 3.89402242725002 | 2.78052971426822 | 2.90206314089939 | 2.18994296053331 | 2.52491846837501 | 3.97730344921293 | 2.53897485660257 | -1.43832859261036 | 0.020665651265898 | 0.45594653910607 | --- | --- | --- | --- |
| WBGene00005542 | sri-30 | --- | 6.00083678684673 | 4.95627682518266 | 5.52391793382715 | 4.56451905955733 | 3.84406212723193 | 5.01728501126819 | 5.49367718195218 | 4.47528873268582 | -1.01838844926636 | 0.0359150520862094 | 0.512376675182869 | --- | --- | --- | --- |
| WBGene00018545 | nhr-189 | --- | 4.63678128998955 | 4.10248475785757 | 1.97655534744632 | 0 | 2.18994296053331 | 2.52491846837501 | 3.57194046509781 | 1.57162047630277 | -2.00031998879504 | 0.028616162916888 | 0.487183645467303 | --- | --- | --- | --- |
| WBGene00018543 | ugt-32 | --- | 6.68925110055564 | 5.66361142672134 | 6.65546317217305 | 4.1911300965271 | 4.69711331580184 | 5.68390234537511 | 6.33610856648334 | 4.85738191923468 | -1.47872664724866 | 0.00255588422557876 | 0.260973196997361 | --- | --- | --- | ko05204//Chemical carcinogenesis,ko00040//Pentose and glucuronate interconversions,ko00830//Retinol metabolism,ko00983//Drug metabolism - other enzymes,ko00053//Ascorbate and aldarate metabolism,ko01100//Metabolic pathways,ko00140//Steroid hormone biosynthesis,ko00980//Metabolism of xenobiotics by cytochrome P450,ko00500//Starch and sucrose metabolism,ko00860//Porphyrin and chlorophyll metabolism,ko00982//Drug metabolism - cytochrome P450 |
| WBGene00012727 | Y39G8B.7 | --- | 0 | 3.51097475443316 | 2.55888921229042 | 3.26802428728875 | 5.39139161201497 | 4.68527453225857 | 2.02328798890786 | 4.4482301438541 | 2.42494215494624 | 0.0120844560062942 | 0.410524674504398 | --- | --- | --- | --- |
| WBGene00013186 | Y53H1C.3 | --- | 1.95058941391142 | 4.90155095969583 | 4.77714121903117 | 6.21689560712181 | 6.13906120190136 | 5.28700207279648 | 3.87642719754614 | 5.88098629393988 | 2.00455909639374 | 0.00936321016495181 | 0.377752447714155 | --- | --- | --- | --- |
| WBGene00012119 | T28C6.3 | --- | 5.45877119202772 | 5.489014266081 | 5.36172672853657 | 6.90336782694958 | 6.24451347054481 | 6.37143751277807 | 5.4365040622151 | 6.50643960342415 | 1.06993554120906 | 0.00114163970143922 | 0.193517828004077 | --- | --- | --- | --- |
| WBGene00011137 | R08A2.7 | --- | 7.91699744087421 | 7.16122985125502 | 7.33516927738697 | 5.78025451726996 | 6.13906120190136 | 7.10806210062809 | 7.47113218983873 | 6.34245927326647 | -1.12867291657226 | 0.00535324490123241 | 0.31991317640062 | --- | --- | --- | --- |
| WBGene00011132 | R08A2.1 | --- | 5.14502076065917 | 6.69829415935076 | 7.14878263880242 | 7.89078425848248 | 7.98848068758395 | 6.74847683511628 | 6.33069918627078 | 7.54258059372757 | 1.21188140745679 | 0.0187937391196552 | 0.444704064511801 | --- | --- | --- | --- |
| WBGene00235285 | F56D6.18 | --- | 0 | 1.72732524432905 | 1.56414170329639 | 3.80218203025436 | 3.75168443677823 | 1.53712885946484 | 1.09715564920848 | 3.03033177549914 | 1.93317612629066 | 0.0344927119461367 | 0.508344006339721 | --- | --- | --- | --- |
| WBGene00219206 | W05H12.4 | --- | 5.14502076065917 | 4.0020155441029 | 3.55625577821207 | 2.41051223419307 | 1.47583054764563 | 3.51877429894335 | 4.23443069432471 | 2.46837236026068 | -1.76605833406403 | 0.0229147730143098 | 0.467647785575674 | --- | --- | --- | --- |
| WBGene00011203 | R10E4.7 | --- | 6.51940454603552 | 8.37283073036744 | 8.38237588027138 | 9.10969894001517 | 9.09307363291604 | 8.2086506982808 | 7.75820371889145 | 8.80380775707067 | 1.04560403817922 | 0.0286250580252081 | 0.487183645467303 | --- | --- | --- | --- |
| WBGene00018970 | clec-68 | extracellular region | 2.75074548289308 | 2.15920951431739 | 2.97261059539981 | 0 | 0 | 0 | 2.62752186420343 | 0 | -2.62752186420343 | 0.00735475568041935 | 0.360501525676194 | --- | GO:0005576//extracellular region | --- | --- |
| WBGene00009414 | F35E2.7 | --- | 4.89011792583899 | 1.72732524432905 | 5.17896457061937 | 0 | 3.17256232570554 | 2.26433442226316 | 3.9321359135958 | 1.81229891598957 | -2.11983699760624 | 0.0191790867458076 | 0.445783970494014 | --- | --- | --- | --- |
| WBGene00012682 | asp-16 | --- | 6.76722058099772 | 5.98642478263922 | 6.17853654546838 | 4.62885921890615 | 4.92570108603844 | 6.07843321717785 | 6.31072730303511 | 5.21099784070748 | -1.09972946232763 | 0.0268786772861854 | 0.483702049754328 | --- | --- | --- | --- |
| WBGene00017411 | F13A2.2 | --- | 6.77981544755181 | 5.69613129357992 | 6.09839065878567 | 4.42653282953089 | 4.09025515219686 | 6.03724082571402 | 6.19144579997247 | 4.85134293581392 | -1.34010286415854 | 0.0328792436872766 | 0.50635056273794 | --- | --- | --- | --- |
| WBGene00021384 | Y37F4.3 | --- | 2.52995591386304 | 1.72732524432905 | 3.14209662450744 | 0 | 0 | 0 | 2.46645926089984 | 0 | -2.46645926089984 | 0.011774600328228 | 0.406223711323865 | --- | --- | --- | --- |
| WBGene00001556 | gcy-36 | --- | 7.82276520589355 | 7.10178542024646 | 6.58368818210817 | 5.46487427104472 | 5.61761601222003 | 6.68321576220111 | 7.16941293608273 | 5.92190201515529 | -1.24751092092744 | 0.00367697900008799 | 0.283032734483984 | --- | --- | --- | ko04540//Gap junction,ko04730//Long-term depression,ko04970//Salivary secretion,ko04713//Circadian entrainment,ko04270//Vascular smooth muscle contraction,ko00230//Purine metabolism |
| WBGene00008551 | lgc-36 | --- | 4.4602430554365 | 5.05986881279458 | 4.55493725636714 | 4.1911300965271 | 3.02250078652137 | 3.25697082212889 | 4.69168304153274 | 3.49020056839245 | -1.20148247314029 | 0.0409948210142688 | 0.525699473916166 | --- | --- | --- | ko05033//Nicotine addiction,ko04723//Retrograde endocannabinoid signaling,ko04080//Neuroactive ligand-receptor interaction,ko05032//Morphine addiction,ko04727//GABAergic synapse |
| WBGene00017594 | F19C7.4 | --- | 5.48976675508523 | 4.44626965381121 | 3.877656608957 | 5.35504563204723 | 6.24451347054481 | 5.57298085597843 | 4.60456433928448 | 5.72417998619016 | 1.11961564690568 | 0.0439812250418907 | 0.536801723800692 | --- | --- | --- | --- |
| WBGene00022506 | ZC21.6 | --- | 6.25651133391332 | 5.05986881279458 | 4.92387316844588 | 3.80218203025436 | 4.36427892765617 | 4.68527453225857 | 5.41341777171793 | 4.28391183005637 | -1.12950594166156 | 0.0186183580743318 | 0.4442059641914 | --- | --- | --- | --- |
| WBGene00000193 | arl-6 | --- | 6.25651133391332 | 5.81934138386308 | 5.55427754328056 | 4.42653282953089 | 4.30049650016008 | 5.54386476620498 | 5.87671008701899 | 4.75696469863198 | -1.119745388387 | 0.0236141797268822 | 0.472571062480819 | --- | --- | --- | --- |
| WBGene00004777 | ser-2 | --- | 8.09887904670833 | 7.36711319849846 | 7.91129834010876 | 6.51805105660933 | 5.90177025743039 | 7.81059878859118 | 7.79243019510518 | 6.74347336754363 | -1.04895682756155 | 0.0450380962602128 | 0.540216053574397 | --- | --- | --- | --- |
| WBGene00017860 | F27C1.13 | --- | 5.48976675508523 | 3.51097475443316 | 4.92387316844588 | 3.80218203025436 | 3.02250078652137 | 3.74028684181311 | 4.64153822598809 | 3.52165655286295 | -1.11988167312514 | 0.0354625711148676 | 0.510495966447296 | --- | --- | --- | --- |
| WBGene00019041 | fbxc-22 | --- | 4.84288501226145 | 5.66361142672134 | 5.25486952968103 | 3.90968186346369 | 4.59446996242318 | 4.17946905791092 | 5.25378865622127 | 4.2278736279326 | -1.02591502828868 | 0.0273778664175152 | 0.483881533032992 | --- | --- | --- | --- |
| WBGene00010801 | M01B2.8 | --- | 0 | 1.72732524432905 | 2.29690683561209 | 3.55968471353959 | 3.17256232570554 | 3.39380306024938 | 1.34141069331371 | 3.37535003316484 | 2.03393933985112 | 0.0432050091738016 | 0.531447402532409 | --- | --- | --- | --- |
| WBGene00007190 | rmd-3 | --- | 6.59261857699403 | 8.02792001196414 | 7.83297716574773 | 8.89756876115275 | 9.03301498827445 | 7.86458213045043 | 7.48450525156863 | 8.59838862662588 | 1.11388337505724 | 0.00409550240687178 | 0.292928312393939 | --- | --- | --- | --- |
| WBGene00008724 | F13A7.1 | --- | 6.42634220774996 | 8.40761226858653 | 8.05623436407289 | 9.02257541002533 | 8.96264070842549 | 8.06745581294836 | 7.63006294680313 | 8.68422397713306 | 1.05416103032993 | 0.0277831471527763 | 0.484378742276146 | --- | --- | --- | --- |
| WBGene00012603 | nspe-6 | --- | 7.35198487648021 | 6.33510644911722 | 6.52358094594995 | 4.2739395423331 | 4.16379285097932 | 6.33790500975221 | 6.73689075718246 | 4.92521246768821 | -1.81167828949425 | 0.00687332010978305 | 0.351150021288132 | --- | --- | --- | --- |
| WBGene00005755 | srw-8 | --- | 4.98018335957823 | 3.18411645795122 | 3.14209662450744 | 0 | 0 | 3.1057877649464 | 3.7687988140123 | 1.03526258831547 | -2.73353622569683 | 0.0325340830487437 | 0.506076459183572 | --- | --- | --- | --- |
| WBGene00009238 | selt-1.2 | --- | 3.63938229466363 | 4.59163971678492 | 4.6137781794739 | 6.39395050920276 | 6.02528886948196 | 4.88422935266298 | 4.28160006364082 | 5.76782291044923 | 1.48622284680842 | 0.002197268873784 | 0.260973196997361 | --- | --- | --- | --- |
| WBGene00004141 | pqn-57 | --- | 5.48976675508523 | 6.86488790972594 | 0 | 0 | 0 | 0 | 4.11821822160372 | 0 | -4.11821822160372 | 0.0355036378440693 | 0.510495966447296 | --- | --- | --- | --- |
| WBGene00044376 | Y49F6B.12 | --- | 4.18522789582735 | 4.44626965381121 | 3.4309644979598 | 2.41051223419307 | 1.8766272871374 | 2.93688715379468 | 4.02082068253279 | 2.40800889170838 | -1.6128117908244 | 0.0194757339835476 | 0.44799497141505 | --- | --- | --- | --- |
| WBGene00005651 | srp-10 | --- | 3.63938229466363 | 4.0020155441029 | 3.4309644979598 | 5.06068424222558 | 5.32882278978024 | 4.39044193885787 | 3.69078744557544 | 4.92664965695456 | 1.23586221137912 | 0.0140726828630344 | 0.423697561559018 | --- | --- | --- | ko05146//Amoebiasis |
| WBGene00004054 | AC8.1 | --- | 5.66293518117086 | 4.36769507785014 | 4.14033939827645 | 5.88934000536619 | 5.71868163195237 | 6.46759783247014 | 4.72365655243248 | 6.02520648992957 | 1.30154993749708 | 0.0155322530202817 | 0.431837560479778 | --- | --- | --- | ko03410//Base excision repair |
| WBGene00021971 | Y57G7A.8 | --- | 4.39624644935057 | 4.36769507785014 | 4.05798066360249 | 5.66224116836635 | 5.42168700451196 | 4.92995836187629 | 4.27397406360107 | 5.33796217825153 | 1.06398811465047 | 0.0145326577873408 | 0.425396261145933 | --- | --- | --- | --- |
| WBGene00000684 | col-110 | --- | 4.10742315433226 | 5.84856721965967 | 4.77714121903117 | 6.96697407566825 | 7.17398384902016 | 4.88422935266298 | 4.91104386434103 | 6.34172909245046 | 1.43068522810943 | 0.00786440387537959 | 0.364632838670264 | --- | --- | --- | --- |
| WBGene00044174 | fipr-5 | --- | 4.58030189189444 | 5.29033980728046 | 5.92336234371516 | 7.10907865766053 | 6.75336697905173 | 5.45281553423703 | 5.26466801429669 | 6.43842039031643 | 1.17375237601974 | 0.0118101177455481 | 0.406970273664159 | --- | --- | --- | --- |
| WBGene00014898 | Y45F3A.6 | --- | 1.95058941391142 | 1.10806728817932 | 4.42952628971284 | 0 | 1.47583054764563 | 0.964274272708593 | 2.49606099726786 | 0.813368273451408 | -1.68269272381645 | 0.0394100783753129 | 0.525042823265642 | --- | --- | --- | --- |
| WBGene00005064 | sra-38 | integral component of membrane | 2.52995591386304 | 2.76082432963029 | 3.29374844389101 | 0 | 0 | 0 | 2.86150956246145 | 0 | -2.86150956246145 | 0.00273420115903947 | 0.26549817545971 | --- | GO:0031224//intrinsic component of membrane,GO:0016020//membrane,GO:0044425//membrane part,GO:0016021//integral component of membrane | --- | --- |
| WBGene00020533 | T16A1.2 | --- | 4.84288501226145 | 4.36769507785014 | 5.96915006714066 | 6.6479261154587 | 6.5626309765485 | 5.83576814832265 | 5.05991005241742 | 6.34877508010995 | 1.28886502769253 | 0.0117147614660307 | 0.406223711323865 | --- | --- | --- | --- |
| WBGene00001988 | hot-3 | --- | 7.25608771131673 | 6.85051336114039 | 6.89965207924803 | 5.27688809962558 | 5.64355387907325 | 6.92822095189483 | 7.00208438390172 | 5.94955431019789 | -1.05253007370383 | 0.0372119344260427 | 0.517220675704335 | --- | --- | --- | --- |
| WBGene00015033 | B0207.10 | --- | 3.63938229466363 | 4.28459328350387 | 2.55888921229042 | 0 | 2.66546365057537 | 0 | 3.49428826348597 | 0.888487883525123 | -2.60580037996085 | 0.0332748520914695 | 0.506840182950125 | --- | --- | --- | --- |
| WBGene00012266 | W04G5.5 | --- | 6.00083678684673 | 5.05986881279458 | 6.1188483874836 | 4.00972402417049 | 4.59446996242318 | 5.38875843712698 | 5.7265179957083 | 4.66431747457355 | -1.06220052113475 | 0.0349403001212534 | 0.508344006339721 | --- | --- | --- | --- |
| WBGene00012268 | W04G5.8 | --- | 7.62056553128369 | 7.21832157027186 | 7.33516927738697 | 5.42918571762883 | 6.44955651538865 | 6.60079399830084 | 7.39135212631417 | 6.15984541043944 | -1.23150671587473 | 0.000606604133962486 | 0.150821760167858 | --- | --- | --- | --- |
| WBGene00020737 | T23F2.4 | integral component of membrane | 8.01080259082833 | 6.22797738828682 | 7.42004720220612 | 5.15108941575715 | 5.71868163195237 | 7.11797406558528 | 7.21960906044042 | 5.99591503776493 | -1.22369402267549 | 0.0272385042123502 | 0.483881533032992 | --- | GO:0031224//intrinsic component of membrane,GO:0016020//membrane,GO:0044425//membrane part,GO:0016021//integral component of membrane | --- | --- |
| WBGene00049360 | 21ur-3265 | --- | 0.967248346793504 | 1.10806728817932 | 0.984412639926697 | 3.42121237360488 | 3.93087903020093 | 4.01946253970874 | 1.01990942496651 | 3.79051798117152 | 2.77060855620501 | 0.00110526350503622 | 0.191786108196373 | --- | --- | --- | --- |
| WBGene00199858 | C40H5.15 | --- | 3.93796774921411 | 2.76082432963029 | 2.78052971426822 | 1.05599915505846 | 2.18994296053331 | 0 | 3.15977393103754 | 1.08198070519726 | -2.07779322584028 | 0.0431728380690335 | 0.531404546570963 | --- | --- | --- | --- |
| WBGene00008818 | srz-101 | --- | 3.26242902632363 | 3.18411645795122 | 1.97655534744632 | 0 | 0 | 1.53712885946484 | 2.80770027724039 | 0.51237628648828 | -2.29532399075211 | 0.0343803799252855 | 0.508344006339721 | --- | --- | --- | --- |
| WBGene00008811 | F14F7.4 | --- | 5.45877119202772 | 6.43482743968778 | 7.14878263880242 | 7.7818121470775 | 7.7704018683099 | 7.08803150219802 | 6.34746042350597 | 7.54674850586181 | 1.19928808235583 | 0.00706777843570806 | 0.354901716827292 | --- | --- | --- | --- |
| WBGene00017944 | F31D5.1 | integral component of membrane | 2.75074548289308 | 4.28459328350387 | 4.67031299909947 | 6.1526954813069 | 5.94412908681556 | 4.63093224703473 | 3.90188392183214 | 5.57591893838573 | 1.67403501655359 | 0.00566012612182814 | 0.329331743100417 | --- | GO:0031224//intrinsic component of membrane,GO:0016020//membrane,GO:0044425//membrane part,GO:0016021//integral component of membrane | --- | --- |
| WBGene00014247 | ZK1307.4 | --- | 5.71632795609053 | 7.73110671125015 | 7.45264782179216 | 8.54005142551681 | 8.22158720488575 | 7.18551410430327 | 6.96669416304428 | 7.98238424490194 | 1.01569008185766 | 0.0498396890210454 | 0.553669076757121 | --- | --- | --- | --- |
| WBGene00220003 | K02G10.16 | --- | 0.967248346793504 | 5.69613129357992 | 4.87660150221946 | 1.05599915505846 | 0.918934670839788 | 0.964274272708593 | 3.84666038086429 | 0.979736032868947 | -2.86692434799535 | 0.000176868282860496 | 0.0836558450787751 | --- | --- | --- | --- |
| WBGene00010261 | srh-308 | --- | 0 | 0 | 0 | 3.68602244576112 | 2.44720705578603 | 1.94607408114065 | 0 | 2.69310119422927 | 2.69310119422927 | 0.00460875720790828 | 0.30021454588333 | --- | --- | --- | --- |
| WBGene00006654 | ttx-3 | --- | 6.87680156603779 | 6.20556278983239 | 6.23585304165929 | 3.90968186346369 | 5.36044637087392 | 5.71034959579942 | 6.43940579917649 | 4.99349261004568 | -1.44591318913081 | 0.00474840365098148 | 0.306553778601445 | --- | --- | --- | --- |
| WBGene00020829 | T26C11.3 | --- | 3.63938229466363 | 2.98793946356524 | 2.97261059539981 | 4.42653282953089 | 4.5402731567933 | 4.57446264870628 | 3.19997745120956 | 4.51375621167682 | 1.31377876046726 | 0.0227837314367429 | 0.467515398503806 | --- | --- | --- | --- |
| WBGene00220230 | Y111B2A.36 | --- | 1.54112629318484 | 1.72732524432905 | 1.56414170329639 | 1.65915520751611 | 1.47583054764563 | 6.99428419500633 | 1.61086441360343 | 3.37642331672269 | 1.76555890311926 | 0.0126697176057096 | 0.414590197463799 | --- | --- | --- | --- |
| WBGene00007249 | C01G12.9 | --- | 3.63938229466363 | 5.81934138386308 | 5.72412922464358 | 6.53493936415773 | 7.15607558824824 | 6.03724082571402 | 5.06095096772343 | 6.57608525937333 | 1.5151342916499 | 0.0115385032402231 | 0.403297505982768 | --- | --- | --- | --- |
| WBGene00004240 | puf-4 | --- | 0 | 2.15920951431739 | 2.55888921229042 | 3.42121237360488 | 5.53688125899629 | 3.83947041366737 | 1.57269957553594 | 4.26585468208951 | 2.69315510655358 | 0.00174702815947836 | 0.242143904439996 | --- | --- | --- | --- |
| WBGene00268202 | T27B7.9 | --- | 0 | 0 | 0 | 3.42121237360488 | 3.17256232570554 | 1.53712885946484 | 0 | 2.71030118625842 | 2.71030118625842 | 0.00426760956858703 | 0.296249692756642 | --- | --- | --- | --- |
| WBGene00016053 | ptp-5.1 | protein tyrosine phosphatase activity | 5.10554323900828 | 7.2839768953653 | 7.14878263880242 | 8.18632924607493 | 7.9732205351209 | 6.87075901336379 | 6.51276759105867 | 7.67676959818654 | 1.16400200712787 | 0.0411862289312834 | 0.525699473916166 | GO:0044260//cellular macromolecule metabolic process,GO:0016311//dephosphorylation,GO:0043412//macromolecule modification,GO:0009987//cellular process,GO:0006796//phosphate-containing compound metabolic process,GO:0006793//phosphorus metabolic process,GO:0006470//protein dephosphorylation,GO:0035335//peptidyl-tyrosine dephosphorylation,GO:0019538//protein metabolic process,GO:0036211//protein modification process,GO:0071704//organic substance metabolic process,GO:0043170//macromolecule metabolic process,GO:0006464//cellular protein modification process,GO:0044238//primary metabolic process,GO:0044237//cellular metabolic process,GO:0044267//cellular protein metabolic process,GO:0008152//metabolic process | --- | GO:0016791//phosphatase activity,GO:0004721//phosphoprotein phosphatase activity,GO:0042578//phosphoric ester hydrolase activity,GO:0016787//hydrolase activity,GO:0004725//protein tyrosine phosphatase activity,GO:0016788//hydrolase activity, acting on ester bonds,GO:0003824//catalytic activity | --- |
| WBGene00021447 | Y39A3CR.8 | --- | 2.52995591386304 | 2.76082432963029 | 2.55888921229042 | 3.68602244576112 | 4.69711331580184 | 4.68527453225857 | 2.61655648526125 | 4.35613676460718 | 1.73958027934593 | 0.00440100487354948 | 0.296476229896414 | --- | --- | --- | --- |
| WBGene00009309 | F32A11.4 | --- | 5.97915703948899 | 5.00900241559391 | 5.326979702477 | 2.41051223419307 | 3.93087903020093 | 4.4544259627836 | 5.43837971918663 | 3.59860574239253 | -1.8397739767941 | 0.00300440869093101 | 0.271870574624068 | --- | --- | --- | --- |
| WBGene00010547 | K03H1.12 | --- | 4.93585336098973 | 6.66582356161052 | 6.7238359070583 | 7.45798442508994 | 7.75263611819355 | 6.7611821291931 | 6.10850427655285 | 7.32393422415886 | 1.21542994760601 | 0.0151257514910664 | 0.431215695253424 | --- | --- | --- | --- |
| WBGene00022621 | ZC477.7 | --- | 5.88902546675387 | 7.82231927066138 | 7.83915515953562 | 8.60945916861219 | 8.58164607498035 | 7.48262484952948 | 7.18349996565029 | 8.22457669770734 | 1.04107673205705 | 0.0442877178676537 | 0.53772899875954 | --- | --- | --- | --- |
| WBGene00000433 | ceh-8 | --- | 6.76722058099772 | 6.22797738828682 | 5.61314483753485 | 3.68602244576112 | 4.59446996242318 | 5.60152093819677 | 6.20278093560646 | 4.62733778212702 | -1.57544315347944 | 0.00484824209208859 | 0.309075433370647 | --- | --- | --- | --- |
| WBGene00018049 | clec-138 | --- | 0 | 0 | 1.56414170329639 | 3.26802428728875 | 2.66546365057537 | 2.52491846837501 | 0.521380567765463 | 2.81946880207971 | 2.29808823431425 | 0.0405510039487448 | 0.525699473916166 | --- | --- | --- | --- |
| WBGene00044235 | C06A1.7 | --- | 0 | 0 | 1.56414170329639 | 3.09661667470929 | 3.6529845535538 | 1.53712885946484 | 0.521380567765463 | 2.76224336257598 | 2.24086279481051 | 0.0348581292710049 | 0.508344006339721 | --- | --- | --- | --- |
| WBGene00022842 | ZK994.6 | --- | 6.37745857411846 | 5.66361142672134 | 5.49290568806793 | 3.90968186346369 | 3.93087903020093 | 5.42114248134784 | 5.84465856296924 | 4.42056779167082 | -1.42409077129842 | 0.0122981511911293 | 0.412740168948743 | --- | --- | --- | --- |
| WBGene00005428 | srh-218 | integral component of membrane | 0 | 0 | 0 | 3.09661667470929 | 2.44720705578603 | 3.25697082212889 | 0 | 2.93359818420807 | 2.93359818420807 | 0.00226731620634427 | 0.260973196997361 | --- | GO:0031224//intrinsic component of membrane,GO:0016020//membrane,GO:0044425//membrane part,GO:0016021//integral component of membrane | --- | --- |
| WBGene00018365 | tpst-2 | --- | 5.25735820612055 | 5.63034159019379 | 5.92336234371516 | 3.09661667470929 | 4.01276678014068 | 4.25327487984212 | 5.60368738000983 | 3.7875527782307 | -1.81613460177914 | 0.000222835003131148 | 0.0931312932373682 | --- | --- | --- | --- |
| WBGene00013856 | ZC168.2 | extracellular region | 5.57894761177708 | 5.41279526437416 | 4.05798066360249 | 2.67712217824935 | 3.02250078652137 | 4.01946253970874 | 5.01657451325124 | 3.23969516815982 | -1.77687934509142 | 0.00330604737251095 | 0.277192646374062 | --- | GO:0005576//extracellular region | --- | --- |
| WBGene00018822 | F54D12.9 | --- | 0 | 0 | 0 | 4.1911300965271 | 2.18994296053331 | 0 | 0 | 2.12702435235347 | 2.12702435235347 | 0.0131315862101406 | 0.421187542609031 | --- | --- | --- | --- |
| WBGene00206390 | F14F7.7 | --- | 2.75074548289308 | 2.98793946356524 | 2.55888921229042 | 3.68602244576112 | 4.83856241132295 | 3.74028684181311 | 2.76585805291625 | 4.08829056629906 | 1.32243251338281 | 0.0315025796251445 | 0.502642069752007 | --- | --- | --- | --- |
| WBGene00013886 | ZC412.5 | --- | 4.58030189189444 | 5.489014266081 | 5.09884313101069 | 6.44844858827206 | 6.27801822751416 | 5.60152093819677 | 5.05605309632871 | 6.10932925132766 | 1.05327615499895 | 0.00938985442938394 | 0.377752447714155 | --- | --- | --- | --- |
| WBGene00268192 | ZK697.17 | --- | 4.84288501226145 | 3.65026690971359 | 4.29216665132285 | 2.67712217824935 | 0 | 1.94607408114065 | 4.26177285776596 | 1.54106541979667 | -2.7207074379693 | 0.00174426350546768 | 0.242143904439996 | --- | --- | --- | --- |
| WBGene00005599 | srj-11 | --- | 3.52433812248388 | 0 | 2.29690683561209 | 0 | 0 | 0 | 1.94041498603199 | 0 | -1.94041498603199 | 0.0346792380100236 | 0.508344006339721 | --- | --- | --- | --- |
| WBGene00016107 | C25G6.1 | --- | 6.76722058099772 | 6.13615055724891 | 6.13902007258417 | 5.01326446423341 | 4.79293645953538 | 5.9948376372027 | 6.3474637369436 | 5.26701285365716 | -1.08045088328644 | 0.0153560763946931 | 0.431837560479778 | --- | --- | --- | --- |
| WBGene00019828 | R02E12.4 | --- | 6.36078889106518 | 6.18279444106444 | 5.99151026546355 | 4.80626946887653 | 4.42536048117021 | 5.73632073444849 | 6.17836453253106 | 4.98931689483174 | -1.18904763769931 | 0.00940041004659367 | 0.377752447714155 | --- | --- | --- | --- |
| WBGene00022731 | ZK402.5 | --- | 2.52995591386304 | 0 | 0 | 4.1911300965271 | 3.93087903020093 | 3.51877429894335 | 0.843318637954347 | 3.88026114189046 | 3.03694250393611 | 0.00604943668314542 | 0.334814544247929 | --- | --- | --- | --- |
| WBGene00219680 | linc-35 | --- | 5.02319172990672 | 5.24709728418621 | 3.97063416719798 | 3.42121237360488 | 2.66546365057537 | 3.74028684181311 | 4.74697439376364 | 3.27565428866445 | -1.47132010509918 | 0.0109050511772804 | 0.393319628595338 | --- | --- | --- | --- |
| WBGene00014182 | ZK1025.2 | --- | 2.75074548289308 | 5.10900267968443 | 5.29137510420009 | 6.25816151730039 | 6.52125962818014 | 5.28700207279648 | 4.38370775559253 | 6.022141072759 | 1.63843331716647 | 0.0185851685074535 | 0.4442059641914 | --- | --- | --- | --- |
| WBGene00014185 | ZK1025.5 | --- | 0 | 0 | 0 | 2.41051223419307 | 2.66546365057537 | 2.93688715379468 | 0 | 2.67095434618771 | 2.67095434618771 | 0.00730202717235116 | 0.360491492978447 | --- | --- | --- | --- |
| WBGene00014188 | ZK1025.8 | --- | 2.75074548289308 | 5.10900267968443 | 5.29137510420009 | 6.25816151730039 | 6.52125962818014 | 5.28700207279648 | 4.38370775559253 | 6.022141072759 | 1.63843331716647 | 0.0185851685074535 | 0.4442059641914 | --- | --- | --- | --- |
| WBGene00015854 | C16C8.17 | --- | 0 | 0 | 0 | 0 | 3.17256232570554 | 3.1057877649464 | 0 | 2.09278336355065 | 2.09278336355065 | 0.0276424949378777 | 0.483949948688515 | --- | --- | --- | --- |
| WBGene00017363 | F10G2.2 | --- | 6.77981544755181 | 6.94825138398901 | 7.43643959467738 | 5.990753954614 | 5.2295672819676 | 6.78626189255513 | 7.05483547540607 | 6.00219437637891 | -1.05264109902716 | 0.0190598243218989 | 0.445717183604214 | --- | --- | --- | --- |
| WBGene00021494 | Y40B10B.1 | extracellular exosome | 7.74139348060881 | 6.33510644911722 | 6.82690916902505 | 5.15108941575715 | 4.42536048117021 | 6.79864015199436 | 6.96780303291703 | 5.45836334964057 | -1.50943968327645 | 0.0213484686187007 | 0.460882445614097 | --- | GO:1903561//extracellular vesicle,GO:0043226//organelle,GO:0031982//vesicle,GO:0005576//extracellular region,GO:0070062//extracellular exosome,GO:0043227//membrane-bounded organelle,GO:0043230//extracellular organelle,GO:0044421//extracellular region part | --- | --- |
| WBGene00194932 | F54F7.11 | --- | 1.54112629318484 | 0 | 1.56414170329639 | 2.41051223419307 | 4.30049650016008 | 2.26433442226316 | 1.03508933216041 | 2.99178105220544 | 1.95669172004503 | 0.0323489648686169 | 0.506076459183572 | --- | --- | --- | --- |
| WBGene00001769 | gst-21 | glutathione transferase activity | 4.63678128998955 | 4.95627682518266 | 5.42880664758102 | 4.35225271272185 | 3.3084753592717 | 3.74028684181311 | 5.00728825425108 | 3.80033830460222 | -1.20694994964886 | 0.0212353913948458 | 0.460597524152259 | --- | --- | GO:0016765//transferase activity, transferring alkyl or aryl (other than methyl) groups,GO:0004364//glutathione transferase activity,GO:0003824//catalytic activity,GO:0016740//transferase activity | ko05204//Chemical carcinogenesis,ko00980//Metabolism of xenobiotics by cytochrome P450,ko00480//Glutathione metabolism,ko00982//Drug metabolism - cytochrome P450 |
| WBGene00018517 | F46G11.4 | --- | 7.37706902229393 | 6.69829415935076 | 6.94618642101397 | 5.7516438052487 | 5.66903363932878 | 6.48301938949434 | 7.00718320088622 | 5.96789894469061 | -1.03928425619561 | 0.00292799126505361 | 0.271870574624068 | --- | --- | --- | --- |
| WBGene00005511 | srh-307 | --- | 3.26242902632363 | 3.77728662599979 | 2.29690683561209 | 1.65915520751611 | 1.8766272871374 | 0 | 3.1122074959785 | 1.1785941648845 | -1.933613331094 | 0.0465903204864663 | 0.545102168225596 | --- | --- | --- | --- |
| WBGene00219490 | Y116A8B.98 | --- | 3.52433812248388 | 2.15920951431739 | 3.29374844389101 | 0 | 1.47583054764563 | 0.964274272708593 | 2.99243202689743 | 0.813368273451408 | -2.17906375344602 | 0.0248076113200884 | 0.479065382160599 | --- | --- | --- | --- |
| WBGene00235129 | ZC518.8 | --- | 2.26916251373634 | 2.98793946356524 | 1.97655534744632 | 0 | 0 | 0.964274272708593 | 2.4112191082493 | 0.321424757569531 | -2.08979435067977 | 0.0416025866205553 | 0.52685954148577 | --- | --- | --- | --- |
| WBGene00004107 | pqn-16 | --- | 6.08443773604644 | 5.10900267968443 | 5.6417015684825 | 3.42121237360488 | 4.92570108603844 | 5.05904292354522 | 5.61171399473779 | 4.46865212772951 | -1.14306186700828 | 0.0421697651793199 | 0.527676068006671 | --- | --- | --- | --- |
| WBGene00017114 | E03H12.7 | --- | 4.98018335957823 | 7.3052144046413 | 7.50798419410419 | 8.23881066753628 | 8.16450812126082 | 7.39566607051229 | 6.59779398610791 | 7.93299495310313 | 1.33520096699522 | 0.0340817586038559 | 0.508344006339721 | --- | --- | --- | --- |
| WBGene00007638 | C17D12.3 | --- | 9.53187586302052 | 8.46062919241572 | 8.96589651893642 | 7.50159302436696 | 7.50633439170207 | 8.85260652851722 | 8.98613385812422 | 7.95351131486208 | -1.03262254326214 | 0.0100173837821497 | 0.382082839294636 | --- | --- | --- | --- |
| WBGene00077664 | C43D7.11 | --- | 1.54112629318484 | 3.51097475443316 | 0 | 4.86086841734319 | 3.43267949213248 | 3.74028684181311 | 1.68403368253933 | 4.01127825042959 | 2.32724456789026 | 0.0420429487907295 | 0.527676068006671 | --- | --- | --- | --- |
| WBGene00005724 | srv-13 | --- | 5.3276669009305 | 4.95627682518266 | 4.49359406380416 | 0 | 3.3084753592717 | 4.17946905791092 | 4.92584592997244 | 2.49598147239421 | -2.42986445757823 | 0.0246476186425086 | 0.478957595700876 | --- | --- | --- | --- |
| WBGene00005725 | srv-14 | --- | 5.10554323900828 | 3.35678254004612 | 4.42952628971284 | 0 | 2.18994296053331 | 3.83947041366737 | 4.29728402292241 | 2.00980445806689 | -2.28747956485552 | 0.0446297746202857 | 0.539253457247581 | --- | --- | --- | --- |
| WBGene00012183 | W01D2.6 | --- | 3.84514002926505 | 3.51097475443316 | 4.21824936537107 | 2.41051223419307 | 1.47583054764563 | 1.53712885946484 | 3.85812138302309 | 1.80782388043451 | -2.05029750258858 | 0.00629066773078003 | 0.341618205935416 | --- | --- | --- | --- |
| WBGene00050931 | F59B2.14 | --- | 3.39931885361132 | 2.76082432963029 | 0 | 0 | 0 | 0 | 2.05338106108054 | 0 | -2.05338106108054 | 0.0285072440907419 | 0.487164879347905 | --- | --- | --- | --- |
| WBGene00021083 | W08E12.2 | --- | 0 | 0 | 0 | 3.26802428728875 | 0 | 3.1057877649464 | 0 | 2.12460401741172 | 2.12460401741172 | 0.0260064561617233 | 0.481198798674787 | --- | --- | --- | --- |
| WBGene00011161 | chil-18 | --- | 4.89011792583899 | 5.29033980728046 | 3.97063416719798 | 3.42121237360488 | 3.02250078652137 | 3.74028684181311 | 4.71703063343914 | 3.39466666731312 | -1.32236396612602 | 0.015605383675301 | 0.431837560479778 | --- | --- | --- | --- |
| WBGene00023172 | Y23H5A.8 | --- | 3.1111756519858 | 3.51097475443316 | 3.67153022453517 | 4.42653282953089 | 4.96737304705394 | 4.32348797408789 | 3.43122687698471 | 4.57246461689091 | 1.1412377399062 | 0.034933817436007 | 0.508344006339721 | --- | --- | --- | --- |
| WBGene00022229 | Y73B6A.2 | --- | 4.18522789582735 | 4.36769507785014 | 5.6417015684825 | 6.06239410914001 | 6.10212509890849 | 5.71034959579942 | 4.73154151405333 | 5.95828960128264 | 1.22674808722931 | 0.0198341198959992 | 0.451931286674574 | --- | --- | --- | --- |
| WBGene00021949 | clec-164 | --- | 2.26916251373634 | 3.65026690971359 | 4.21824936537107 | 2.67712217824935 | 0 | 0 | 3.37922626294033 | 0.89237405941645 | -2.48685220352388 | 0.0399436576484776 | 0.525699473916166 | --- | --- | --- | --- |
| WBGene00021947 | srt-23 | integral component of membrane | 2.26916251373634 | 0 | 3.97063416719798 | 0 | 0 | 0 | 2.07993222697811 | 0 | -2.07993222697811 | 0.0158886591788111 | 0.433452308356085 | --- | GO:0031224//intrinsic component of membrane,GO:0016020//membrane,GO:0044425//membrane part,GO:0016021//integral component of membrane | --- | --- |
| WBGene00019887 | R05D8.11 | --- | 4.58030189189444 | 4.19641051469006 | 4.6137781794739 | 1.65915520751611 | 2.18994296053331 | 3.1057877649464 | 4.46349686201947 | 2.31829531099861 | -2.14520155102086 | 0.00181251786109416 | 0.243287789015961 | --- | --- | --- | --- |

| Gene\_ID | Symbol | description | op50\_1 | op50\_2 | op50\_3 | S20mg\_1 | S20mg\_2 | S20mg\_3 | G1 | G2 | G2\_vs\_G1\_log2FoldChange | G2\_vs\_G1\_Pvalue | G2\_vs\_G1\_FDR | biological\_process | cellular\_component | molecular\_function | KEGG\_pathway |
| --- | --- | --- | --- | --- | --- | --- | --- | --- | --- | --- | --- | --- | --- | --- | --- | --- | --- |
| WBGene00014161 | arrd-5 | --- | 4.69113265039224 | 4.59163971678492 | 5.09884313101069 | 0 | 4.01276678014068 | 3.6337778023327 | 4.79387183272928 | 2.54884819415779 | -2.24502363857149 | 0.0427566498311083 | 0.529320350796076 | --- | --- | --- | --- |
| WBGene00008529 | F02E9.3 | --- | 5.48976675508523 | 6.97500206945319 | 6.95758920437985 | 8.12630528602139 | 7.99353174469564 | 6.66980166461679 | 6.47411934297276 | 7.59654623177794 | 1.12242688880518 | 0.0123667749936071 | 0.413425244614386 | --- | --- | --- | --- |
| WBGene00012372 | W09H1.1 | --- | 7.21029768889039 | 6.77641041662129 | 7.20726960578539 | 5.80830885368026 | 5.32882278978024 | 6.32084188278136 | 7.06465923709902 | 5.81932450874729 | -1.24533472835174 | 0.000339354826826513 | 0.115716049961483 | --- | --- | --- | --- |
| WBGene00016619 | C43H6.6 | --- | 7.70883252812198 | 6.79153863525114 | 7.05635084982705 | 5.78025451726996 | 5.81312840960928 | 6.65626167202517 | 7.18557400440006 | 6.08321486630147 | -1.10235913809859 | 0.00269037000954025 | 0.262548496842802 | --- | --- | --- | ko04977//Vitamin digestion and absorption |
| WBGene00021233 | Y19D10B.4 | --- | 0 | 0 | 0 | 3.26802428728875 | 1.47583054764563 | 2.26433442226316 | 0 | 2.33606308573251 | 2.33606308573251 | 0.0181355419170823 | 0.443675833929587 | --- | --- | --- | --- |
| WBGene00020547 | srz-7 | --- | 0 | 2.15920951431739 | 3.4309644979598 | 0 | 0 | 0 | 1.86339133742573 | 0 | -1.86339133742573 | 0.047529690000053 | 0.547450180381601 | --- | --- | --- | --- |
| WBGene00005408 | srh-195 | integral component of membrane | 6.04324273772971 | 5.20251847810575 | 6.46086016951256 | 4.86086841734319 | 4.8827895448963 | 4.73764398207432 | 5.90220712844934 | 4.8271006481046 | -1.07510648034474 | 0.00617910925759978 | 0.338641765940775 | --- | GO:0031224//intrinsic component of membrane,GO:0016020//membrane,GO:0044425//membrane part,GO:0016021//integral component of membrane | --- | --- |
| WBGene00012945 | Y47H9A.1 | --- | 9.53187586302052 | 8.46535368459049 | 8.96023912772479 | 7.51867395156043 | 7.53433209201753 | 8.85557167350941 | 8.9858228917786 | 7.96952590569579 | -1.01629698608281 | 0.0104276735123871 | 0.388300803894593 | --- | --- | --- | --- |
| WBGene00004149 | trpl-5 | --- | 1.54112629318484 | 2.76082432963029 | 1.56414170329639 | 4.42653282953089 | 4.48396063605928 | 2.52491846837501 | 1.95536410870384 | 3.81180397798839 | 1.85643986928455 | 0.0134996960071696 | 0.421564246360991 | --- | --- | --- | --- |
| WBGene00009041 | cut-3 | --- | 10.2087510697115 | 9.7061838707901 | 10.0171718238778 | 8.57313020008737 | 8.41093376145063 | 9.20853141411787 | 9.9773689214598 | 8.73086512521862 | -1.24650379624118 | 6.63083830197897e-08 | 0.000277784761722191 | --- | --- | --- | --- |
| … | … | … | … | … | … | … | … | … | … | … | … | … | … | … | … | … | … |

| Gene\_ID | Symbol | description | op50\_1 | op50\_2 | op50\_3 | S20mg\_1 | S20mg\_2 | S20mg\_3 | G1 | G2 | G2\_vs\_G1\_log2FoldChange | G2\_vs\_G1\_Pvalue | G2\_vs\_G1\_FDR | biological\_process | cellular\_component | molecular\_function | KEGG\_pathway |
| --- | --- | --- | --- | --- | --- | --- | --- | --- | --- | --- | --- | --- | --- | --- | --- | --- | --- |
| WBGene00014161 | arrd-5 | --- | 4.69113265039224 | 4.59163971678492 | 5.09884313101069 | 0 | 4.01276678014068 | 3.6337778023327 | 4.79387183272928 | 2.54884819415779 | -2.24502363857149 | 0.0427566498311083 | 0.529320350796076 | --- | --- | --- | --- |
| WBGene00008529 | F02E9.3 | --- | 5.48976675508523 | 6.97500206945319 | 6.95758920437985 | 8.12630528602139 | 7.99353174469564 | 6.66980166461679 | 6.47411934297276 | 7.59654623177794 | 1.12242688880518 | 0.0123667749936071 | 0.413425244614386 | --- | --- | --- | --- |
| WBGene00012372 | W09H1.1 | --- | 7.21029768889039 | 6.77641041662129 | 7.20726960578539 | 5.80830885368026 | 5.32882278978024 | 6.32084188278136 | 7.06465923709902 | 5.81932450874729 | -1.24533472835174 | 0.000339354826826513 | 0.115716049961483 | --- | --- | --- | --- |
| WBGene00016619 | C43H6.6 | --- | 7.70883252812198 | 6.79153863525114 | 7.05635084982705 | 5.78025451726996 | 5.81312840960928 | 6.65626167202517 | 7.18557400440006 | 6.08321486630147 | -1.10235913809859 | 0.00269037000954025 | 0.262548496842802 | --- | --- | --- | ko04977//Vitamin digestion and absorption |
| WBGene00021233 | Y19D10B.4 | --- | 0 | 0 | 0 | 3.26802428728875 | 1.47583054764563 | 2.26433442226316 | 0 | 2.33606308573251 | 2.33606308573251 | 0.0181355419170823 | 0.443675833929587 | --- | --- | --- | --- |
| WBGene00020547 | srz-7 | --- | 0 | 2.15920951431739 | 3.4309644979598 | 0 | 0 | 0 | 1.86339133742573 | 0 | -1.86339133742573 | 0.047529690000053 | 0.547450180381601 | --- | --- | --- | --- |
| WBGene00005408 | srh-195 | integral component of membrane | 6.04324273772971 | 5.20251847810575 | 6.46086016951256 | 4.86086841734319 | 4.8827895448963 | 4.73764398207432 | 5.90220712844934 | 4.8271006481046 | -1.07510648034474 | 0.00617910925759978 | 0.338641765940775 | --- | GO:0031224//intrinsic component of membrane,GO:0016020//membrane,GO:0044425//membrane part,GO:0016021//integral component of membrane | --- | --- |
| WBGene00012945 | Y47H9A.1 | --- | 9.53187586302052 | 8.46535368459049 | 8.96023912772479 | 7.51867395156043 | 7.53433209201753 | 8.85557167350941 | 8.9858228917786 | 7.96952590569579 | -1.01629698608281 | 0.0104276735123871 | 0.388300803894593 | --- | --- | --- | --- |
| WBGene00004149 | trpl-5 | --- | 1.54112629318484 | 2.76082432963029 | 1.56414170329639 | 4.42653282953089 | 4.48396063605928 | 2.52491846837501 | 1.95536410870384 | 3.81180397798839 | 1.85643986928455 | 0.0134996960071696 | 0.421564246360991 | --- | --- | --- | --- |
| WBGene00009041 | cut-3 | --- | 10.2087510697115 | 9.7061838707901 | 10.0171718238778 | 8.57313020008737 | 8.41093376145063 | 9.20853141411787 | 9.9773689214598 | 8.73086512521862 | -1.24650379624118 | 6.63083830197897e-08 | 0.000277784761722191 | --- | --- | --- | --- |
| … | … | … | … | … | … | … | … | … | … | … | … | … | … | … | … | … | … |

… … … … … … … … … … … … … … … … … …

  
  

| 表头 | 表头说明 |
| --- | --- |
| gene\_id | 基因对应基因编号 |
| symbol | 基因对应基因简称 |
| description | 基因对应基因功能描述 |
| Sample name | 经过TMM标准化后的序列数取Log2 |
| G1 | 对照样本组G1标准化后的平均表达量 |
| G2 | 实验样本组G1标准化后的平均表达量 |
| G2\_vs\_G1\_log2FoldChange | G2的平均表达量与G1的平均表达量的Log2FC，正值表示G2相对G1上调表达， 负值表示G2相对G1下调表达 |
| G2\_vs\_G1\_Pvalue | 检验统计量的未校正p-value(The uncorrected p-value of the test statistic) |
| G2\_vs\_G1\_FDR | 检验统计量的经FDR校正的p-value，q-value， FDR采用Benjamini-Hochberg correction多重检验方法计算校正p-value |
| biological\_process | GO中BP条目， 包括GO编号和GO术语， 最多显示50个，针对mRNA部分 |
| cellular\_component | GO中CC条目， 包括GO编号和GO术语， 最多显示50个，针对mRNA部分 |
| molecular\_function | GO中MF条目， 包括GO编号和GO术语， 最多显示50个，针对mRNA部分 |

  

### 两组样本间的火山图(Volcano\_Plot.png)

  

注：火山图的每个点代表一个基因，横坐标表示log2FC的值，设定∣log2FC∣≥1为显著差异基因，其中≥1的点表示实验组G2相对于对照组G1显著上调的基因，≤-1的点表示实验组G2相对于对照组G1显著下调的基因；纵坐标表示-log10(P-Value)，当P-Value≤0.05时，认为是显著差异基因。因此在火山图中，左边紫色的点表示实验组G2相对于对照组G1显著下调的基因，右边蓝色的点表示实验组G2相对于对照组G1显著上调的基因，灰色的点表示非显著差异基因。

  

### 两组样本间的散点图(Scatter\_Plot.png)

  

注：散点图的横坐标代表基因在对照组G1的表达量，纵坐标代表基因在实验组G2的表达量；因此红色的点代表实验组G2相对于对照组G1显著上调的基因，绿色的点代表实验组G2相对于对照组G1显著下调的基因，灰色点代表非显著差异基因，显著差异基因筛选条件同火山图一样。

  

### 差异表达基因聚类（\*\_heatmap.png）

针对样本组间筛选的差异表达基因，采用对基因和样本进行双向层级聚类并且用热图显示，聚类参数(Distance metric: pearson correlation; Linkage rule: Average Linkage)。 针对样本量大于等于6个, 差异基因并集采用Mfuzz聚类方法将表达模式分为10类。

结果见报告--> 4-DGE > \*\_vs\_\* >gene\_exp\_significant\_log2normalized\_count\_heatmap.pdf

  

注：聚类热图中白色代表显著差异基因的表达量均值，越红代表表达量越高，越蓝代表表达量越低，每一行代表一个基因，每一列代表一个样本。

  

关于4-DEG目录下其他有关文件的说明：

4-DGE/merge/gene\_sample\_count\*.xls：所有样本基因的count值统计表

4-DGE/merge/gene\_sample\_FPKM\_\*.xls：所有样本基因的FPKM值统计表

4-DGE/merge/gene\_sample\_log2FPKM\*.xls：所有样本基因的log2FPKM值统计表

4-DGE/merge/gene\_sample\_log2normalized\*.xls：所有样本基因的log2normalized\_count值统计表

4-DGE/merge/transcript\_sample\_count\*.xls：所有样本转录本的count值统计表

4-DGE/merge/transcript\_sample\_log2normalized\*.xls：所有样本转录本的log2normalized\_count值统计表

4-DGE/overlap/genelist\_overlap\_log2normalized\_count\_heatmap.\*：所有比较组差异基因并集结果表与heatmap绘图

  


## 5.3 表达趋势聚类

目录链接

  
  
  
  

本分析利用R包Mfuzz对基因表达谱进行表达趋势聚类分析，其核心算法是模糊c均值聚类分析，用于识别相似的基因表达谱。

### 表达趋势聚类图（Cluster.png）

  

注：表达趋势聚类图，横坐标为样本，纵坐标为基因表达量。

  
  

  


# 6. 可变剪切

## 6.1 可变性剪切统计

目录链接

  
  
  
  

针对样本的StringTie组装转录本，采用rMATS软件进行可变性剪切事件统计，可变性剪切事件主要分为5类。针对基因水平， 计算每个样本的可变性剪切事件总数，可变性剪切事件类型包括可变性剪切事件包括SE(Skipped exon外显子跳跃)， RI(Retained intron内含子延伸)，A5SS(Alternative 5' splice site外显子5端可变剪切)， A3SS(Alternative 3'splice site外显子3端可变剪切)， MXE(Mutually exclusive exons外显子互斥)。

  

### 可变性剪切示意图

### 样本的可变性剪切事件统计图（AS\_event\_stat.png）

注：图中，纵坐标为可变剪切事件类型，横坐标表示数量。

  
  


## 6.2 差异可变性剪切分析

目录链接

  
  
  
  

针对有重复或者无重复样本实验设计，采用rMATS算法来对可变性剪切事件进行表达定量。 可变剪接事件定量是通过外显子包含比率(exon inclusion level)表示， 外显子包含比率定义为支持包含可变性剪切外显子的跨外显子序列数(Inclusive Junction Count)与不支持包含可变性剪切外显子的跨外显子序列数(Skipped Junction Counts)的比率。 采用Fisher精确检验两样本组间差异可变性剪切事件的筛选方法通过计算差异显著性P值， Pvalue <= 0.05。

结果见报告--> 4-AS > \*\_vs\_\* > diff\_SE\_with\_symbol.xls

### 两样本间差异可变性剪切事件的筛选结果(diff\_\*\_with\_symbol.xls)

显示 10203050100150全文 行  
AS-S20mg\_vs\_op50
 

| GeneID | Symbol | description | chr | strand | longExonStart\_0base | longExonEnd | shortES | shortEE | flankingES | flankingEE | IncFormLen | SkipFormLen | op50\_1 | op50\_2 | op50\_3 | S20mg\_1 | S20mg\_2 | S20mg\_3 | G1\_IncLevel | G2\_IncLevel | IncLevelDifference | PValue | FDR |
| --- | --- | --- | --- | --- | --- | --- | --- | --- | --- | --- | --- | --- | --- | --- | --- | --- | --- | --- | --- | --- | --- | --- | --- |
| WBGene00016074 | dnsn-1 | --- | II | - | 410475 | 410581 | 410475 | 410575 | 410624 | 410704 | 129 | 124 | 65,104 | 51,96 | 34,95 | 62,76 | 104,128 | 54,110 | 0.328302885888271 | 0.402442982326734 | 0.074140096438463 | 1.721735e-02 | 9.431641e-02 |
| WBGene00018371 | ess-2 | nucleus | III | - | 8488343 | 8488819 | 8488343 | 8488813 | 8488940 | 8489536 | 129 | 124 | 57,70 | 49,68 | 37,63 | 85,75 | 85,93 | 46,50 | 0.406129039646366 | 0.487815539285324 | 0.0816864996389577 | 2.483565e-02 | 1.254223e-01 |
| WBGene00003229 | mex-3 | --- | I | + | 129166 | 129333 | 129175 | 129333 | 128944 | 129124 | 132 | 124 | 483,79 | 437,69 | 496,73 | 684,37 | 827,58 | 486,78 | 0.857527984527926 | 0.915567321344061 | 0.0580393368161346 | 3.719193e-08 | 2.313870e-06 |
| WBGene00009065 | F22G12.5 | --- | I | + | 13168321 | 13168707 | 13168327 | 13168707 | 13166923 | 13167566 | 129 | 124 | 33,48 | 41,39 | 44,41 | 57,27 | 50,32 | 33,32 | 0.469817621371693 | 0.596584075054126 | 0.126766453682433 | 5.922633e-03 | 4.126891e-02 |
| WBGene00009678 | magu-4 | --- | IV | + | 10011335 | 10011583 | 10011341 | 10011583 | 10011006 | 10011292 | 129 | 124 | 38,19 | 37,12 | 28,12 | 31,3 | 34,13 | 60,8 | 0.697199628800699 | 0.833512583351258 | 0.13631295455056 | 8.048879e-03 | 5.231771e-02 |
| WBGene00008274 | C53B4.4 | --- | IV | + | 8983693 | 8983848 | 8983702 | 8983848 | 8983469 | 8983647 | 132 | 124 | 187,433 | 152,237 | 193,311 | 221,170 | 240,239 | 231,318 | 0.337501279034073 | 0.472063904231675 | 0.134562625197601 | 8.297448e-14 | 2.409026e-11 |
| WBGene00000894 | dab-1 | --- | II | + | 8227552 | 8228193 | 8227558 | 8228193 | 8227056 | 8227476 | 129 | 124 | 237,498 | 238,359 | 189,429 | 298,329 | 331,387 | 230,409 | 0.33169238206502 | 0.423285553625999 | 0.0915931715609786 | 3.032679e-09 | 2.416399e-07 |
| WBGene00000464 | ceh-44 | --- | III | + | 2544834 | 2544895 | 2544840 | 2544895 | 2544361 | 2544645 | 129 | 124 | 71,45 | 78,33 | 62,40 | 100,25 | 94,20 | 88,33 | 0.632194461895327 | 0.776548967355097 | 0.14435450545977 | 4.678487e-05 | 9.261278e-04 |
| WBGene00008989 | F20G2.6 | --- | V | + | 13765225 | 13765481 | 13765228 | 13765481 | 13764566 | 13764711 | 126 | 124 | 31,33 | 8,3 | 22,6 | 6,1 | 10,1 | 13,6 | 0.588363410080896 | 0.781059947871416 | 0.19269653779052 | 4.565180e-02 | 1.884489e-01 |
| WBGene00007016 | mdt-15 | --- | III | - | 5832293 | 5832784 | 5832293 | 5832775 | 5832834 | 5833058 | 132 | 124 | 499,402 | 307,256 | 429,333 | 305,286 | 302,318 | 404,319 | 0.539316504197893 | 0.507135922330097 | -0.0321805818677955 | 3.959880e-02 | 1.741947e-01 |
| WBGene00014309 | F07H5.5 | --- | II | + | 8789502 | 8789716 | 8789562 | 8789716 | 8788960 | 8789460 | 183 | 124 | 4398,11893 | 2547,7553 | 3540,10184 | 2421,6849 | 3332,8310 | 4113,11263 | 0.193403278278837 | 0.201924863953943 | 0.00852158567510655 | 1.046397e-03 | 1.111477e-02 |
| WBGene00006795 | unc-61 | --- | V | - | 14731094 | 14731730 | 14731094 | 14731718 | 14731774 | 14732264 | 135 | 124 | 53,131 | 61,99 | 77,101 | 68,121 | 79,186 | 54,140 | 0.346414310579356 | 0.292298490658973 | -0.0541158199203828 | 4.639012e-02 | 1.893008e-01 |
| WBGene00012701 | Y39B6A.43 | --- | V | - | 18966504 | 18966862 | 18966504 | 18966853 | 18967506 | 18967619 | 132 | 124 | 33,40 | 59,20 | 58,38 | 58,21 | 90,18 | 52,31 | 0.589802130898021 | 0.72855464159812 | 0.138752510700099 | 1.021947e-03 | 1.098908e-02 |
| WBGene00001056 | dpf-3 | --- | I | + | 6839003 | 6839144 | 6839015 | 6839144 | 6838813 | 6838960 | 135 | 124 | 51,180 | 73,176 | 87,236 | 110,245 | 125,265 | 65,170 | 0.246634742279703 | 0.288372093023256 | 0.0417373507435529 | 4.558068e-02 | 1.884489e-01 |
| WBGene00000223 | atf-7 | --- | III | - | 4492651 | 4492768 | 4492651 | 4492759 | 4492809 | 4493101 | 132 | 124 | 338,349 | 240,209 | 328,302 | 313,161 | 365,222 | 298,268 | 0.497396663478908 | 0.58478130617136 | 0.0873846426924524 | 3.715833e-07 | 1.859046e-05 |
| WBGene00017699 | flcn-1 | --- | II | + | 6932202 | 6932278 | 6932211 | 6932278 | 6931614 | 6931792 | 132 | 124 | 31,125 | 21,121 | 41,144 | 54,112 | 57,115 | 18,104 | 0.183012759474386 | 0.267993566546039 | 0.0849808070716536 | 1.628255e-03 | 1.593494e-02 |
| WBGene00007008 | rfp-1 | --- | III | + | 8360724 | 8361113 | 8360733 | 8361113 | 8359975 | 8360596 | 132 | 124 | 97,76 | 75,56 | 95,84 | 140,55 | 116,75 | 74,76 | 0.537293086660175 | 0.60077519379845 | 0.0634821071382744 | 4.829886e-02 | 1.935023e-01 |
| WBGene00012458 | ash-2 | --- | II | + | 11983000 | 11983123 | 11983009 | 11983123 | 11982033 | 11982254 | 132 | 124 | 72,110 | 77,72 | 72,113 | 97,80 | 149,98 | 51,92 | 0.413059206559749 | 0.508196721311475 | 0.0951375147517262 | 1.889580e-03 | 1.808598e-02 |
| WBGene00010731 | K10C3.4 | --- | I | + | 9860496 | 9860616 | 9860502 | 9860616 | 9859271 | 9859349 | 129 | 124 | 590,33 | 386,23 | 596,33 | 457,14 | 504,19 | 454,21 | 0.944377425402962 | 0.961814653612972 | 0.0174372282100098 | 2.573571e-02 | 1.259315e-01 |
| WBGene00003620 | nhr-21 | --- | II | + | 7347570 | 7347751 | 7347576 | 7347751 | 7347373 | 7347521 | 129 | 124 | 500,20 | 250,24 | 231,11 | 210,19 | 201,13 | 298,33 | 0.944888495327756 | 0.9129292530711 | -0.0319592422566561 | 9.883842e-03 | 6.062554e-02 |
| WBGene00000846 | cup-5 | --- | III | + | 7586096 | 7586380 | 7586108 | 7586380 | 7585418 | 7585730 | 135 | 124 | 73,98 | 61,54 | 77,113 | 106,78 | 111,101 | 69,61 | 0.422415602447569 | 0.522574560886479 | 0.100158958438909 | 1.569506e-03 | 1.553454e-02 |
| WBGene00002041 | hum-8 | --- | IV | - | 430931 | 431224 | 430931 | 430999 | 432628 | 432930 | 248 | 124 | 373,0 | 272,1 | 286,3 | 273,0 | 441,0 | 384,0 | 0.991480298189563 | 1 | 0.0085197018104366 | 4.458502e-02 | 1.866998e-01 |
| WBGene00010479 | K01G5.8 | --- | III | - | 10740524 | 10740606 | 10740524 | 10740597 | 10740644 | 10740772 | 132 | 124 | 188,393 | 102,182 | 181,326 | 213,199 | 215,282 | 191,323 | 0.329340912166734 | 0.419697731895628 | 0.0903568197288936 | 6.826836e-07 | 2.973087e-05 |
| WBGene00007505 | pezo-1 | --- | IV | - | 9363870 | 9363974 | 9363870 | 9363968 | 9364021 | 9364178 | 129 | 124 | 1,45 | 9,22 | 4,22 | 12,22 | 7,21 | 7,29 | 0.131345993795869 | 0.2576726342711 | 0.126326640475231 | 3.309303e-02 | 1.501251e-01 |
| WBGene00008339 | C55A6.10 | --- | V | + | 11506870 | 11507151 | 11506876 | 11507151 | 11506171 | 11506385 | 129 | 124 | 70,73 | 53,66 | 38,76 | 69,49 | 77,68 | 36,57 | 0.418541269208998 | 0.501355133958324 | 0.082813864749326 | 2.631833e-02 | 1.280629e-01 |
| WBGene00000911 | daf-15 | --- | IV | + | 9391268 | 9391448 | 9391286 | 9391448 | 9391009 | 9391226 | 141 | 124 | 66,221 | 66,115 | 80,156 | 99,127 | 137,169 | 53,198 | 0.274806606732176 | 0.339709925111385 | 0.0649033183792086 | 6.010566e-03 | 4.154923e-02 |
| WBGene00011980 | T24B8.7 | --- | II | - | 9047601 | 9047831 | 9047601 | 9047822 | 9047876 | 9048421 | 132 | 124 | 11,15 | 14,18 | 10,23 | 34,11 | 24,7 | 17,34 | 0.369928400954654 | 0.575352635486266 | 0.205424234531612 | 3.820296e-03 | 2.866799e-02 |
| WBGene00004732 | sax-7 | --- | IV | + | 8082680 | 8083478 | 8082689 | 8083478 | 8082520 | 8082636 | 132 | 124 | 90,271 | 109,225 | 90,262 | 142,211 | 198,234 | 103,249 | 0.26370941630118 | 0.374860106455575 | 0.111150690154395 | 2.270256e-08 | 1.521071e-06 |
| WBGene00015501 | C06A5.3 | --- | I | + | 5988821 | 5988932 | 5988830 | 5988932 | 5988624 | 5988761 | 132 | 124 | 117,177 | 66,88 | 92,96 | 145,82 | 129,126 | 135,118 | 0.417115177610334 | 0.540982207620429 | 0.123867030010096 | 5.271573e-06 | 1.586326e-04 |
| WBGene00000459 | ceh-38 | --- | II | + | 6953181 | 6953668 | 6953208 | 6953668 | 6952743 | 6953059 | 150 | 124 | 345,77 | 286,67 | 297,97 | 352,56 | 452,76 | 301,45 | 0.7609474811866 | 0.837684171914165 | 0.0767366907275655 | 7.864132e-06 | 2.209568e-04 |
| WBGene00004767 | sel-10 | --- | V | + | 13819629 | 13819726 | 13819635 | 13819726 | 13819519 | 13819587 | 129 | 124 | 108,130 | 158,91 | 165,110 | 178,99 | 203,129 | 98,71 | 0.555880303298212 | 0.606285790112997 | 0.0504054868147852 | 4.890795e-02 | 1.945152e-01 |
| WBGene00017699 | flcn-1 | --- | II | + | 6944725 | 6944848 | 6944737 | 6944848 | 6942711 | 6942777 | 135 | 124 | 51,221 | 81,119 | 49,185 | 93,132 | 90,149 | 61,144 | 0.240508363784438 | 0.345265944699935 | 0.104757580915497 | 1.524993e-05 | 3.603553e-04 |
| WBGene00044188 | F28F8.9 | --- | V | - | 15578078 | 15578168 | 15578078 | 15578157 | 15578219 | 15578405 | 134 | 124 | 92,34 | 85,38 | 78,40 | 116,55 | 129,92 | 86,55 | 0.678133310457236 | 0.602595724688748 | -0.0755375857684881 | 2.296422e-02 | 1.183540e-01 |
| WBGene00012973 | spat-2 | --- | III | + | 11057277 | 11057395 | 11057283 | 11057395 | 11055999 | 11056276 | 129 | 124 | 145,293 | 102,186 | 151,268 | 181,173 | 230,182 | 115,218 | 0.338688535840511 | 0.468761903392961 | 0.130073367552451 | 3.523541e-10 | 4.384292e-08 |
| WBGene00013465 | Y67H2A.10 | --- | IV | - | 13285961 | 13286111 | 13285961 | 13286102 | 13286164 | 13286322 | 132 | 124 | 92,68 | 91,75 | 100,69 | 96,39 | 182,70 | 104,67 | 0.556344726996005 | 0.670934844192635 | 0.11459011719663 | 1.596387e-04 | 2.780906e-03 |
| WBGene00003965 | pdk-1 | --- | X | + | 1325068 | 1325162 | 1325074 | 1325162 | 1324695 | 1325028 | 129 | 124 | 16,69 | 11,30 | 9,60 | 17,40 | 12,45 | 22,49 | 0.178738738738739 | 0.267852604828463 | 0.0891138660897238 | 3.822842e-02 | 1.690201e-01 |
| WBGene00004095 | pqe-1 | --- | III | + | 5315808 | 5316045 | 5315814 | 5316045 | 5315712 | 5315769 | 129 | 124 | 19,320 | 23,212 | 28,241 | 61,227 | 86,291 | 48,283 | 0.0800760168639354 | 0.189633672917206 | 0.10955765605327 | 3.889401e-12 | 5.646114e-10 |
| WBGene00020346 | rbm-5 | --- | I | + | 6217016 | 6217254 | 6217122 | 6217254 | 6216520 | 6216793 | 229 | 124 | 49,70 | 18,53 | 41,64 | 34,89 | 25,95 | 36,87 | 0.238228230899226 | 0.159536288411273 | -0.078691942487953 | 3.883838e-03 | 2.866799e-02 |
| WBGene00000459 | ceh-38 | --- | II | + | 6953782 | 6954029 | 6953788 | 6954029 | 6953208 | 6953668 | 129 | 124 | 133,305 | 173,207 | 194,259 | 210,223 | 198,194 | 148,310 | 0.38399841445816 | 0.423678922366909 | 0.0396805079087493 | 4.056246e-02 | 1.751372e-01 |
| WBGene00015754 | C14B9.8 | --- | III | - | 8141868 | 8142029 | 8141868 | 8142020 | 8142070 | 8142174 | 132 | 124 | 31,53 | 19,50 | 15,56 | 26,43 | 52,45 | 27,37 | 0.277471770862022 | 0.441056910569106 | 0.163585139707084 | 3.214316e-04 | 4.556636e-03 |
| WBGene00013878 | atfs-1 | --- | V | + | 14196332 | 14196419 | 14196338 | 14196419 | 14196214 | 14196286 | 129 | 124 | 141,165 | 116,122 | 105,143 | 126,88 | 201,132 | 117,154 | 0.44727874210327 | 0.532961607713307 | 0.0856828656100363 | 6.945667e-04 | 8.078939e-03 |
| WBGene00018512 | trpp-8 | --- | I | - | 5614436 | 5614690 | 5614436 | 5614684 | 5614734 | 5615129 | 129 | 124 | 23,78 | 20,68 | 25,36 | 33,40 | 47,53 | 31,73 | 0.264243183954873 | 0.39126726931605 | 0.127024085361177 | 2.327256e-03 | 2.089732e-02 |
| WBGene00017852 | F27C1.2 | --- | I | + | 5430760 | 5430992 | 5430772 | 5430992 | 5430577 | 5430705 | 135 | 124 | 284,844 | 317,736 | 358,659 | 345,603 | 446,673 | 361,886 | 0.282339421768788 | 0.328599229845555 | 0.0462598080767668 | 4.041067e-05 | 8.380403e-04 |
| WBGene00010406 | math-33 | --- | V | + | 11117598 | 11117844 | 11117601 | 11117844 | 11117430 | 11117536 | 126 | 124 | 217,20 | 209,14 | 230,25 | 222,33 | 290,41 | 213,20 | 0.91626303814008 | 0.883590187136342 | -0.0326728510037376 | 4.020058e-02 | 1.751372e-01 |
| WBGene00002992 | lin-3 | --- | IV | + | 11059398 | 11059644 | 11059404 | 11059644 | 11057788 | 11057868 | 129 | 124 | 20,81 | 26,68 | 22,88 | 35,62 | 45,81 | 29,89 | 0.21617741315216 | 0.311113157167848 | 0.0949357440156882 | 6.212470e-03 | 4.227392e-02 |
| WBGene00012674 | bed-1 | --- | V | - | 19121802 | 19121934 | 19121802 | 19121928 | 19121983 | 19122093 | 129 | 124 | 49,134 | 40,54 | 71,80 | 92,71 | 83,84 | 43,116 | 0.364625450268323 | 0.436063299511219 | 0.0714378492428962 | 3.137379e-02 | 1.469171e-01 |
| WBGene00017093 | E02C12.8 | --- | V | - | 9372057 | 9372503 | 9372057 | 9372458 | 9372654 | 9372834 | 168 | 124 | 2,0 | 4,1 | 7,1 | 6,3 | 6,7 | 4,3 | 0.827515400410678 | 0.476007677543186 | -0.351507722867491 | 4.843110e-02 | 1.935023e-01 |
| WBGene00006751 | unc-11 | --- | I | - | 3799880 | 3800032 | 3799880 | 3800026 | 3800191 | 3800279 | 129 | 124 | 57,122 | 47,120 | 50,148 | 77,134 | 102,132 | 52,141 | 0.27513471457799 | 0.352989019926799 | 0.0778543053488091 | 4.175565e-03 | 3.056233e-02 |
| WBGene00015145 | arle-14 | --- | III | + | 5693254 | 5693579 | 5693263 | 5693579 | 5693118 | 5693214 | 132 | 124 | 156,48 | 129,35 | 132,47 | 159,40 | 232,27 | 156,47 | 0.750827670325841 | 0.818427530286211 | 0.0675998599603699 | 6.154406e-03 | 4.220856e-02 |
| WBGene00001325 | eor-2 | --- | X | + | 14604010 | 14604439 | 14604016 | 14604439 | 14603856 | 14603955 | 129 | 124 | 33,47 | 25,39 | 36,44 | 26,49 | 23,44 | 14,49 | 0.410047139942306 | 0.298966704936854 | -0.111080435005452 | 1.639038e-02 | 9.210338e-02 |
| WBGene00008274 | C53B4.4 | --- | IV | + | 8981694 | 8982083 | 8981700 | 8982083 | 8980878 | 8981358 | 129 | 124 | 119,311 | 103,163 | 102,210 | 176,154 | 163,151 | 141,212 | 0.31286795626577 | 0.471583751277602 | 0.158715795011833 | 2.736842e-13 | 5.959474e-11 |
| WBGene00015143 | rbm-26 | --- | III | + | 5712607 | 5713700 | 5712613 | 5713700 | 5712349 | 5712541 | 129 | 124 | 55,363 | 74,312 | 67,257 | 80,323 | 108,352 | 75,310 | 0.168156532809343 | 0.204237304057566 | 0.036080771248223 | 2.519966e-02 | 1.254223e-01 |
| WBGene00007428 | wdr-20 | --- | V | - | 10130877 | 10130894 | 10130877 | 10130885 | 10130930 | 10131212 | 132 | 124 | 30,190 | 25,180 | 35,176 | 28,236 | 21,273 | 18,226 | 0.134083044982699 | 0.0788774115145071 | -0.0552056334681919 | 6.244679e-04 | 7.450843e-03 |
| WBGene00011705 | clp-9 | --- | V | - | 9885039 | 9885231 | 9885039 | 9885142 | 9885566 | 9885619 | 212 | 124 | 22,3 | 22,4 | 38,6 | 25,2 | 32,1 | 57,3 | 0.786753327143299 | 0.917445482866044 | 0.130692155722744 | 3.060879e-02 | 1.441095e-01 |
| WBGene00006392 | taf-10 | --- | V | + | 4687230 | 4687298 | 4687239 | 4687298 | 4687108 | 4687188 | 132 | 124 | 207,396 | 169,229 | 182,293 | 228,189 | 251,215 | 184,285 | 0.363464447806354 | 0.474774774774775 | 0.111310326968421 | 1.859245e-09 | 1.799336e-07 |
| WBGene00000102 | akt-1 | --- | V | + | 10250840 | 10251032 | 10250944 | 10251032 | 10250478 | 10250676 | 227 | 124 | 677,6 | 527,2 | 709,10 | 706,4 | 892,2 | 700,1 | 0.983066581571335 | 0.994454545771809 | 0.0113879642004742 | 8.634887e-03 | 5.449990e-02 |
| WBGene00003919 | par-4 | --- | V | + | 18101697 | 18101964 | 18101709 | 18101964 | 18101533 | 18101644 | 135 | 124 | 179,75 | 156,34 | 178,39 | 247,17 | 310,54 | 151,49 | 0.760981912144703 | 0.844218786060466 | 0.0832368739157633 | 1.041088e-04 | 1.889141e-03 |
| WBGene00016650 | ubr-4 | --- | I | - | 4612025 | 4613022 | 4612025 | 4613016 | 4613278 | 4613673 | 129 | 124 | 38,146 | 69,137 | 70,131 | 87,131 | 137,155 | 78,244 | 0.291265228123258 | 0.353890642423784 | 0.062625414300526 | 1.433646e-02 | 8.269572e-02 |
| WBGene00013111 | sta-1 | --- | IV | + | 16670717 | 16670840 | 16670726 | 16670840 | 16670540 | 16670675 | 132 | 124 | 97,135 | 78,75 | 112,94 | 60,66 | 48,73 | 104,149 | 0.470019546727244 | 0.408808161234138 | -0.0612113854931061 | 4.426847e-02 | 1.862697e-01 |
| WBGene00018319 | hda-6 | --- | IV | - | 5363535 | 5363810 | 5363535 | 5363804 | 5363853 | 5364141 | 129 | 124 | 32,125 | 23,130 | 24,112 | 45,121 | 55,149 | 33,99 | 0.171441572306131 | 0.257313591187805 | 0.0858720188816745 | 1.342236e-03 | 1.375397e-02 |
| WBGene00006941 | wnk-1 | --- | IV | + | 9211243 | 9212222 | 9211250 | 9212222 | 9210471 | 9210676 | 130 | 124 | 272,556 | 210,406 | 197,322 | 235,251 | 274,339 | 203,449 | 0.335287277592826 | 0.395275745663912 | 0.0599884680710862 | 1.424783e-04 | 2.532625e-03 |
| WBGene00000818 | csn-6 | --- | IV | + | 13302936 | 13303066 | 13302951 | 13303066 | 13302556 | 13302898 | 138 | 124 | 28,121 | 44,88 | 46,81 | 74,80 | 80,111 | 43,97 | 0.267730366683744 | 0.380664464252322 | 0.112934097568577 | 3.298358e-04 | 4.556636e-03 |
| WBGene00003196 | mel-11 | --- | II | + | 9363209 | 9363355 | 9363218 | 9363355 | 9362967 | 9363151 | 132 | 124 | 90,127 | 99,119 | 76,160 | 146,141 | 190,129 | 104,148 | 0.380095313006061 | 0.4971932638332 | 0.117097950827138 | 5.263501e-06 | 1.586326e-04 |
| WBGene00008682 | lex-1 | --- | IV | + | 12080087 | 12082158 | 12080093 | 12082158 | 12079827 | 12080045 | 129 | 124 | 124,59 | 90,47 | 113,73 | 188,27 | 170,50 | 105,50 | 0.637156460660915 | 0.777993088962667 | 0.140836628301751 | 3.841885e-07 | 1.859046e-05 |
| WBGene00011194 | R10D12.13 | U2 snRNP | V | + | 13962612 | 13962727 | 13962679 | 13962727 | 13962416 | 13962562 | 190 | 124 | 89,62 | 72,18 | 96,60 | 104,42 | 139,42 | 135,41 | 0.545050283915988 | 0.663702528956982 | 0.118652245040994 | 7.091430e-04 | 8.078939e-03 |
| WBGene00018756 | plpp-1.1 | --- | II | - | 3925519 | 3926085 | 3925519 | 3926079 | 3926916 | 3927137 | 129 | 124 | 142,909 | 151,684 | 150,651 | 131,509 | 181,648 | 197,819 | 0.159496875798472 | 0.198465505314131 | 0.0389686295156596 | 2.161951e-04 | 3.487146e-03 |
| WBGene00020830 | T26C11.4 | --- | X | - | 1843353 | 1843473 | 1843353 | 1843464 | 1843696 | 1843792 | 132 | 124 | 2,32 | 4,23 | 8,32 | 7,33 | 12,20 | 12,31 | 0.131316187594554 | 0.25743369943745 | 0.126117511842896 | 1.941910e-02 | 1.037671e-01 |
| WBGene00021009 | afd-1 | --- | I | + | 2204924 | 2205308 | 2205212 | 2205308 | 2203573 | 2203864 | 248 | 124 | 444,44 | 381,16 | 366,27 | 384,15 | 459,17 | 442,15 | 0.872527472527473 | 0.93183466279913 | 0.0593071902716572 | 1.751098e-04 | 2.933089e-03 |
| WBGene00021597 | spsb-1 | --- | II | + | 15222824 | 15223191 | 15222830 | 15223191 | 15220191 | 15220445 | 129 | 124 | 39,466 | 64,333 | 65,479 | 70,383 | 73,338 | 61,494 | 0.112184561698278 | 0.13896534106828 | 0.0267807793700018 | 3.013532e-02 | 1.432673e-01 |
| WBGene00022152 | atg-5 | --- | I | + | 1712709 | 1712828 | 1712715 | 1712828 | 1710668 | 1710996 | 129 | 124 | 102,169 | 90,101 | 80,94 | 112,95 | 159,122 | 57,132 | 0.4180258787368 | 0.47462453175872 | 0.0565986530219197 | 4.040915e-02 | 1.751372e-01 |
| WBGene00013877 | ZC376.6 | --- | V | + | 14190161 | 14190295 | 14190173 | 14190295 | 14189974 | 14190121 | 135 | 124 | 62,57 | 65,39 | 54,24 | 82,29 | 102,33 | 82,32 | 0.580788738225857 | 0.722161404737925 | 0.141372666512069 | 2.317418e-04 | 3.669948e-03 |
| WBGene00004336 | ret-1 | --- | V | - | 14829738 | 14830122 | 14829738 | 14830110 | 14830166 | 14830317 | 135 | 124 | 278,1224 | 275,960 | 225,952 | 275,860 | 335,903 | 268,1087 | 0.185583034518845 | 0.220557430584536 | 0.0349743960656918 | 1.005714e-04 | 1.863780e-03 |
| WBGene00006697 | uaf-1 | --- | III | - | 1067832 | 1068251 | 1067832 | 1067952 | 1070422 | 1070655 | 248 | 124 | 734,433 | 359,312 | 489,382 | 296,345 | 393,432 | 584,381 | 0.412408759124088 | 0.354694901086654 | -0.0577138580374339 | 1.490892e-05 | 3.603553e-04 |
| WBGene00009724 | F45D3.4 | --- | V | - | 12552210 | 12553023 | 12552210 | 12552906 | 12553078 | 12553191 | 240 | 124 | 5174,611 | 3260,364 | 4167,350 | 3405,343 | 3828,338 | 5135,495 | 0.830898196460136 | 0.844570542417087 | 0.0136723459569511 | 1.679268e-02 | 9.375912e-02 |
| WBGene00000463 | ceh-43 | --- | III | - | 4447361 | 4447601 | 4447361 | 4447574 | 4448971 | 4449047 | 150 | 124 | 7,118 | 9,61 | 3,59 | 12,36 | 6,53 | 23,88 | 0.0619087660290099 | 0.160713156730101 | 0.0988043907010906 | 2.623235e-04 | 3.939376e-03 |
| WBGene00006941 | wnk-1 | --- | IV | + | 9208075 | 9208516 | 9208084 | 9208516 | 9207233 | 9207998 | 132 | 124 | 155,735 | 159,534 | 152,521 | 155,355 | 179,433 | 101,594 | 0.196501441862996 | 0.228207341219475 | 0.0317058993564792 | 1.223461e-02 | 7.200235e-02 |
| WBGene00018161 | F38A5.2 | --- | IV | - | 6584908 | 6585136 | 6584908 | 6585130 | 6585207 | 6585253 | 129 | 124 | 68,119 | 62,95 | 82,84 | 106,70 | 141,83 | 68,86 | 0.406117719758999 | 0.558870240803537 | 0.152752521044538 | 6.544602e-07 | 2.973087e-05 |
| WBGene00004105 | szy-20 | --- | II | - | 8968126 | 8968435 | 8968126 | 8968429 | 8968515 | 8968963 | 129 | 124 | 108,78 | 108,45 | 122,84 | 146,56 | 206,65 | 82,63 | 0.610828536034395 | 0.693934392407675 | 0.0831058563732799 | 3.454388e-03 | 2.686404e-02 |
| WBGene00011240 | mask-1 | --- | IV | + | 10381652 | 10381807 | 10381658 | 10381807 | 10381446 | 10381578 | 129 | 124 | 18,48 | 20,37 | 16,53 | 32,53 | 49,35 | 21,57 | 0.273328434974284 | 0.403406372595924 | 0.13007793762164 | 4.858042e-03 | 3.496987e-02 |
| WBGene00003476 | mtm-3 | --- | III | - | 3799800 | 3799875 | 3799800 | 3799863 | 3799917 | 3800054 | 135 | 124 | 103,374 | 105,291 | 110,354 | 131,276 | 200,286 | 84,346 | 0.222783437007407 | 0.295679154217421 | 0.0728957172100143 | 1.396265e-05 | 3.576902e-04 |
| WBGene00000894 | dab-1 | --- | II | + | 8228238 | 8228386 | 8228244 | 8228386 | 8227558 | 8228193 | 129 | 124 | 209,442 | 153,392 | 166,421 | 219,297 | 306,410 | 195,421 | 0.287957355289026 | 0.380251456608402 | 0.0922941013193762 | 3.370912e-09 | 2.446720e-07 |
| WBGene00014101 | gopc-1 | --- | I | - | 14188019 | 14188130 | 14188019 | 14188124 | 14188194 | 14188293 | 129 | 124 | 72,18 | 40,19 | 58,45 | 38,1 | 33,9 | 75,16 | 0.665866447659359 | 0.843694659334514 | 0.177828211675155 | 4.222709e-05 | 8.553441e-04 |
| WBGene00013307 | Y57G11C.9 | --- | IV | + | 14787505 | 14788106 | 14787980 | 14788106 | 14787167 | 14787383 | 248 | 124 | 211,271 | 127,261 | 118,240 | 122,306 | 137,322 | 158,281 | 0.228 | 0.186577181208054 | -0.0414228187919463 | 2.644471e-03 | 2.269367e-02 |
| WBGene00004226 | ptr-12 | --- | I | - | 2591000 | 2591749 | 2591000 | 2591144 | 2591886 | 2592387 | 248 | 124 | 46,31 | 18,21 | 21,22 | 20,13 | 12,25 | 24,46 | 0.36480686695279 | 0.25 | -0.11480686695279 | 2.086684e-02 | 1.088324e-01 |
| WBGene00003209 | mel-26 | --- | I | + | 9134974 | 9135094 | 9134986 | 9135094 | 9134180 | 9134438 | 135 | 124 | 196,313 | 254,242 | 205,206 | 265,266 | 297,255 | 250,256 | 0.441521024163518 | 0.489768122850625 | 0.0482470986871065 | 8.487010e-03 | 5.395756e-02 |
| WBGene00000156 | apr-1 | --- | I | + | 8052481 | 8054035 | 8052487 | 8054035 | 8051852 | 8052440 | 129 | 124 | 84,113 | 88,92 | 91,94 | 147,92 | 150,125 | 91,96 | 0.458143095963924 | 0.543705997355604 | 0.0855629013916795 | 2.682627e-03 | 2.269367e-02 |
| WBGene00021349 | arl-13 | --- | I | + | 2068868 | 2069049 | 2068877 | 2069049 | 2068504 | 2068827 | 132 | 124 | 0,14 | 0,12 | 6,10 | 1,6 | 7,7 | 9,8 | 0.135371179039301 | 0.431967213114754 | 0.296596034075453 | 3.244320e-03 | 2.591194e-02 |
| WBGene00001695 | grd-6 | --- | V | + | 9223265 | 9223564 | 9223484 | 9223564 | 9222828 | 9223215 | 248 | 124 | 12,2 | 61,13 | 106,23 | 165,8 | 147,15 | 34,9 | 0.701960784313725 | 0.84390243902439 | 0.141941654710665 | 1.400625e-03 | 1.418540e-02 |
| WBGene00012245 | rbm-25 | --- | V | + | 12502563 | 12503086 | 12502569 | 12503086 | 12501664 | 12502232 | 129 | 124 | 128,372 | 115,215 | 128,328 | 154,200 | 242,255 | 145,341 | 0.280445503813118 | 0.395151029640451 | 0.114705525827333 | 4.335440e-10 | 4.720211e-08 |
| WBGene00000549 | cls-2 | --- | III | + | 9058263 | 9058392 | 9058272 | 9058392 | 9057780 | 9058221 | 132 | 124 | 78,134 | 50,93 | 71,68 | 54,67 | 61,84 | 34,154 | 0.387889839034205 | 0.314560065377281 | -0.0733297736569238 | 1.831184e-02 | 9.906590e-02 |
| WBGene00015470 | C05D2.10 | --- | III | - | 5599322 | 5599799 | 5599322 | 5599793 | 5599843 | 5600058 | 129 | 124 | 39,383 | 55,298 | 59,369 | 62,348 | 96,441 | 62,390 | 0.122858141974589 | 0.152087015180826 | 0.0292288732062372 | 3.291386e-02 | 1.500941e-01 |
| WBGene00009665 | lmd-1 | integral component of membrane | I | + | 8610348 | 8610449 | 8610356 | 8610449 | 8610159 | 8610307 | 131 | 124 | 33,88 | 21,114 | 17,112 | 14,138 | 36,198 | 18,124 | 0.176298610276743 | 0.122750829790951 | -0.0535477804857922 | 2.497557e-02 | 1.254223e-01 |
| WBGene00010367 | H05L14.2 | --- | I | + | 7985303 | 7985586 | 7985312 | 7985586 | 7985086 | 7985148 | 132 | 124 | 0,19 | 7,7 | 7,16 | 2,14 | 1,20 | 1,14 | 0.238461538461538 | 0.0725995316159251 | -0.165862006845613 | 1.998008e-02 | 1.054706e-01 |
| WBGene00019665 | K11H12.9 | --- | IV | + | 649903 | 650375 | 649915 | 650375 | 649665 | 649859 | 135 | 124 | 7,24 | 2,7 | 1,10 | 1,5 | 5,2 | 9,13 | 0.183025830258303 | 0.407894736842105 | 0.224868906583803 | 2.913692e-02 | 1.394410e-01 |
| WBGene00000938 | dcp-66 | --- | I | - | 7539174 | 7539259 | 7539174 | 7539250 | 7539302 | 7539501 | 132 | 124 | 118,446 | 91,204 | 103,354 | 155,234 | 209,256 | 99,321 | 0.225960190636389 | 0.349085514155073 | 0.123125323518684 | 2.335682e-12 | 4.068759e-10 |
| WBGene00021898 | Y54G2A.36 | --- | IV | + | 2900872 | 2901365 | 2900898 | 2901365 | 2897695 | 2897802 | 149 | 124 | 35,83 | 49,121 | 42,125 | 54,191 | 55,195 | 19,103 | 0.241689225771521 | 0.178873699750938 | -0.0628155260205832 | 8.908718e-03 | 5.582369e-02 |
| WBGene00003676 | nhr-86 | --- | V | - | 2062237 | 2062517 | 2062237 | 2062508 | 2063330 | 2063515 | 132 | 124 | 64,50 | 81,34 | 50,27 | 93,30 | 105,30 | 94,43 | 0.622682323856613 | 0.7270098787246 | 0.104327554867987 | 3.850807e-03 | 2.866799e-02 |
| WBGene00010743 | K10D6.3 | --- | V | + | 11200744 | 11201025 | 11200934 | 11201025 | 11200664 | 11200693 | 248 | 124 | 11,0 | 4,0 | 9,4 | 7,0 | 15,0 | 14,0 | 0.75 | 1 | 0.25 | 3.222501e-02 | 1.492978e-01 |
| WBGene00002185 | kel-3 | --- | III | + | 13466960 | 13467035 | 13466966 | 13467035 | 13466829 | 13466917 | 129 | 124 | 9,103 | 34,121 | 20,132 | 36,118 | 42,134 | 33,123 | 0.145377400625279 | 0.221503403659537 | 0.0761260030342584 | 3.064884e-03 | 2.566840e-02 |
| WBGene00007631 | wht-3 | --- | III | - | 4150624 | 4150811 | 4150624 | 4150769 | 4150869 | 4150892 | 165 | 124 | 0,0 | 2,2 | 5,2 | 9,0 | 8,0 | 12,0 | 0.568062827225131 | 1 | 0.431937172774869 | 3.610898e-03 | 2.758853e-02 |
| WBGene00006405 | itsn-1 | --- | IV | + | 17121826 | 17122463 | 17121832 | 17122463 | 17121561 | 17121786 | 129 | 124 | 40,266 | 30,169 | 38,221 | 52,224 | 88,324 | 40,234 | 0.136630754162586 | 0.18117177226903 | 0.0445410181064446 | 1.132551e-02 | 6.806785e-02 |
| WBGene00009806 | F47B8.5 | --- | V | - | 14326480 | 14326558 | 14326480 | 14326552 | 14327060 | 14327180 | 129 | 124 | 11,0 | 4,2 | 10,1 | 8,3 | 4,6 | 3,0 | 0.889016346429596 | 0.615690168818272 | -0.273326177611323 | 4.489360e-02 | 1.870925e-01 |
| WBGene00003026 | lin-41 | --- | I | - | 9338502 | 9338816 | 9338502 | 9338804 | 9338855 | 9338952 | 135 | 124 | 67,109 | 62,99 | 61,99 | 79,116 | 127,85 | 60,105 | 0.362433658949312 | 0.443965865345788 | 0.0815322063964767 | 6.444811e-03 | 4.351496e-02 |
| WBGene00000421 | ced-7 | --- | III | - | 9573464 | 9573649 | 9573464 | 9573643 | 9573695 | 9574153 | 129 | 124 | 92,169 | 64,118 | 72,222 | 120,153 | 129,190 | 88,200 | 0.30098048609115 | 0.373657620601779 | 0.0726771345106293 | 2.015705e-03 | 1.867744e-02 |
| WBGene00017920 | jmjd-1.2 | --- | IV | + | 4661356 | 4661641 | 4661452 | 4661641 | 4661238 | 4661310 | 219 | 124 | 65,86 | 29,64 | 53,96 | 42,114 | 49,130 | 59,94 | 0.252808521261546 | 0.200816220768284 | -0.0519923004932621 | 3.801251e-02 | 1.690201e-01 |
| WBGene00000793 | crh-1 | --- | III | + | 11688125 | 11688336 | 11688131 | 11688336 | 11685209 | 11685303 | 129 | 124 | 89,450 | 54,293 | 54,327 | 65,184 | 78,237 | 44,294 | 0.150365017419887 | 0.200895835318784 | 0.050530817898897 | 2.043637e-03 | 1.873692e-02 |
| WBGene00021554 | Y45G5AL.1 | --- | V | + | 4144760 | 4144929 | 4144766 | 4144929 | 4144504 | 4144718 | 129 | 124 | 93,211 | 114,147 | 85,163 | 130,125 | 181,163 | 91,217 | 0.350116518560778 | 0.433487255746002 | 0.0833707371852238 | 3.957752e-04 | 5.145077e-03 |
| WBGene00009163 | drsh-1 | --- | I | - | 9781741 | 9782828 | 9781741 | 9782822 | 9783235 | 9784106 | 129 | 124 | 42,15 | 47,17 | 42,16 | 76,1 | 94,10 | 38,11 | 0.724014975931539 | 0.900873209919665 | 0.176858233988126 | 5.281684e-06 | 1.586326e-04 |
| WBGene00019504 | K07H8.2 | --- | IV | + | 8292764 | 8292879 | 8292776 | 8292879 | 8291731 | 8291835 | 135 | 124 | 109,100 | 120,66 | 117,120 | 247,88 | 146,95 | 123,105 | 0.526339033785607 | 0.622025198320112 | 0.0956861645345046 | 3.385829e-04 | 4.556636e-03 |
| WBGene00012496 | Y24F12A.1 | --- | IV | + | 11605659 | 11605794 | 11605665 | 11605794 | 11605434 | 11605618 | 129 | 124 | 129,15 | 124,13 | 134,11 | 199,24 | 175,40 | 120,24 | 0.905109489051095 | 0.843653591890701 | -0.0614558971603941 | 5.216542e-03 | 3.724269e-02 |
| WBGene00022712 | ZK355.2 | --- | II | + | 2924058 | 2924175 | 2924088 | 2924175 | 2923916 | 2924005 | 153 | 124 | 95,39 | 81,73 | 100,65 | 67,44 | 62,52 | 74,79 | 0.558257890873501 | 0.484570812558954 | -0.073687078314547 | 4.088360e-02 | 1.754168e-01 |
| WBGene00009796 | F46G10.1 | --- | X | - | 13311352 | 13311471 | 13311352 | 13311464 | 13311515 | 13311574 | 130 | 124 | 50,1000 | 35,809 | 43,961 | 31,592 | 51,788 | 72,945 | 0.0422159096954028 | 0.0594250434111518 | 0.017209133715749 | 3.857646e-03 | 2.866799e-02 |
| WBGene00004203 | swsn-1 | --- | V | - | 20239179 | 20240016 | 20239179 | 20240007 | 20241259 | 20242225 | 132 | 124 | 38,182 | 31,128 | 25,127 | 26,122 | 51,111 | 30,121 | 0.168099221228728 | 0.221148076538436 | 0.0530488553097079 | 3.272772e-02 | 1.500308e-01 |
| WBGene00003695 | nhr-105 | --- | IV | + | 7051727 | 7051871 | 7051775 | 7051871 | 7051548 | 7051649 | 171 | 124 | 582,146 | 337,61 | 294,69 | 297,44 | 262,69 | 515,64 | 0.76116351564714 | 0.814816174446137 | 0.0536526587989974 | 2.297330e-03 | 2.084349e-02 |
| WBGene00017885 | dip-2 | --- | I | + | 4943863 | 4944395 | 4943869 | 4944395 | 4943629 | 4943805 | 129 | 124 | 19,117 | 16,88 | 15,83 | 28,99 | 45,56 | 16,97 | 0.143015316479055 | 0.253444791475289 | 0.110429474996234 | 2.885248e-04 | 4.259408e-03 |
| WBGene00012458 | ash-2 | --- | II | + | 11980765 | 11981096 | 11980771 | 11981096 | 11980627 | 11980720 | 129 | 124 | 60,72 | 58,31 | 48,70 | 64,50 | 61,92 | 22,66 | 0.479802335609893 | 0.404527296937417 | -0.0752750386724758 | 4.757562e-02 | 1.927366e-01 |
| WBGene00015751 | C14A11.6 | --- | X | - | 2813106 | 2813187 | 2813106 | 2813163 | 2813781 | 2813972 | 147 | 124 | 2,4 | 1,9 | 1,17 | 1,6 | 3,3 | 3,1 | 0.101100693028944 | 0.37125748502994 | 0.270156792000996 | 2.829967e-02 | 1.364213e-01 |
| WBGene00010502 | K02C4.3 | --- | II | - | 8082097 | 8082440 | 8082097 | 8082431 | 8082742 | 8083077 | 132 | 124 | 185,24 | 174,19 | 178,43 | 232,17 | 283,11 | 187,19 | 0.854349499615089 | 0.933470595804916 | 0.0791210961898272 | 3.648995e-06 | 1.339875e-04 |
| WBGene00010013 | F54B3.1 | --- | II | + | 10266646 | 10266839 | 10266652 | 10266839 | 10265735 | 10266186 | 129 | 124 | 75,138 | 114,93 | 107,142 | 137,84 | 165,130 | 139,144 | 0.432723028495302 | 0.542145024091369 | 0.109421995596067 | 3.419145e-05 | 7.263598e-04 |
| WBGene00014087 | ZK809.5 | --- | IV | + | 11657039 | 11657303 | 11657045 | 11657303 | 11656918 | 11656990 | 129 | 124 | 33,86 | 28,45 | 27,57 | 52,67 | 67,77 | 45,52 | 0.310317370037538 | 0.445769399386234 | 0.135452029348696 | 5.801363e-04 | 7.116884e-03 |
| WBGene00009284 | F31C3.2 | --- | I | + | 15038052 | 15038268 | 15038061 | 15038268 | 15037738 | 15038007 | 132 | 124 | 157,70 | 133,74 | 132,72 | 185,59 | 269,82 | 140,85 | 0.6473033151905 | 0.711734693877551 | 0.0644313786870513 | 9.809235e-03 | 6.059464e-02 |
| WBGene00020453 | fbxa-55 | --- | III | - | 933838 | 934466 | 933838 | 934379 | 935954 | 936067 | 210 | 124 | 8,21 | 20,6 | 23,13 | 23,5 | 23,11 | 42,11 | 0.429502852485738 | 0.658062959835967 | 0.228560107350229 | 2.626603e-03 | 2.269367e-02 |
| WBGene00003478 | mtm-6 | --- | III | + | 13352615 | 13353562 | 13352621 | 13353562 | 13352328 | 13352404 | 129 | 124 | 110,25 | 99,28 | 86,10 | 105,40 | 125,48 | 68,33 | 0.818216386695596 | 0.703030764254866 | -0.11518562244073 | 2.610380e-04 | 3.939376e-03 |
| WBGene00018788 | shc-1 | --- | I | - | 971459 | 972086 | 971459 | 971950 | 972647 | 972720 | 248 | 124 | 124,25 | 62,20 | 64,24 | 24,20 | 33,22 | 92,27 | 0.644329896907217 | 0.519163763066202 | -0.125166133841014 | 1.178155e-02 | 6.980769e-02 |
| WBGene00018316 | F41H10.3 | --- | IV | + | 5372673 | 5373162 | 5372685 | 5373162 | 5372023 | 5372567 | 135 | 124 | 58,134 | 57,123 | 59,134 | 73,104 | 88,130 | 53,138 | 0.290152095856699 | 0.345718901453958 | 0.0555668055972589 | 4.590655e-02 | 1.886066e-01 |
| WBGene00004117 | sin-3 | --- | I | + | 8422853 | 8422946 | 8422859 | 8422946 | 8422657 | 8422807 | 129 | 124 | 129,106 | 164,56 | 182,92 | 207,49 | 254,77 | 167,83 | 0.642550127637292 | 0.74281953201759 | 0.100269404380298 | 2.386872e-05 | 5.197414e-04 |
| WBGene00012973 | spat-2 | --- | III | + | 11042085 | 11042259 | 11042092 | 11042259 | 11038430 | 11038511 | 130 | 124 | 30,95 | 26,81 | 33,89 | 56,72 | 33,75 | 30,93 | 0.242624104119949 | 0.321089738010271 | 0.0784656338903217 | 2.102952e-02 | 1.090281e-01 |
| WBGene00011344 | T01G9.2 | --- | I | - | 8287863 | 8288004 | 8287863 | 8287995 | 8288048 | 8288124 | 132 | 124 | 34,247 | 43,184 | 42,231 | 71,266 | 77,284 | 46,245 | 0.144468376737811 | 0.186486402679153 | 0.042018025941342 | 1.714380e-02 | 9.431641e-02 |
| WBGene00007621 | C16C2.4 | --- | I | - | 9714233 | 9714526 | 9714233 | 9714514 | 9714565 | 9714811 | 135 | 124 | 56,502 | 61,416 | 75,483 | 99,439 | 147,534 | 77,576 | 0.11180456741945 | 0.160743597667428 | 0.0489390302479776 | 1.929586e-05 | 4.422814e-04 |
| WBGene00001173 | egl-4 | --- | IV | + | 1875641 | 1876207 | 1875650 | 1876207 | 1873584 | 1873692 | 132 | 124 | 122,1092 | 115,681 | 136,795 | 124,588 | 160,765 | 103,762 | 0.120063962121133 | 0.146676936619718 | 0.0266129744985849 | 3.251032e-03 | 2.591194e-02 |
| WBGene00006379 | sys-1 | --- | I | - | 9982738 | 9982857 | 9982738 | 9982846 | 9982895 | 9983208 | 134 | 124 | 1,42 | 3,61 | 8,46 | 11,52 | 17,63 | 8,38 | 0.0693576955346322 | 0.178803172314348 | 0.109445476779715 | 1.713169e-03 | 1.657966e-02 |
| WBGene00003611 | nhr-12 | --- | V | - | 10082414 | 10082685 | 10082414 | 10082676 | 10082744 | 10082859 | 132 | 124 | 21,2 | 7,2 | 20,3 | 14,4 | 18,13 | 15,6 | 0.865619546247818 | 0.657490974729242 | -0.208128571518576 | 1.109085e-02 | 6.755334e-02 |
| WBGene00001194 | egl-27 | --- | II | + | 6846530 | 6847040 | 6846536 | 6847040 | 6845361 | 6846487 | 129 | 124 | 212,425 | 175,221 | 219,350 | 204,241 | 276,305 | 204,377 | 0.369025870705404 | 0.416003296008005 | 0.0469774253026006 | 6.916748e-03 | 4.564005e-02 |
| WBGene00006888 | vbh-1 | --- | I | - | 3161642 | 3161702 | 3161642 | 3161693 | 3161738 | 3161789 | 132 | 124 | 410,441 | 397,369 | 430,350 | 575,364 | 634,390 | 405,423 | 0.500437182716275 | 0.562970464135021 | 0.0625332814187461 | 7.515700e-06 | 2.182058e-04 |
| WBGene00018953 | F56C9.10 | --- | III | - | 7332939 | 7333431 | 7332939 | 7333422 | 7333607 | 7333790 | 132 | 124 | 214,384 | 152,229 | 164,316 | 203,246 | 193,281 | 149,256 | 0.348928579013316 | 0.395352646604577 | 0.0464240675912613 | 1.133162e-02 | 6.806785e-02 |
| WBGene00012435 | flh-1 | --- | IV | - | 9261809 | 9262611 | 9261809 | 9262602 | 9262657 | 9263428 | 132 | 124 | 213,49 | 215,46 | 227,44 | 251,51 | 324,34 | 190,34 | 0.815723927366222 | 0.857933579335793 | 0.0422096519695713 | 2.518908e-02 | 1.254223e-01 |
| WBGene00004031 | pis-1 | --- | IV | - | 9796146 | 9796251 | 9796146 | 9796245 | 9796297 | 9796358 | 129 | 124 | 75,77 | 86,31 | 90,49 | 118,33 | 156,23 | 82,45 | 0.605796368024602 | 0.772112710545187 | 0.166316342520585 | 1.694901e-07 | 9.226617e-06 |
| WBGene00002066 | ifg-1 | --- | II | + | 8217344 | 8217438 | 8217356 | 8217438 | 8217230 | 8217302 | 135 | 124 | 261,63 | 220,55 | 243,75 | 216,42 | 302,54 | 246,50 | 0.775060217040343 | 0.827779039896545 | 0.0527188228562018 | 6.715460e-03 | 4.466832e-02 |
| WBGene00004210 | ptc-3 | --- | II | - | 2848168 | 2848309 | 2848168 | 2848300 | 2848350 | 2849649 | 132 | 124 | 86,109 | 106,104 | 129,140 | 91,92 | 58,143 | 109,130 | 0.460694444444444 | 0.39904205957192 | -0.0616523848725241 | 2.544842e-02 | 1.259315e-01 |
| WBGene00022300 | Y76B12C.6 | --- | IV | + | 1990549 | 1991354 | 1990558 | 1991354 | 1989258 | 1989466 | 132 | 124 | 73,68 | 87,31 | 79,43 | 130,17 | 177,19 | 105,28 | 0.612567176519223 | 0.858102660575114 | 0.245535484055891 | 5.854275e-16 | 2.549537e-13 |
| WBGene00017512 | nhr-179 | --- | V | - | 1616394 | 1616850 | 1616394 | 1616829 | 1617448 | 1617540 | 144 | 124 | 4,0 | 2,0 | 2,0 | 0,0 | 1,4 | 2,0 | 1 | 0.392405063291139 | -0.607594936708861 | 2.564103e-02 | 1.259315e-01 |
| WBGene00000994 | dic-1 | nucleus | IV | + | 8693173 | 8693345 | 8693182 | 8693345 | 8692874 | 8693091 | 132 | 124 | 43,26 | 21,24 | 29,16 | 39,16 | 64,17 | 34,27 | 0.569650266745702 | 0.68202986992131 | 0.112379603175608 | 3.432745e-02 | 1.549182e-01 |
| WBGene00004167 | pqn-87 | --- | II | - | 12210812 | 12211182 | 12210812 | 12211173 | 12211789 | 12212378 | 132 | 124 | 184,487 | 196,394 | 186,403 | 235,266 | 279,384 | 144,446 | 0.292833539170199 | 0.360605310610614 | 0.0677717714404149 | 1.270196e-05 | 3.352547e-04 |
| WBGene00020012 | R11G1.6 | --- | X | - | 3639765 | 3639842 | 3639765 | 3639836 | 3639931 | 3640048 | 129 | 124 | 20,154 | 20,111 | 17,119 | 8,115 | 5,127 | 17,114 | 0.124867500529998 | 0.0749335267101765 | -0.0499339738198214 | 1.704666e-02 | 9.431641e-02 |
| WBGene00007554 | pptr-2 | --- | V | - | 11029177 | 11029282 | 11029177 | 11029270 | 11029321 | 11029387 | 135 | 124 | 61,181 | 55,170 | 62,173 | 80,180 | 103,185 | 70,134 | 0.237814075766065 | 0.317732967377984 | 0.0799188916119192 | 5.647415e-04 | 7.026997e-03 |
| WBGene00019351 | lron-7 | --- | X | + | 7295325 | 7295425 | 7295331 | 7295425 | 7295148 | 7295244 | 129 | 124 | 430,29 | 337,9 | 367,8 | 381,22 | 552,35 | 489,32 | 0.959508700102354 | 0.938868744309378 | -0.020639955792976 | 2.050480e-02 | 1.075885e-01 |
| WBGene00000912 | daf-16 | --- | I | + | 10764350 | 10764496 | 10764356 | 10764496 | 10763587 | 10763706 | 129 | 124 | 202,347 | 127,210 | 139,263 | 169,168 | 173,244 | 138,241 | 0.354259761189656 | 0.414032012354181 | 0.0597722511645246 | 2.657291e-03 | 2.269367e-02 |
| WBGene00018681 | denn-4 | --- | IV | + | 1929365 | 1929521 | 1929371 | 1929521 | 1929166 | 1929316 | 129 | 124 | 6,63 | 14,41 | 10,52 | 8,22 | 28,38 | 16,45 | 0.15601409159537 | 0.322512879507828 | 0.166498787912458 | 3.400474e-04 | 4.556636e-03 |
| WBGene00018681 | denn-4 | --- | IV | + | 1939090 | 1939188 | 1939096 | 1939188 | 1938108 | 1938199 | 129 | 124 | 36,100 | 20,46 | 37,47 | 35,55 | 38,41 | 37,51 | 0.31656098163551 | 0.418366407999264 | 0.101805426363754 | 1.638444e-02 | 9.210338e-02 |
| WBGene00001725 | grl-16 | --- | I | - | 573639 | 574073 | 573639 | 573893 | 574144 | 574662 | 248 | 124 | 157,103 | 252,120 | 536,149 | 653,38 | 910,50 | 228,67 | 0.559502664298401 | 0.852451213707758 | 0.292948549409357 | 4.450972e-53 | 3.876796e-50 |
| WBGene00001561 | gei-4 | --- | III | - | 346767 | 346883 | 346767 | 346868 | 347038 | 347437 | 138 | 124 | 41,811 | 51,501 | 49,560 | 46,393 | 62,560 | 65,576 | 0.0633891668479443 | 0.0922849251894999 | 0.0288957583415555 | 5.898517e-04 | 7.135567e-03 |
| WBGene00003965 | pdk-1 | --- | X | + | 1326072 | 1326129 | 1326078 | 1326129 | 1325428 | 1325670 | 129 | 124 | 22,79 | 12,54 | 19,65 | 23,30 | 23,45 | 13,61 | 0.204645948807374 | 0.294288012872084 | 0.0896420640647099 | 2.834932e-02 | 1.364213e-01 |
| WBGene00022348 | Y82E9BR.16 | --- | III | - | 1421400 | 1421868 | 1421400 | 1421781 | 1424267 | 1424359 | 210 | 124 | 8,174 | 3,129 | 1,150 | 0,174 | 4,217 | 1,161 | 0.0154008569831708 | 0.00532006178136262 | -0.0100807952018082 | 4.794446e-02 | 1.933316e-01 |
| WBGene00007007 | mak-2 | --- | IV | + | 886441 | 886613 | 886495 | 886613 | 885345 | 885421 | 177 | 124 | 19,471 | 37,292 | 52,335 | 25,183 | 42,196 | 55,439 | 0.0644658175201456 | 0.0946008479557762 | 0.0301350304356307 | 3.105096e-03 | 2.575751e-02 |
| WBGene00012128 | nra-1 | --- | IV | - | 17283003 | 17283115 | 17283003 | 17283079 | 17283234 | 17283276 | 159 | 124 | 38,142 | 19,111 | 42,94 | 29,150 | 32,177 | 32,146 | 0.182004180936708 | 0.132950575865528 | -0.0490536050711801 | 2.363525e-02 | 1.210959e-01 |
| WBGene00000059 | acr-20 | --- | II | - | 14374438 | 14374550 | 14374438 | 14374541 | 14374938 | 14375356 | 132 | 124 | 19,2 | 5,2 | 9,1 | 0,3 | 7,3 | 9,11 | 0.861111111111111 | 0.469252601702933 | -0.391858509408178 | 7.083216e-04 | 8.078939e-03 |
| WBGene00019831 | R02F2.1 | --- | III | + | 5505247 | 5505401 | 5505253 | 5505401 | 5504786 | 5505202 | 129 | 124 | 759,3229 | 577,2078 | 586,2428 | 596,1882 | 767,2066 | 602,2838 | 0.192799700358292 | 0.217737481837338 | 0.0249377814790458 | 2.321667e-05 | 5.185056e-04 |
| WBGene00018950 | F56C9.6 | --- | III | - | 7310162 | 7310277 | 7310162 | 7310238 | 7310357 | 7310422 | 162 | 124 | 144,180 | 112,166 | 139,172 | 200,229 | 245,232 | 142,123 | 0.36855887310378 | 0.434825204903343 | 0.0662663317995626 | 1.962339e-03 | 1.837846e-02 |
| WBGene00011488 | nra-2 | --- | I | + | 9621325 | 9621458 | 9621334 | 9621458 | 9620739 | 9621283 | 132 | 124 | 153,191 | 125,152 | 132,223 | 229,201 | 222,241 | 118,179 | 0.404931821078119 | 0.462577362844855 | 0.0576455417667357 | 7.160150e-03 | 4.689091e-02 |
| WBGene00017988 | fipp-1 | --- | V | + | 4372936 | 4373811 | 4372951 | 4373811 | 4372674 | 4372893 | 138 | 124 | 174,125 | 228,113 | 168,164 | 192,110 | 308,140 | 161,110 | 0.560258727290022 | 0.622618577375346 | 0.0623598500853239 | 5.653566e-03 | 3.971174e-02 |

| GeneID | Symbol | description | chr | strand | longExonStart\_0base | longExonEnd | shortES | shortEE | flankingES | flankingEE | IncFormLen | SkipFormLen | op50\_1 | op50\_2 | op50\_3 | S20mg\_1 | S20mg\_2 | S20mg\_3 | G1\_IncLevel | G2\_IncLevel | IncLevelDifference | PValue | FDR |
| --- | --- | --- | --- | --- | --- | --- | --- | --- | --- | --- | --- | --- | --- | --- | --- | --- | --- | --- | --- | --- | --- | --- | --- |
| WBGene00016074 | dnsn-1 | --- | II | - | 410475 | 410581 | 410475 | 410575 | 410624 | 410704 | 129 | 124 | 65,104 | 51,96 | 34,95 | 62,76 | 104,128 | 54,110 | 0.328302885888271 | 0.402442982326734 | 0.074140096438463 | 1.721735e-02 | 9.431641e-02 |
| WBGene00018371 | ess-2 | nucleus | III | - | 8488343 | 8488819 | 8488343 | 8488813 | 8488940 | 8489536 | 129 | 124 | 57,70 | 49,68 | 37,63 | 85,75 | 85,93 | 46,50 | 0.406129039646366 | 0.487815539285324 | 0.0816864996389577 | 2.483565e-02 | 1.254223e-01 |
| WBGene00003229 | mex-3 | --- | I | + | 129166 | 129333 | 129175 | 129333 | 128944 | 129124 | 132 | 124 | 483,79 | 437,69 | 496,73 | 684,37 | 827,58 | 486,78 | 0.857527984527926 | 0.915567321344061 | 0.0580393368161346 | 3.719193e-08 | 2.313870e-06 |
| WBGene00009065 | F22G12.5 | --- | I | + | 13168321 | 13168707 | 13168327 | 13168707 | 13166923 | 13167566 | 129 | 124 | 33,48 | 41,39 | 44,41 | 57,27 | 50,32 | 33,32 | 0.469817621371693 | 0.596584075054126 | 0.126766453682433 | 5.922633e-03 | 4.126891e-02 |
| WBGene00009678 | magu-4 | --- | IV | + | 10011335 | 10011583 | 10011341 | 10011583 | 10011006 | 10011292 | 129 | 124 | 38,19 | 37,12 | 28,12 | 31,3 | 34,13 | 60,8 | 0.697199628800699 | 0.833512583351258 | 0.13631295455056 | 8.048879e-03 | 5.231771e-02 |
| WBGene00008274 | C53B4.4 | --- | IV | + | 8983693 | 8983848 | 8983702 | 8983848 | 8983469 | 8983647 | 132 | 124 | 187,433 | 152,237 | 193,311 | 221,170 | 240,239 | 231,318 | 0.337501279034073 | 0.472063904231675 | 0.134562625197601 | 8.297448e-14 | 2.409026e-11 |
| WBGene00000894 | dab-1 | --- | II | + | 8227552 | 8228193 | 8227558 | 8228193 | 8227056 | 8227476 | 129 | 124 | 237,498 | 238,359 | 189,429 | 298,329 | 331,387 | 230,409 | 0.33169238206502 | 0.423285553625999 | 0.0915931715609786 | 3.032679e-09 | 2.416399e-07 |
| WBGene00000464 | ceh-44 | --- | III | + | 2544834 | 2544895 | 2544840 | 2544895 | 2544361 | 2544645 | 129 | 124 | 71,45 | 78,33 | 62,40 | 100,25 | 94,20 | 88,33 | 0.632194461895327 | 0.776548967355097 | 0.14435450545977 | 4.678487e-05 | 9.261278e-04 |
| WBGene00008989 | F20G2.6 | --- | V | + | 13765225 | 13765481 | 13765228 | 13765481 | 13764566 | 13764711 | 126 | 124 | 31,33 | 8,3 | 22,6 | 6,1 | 10,1 | 13,6 | 0.588363410080896 | 0.781059947871416 | 0.19269653779052 | 4.565180e-02 | 1.884489e-01 |
| WBGene00007016 | mdt-15 | --- | III | - | 5832293 | 5832784 | 5832293 | 5832775 | 5832834 | 5833058 | 132 | 124 | 499,402 | 307,256 | 429,333 | 305,286 | 302,318 | 404,319 | 0.539316504197893 | 0.507135922330097 | -0.0321805818677955 | 3.959880e-02 | 1.741947e-01 |
| … | … | … | … | … | … | … | … | … | … | … | … | … | … | … | … | … | … | … | … | … | … | … | … |

| GeneID | Symbol | description | chr | strand | longExonStart\_0base | longExonEnd | shortES | shortEE | flankingES | flankingEE | IncFormLen | SkipFormLen | op50\_1 | op50\_2 | op50\_3 | S20mg\_1 | S20mg\_2 | S20mg\_3 | G1\_IncLevel | G2\_IncLevel | IncLevelDifference | PValue | FDR |
| --- | --- | --- | --- | --- | --- | --- | --- | --- | --- | --- | --- | --- | --- | --- | --- | --- | --- | --- | --- | --- | --- | --- | --- |
| WBGene00016074 | dnsn-1 | --- | II | - | 410475 | 410581 | 410475 | 410575 | 410624 | 410704 | 129 | 124 | 65,104 | 51,96 | 34,95 | 62,76 | 104,128 | 54,110 | 0.328302885888271 | 0.402442982326734 | 0.074140096438463 | 1.721735e-02 | 9.431641e-02 |
| WBGene00018371 | ess-2 | nucleus | III | - | 8488343 | 8488819 | 8488343 | 8488813 | 8488940 | 8489536 | 129 | 124 | 57,70 | 49,68 | 37,63 | 85,75 | 85,93 | 46,50 | 0.406129039646366 | 0.487815539285324 | 0.0816864996389577 | 2.483565e-02 | 1.254223e-01 |
| WBGene00003229 | mex-3 | --- | I | + | 129166 | 129333 | 129175 | 129333 | 128944 | 129124 | 132 | 124 | 483,79 | 437,69 | 496,73 | 684,37 | 827,58 | 486,78 | 0.857527984527926 | 0.915567321344061 | 0.0580393368161346 | 3.719193e-08 | 2.313870e-06 |
| WBGene00009065 | F22G12.5 | --- | I | + | 13168321 | 13168707 | 13168327 | 13168707 | 13166923 | 13167566 | 129 | 124 | 33,48 | 41,39 | 44,41 | 57,27 | 50,32 | 33,32 | 0.469817621371693 | 0.596584075054126 | 0.126766453682433 | 5.922633e-03 | 4.126891e-02 |
| WBGene00009678 | magu-4 | --- | IV | + | 10011335 | 10011583 | 10011341 | 10011583 | 10011006 | 10011292 | 129 | 124 | 38,19 | 37,12 | 28,12 | 31,3 | 34,13 | 60,8 | 0.697199628800699 | 0.833512583351258 | 0.13631295455056 | 8.048879e-03 | 5.231771e-02 |
| WBGene00008274 | C53B4.4 | --- | IV | + | 8983693 | 8983848 | 8983702 | 8983848 | 8983469 | 8983647 | 132 | 124 | 187,433 | 152,237 | 193,311 | 221,170 | 240,239 | 231,318 | 0.337501279034073 | 0.472063904231675 | 0.134562625197601 | 8.297448e-14 | 2.409026e-11 |
| WBGene00000894 | dab-1 | --- | II | + | 8227552 | 8228193 | 8227558 | 8228193 | 8227056 | 8227476 | 129 | 124 | 237,498 | 238,359 | 189,429 | 298,329 | 331,387 | 230,409 | 0.33169238206502 | 0.423285553625999 | 0.0915931715609786 | 3.032679e-09 | 2.416399e-07 |
| WBGene00000464 | ceh-44 | --- | III | + | 2544834 | 2544895 | 2544840 | 2544895 | 2544361 | 2544645 | 129 | 124 | 71,45 | 78,33 | 62,40 | 100,25 | 94,20 | 88,33 | 0.632194461895327 | 0.776548967355097 | 0.14435450545977 | 4.678487e-05 | 9.261278e-04 |
| WBGene00008989 | F20G2.6 | --- | V | + | 13765225 | 13765481 | 13765228 | 13765481 | 13764566 | 13764711 | 126 | 124 | 31,33 | 8,3 | 22,6 | 6,1 | 10,1 | 13,6 | 0.588363410080896 | 0.781059947871416 | 0.19269653779052 | 4.565180e-02 | 1.884489e-01 |
| WBGene00007016 | mdt-15 | --- | III | - | 5832293 | 5832784 | 5832293 | 5832775 | 5832834 | 5833058 | 132 | 124 | 499,402 | 307,256 | 429,333 | 305,286 | 302,318 | 404,319 | 0.539316504197893 | 0.507135922330097 | -0.0321805818677955 | 3.959880e-02 | 1.741947e-01 |
| … | … | … | … | … | … | … | … | … | … | … | … | … | … | … | … | … | … | … | … | … | … | … | … |

… … … … … … … … … … … … … … … … … … … … … … … …

  
  

| 表头 | 表头说明 |
| --- | --- |
| ID | 事件编号 |
| GeneID | 基因标识 |
| Symbol | 基因简称 |
| description | 基因功能描述信息 |
| Chr | 染色体 |
| Strand | 链方向 |
| exonStart\_0base | 被跳跃外显子开始位置(0-based) |
| exonEnd | 被跳跃外显子结束位置(1-based) |
| upstreamES | 上游外显子的开始位置(0-based) |
| upstreamEE | 上游外显子的结束位置(1-based) |
| downstreamES | 下游外显子的开始位置(0-based) |
| downstreamEE | 下游外显子的结束位置(1-based) |
| IJC\_SAMPLE\_1,SJC\_SAMPLE\_1 | 样本组1的支持包含被跳跃外显子的Junction序列数;样本组1的支持不包含被跳跃外显子的Junction序列数 |
| IJC\_SAMPLE\_2,SJC\_SAMPLE\_2 | 样本组2的支持包含被跳跃外显子的Junction序列数;样本组2的支持不包含被跳跃外显子的Junction序列数 |
| IncFormLen | 被跳跃外显子与上下游外显子连接区域长度 |
| SkipFormLen | 被跳跃外显子的上下游外显子连接区域长度 |
| PValue | 样本组2相比于样本组1差异的被跳跃外显子包含率的显著性概率值 |
| FDR | Pvalue多重检验后的假阳性率 |
| IncLevel1 | 样本组1的被跳跃外显子包含比率(标准化后) |
| IncLevel2 | 样本组2的被跳跃外显子包含比率(标准化后) |
| IncLevelDifference | 样本组2相比于样本组1被跳跃外显子包含比率之差 |

  
  

  


## 7.2 KEGG富集分析

目录链接

  
  
  
  

KEGG pathway功能分析是针对这些基因进行KEGG数据库中Pathway的功能注释和归类，KEGG pathway功能富集分析方法与GO功能富集分析类似。为了方便相关基因的功能查找起见，将代谢通路信息与每个基因差异表达信息整合为一个表格，并将命名为 KEGG\_enrichment\_significant\_and\_gene\_exp\_significant.xls。

全部差异基因的KEGG功能分析结果见报告-> 6-FUN > \*\_vs\_\* > GO\_KEGG/KEGG\*

上调的差异基因的KEGG功能分析结果见报告-> 6-FUN > \*\_vs\_\* > GO\_KEGG\_Up/KEGG\*

下调的差异基因的KEGG功能分析结果见报告-> 6-FUN > \*\_vs\_\* > GO\_KEGG\_Down/KEGG\*

### KEGG富集分析结果(KEGG\_enrichment\_significant.xls)

显示 1020全文 行  
FUN-S20mg\_vs\_op50-GO\_KEGG
FUN-S20mg\_vs\_op50-GO\_KEGG\_Down
FUN-S20mg\_vs\_op50-GO\_KEGG\_Up
 

| Pathway\_id | Pathway\_name | Pathway\_class | Annotated | Significant | SymbolList | Expected | classic\_fisher | FDR | WebSite | rich\_factor |
| --- | --- | --- | --- | --- | --- | --- | --- | --- | --- | --- |
| ko05168 | Herpes simplex infection | Infectious diseases Viral | 122 | 6 | csp-3,ZC477.2,T28F4.3,F38E11.8,trf-1,sdf-9 | 1.17773992049972 | 0.0009263937 | 0.1120936 | http://www.kegg.jp/kegg-bin/show\_pathway?ko05168/ | 0.0491803278688525 |
| ko05205 | Proteoglycans in cancer | Cancers Overview | 126 | 5 | csp-3,ZC477.2,T28F4.3,F38E11.8,sdf-9 | 1.21635434412266 | 0.0065425582 | 0.2208139 | http://www.kegg.jp/kegg-bin/show\_pathway?ko05205/ | 0.0396825396825397 |
| ko04920 | Adipocytokine signaling pathway | Endocrine system | 42 | 3 | T28F4.3,acs-23,sdf-9 | 0.405451448040886 | 0.0072996343 | 0.2208139 | http://www.kegg.jp/kegg-bin/show\_pathway?ko04920/ | 0.0714285714285714 |
| ko04650 | Natural killer cell mediated cytotoxicity | Immune system | 34 | 3 | csp-3,T28F4.3,sdf-9 | 0.328222600795003 | 0.004010468 | 0.2208139 | http://www.kegg.jp/kegg-bin/show\_pathway?ko04650/ | 0.0882352941176471 |
| ko04013 | MAPK signaling pathway - fly | Signal transduction | 16 | 2 | T28F4.3,sdf-9 | 0.154457694491766 | 0.009974506 | 0.241383 | http://www.kegg.jp/kegg-bin/show\_pathway?ko04013/ | 0.125 |
| ko04730 | Long-term depression | Nervous system | 28 | 2 | gcy-36,gpa-2 | 0.270300965360591 | 0.0292328348 | 0.2526552 | http://www.kegg.jp/kegg-bin/show\_pathway?ko04730/ | 0.0714285714285714 |
| ko04630 | Jak-STAT signaling pathway | Signal transduction | 28 | 2 | T28F4.3,sdf-9 | 0.270300965360591 | 0.0292328348 | 0.2526552 | http://www.kegg.jp/kegg-bin/show\_pathway?ko04630/ | 0.0714285714285714 |
| ko04270 | Vascular smooth muscle contraction | Circulatory system | 64 | 3 | ZC477.2,F38E11.8,gcy-36 | 0.617830777967064 | 0.0229376575 | 0.2526552 | http://www.kegg.jp/kegg-bin/show\_pathway?ko04270/ | 0.046875 |
| ko05133 | Pertussis | Infectious diseases Bacterial | 27 | 2 | csp-3,trf-1 | 0.260647359454855 | 0.0273079236 | 0.2526552 | http://www.kegg.jp/kegg-bin/show\_pathway?ko05133/ | 0.0740740740740741 |
| ko05146 | Amoebiasis | Infectious diseases Parasitic | 22 | 2 | csp-3,srp-10 | 0.212379329926178 | 0.0185194877 | 0.2526552 | http://www.kegg.jp/kegg-bin/show\_pathway?ko05146/ | 0.0909090909090909 |
| ko04722 | Neurotrophin signaling pathway | Nervous system | 67 | 3 | T28F4.3,trf-1,sdf-9 | 0.64679159568427 | 0.0258622521 | 0.2526552 | http://www.kegg.jp/kegg-bin/show\_pathway?ko04722/ | 0.0447761194029851 |
| ko05145 | Toxoplasmosis | Infectious diseases Parasitic | 52 | 3 | csp-3,trf-1,gpa-2 | 0.50198750709824 | 0.0131602259 | 0.2526552 | http://www.kegg.jp/kegg-bin/show\_pathway?ko05145/ | 0.0576923076923077 |
| ko00230 | Purine metabolism | Nucleotide metabolism | 118 | 4 | gcy-6,gcy-5,gcy-29,gcy-36 | 1.13912549687677 | 0.0255647488 | 0.2526552 | http://www.kegg.jp/kegg-bin/show\_pathway?ko00230/ | 0.0338983050847458 |
| ko05120 | Epithelial cell signaling in Helicobacter pylori infection | Infectious diseases Bacterial | 59 | 3 | csp-3,T28F4.3,sdf-9 | 0.569562748438387 | 0.0184917918 | 0.2526552 | http://www.kegg.jp/kegg-bin/show\_pathway?ko05120/ | 0.0508474576271186 |
| ko05032 | Morphine addiction | Substance dependence | 34 | 2 | gpa-2,lgc-36 | 0.328222600795003 | 0.0418562542 | 0.3095851 | http://www.kegg.jp/kegg-bin/show\_pathway?ko05032/ | 0.0588235294117647 |
| ko05220 | Chronic myeloid leukemia | Cancers Specific types | 34 | 2 | T28F4.3,sdf-9 | 0.328222600795003 | 0.0418562542 | 0.3095851 | http://www.kegg.jp/kegg-bin/show\_pathway?ko05220/ | 0.0588235294117647 |
| ko04713 | Circadian entrainment | Environmental adaptation | 35 | 2 | gcy-36,gpa-2 | 0.337876206700738 | 0.0441288952 | 0.3095851 | http://www.kegg.jp/kegg-bin/show\_pathway?ko04713/ | 0.0571428571428571 |
| ko04728 | Dopaminergic synapse | Nervous system | 84 | 3 | ZC477.2,F38E11.8,gpa-2 | 0.810902896081772 | 0.0460539752 | 0.3095851 | http://www.kegg.jp/kegg-bin/show\_pathway?ko04728/ | 0.0357142857142857 |

| Pathway\_id | Pathway\_name | Pathway\_class | Annotated | Significant | SymbolList | Expected | classic\_fisher | FDR | WebSite | rich\_factor |
| --- | --- | --- | --- | --- | --- | --- | --- | --- | --- | --- |
| ko00230 | Purine metabolism | Nucleotide metabolism | 118 | 4 | gcy-6,gcy-5,gcy-29,gcy-36 | 0.703577512776831 | 0.004606629 | 0.2234215 | http://www.kegg.jp/kegg-bin/show\_pathway?ko00230/ | 0.0338983050847458 |
| ko05145 | Toxoplasmosis | Infectious diseases Parasitic | 52 | 3 | csp-3,trf-1,gpa-2 | 0.310051107325383 | 0.00334713 | 0.2234215 | http://www.kegg.jp/kegg-bin/show\_pathway?ko05145/ | 0.0576923076923077 |
| ko04650 | Natural killer cell mediated cytotoxicity | Immune system | 34 | 2 | csp-3,sdf-9 | 0.202725724020443 | 0.016937449 | 0.2480406 | http://www.kegg.jp/kegg-bin/show\_pathway?ko04650/ | 0.0588235294117647 |
| ko04713 | Circadian entrainment | Environmental adaptation | 35 | 2 | gcy-36,gpa-2 | 0.208688245315162 | 0.017899834 | 0.2480406 | http://www.kegg.jp/kegg-bin/show\_pathway?ko04713/ | 0.0571428571428571 |
| ko04730 | Long-term depression | Nervous system | 28 | 2 | gcy-36,gpa-2 | 0.166950596252129 | 0.011660148 | 0.2480406 | http://www.kegg.jp/kegg-bin/show\_pathway?ko04730/ | 0.0714285714285714 |
| ko05032 | Morphine addiction | Substance dependence | 34 | 2 | gpa-2,lgc-36 | 0.202725724020443 | 0.016937449 | 0.2480406 | http://www.kegg.jp/kegg-bin/show\_pathway?ko05032/ | 0.0588235294117647 |
| ko05133 | Pertussis | Infectious diseases Bacterial | 27 | 2 | csp-3,trf-1 | 0.160988074957411 | 0.010866141 | 0.2480406 | http://www.kegg.jp/kegg-bin/show\_pathway?ko05133/ | 0.0740740740740741 |
| ko05142 | Chagas disease (American trypanosomiasis) | Infectious diseases Parasitic | 41 | 2 | trf-1,gpa-2 | 0.244463373083475 | 0.024145484 | 0.292764 | http://www.kegg.jp/kegg-bin/show\_pathway?ko05142/ | 0.0487804878048781 |
| ko05168 | Herpes simplex infection | Infectious diseases Viral | 122 | 3 | csp-3,trf-1,sdf-9 | 0.727427597955707 | 0.03420393 | 0.3312379 | http://www.kegg.jp/kegg-bin/show\_pathway?ko05168/ | 0.0245901639344262 |
| ko00281 | Geraniol degradation | Metabolism of terpenoids and polyketides | 8 | 1 | ech-9 | 0.0477001703577513 | 0.046762029 | 0.3312379 | http://www.kegg.jp/kegg-bin/show\_pathway?ko00281/ | 0.125 |
| ko04726 | Serotonergic synapse | Nervous system | 54 | 2 | csp-3,gpa-2 | 0.321976149914821 | 0.040229235 | 0.3312379 | http://www.kegg.jp/kegg-bin/show\_pathway?ko04726/ | 0.037037037037037 |
| ko05120 | Epithelial cell signaling in Helicobacter pylori infection | Infectious diseases Bacterial | 59 | 2 | csp-3,sdf-9 | 0.351788756388416 | 0.047253388 | 0.3312379 | http://www.kegg.jp/kegg-bin/show\_pathway?ko05120/ | 0.0338983050847458 |
| ko04727 | GABAergic synapse | Nervous system | 51 | 2 | gpa-2,lgc-36 | 0.304088586030664 | 0.036228389 | 0.3312379 | http://www.kegg.jp/kegg-bin/show\_pathway?ko04727/ | 0.0392156862745098 |

| Pathway\_id | Pathway\_name | Pathway\_class | Annotated | Significant | SymbolList | Expected | classic\_fisher | FDR | WebSite | rich\_factor |
| --- | --- | --- | --- | --- | --- | --- | --- | --- | --- | --- |
| ko05205 | Proteoglycans in cancer | Cancers Overview | 126 | 3 | ZC477.2,T28F4.3,F38E11.8 | 0.465076660988075 | 0.009829806 | 0.1069032 | http://www.kegg.jp/kegg-bin/show\_pathway?ko05205/ | 0.0238095238095238 |
| ko04720 | Long-term potentiation | Nervous system | 50 | 2 | ZC477.2,F38E11.8 | 0.184554230550823 | 0.013943896 | 0.1069032 | http://www.kegg.jp/kegg-bin/show\_pathway?ko04720/ | 0.04 |
| ko04920 | Adipocytokine signaling pathway | Endocrine system | 42 | 2 | T28F4.3,acs-23 | 0.155025553662692 | 0.00996506 | 0.1069032 | http://www.kegg.jp/kegg-bin/show\_pathway?ko04920/ | 0.0476190476190476 |
| ko05168 | Herpes simplex infection | Infectious diseases Viral | 122 | 3 | ZC477.2,T28F4.3,F38E11.8 | 0.450312322544009 | 0.008993111 | 0.1069032 | http://www.kegg.jp/kegg-bin/show\_pathway?ko05168/ | 0.0245901639344262 |
| ko05031 | Amphetamine addiction | Substance dependence | 47 | 2 | ZC477.2,F38E11.8 | 0.173480976717774 | 0.012381809 | 0.1069032 | http://www.kegg.jp/kegg-bin/show\_pathway?ko05031/ | 0.0425531914893617 |
| ko04910 | Insulin signaling pathway | Endocrine system | 98 | 3 | ZC477.2,F38E11.8,F55H12.5 | 0.361726291879614 | 0.004878379 | 0.1069032 | http://www.kegg.jp/kegg-bin/show\_pathway?ko04910/ | 0.0306122448979592 |
| ko04270 | Vascular smooth muscle contraction | Circulatory system | 64 | 2 | ZC477.2,F38E11.8 | 0.236229415105054 | 0.022289328 | 0.1319578 | http://www.kegg.jp/kegg-bin/show\_pathway?ko04270/ | 0.03125 |
| ko04113 | Meiosis - yeast | Cell growth and death | 65 | 2 | ZC477.2,F38E11.8 | 0.23992049971607 | 0.022949184 | 0.1319578 | http://www.kegg.jp/kegg-bin/show\_pathway?ko04113/ | 0.0307692307692308 |
| ko03015 | mRNA surveillance pathway | Translation | 82 | 2 | ZC477.2,F38E11.8 | 0.30266893810335 | 0.035370325 | 0.154618 | http://www.kegg.jp/kegg-bin/show\_pathway?ko03015/ | 0.024390243902439 |
| ko04390 | Hippo signaling pathway | Signal transduction | 79 | 2 | ZC477.2,F38E11.8 | 0.291595684270301 | 0.033019198 | 0.154618 | http://www.kegg.jp/kegg-bin/show\_pathway?ko04390/ | 0.0253164556962025 |
| ko04728 | Dopaminergic synapse | Nervous system | 84 | 2 | ZC477.2,F38E11.8 | 0.310051107325383 | 0.03697386 | 0.154618 | http://www.kegg.jp/kegg-bin/show\_pathway?ko04728/ | 0.0238095238095238 |

| Pathway\_id | Pathway\_name | Pathway\_class | Annotated | Significant | SymbolList | Expected | classic\_fisher | FDR | WebSite | rich\_factor |
| --- | --- | --- | --- | --- | --- | --- | --- | --- | --- | --- |
| ko05168 | Herpes simplex infection | Infectious diseases Viral | 122 | 6 | csp-3,ZC477.2,T28F4.3,F38E11.8,trf-1,sdf-9 | 1.17773992049972 | 0.0009263937 | 0.1120936 | http://www.kegg.jp/kegg-bin/show\_pathway?ko05168/ | 0.0491803278688525 |
| ko05205 | Proteoglycans in cancer | Cancers Overview | 126 | 5 | csp-3,ZC477.2,T28F4.3,F38E11.8,sdf-9 | 1.21635434412266 | 0.0065425582 | 0.2208139 | http://www.kegg.jp/kegg-bin/show\_pathway?ko05205/ | 0.0396825396825397 |
| ko04920 | Adipocytokine signaling pathway | Endocrine system | 42 | 3 | T28F4.3,acs-23,sdf-9 | 0.405451448040886 | 0.0072996343 | 0.2208139 | http://www.kegg.jp/kegg-bin/show\_pathway?ko04920/ | 0.0714285714285714 |
| ko04650 | Natural killer cell mediated cytotoxicity | Immune system | 34 | 3 | csp-3,T28F4.3,sdf-9 | 0.328222600795003 | 0.004010468 | 0.2208139 | http://www.kegg.jp/kegg-bin/show\_pathway?ko04650/ | 0.0882352941176471 |
| ko04013 | MAPK signaling pathway - fly | Signal transduction | 16 | 2 | T28F4.3,sdf-9 | 0.154457694491766 | 0.009974506 | 0.241383 | http://www.kegg.jp/kegg-bin/show\_pathway?ko04013/ | 0.125 |
| ko04730 | Long-term depression | Nervous system | 28 | 2 | gcy-36,gpa-2 | 0.270300965360591 | 0.0292328348 | 0.2526552 | http://www.kegg.jp/kegg-bin/show\_pathway?ko04730/ | 0.0714285714285714 |
| ko04630 | Jak-STAT signaling pathway | Signal transduction | 28 | 2 | T28F4.3,sdf-9 | 0.270300965360591 | 0.0292328348 | 0.2526552 | http://www.kegg.jp/kegg-bin/show\_pathway?ko04630/ | 0.0714285714285714 |
| ko04270 | Vascular smooth muscle contraction | Circulatory system | 64 | 3 | ZC477.2,F38E11.8,gcy-36 | 0.617830777967064 | 0.0229376575 | 0.2526552 | http://www.kegg.jp/kegg-bin/show\_pathway?ko04270/ | 0.046875 |
| ko05133 | Pertussis | Infectious diseases Bacterial | 27 | 2 | csp-3,trf-1 | 0.260647359454855 | 0.0273079236 | 0.2526552 | http://www.kegg.jp/kegg-bin/show\_pathway?ko05133/ | 0.0740740740740741 |
| ko05146 | Amoebiasis | Infectious diseases Parasitic | 22 | 2 | csp-3,srp-10 | 0.212379329926178 | 0.0185194877 | 0.2526552 | http://www.kegg.jp/kegg-bin/show\_pathway?ko05146/ | 0.0909090909090909 |
| … | … | … | … | … | … | … | … | … | … | … |

| Pathway\_id | Pathway\_name | Pathway\_class | Annotated | Significant | SymbolList | Expected | classic\_fisher | FDR | WebSite | rich\_factor |
| --- | --- | --- | --- | --- | --- | --- | --- | --- | --- | --- |
| ko00230 | Purine metabolism | Nucleotide metabolism | 118 | 4 | gcy-6,gcy-5,gcy-29,gcy-36 | 0.703577512776831 | 0.004606629 | 0.2234215 | http://www.kegg.jp/kegg-bin/show\_pathway?ko00230/ | 0.0338983050847458 |
| ko05145 | Toxoplasmosis | Infectious diseases Parasitic | 52 | 3 | csp-3,trf-1,gpa-2 | 0.310051107325383 | 0.00334713 | 0.2234215 | http://www.kegg.jp/kegg-bin/show\_pathway?ko05145/ | 0.0576923076923077 |
| ko04650 | Natural killer cell mediated cytotoxicity | Immune system | 34 | 2 | csp-3,sdf-9 | 0.202725724020443 | 0.016937449 | 0.2480406 | http://www.kegg.jp/kegg-bin/show\_pathway?ko04650/ | 0.0588235294117647 |
| ko04713 | Circadian entrainment | Environmental adaptation | 35 | 2 | gcy-36,gpa-2 | 0.208688245315162 | 0.017899834 | 0.2480406 | http://www.kegg.jp/kegg-bin/show\_pathway?ko04713/ | 0.0571428571428571 |
| ko04730 | Long-term depression | Nervous system | 28 | 2 | gcy-36,gpa-2 | 0.166950596252129 | 0.011660148 | 0.2480406 | http://www.kegg.jp/kegg-bin/show\_pathway?ko04730/ | 0.0714285714285714 |
| ko05032 | Morphine addiction | Substance dependence | 34 | 2 | gpa-2,lgc-36 | 0.202725724020443 | 0.016937449 | 0.2480406 | http://www.kegg.jp/kegg-bin/show\_pathway?ko05032/ | 0.0588235294117647 |
| ko05133 | Pertussis | Infectious diseases Bacterial | 27 | 2 | csp-3,trf-1 | 0.160988074957411 | 0.010866141 | 0.2480406 | http://www.kegg.jp/kegg-bin/show\_pathway?ko05133/ | 0.0740740740740741 |
| ko05142 | Chagas disease (American trypanosomiasis) | Infectious diseases Parasitic | 41 | 2 | trf-1,gpa-2 | 0.244463373083475 | 0.024145484 | 0.292764 | http://www.kegg.jp/kegg-bin/show\_pathway?ko05142/ | 0.0487804878048781 |
| ko05168 | Herpes simplex infection | Infectious diseases Viral | 122 | 3 | csp-3,trf-1,sdf-9 | 0.727427597955707 | 0.03420393 | 0.3312379 | http://www.kegg.jp/kegg-bin/show\_pathway?ko05168/ | 0.0245901639344262 |
| ko00281 | Geraniol degradation | Metabolism of terpenoids and polyketides | 8 | 1 | ech-9 | 0.0477001703577513 | 0.046762029 | 0.3312379 | http://www.kegg.jp/kegg-bin/show\_pathway?ko00281/ | 0.125 |
| … | … | … | … | … | … | … | … | … | … | … |

| Pathway\_id | Pathway\_name | Pathway\_class | Annotated | Significant | SymbolList | Expected | classic\_fisher | FDR | WebSite | rich\_factor |
| --- | --- | --- | --- | --- | --- | --- | --- | --- | --- | --- |
| ko05205 | Proteoglycans in cancer | Cancers Overview | 126 | 3 | ZC477.2,T28F4.3,F38E11.8 | 0.465076660988075 | 0.009829806 | 0.1069032 | http://www.kegg.jp/kegg-bin/show\_pathway?ko05205/ | 0.0238095238095238 |
| ko04720 | Long-term potentiation | Nervous system | 50 | 2 | ZC477.2,F38E11.8 | 0.184554230550823 | 0.013943896 | 0.1069032 | http://www.kegg.jp/kegg-bin/show\_pathway?ko04720/ | 0.04 |
| ko04920 | Adipocytokine signaling pathway | Endocrine system | 42 | 2 | T28F4.3,acs-23 | 0.155025553662692 | 0.00996506 | 0.1069032 | http://www.kegg.jp/kegg-bin/show\_pathway?ko04920/ | 0.0476190476190476 |
| ko05168 | Herpes simplex infection | Infectious diseases Viral | 122 | 3 | ZC477.2,T28F4.3,F38E11.8 | 0.450312322544009 | 0.008993111 | 0.1069032 | http://www.kegg.jp/kegg-bin/show\_pathway?ko05168/ | 0.0245901639344262 |
| ko05031 | Amphetamine addiction | Substance dependence | 47 | 2 | ZC477.2,F38E11.8 | 0.173480976717774 | 0.012381809 | 0.1069032 | http://www.kegg.jp/kegg-bin/show\_pathway?ko05031/ | 0.0425531914893617 |
| ko04910 | Insulin signaling pathway | Endocrine system | 98 | 3 | ZC477.2,F38E11.8,F55H12.5 | 0.361726291879614 | 0.004878379 | 0.1069032 | http://www.kegg.jp/kegg-bin/show\_pathway?ko04910/ | 0.0306122448979592 |
| ko04270 | Vascular smooth muscle contraction | Circulatory system | 64 | 2 | ZC477.2,F38E11.8 | 0.236229415105054 | 0.022289328 | 0.1319578 | http://www.kegg.jp/kegg-bin/show\_pathway?ko04270/ | 0.03125 |
| ko04113 | Meiosis - yeast | Cell growth and death | 65 | 2 | ZC477.2,F38E11.8 | 0.23992049971607 | 0.022949184 | 0.1319578 | http://www.kegg.jp/kegg-bin/show\_pathway?ko04113/ | 0.0307692307692308 |
| ko03015 | mRNA surveillance pathway | Translation | 82 | 2 | ZC477.2,F38E11.8 | 0.30266893810335 | 0.035370325 | 0.154618 | http://www.kegg.jp/kegg-bin/show\_pathway?ko03015/ | 0.024390243902439 |
| ko04390 | Hippo signaling pathway | Signal transduction | 79 | 2 | ZC477.2,F38E11.8 | 0.291595684270301 | 0.033019198 | 0.154618 | http://www.kegg.jp/kegg-bin/show\_pathway?ko04390/ | 0.0253164556962025 |
| … | … | … | … | … | … | … | … | … | … | … |

| Pathway\_id | Pathway\_name | Pathway\_class | Annotated | Significant | SymbolList | Expected | classic\_fisher | FDR | WebSite | rich\_factor |
| --- | --- | --- | --- | --- | --- | --- | --- | --- | --- | --- |
| ko05168 | Herpes simplex infection | Infectious diseases Viral | 122 | 6 | csp-3,ZC477.2,T28F4.3,F38E11.8,trf-1,sdf-9 | 1.17773992049972 | 0.0009263937 | 0.1120936 | http://www.kegg.jp/kegg-bin/show\_pathway?ko05168/ | 0.0491803278688525 |
| ko05205 | Proteoglycans in cancer | Cancers Overview | 126 | 5 | csp-3,ZC477.2,T28F4.3,F38E11.8,sdf-9 | 1.21635434412266 | 0.0065425582 | 0.2208139 | http://www.kegg.jp/kegg-bin/show\_pathway?ko05205/ | 0.0396825396825397 |
| ko04920 | Adipocytokine signaling pathway | Endocrine system | 42 | 3 | T28F4.3,acs-23,sdf-9 | 0.405451448040886 | 0.0072996343 | 0.2208139 | http://www.kegg.jp/kegg-bin/show\_pathway?ko04920/ | 0.0714285714285714 |
| ko04650 | Natural killer cell mediated cytotoxicity | Immune system | 34 | 3 | csp-3,T28F4.3,sdf-9 | 0.328222600795003 | 0.004010468 | 0.2208139 | http://www.kegg.jp/kegg-bin/show\_pathway?ko04650/ | 0.0882352941176471 |
| ko04013 | MAPK signaling pathway - fly | Signal transduction | 16 | 2 | T28F4.3,sdf-9 | 0.154457694491766 | 0.009974506 | 0.241383 | http://www.kegg.jp/kegg-bin/show\_pathway?ko04013/ | 0.125 |
| ko04730 | Long-term depression | Nervous system | 28 | 2 | gcy-36,gpa-2 | 0.270300965360591 | 0.0292328348 | 0.2526552 | http://www.kegg.jp/kegg-bin/show\_pathway?ko04730/ | 0.0714285714285714 |
| ko04630 | Jak-STAT signaling pathway | Signal transduction | 28 | 2 | T28F4.3,sdf-9 | 0.270300965360591 | 0.0292328348 | 0.2526552 | http://www.kegg.jp/kegg-bin/show\_pathway?ko04630/ | 0.0714285714285714 |
| ko04270 | Vascular smooth muscle contraction | Circulatory system | 64 | 3 | ZC477.2,F38E11.8,gcy-36 | 0.617830777967064 | 0.0229376575 | 0.2526552 | http://www.kegg.jp/kegg-bin/show\_pathway?ko04270/ | 0.046875 |
| ko05133 | Pertussis | Infectious diseases Bacterial | 27 | 2 | csp-3,trf-1 | 0.260647359454855 | 0.0273079236 | 0.2526552 | http://www.kegg.jp/kegg-bin/show\_pathway?ko05133/ | 0.0740740740740741 |
| ko05146 | Amoebiasis | Infectious diseases Parasitic | 22 | 2 | csp-3,srp-10 | 0.212379329926178 | 0.0185194877 | 0.2526552 | http://www.kegg.jp/kegg-bin/show\_pathway?ko05146/ | 0.0909090909090909 |
| … | … | … | … | … | … | … | … | … | … | … |

| Pathway\_id | Pathway\_name | Pathway\_class | Annotated | Significant | SymbolList | Expected | classic\_fisher | FDR | WebSite | rich\_factor |
| --- | --- | --- | --- | --- | --- | --- | --- | --- | --- | --- |
| ko00230 | Purine metabolism | Nucleotide metabolism | 118 | 4 | gcy-6,gcy-5,gcy-29,gcy-36 | 0.703577512776831 | 0.004606629 | 0.2234215 | http://www.kegg.jp/kegg-bin/show\_pathway?ko00230/ | 0.0338983050847458 |
| ko05145 | Toxoplasmosis | Infectious diseases Parasitic | 52 | 3 | csp-3,trf-1,gpa-2 | 0.310051107325383 | 0.00334713 | 0.2234215 | http://www.kegg.jp/kegg-bin/show\_pathway?ko05145/ | 0.0576923076923077 |
| ko04650 | Natural killer cell mediated cytotoxicity | Immune system | 34 | 2 | csp-3,sdf-9 | 0.202725724020443 | 0.016937449 | 0.2480406 | http://www.kegg.jp/kegg-bin/show\_pathway?ko04650/ | 0.0588235294117647 |
| ko04713 | Circadian entrainment | Environmental adaptation | 35 | 2 | gcy-36,gpa-2 | 0.208688245315162 | 0.017899834 | 0.2480406 | http://www.kegg.jp/kegg-bin/show\_pathway?ko04713/ | 0.0571428571428571 |
| ko04730 | Long-term depression | Nervous system | 28 | 2 | gcy-36,gpa-2 | 0.166950596252129 | 0.011660148 | 0.2480406 | http://www.kegg.jp/kegg-bin/show\_pathway?ko04730/ | 0.0714285714285714 |
| ko05032 | Morphine addiction | Substance dependence | 34 | 2 | gpa-2,lgc-36 | 0.202725724020443 | 0.016937449 | 0.2480406 | http://www.kegg.jp/kegg-bin/show\_pathway?ko05032/ | 0.0588235294117647 |
| ko05133 | Pertussis | Infectious diseases Bacterial | 27 | 2 | csp-3,trf-1 | 0.160988074957411 | 0.010866141 | 0.2480406 | http://www.kegg.jp/kegg-bin/show\_pathway?ko05133/ | 0.0740740740740741 |
| ko05142 | Chagas disease (American trypanosomiasis) | Infectious diseases Parasitic | 41 | 2 | trf-1,gpa-2 | 0.244463373083475 | 0.024145484 | 0.292764 | http://www.kegg.jp/kegg-bin/show\_pathway?ko05142/ | 0.0487804878048781 |
| ko05168 | Herpes simplex infection | Infectious diseases Viral | 122 | 3 | csp-3,trf-1,sdf-9 | 0.727427597955707 | 0.03420393 | 0.3312379 | http://www.kegg.jp/kegg-bin/show\_pathway?ko05168/ | 0.0245901639344262 |
| ko00281 | Geraniol degradation | Metabolism of terpenoids and polyketides | 8 | 1 | ech-9 | 0.0477001703577513 | 0.046762029 | 0.3312379 | http://www.kegg.jp/kegg-bin/show\_pathway?ko00281/ | 0.125 |
| … | … | … | … | … | … | … | … | … | … | … |

| Pathway\_id | Pathway\_name | Pathway\_class | Annotated | Significant | SymbolList | Expected | classic\_fisher | FDR | WebSite | rich\_factor |
| --- | --- | --- | --- | --- | --- | --- | --- | --- | --- | --- |
| ko05205 | Proteoglycans in cancer | Cancers Overview | 126 | 3 | ZC477.2,T28F4.3,F38E11.8 | 0.465076660988075 | 0.009829806 | 0.1069032 | http://www.kegg.jp/kegg-bin/show\_pathway?ko05205/ | 0.0238095238095238 |
| ko04720 | Long-term potentiation | Nervous system | 50 | 2 | ZC477.2,F38E11.8 | 0.184554230550823 | 0.013943896 | 0.1069032 | http://www.kegg.jp/kegg-bin/show\_pathway?ko04720/ | 0.04 |
| ko04920 | Adipocytokine signaling pathway | Endocrine system | 42 | 2 | T28F4.3,acs-23 | 0.155025553662692 | 0.00996506 | 0.1069032 | http://www.kegg.jp/kegg-bin/show\_pathway?ko04920/ | 0.0476190476190476 |
| ko05168 | Herpes simplex infection | Infectious diseases Viral | 122 | 3 | ZC477.2,T28F4.3,F38E11.8 | 0.450312322544009 | 0.008993111 | 0.1069032 | http://www.kegg.jp/kegg-bin/show\_pathway?ko05168/ | 0.0245901639344262 |
| ko05031 | Amphetamine addiction | Substance dependence | 47 | 2 | ZC477.2,F38E11.8 | 0.173480976717774 | 0.012381809 | 0.1069032 | http://www.kegg.jp/kegg-bin/show\_pathway?ko05031/ | 0.0425531914893617 |
| ko04910 | Insulin signaling pathway | Endocrine system | 98 | 3 | ZC477.2,F38E11.8,F55H12.5 | 0.361726291879614 | 0.004878379 | 0.1069032 | http://www.kegg.jp/kegg-bin/show\_pathway?ko04910/ | 0.0306122448979592 |
| ko04270 | Vascular smooth muscle contraction | Circulatory system | 64 | 2 | ZC477.2,F38E11.8 | 0.236229415105054 | 0.022289328 | 0.1319578 | http://www.kegg.jp/kegg-bin/show\_pathway?ko04270/ | 0.03125 |
| ko04113 | Meiosis - yeast | Cell growth and death | 65 | 2 | ZC477.2,F38E11.8 | 0.23992049971607 | 0.022949184 | 0.1319578 | http://www.kegg.jp/kegg-bin/show\_pathway?ko04113/ | 0.0307692307692308 |
| ko03015 | mRNA surveillance pathway | Translation | 82 | 2 | ZC477.2,F38E11.8 | 0.30266893810335 | 0.035370325 | 0.154618 | http://www.kegg.jp/kegg-bin/show\_pathway?ko03015/ | 0.024390243902439 |
| ko04390 | Hippo signaling pathway | Signal transduction | 79 | 2 | ZC477.2,F38E11.8 | 0.291595684270301 | 0.033019198 | 0.154618 | http://www.kegg.jp/kegg-bin/show\_pathway?ko04390/ | 0.0253164556962025 |
| … | … | … | … | … | … | … | … | … | … | … |

… … … … … … … … … … …

… … … … … … … … … … …

… … … … … … … … … … …

  
  

| 表头 | 表头说明 |
| --- | --- |
| Pathway\_ID | Pathway标示 |
| Pathway\_name | Pathway名称 |
| Pathway\_class | Pathway分类名 |
| Annotated | 注释在这个pathway且在背景基因列表中的基因总数 |
| Significant | 注释在这个pathway且在候选基因列表中的基因观察总数 |
| SymbolList | 注释在这个pathway且在候选基因列表中的基因列表 |
| Expected | 候选基因列表注释在这个KEGG的期望值(候选基因总数与在背景基因中属于这个KEGG注释基因的比例相乘) |
| classic\_fisher | Fisher精确检验计算的P值 |
| FDR | 原始P值经Benjamini & Hochberg多重检验纠正后的P值 |
| WebSite | 显著富积KEGG pathway图链接地址， 点击可以看见红色方框代表上调基因，蓝色代表下调基因，否则红色方框代表miRNA或者lncRNA靶标基因， 差异环状RNA来源基因。 |
| rich\_factor | 富集因子，该值等于Significant一列基因数与Annotated一列基因数的比值。 |

  

### 显著富集KEGG柱状(KEGG\_enrichment\_Pvalue\_Barplot.png)

  

注：显著富集KEGG pathway柱状图，横坐标代表显著富集的KEGG pathway名称，纵坐标代表-Log10(Pvalue)。纵坐标越显著表示该Pathway越富集显著，红色柱表示显著的Pathway通路(Pvalue <= 0.05)，蓝色柱表示不显著的pathway通路。

  

### 显著富集KEGG气泡图（KEGG\_enrichment\_2D.png）

  

注：显著富集KEGG pathway气泡图，横坐标代表Rich factor(候选基因集属于这个KEGG pathway的基因总数与所有基因属于这个KEGG pathway的基因总数的比值)，Rich factor越大,表示富集的程度越大,纵坐标代表通路名称。根据Rich factor的排序信息，显示最前面20条KEGG pathway。

  

### 百分比排序柱状图（KEGG\_enrichment\_GenePercentage\_Barplot.png）

  

注：根据-Log10(Pvalue)的排序信息，显示最前面20条KEGG Pathway的柱状图，横坐标代表候选基因集属于这个KEGG的基因总数与候选基因集总数的比值)，柱状图上数字表示候选基因集属于这个KEGG的基因总数。

  

### KEGG与候选基因的网络图（KEGG\_enrichment\_KEGG2Symbol\_Net.png）

  

注：根据-Log10(Pvalue)的排序信息，显示最前面20条KEGG Pathway与候选基因的网络图，节点大小表示属于KEGG的候选基因总数。

  

### KEGG与KEGG的网络图（KEGG\_enrichment\_Pathway2Pathway\_Net.png）

  

注：根据-Log10(Pvalue)的排序信息，显示最前面20条KEGG Pathway与Pathway的网络图，重叠基因数与两者唯一基因数的比值大于等于20%，节点大小表示属于KEGG的候选基因总数，颜色表示-Log10(Pvalue)。。

  
  


## 7.5 蛋白质互作分析

目录链接

  
  
  
  

从STRING蛋白质相互作用网络数据库中查询和下载该物种的蛋白质相互作用网络，从而构建差异 表达基因蛋白质相互作用网络，如果测序物种在STRING 数据库中不存在，那么下载近源物种的蛋白质相互作用网络，采用Blastx或者Blastp将该物种的基因与近源物种的蛋白质序列库进行同源比对，通过将该物种基因序列编号映射于近源物种的蛋白质相互作用网络，映射的方法是如果该物种基因A 的近源物种同源基因列表中的1个或者多个与该物种基因B的近源物种同源基因列表中的1个或者多个存在蛋白质相互作用关系，那么认为基因A 与基因B存在可能的蛋白质相互作用，从而构建差异基因蛋白质相互作用网络。由于目的基因数目较多，选取度排序前50个基因来绘制蛋白质相互作用网络图。客户可用cytoscape 软件将差异基因对整个网络文件导入根据需要进行网络可视化。

全部差异基因的蛋白质互作分析结果见报告-> 5-FUN/\*\_vs\_\*/GO\_KEGG/string\*

上调的差异基因的蛋白质互作分析结果见报告-> 5-FUN/\*\_vs\_\*/GO\_KEGG\_up/string\*

下调的差异基因的蛋白质互作分析结果见报告-> 5-FUN/\*\_vs\_\*/GO\_KEGG\_Down/string\*

### 目的基因蛋白质相互作用网络图（strings\_network.png）

  

注：该图展示了基因间的蛋白互作关系，点越大说明与之存在相互作用的基因越多。

  
  
  

  
  
  
  
  
  

  
  


# 9. 分析方法与问题答疑

## 9.1 方法和材料

目录链接

  
  
  
  

为方便客户撰写文章，我们准备了数据分析所设计到的中文版methods和英文版methods，可以点击下载，也可以在analysis\_report/GN-method中找到。

  


## 9.2 F&Q

**1、什么是转录组测序？**

答：通过高通量测序，全面快速地获得特定组织或细胞在某一功能状态下所有转录本的序列信息。主要包括mRNA-seq、lncRNA-seq、circRNA-seq、miRNA-seq。通常所说的转录组测序，是指mRNA-seq。

转录组测序的步骤：样本采集、RNA抽提质检、构建文库、测序、生信分析及数据挖掘。

**2、转录组测序包括哪些？**

答：包括编码RNA测序和非编码RNA测序。编码RNA主要指mRNA，非编码RNA主要包括lncRNA-seq、circRNA-seq、miRNA-seq。对同一组样本同时进行这四种RNA测序分析，就构成了我们常说的全转录组测序。

**3、有参和无参转录组分别是什么？**

答：有参转录组，是指研究的物种已经有参考基因组及完整的注释信息。无参转录组，即指研究的物种目前还没有完整的参考基因组或注释信息。对于无参考基因组的物种，目前不提供相关服务。

**4、什么是链特异性建库？链特异性建库的优势有哪些？**

答：在合成第二链cDNA时，使用dUTP代替dTTP，加上接头后，加入UDG酶处理，将第二链cDNA消化，再进行后续的上机测序。这样就只保留了一条cDNA链的信息。

优势：（1）比对率更高；

（2）得到的基因数目更准确；

（3）定量结果更加准确：在差异分析时可以区分正义链、反义链来源的转录本，因而得到更加准确的定量结果；

（4）丰富度更高：保留了RNA的方向信息，可以获得更加丰富的转录本信息。

（5）由于lncRNA与mRNA有时只是正负链的差别，即lncRNA是反义转录本，所以对于lncRNA-seq必须采用链特异性建库。

我司的mRNA和lncRNA测序均采用链特异性建库，获得更准确的RNA定量信息。

**5、mapping率比较低的原因有哪些？**

答：如果出现比对率特别低的情况，可能出现的原因有：

1）参考基因组的物种不是样本来源的物种；

2）参考基因注释不完整；

3）Total RNA质量不好，有降解，得到的序列存在大量短片段的序列，分析时这些短片段序列会被过滤，造成比对率低；

4）样本污染，包括细菌、霉菌、病毒以及支原体污染。

5）物种混淆或多物种样本（PDX：人和鼠）

**6、显著差异基因的筛选标准?**

答：采用Deseq2来对差异基因进行分析，筛选标准是Pvalue≤0.05和|log2FoldChange|≥1。对于没有组内重复的样本，可以只考虑差异倍数，而不用考虑p值。

**7、转录本水平与基因水平表达量有什么不同？**

答：一条基因形成的前体RNA可通过可变剪切形成多种转录本；转录本水平的表达量是指定转录本的表达量；基因水平的表达量是指该基因所有转录本的表达量总和。

**8、差异表达基因的FDR有何意义，它和p-value有什么关系？**

答：测序完成后，往往能得到上百或上千个差异表达基因，对每个差异基因进行扩大样本qPCR验证似乎不太现实，通常会选取差异倍数越大，p值或FDR值越小的基因进行优先验证。但p值和FDR值究竟有什么统计学意义呢，它们间又有什么联系呢？

假如通过差异比较分析发现，某个基因A在两组样本间差异p-value小于0.05,我们知道任何一种测量手段都可能存在误差，那么基因A是存在真实差异还是测量误差，p-value值小于0.05的意思就是基因A不存在差异的概率小于0.05，换言之测量的随机误差小于0.05，但这个判断还是有0.05的犯错概率，就里就是假阳性率（False positive rate），但这只是一次判断，FDR值计算过程则是对p-value的多次判断校正即多重检验，降低假阳性率。RNA-seq分析中普遍采用BH（Benjamini and Hochberg）多重检验校正法，通过FDR法可以得到每个基因p-value校正后的q-value，通常FDR、Q value、Adjusted p-value是指同一个东西。FDR值比p-value更严格，数值越小越可靠，但没有约定的阈值，不像p-value小于0.05和0.01时才认为差异显著和差异非常显著

**9、什么是富集分析？**

答：就是已知很多个基因功能或代谢通路，把差异基因分别归到不同的基因功能或代谢通路中去。

**10、GO、KEGG分析中的p值表示什么？p值不显著的基因还能做后续研究吗？**

答：功能富集分析，p值只代表这些差异基因在某个生物学功能或代谢通路中的集中程度。用于GO、KEGG分析的基因已经是差异基因，p值不显著，只是说明这些差异基因不集中分布于某个生物学功能或代谢通路，并不影响差异基因的分析。

  


## 9.3 参考文献

[1] Dobin A, Davis CA, Schlesinger F, et al. STAR: ultrafast universal RNA-seq aligner[J]. Bioinformatics, 2012, 29(1): 15-21.

[2] Langmead B, Trapnell C, Pop M, et al. Ultrafast and memory-efficient alignment of short DNA sequences to the human genome[J]. Genome Biology, 2009, 10(3): R25.

[3] Wang L, Wang S, Li W. RSeQC: Quality Control of RNA-seq experiments[J]. Bioinformatics, 2012, 28(16): 2184.

[4] Trapnell C, Williams BA, Pertea G, et al. Transcript assembly and quantification by RNA-Seq reveals unannotated transcripts and isoform switching during cell differentiation[J]. Nature Biotechnology, 2010, 28(5): 511.

[5] Roberts A, Trapnell C, Donaghey J, et al. Improving RNA-Seq expression estimates by correcting for fragment bias[J]. Genome Biology, 2011, 12(3): R22.

[6] Roberts A, Pimentel H, Trapnell C, et al. Identification of novel transcripts in annotated genomes using RNA-Seq[J]. Bioinformatics, 2011, 27(17): 2325.

[7] Sun L, Luo H, Bu D, et al. Utilizing sequence intrinsic composition to classify protein-coding and long non-coding transcripts[J]. Nucleic Acids Research, 2013, 41(17): e166.

[8] Roberts A, Pimentel H, Trapnell C, et al. Identification of novel transcripts in annotated genomes using RNA-Seq[J]. Bioinformatics, 2011, 27(17): 2325.

[9] Li A, Zhang J, Zhou Z. PLEK: a tool for predicting long non-coding RNAs and messenger RNAs based on an improved k-mer scheme[J]. BMC Bioinformatics, 2014, 15: 311.

[10] Sun L, Zhang Z, Bailey TL, et al. Prediction of novel long non-coding RNAs based on RNA-Seq data of mouse Klf1 knockout study[J]. BMC Bioinformatics, 2012, 13: 331.

[11] Wang L, Park HJ, Dasari S, et al. CPAT: Coding-Potential Assessment Tool using an alignment-free logistic regression model[J]. Nucleic Acids Research, 2013, 41(6): e74.

[12] Zhang XO, Dong R, Zhang Y, et al. Diverse alternative back-splicing and alternative splicing landscape of circular RNAs[J]. Genome Res, 2016, 26(9): 1277-1287.

[13] Shen S, Park JW, Lu ZX, et al. rMATS: Robust and flexible detection of differential alternative splicing from replicate RNA-Seq data[J]. PNAS, 2014, 111(51): E5593.

  


## 9.4 参考数据库

**Strings**

http://string-db.org

**KEGG Pathway**

http://www.genome.jp

**Uniprot**

http://www.uniprot.org/downloads

**DisGeNET**

http://www.disgenet.org

**MSigDB**

http://software.broadinstitute.org/gsea/msigdb/index.jsp

  


## 9.5 相关软件

**skewer(v0.2.2)**

https://sourceforge.net/projects/skewer/files/?source=navbar

**FastQC(v0.11.5)**

http://www.bioinformatics.babraham.ac.uk/projects/fastqc

**bwa(0.7.10)**

http://bio-bwa.sourceforge.net

**STAR(2.5.3a)**

https://github.com/alexdobin/STAR

**Samtools(1.3.1)**

http://samtools.sourceforge.net

**RSeQC(v2.6.4)**

https://sourceforge.net/projects/rseqc

**StringTie(v1.3.1c)**

http://ccb.jhu.edu/software/stringtie

**gffcompare(0.9.9)**

https://github.com/gpertea/gffcompare

**DESeq2(v1.16.1)**

https://bioconductor.org/packages/release/bioc/html/DESeq2.html

**pheatmap**

https://cran.r-project.org/web/packages/pheatmap/index.html

**rMATS(v3.2.5)**

http://rnaseq-mats.sourceforge.net

**TopGO**

http://www.bioconductor.org/packages/release/bioc/html/topGO.html

**igraph**

https://cran.r-project.org/web/packages/igraph/index.html

**picard-tools(2.6.0)**

http://broadinstitute.github.io/picard

**TransDecoder(3.0.1)**

https://github.com/TransDecoder/TransDecoder/wiki

**Blast(2.5.0)**

ftp://ftp.ncbi.nlm.nih.gov/blast/executables/blast+/LATEST

**WGCNA(1.61)**

https://cran.r-project.org/web/packages/WGCNA/index.html

**Mfuzz(2.36.0)**

http://www.bioconductor.org/packages/release/bioc/html/Mfuzz.html

 顶端 ↑
