# Supplementary figures and images for: The pivotal regulatory role of the FEV-SLC7A11 axis in ferroptosis elucidates the anti-aging mechanism of β-sitosterol in a cross-species study
Source: Front Pharmacol. 2025 Aug 7;16:1600489. doi: 10.3389/fphar.2025.1600489 (PMC12368974; doi:10.3389/fphar.2025.1600489)

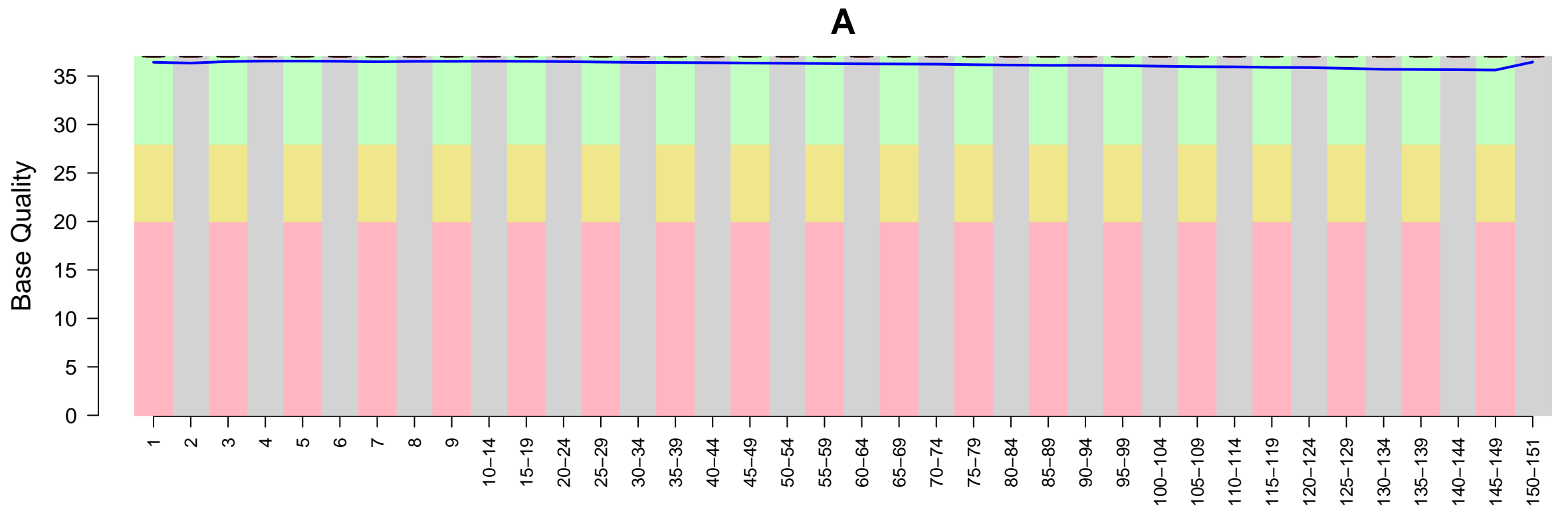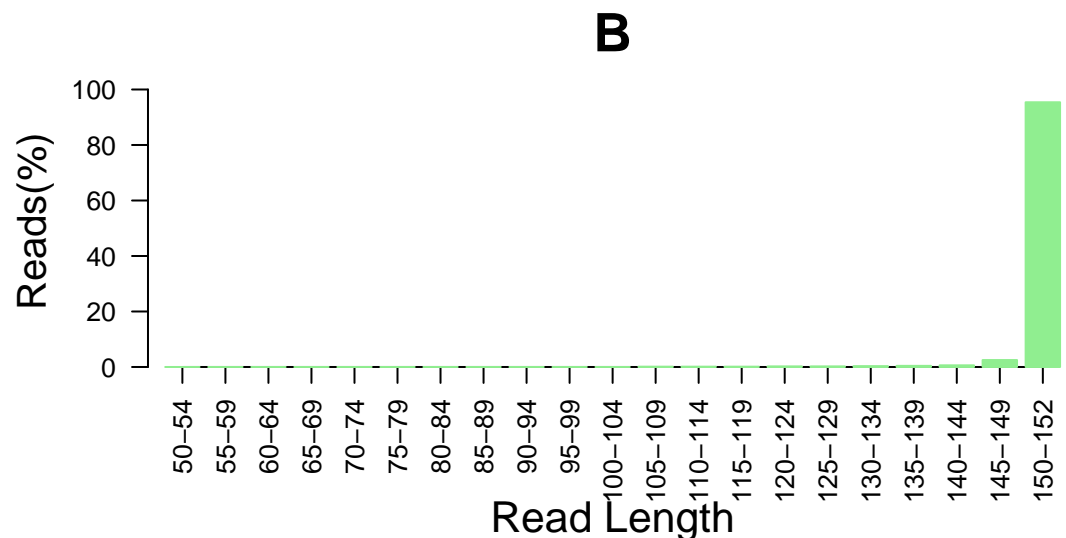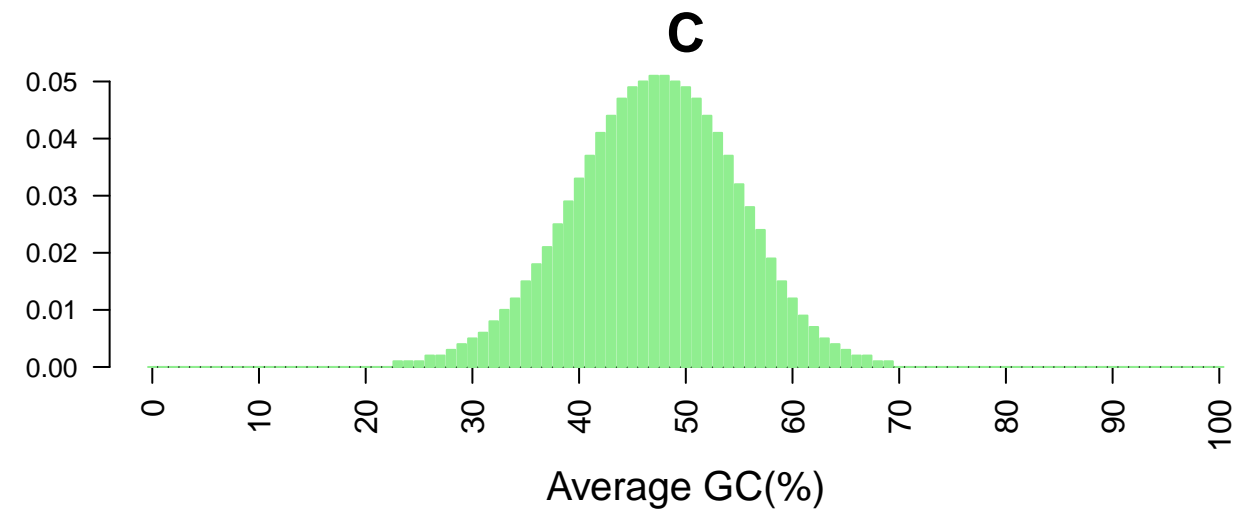

Supplement: Supplementary file 2 [file DataSheet1.zip › Read1_fastqc_data_per_base_quality.pdf]

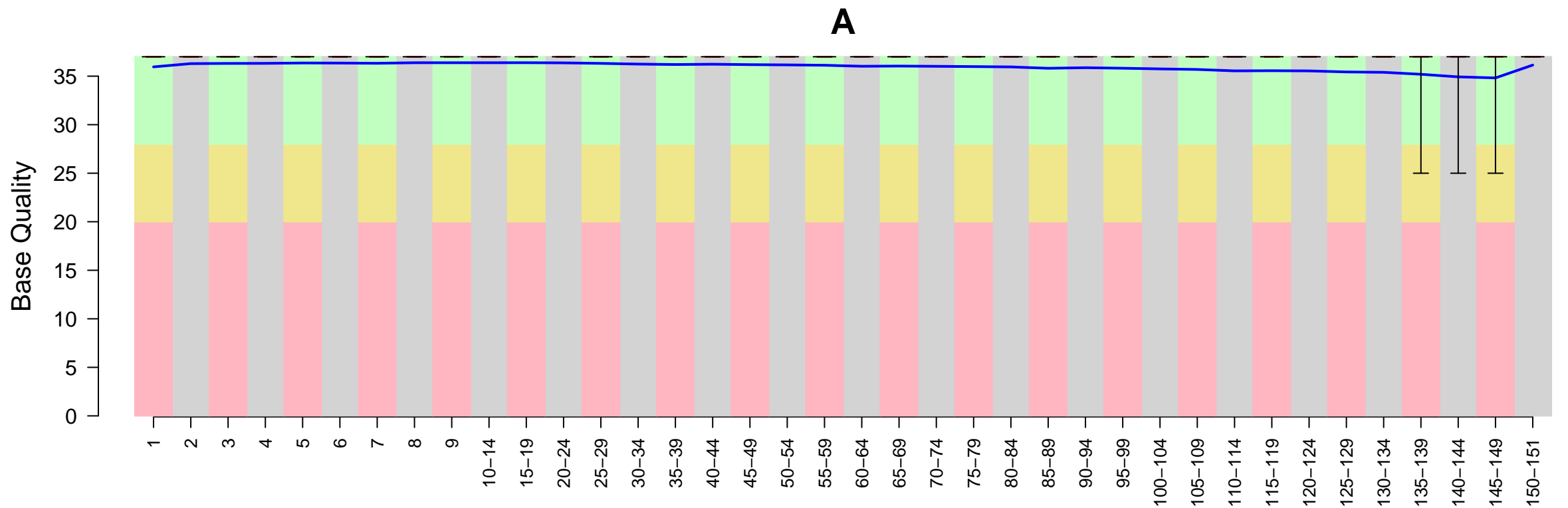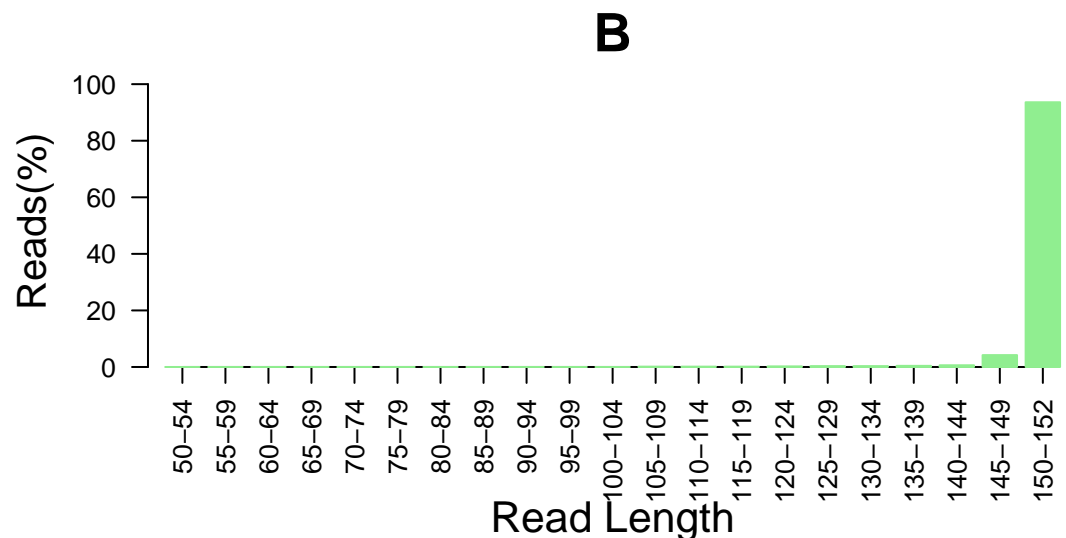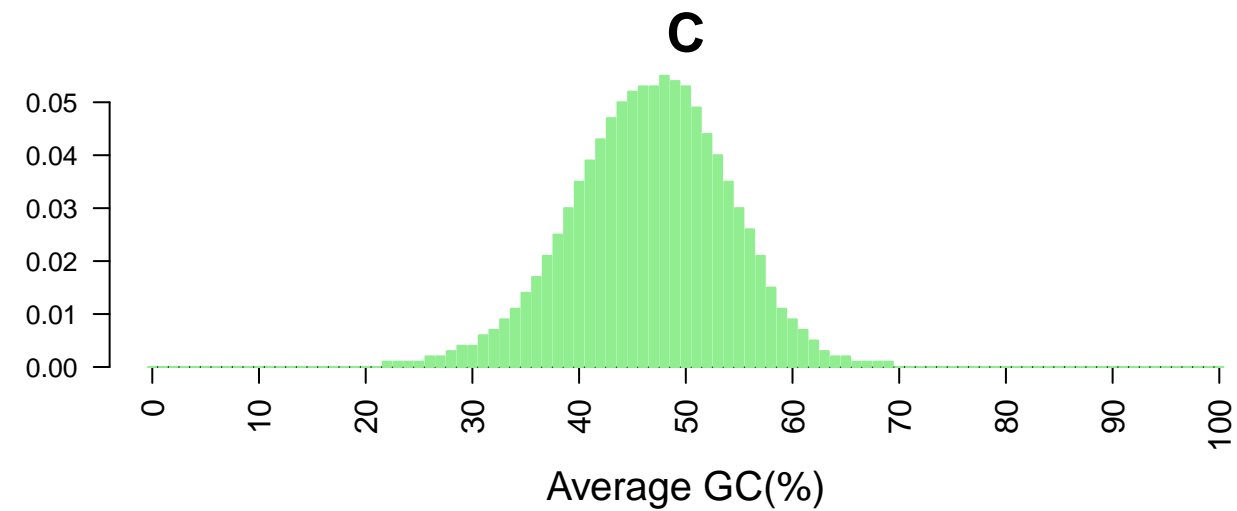

Supplement: Supplementary file 2 [file DataSheet1.zip › Read2_fastqc_data_per_base_quality.pdf]
